# Rosmarinic Acid Present in *Lepechinia floribunda* and *Lepechinia meyenii* as a Potent Inhibitor of the Adenylyl Cyclase gNC1 from *Giardia lamblia*

**DOI:** 10.3390/plants13050646

**Published:** 2024-02-26

**Authors:** Adolfo Zurita, Esteban Vega Hissi, Agostina Cianci Romero, Adela María Luján, Sofía Salido, Agustín Yaneff, Carlos Davio, Justo Cobo, María Cecilia Carpinella, Ricardo Daniel Enriz

**Affiliations:** 1 Facultad de Química, Bioquímica y Farmacia, Universidad Nacional de San Luis, Instituto Multidisciplinario de Investigaciones Biológicas (IMIBIO-SL), Ejército de los Andes 950, San Luis 5700, Argentina; egvega@gmail.com (E.V.H.); agostinacianci.acr@gmail.com (A.C.R.); 2 Laboratorio de Química Fina y Productos Naturales, Centro de Investigación y Desarrollo en Inmunología y Enfermedades Infecciosas (CIDIE) CONICET-UCC, Universidad Católica de Córdoba, Avda. Armada Argentina 3555, Córdoba X5016DHK, Argentina; adem.lujan@gmail.com; 3 Departamento de Química Inorgánica y Orgánica, Universidad de Jaén, Campus Las Lagunillas s/n, 23071 Jaén, Spain; ssalido@ujaen.es (S.S.); jcobo@ujaen.es (J.C.); 4 Instituto de Investigaciones Farmacológicas (ININFA-UBA-CONICET), Facultad de Farmacia y Bioquímica, Universidad de Buenos Aires, Junín 956, Buenos Aires C1113AAD, Argentina; agustinyaneff@hotmail.com (A.Y.); carlosdavio@hotmail.com (C.D.)

**Keywords:** adenylyl cyclase inhibitors, *Giardia lamblia*, rosmarinic acid, *Lepechinia floribunda*, *Lepechinia meyenii*, molecular modeling

## Abstract

Giardiasis is a parasitosis caused by *Giardia lamblia* with significant epidemiological and clinical importance due to its high prevalence and pathogenicity. The lack of optimal therapies for treating this parasite makes the development of new effective chemical entities an urgent need. In the search for new inhibitors of the adenylyl cyclase gNC1 obtained from *G. lamblia*, 14 extracts from Argentinian native plants were screened. *Lepechinia floribunda* and *L. meyenii* extracts exhibited the highest gNC1 inhibitory activity, with IC_50_ values of 9 and 31 µg/mL, respectively. In silico studies showed rosmarinic acid, a hydroxycinnamic acid present in both mentioned species, to be a promising anti-gNC1 compound. This result was confirmed experimentally, with rosmarinic acid showing an IC_50_ value of 10.1 µM. Theoretical and experimental findings elucidate the molecular-level mechanism of rosmarinic acid, pinpointing the key interactions stabilizing the compound–enzyme complex and the binding site. These results strongly support that rosmarinic acid is a promising scaffold for developing novel compounds with inhibitory activity against gNC1, which could serve as potential therapeutic agents to treat giardiasis.

## 1. Introduction

*Giardia lamblia* is the etiological agent responsible for giardiasis, a parasitic infection with high global distribution that affects humans of all ages [1,2] as well as a diverse array of birds and mammals, including livestock, wildlife, and domestic pets [3,4]. In both humans and animals, the disease manifests with clinical symptoms ranging from asymptomatic cases to the development of gastrointestinal discomfort, including nausea, vomiting, general malaise, colic, diarrhea, and loss of weight [4,5,6,7].

Nowadays, giardiasis is treated with non-selective and frequently unsuitable chemotherapeutic agents [8]. This lack of secure and effective drugs, in addition to the absence of vaccines and the emergence of resistance to the most frequently used entity, metronidazole [8,9], represent significant challenges for controlling this infection. In this regard, the development of new chemical entities to counter this parasite is urgently required.

It is well known that phytotherapy is notable for treating a variety of diseases [10]. According to the World Health Organization (WHO), 88% of the population rely on traditional medicine, which mostly includes plant-derived products, for their primary form of health care (https://www.who.int/initiatives/who-global-traditional-medicine-centre (accessed on 17 November 2023)).

Among the different pharmacological uses of plants [11,12], the antiparasitic effect of species belonging to several botanical families [13,14,15], including those from the Argentinian flora [16,17,18], is of high relevance. However, as far as we know, there is scarce information about plants with activity against *G. lamblia*.

The enormous range of therapeutic applications of plants together with the improvement of the isolation techniques of active principles and of biological assays (alongside sophisticated in silico studies [19,20]) have allowed us to establish plant extracts as a prominent source of leading molecules that feed drug discovery pipelines [21,22]. The vast chemical diversity of the phytoconstituents is one of their major attributes resulting in efficacious therapeutic agents [19,23]. To address the success in the identification of these entities, biodiverse-rich flora is of great importance and must be accompanied by an effective mode of access to it.

The flora of Argentina is rich in diversity, with Argentina being one of the 25 most diverse countries worldwide [24]; however, it is far from being studied in depth [25,26]. The country possesses 9938 species [27] distributed across 274 families [24]. This rich botanical resource positions Argentinian plants as a remarkable resource for obtaining new molecules with different pharmacologic properties, including antiparasitic agents.

In a previous work, it was described that *G. lamblia* contains genes encoding two class III nucleotidyl cyclases named gNC1 and gNC2, which are responsible for the synthesis of cAMP from ATP, along with a phosphodiesterase (gPDE), [28] which is responsible for its degradation [29]. cAMP is a ubiquitous second messenger in eukaryotes and prokaryotes that plays a crucial role in intracellular signal transduction [30]. In eukaryotes, cAMP primarily activates protein kinase A (PKA) and the exchange protein activated by cAMP (Epac), which influence cellular processes like proliferation, differentiation, and apoptosis [31,32,33]. In this context, it was observed by in vitro assays that the excystation of *G. lamblia* cysts was significantly blocked when the PKA inhibitor, amide 14–22, was administered during the initial stages of excystation [34,35]. In fact, a notable rise in cAMP levels was observed during the early stages of trophozoite encystation as determined by in vitro methods [28], while increased trophozoite growth rates were observed when the parasite was exposed to the non-hydrolyzable cAMP analogue, di-butyryl-cAMP [36]. These findings underscore the relevance of cAMP in the growth and differentiation processes of *G. lamblia*, therefore highlighting the potential of gNC1 as a target for the future development of drugs to counter the parasite. In support of this statement, the inhibition of gNC1 suggests that it is selective. Despite the fact that this enzyme belongs to a widely distributed family of enzymes present in prokaryotes and eukaryotes [30,37], it exhibits an approximately 30% sequence identity with its homologs found in mammals [36], which potentially will result in a specific inhibition.

In a previous work, the catalytic domain of the enzyme gNC1 from *G. lamblia* (construct gNC1-301) was purified [28]. Subsequently, it was described that the enzymatic properties of this domain were similar to that of soluble adenylyl cyclase from mammals [28]. More recently, using the available structural data of gNC1, an in silico model was developed for this specific enzyme using homology techniques [36]. This was the first theoretical model reported for gNC1 from *G. lamblia*, which demonstrated the capability of the enzyme as a biological site to search for new ligands able to inhibit its activity. Using this in silico model and further validation by experimental techniques, a derivative of *N*-(piperidin-4-yl)-1H-pyrrole-2-carboxamide, named AMJ-147 (compound **8**), was identified as a new inhibitor of gNC1 [36].

Taking advantage of the in silico and experimental models previously developed and the encouraging results obtained [36], in the present work, we focused our efforts on the search for potential inhibitors of gNC1 from *G. lamblia* within extracts obtained mostly from native species of Argentina.

## 2. Results and Discussion

### 2.1. Plants from Central Argentina as Inhibitors of the Adenylyl Cyclase gNC1 from Giardia lamblia

**Figure 1:**
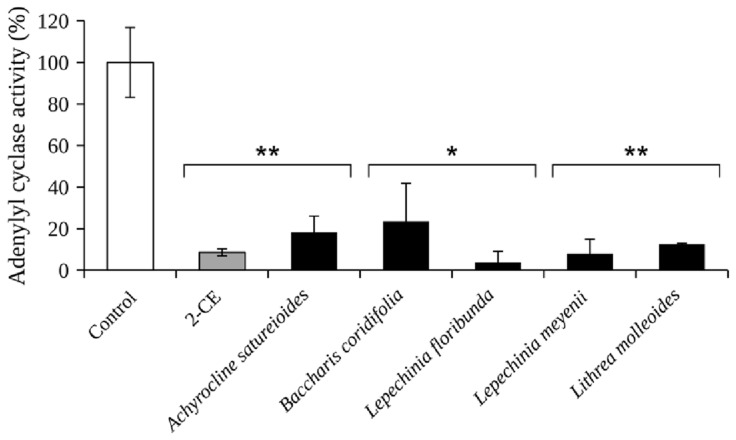
Adenylyl cyclase activity of the catalytic domain of gNC1-301 incubated with the target extracts at 250 μg/mL. 2-Catechol estrogen (2-CE) at 50 µM was used as a positive control. The data are representative of three different experiments. Significant differences between treatments and the negative control were determined by using the two-tailed paired Student’s *t*-test (** *p* < 0.01, * *p* < 0.05).

In order to find new sources of compounds with gNC1 inhibitory activity, 14 extracts obtained from species mostly native to Argentina (Table 1) were screened. As observed in Figure 1, extracts obtained from *Achyrocline satureioides*, *Baccharis coridifolia*, *L. floribunda*, *L. meyenii* and *Lithrea molleoides* proved to be effective in blocking the activity of the enzyme when applied at 250 µg/mL, with percentages of inhibition ranging from 76.5 to 96.2%. The extracts from *L. floribunda* and *L. meyenii* were the most potent, with inhibitory percentages higher than 92% and IC_50_ values of 9.4 and 31.3 µg/mL, respectively (Table 2).

### 2.2. Effect of Rosmarinic Acid (***3***) on the Activity of gNC1

**Figure 2:**
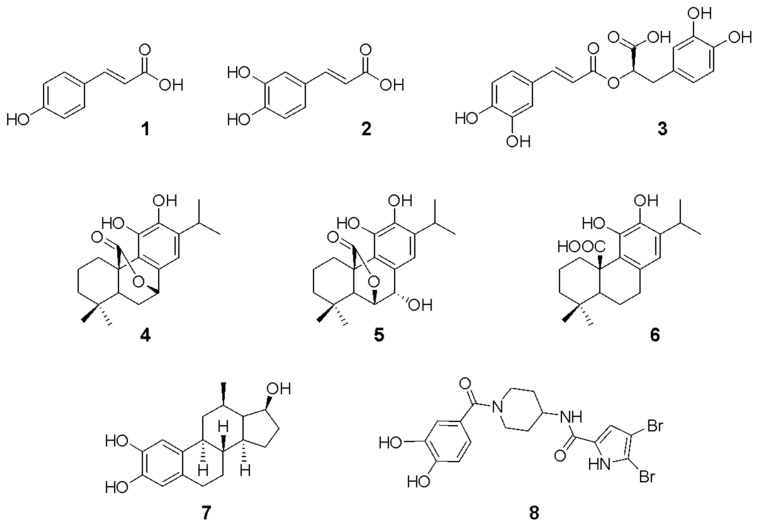
Chemical structures of of *p*-coumaric acid (**1**), caffeic acid (**2**), rosmarinic acid (**3**), carnosol (**4**), rosmanol (**5**), carnosic acid (**6**), 2-catechol estrogen (**7**) and AMJ-147 (**8**).

It is interesting to note that both the reference compound 2-catechol estrogen (2-CE, compound **7**, Figure 2) and AMJ-147 (compound **8**, Figure 2) [36] bear a catechol group. In addition, the well-known inhibitors of class III adenylyl cyclase domain tyrphostins [38], LRE1 [39], (4-aminofurazan-3-yl)-[3-(1H-benzoimidazol-2-ylmethoxy)phenyl] methanone [40], and bisphenol bithionol [41] display bioisosteric groups of catechol. This information suggests that the catechol moiety is important for anti-gNC1 activity.

Previous studies have demonstrated the presence of *p*-coumaric acid (**1**), caffeic acid (**2**) and rosmarinic acid (**3**) (Figure 2) in *L. meyenii*, which were highly effective for inhibiting the enzyme tyrosinase [42]. In addition, *L. meyenii* demonstrated an antibacterial effect, which was attributed to the presence of the diterpenes carnosol (**4**), rosmanol (**5**), and carnosic acid (**6**) (Figure 2) [11].

The structure **1** and the structures **2**–**6** bearing a catechol group (Figure 2) were submitted to a preliminary and exploratory in silico study to determine their potential inhibitory activity on gNC1. Such analyses were carried out by using combined techniques of docking and molecular dynamics (MD) simulations. In addition, the reference compound **7** was included in the docking simulations as a control to ensure robust evaluation of the results.

Docking analysis indicated that all compounds under study **1**–**6** were able to bind into the active site of gNC1, suggesting a competitive inhibitory mechanism. Therefore, standard score criteria was applied to differentiate their binding modes. The docking scores obtained from AutoDock Vina for compounds **1** to **6** provided valuable insights into their binding affinity and the potential interactions with the target receptor.

**Figure 3:**
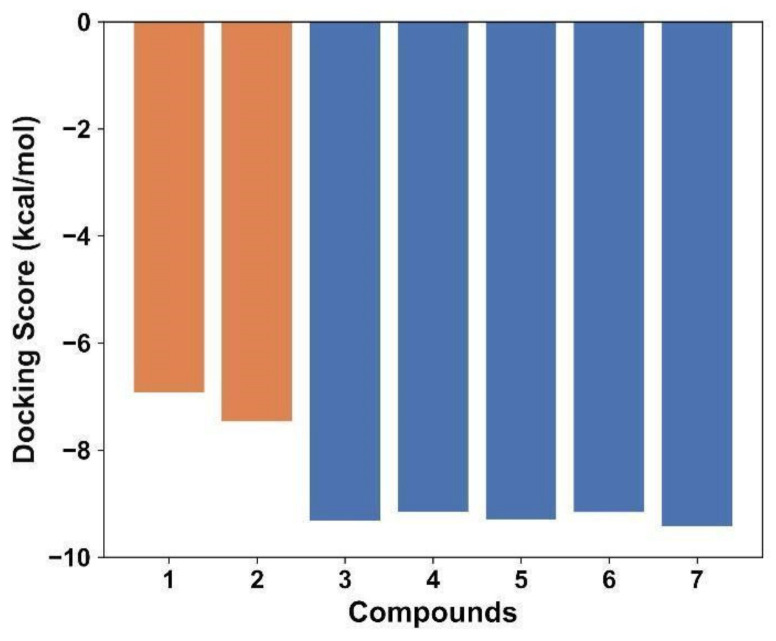
Histogram of the scores from the best docking poses of compounds **1**–**6**. The reference compound **7** was included as control. Bars colored in orange represent compounds with lower scores that were not further studied and bars colored in blue represent compounds with higher scores.

The scores shown in Figure 3 for compounds **1**–**6** corresponding to −6.923, −7.465, −9.318, −9.146, −9.291 and −9.157, respectively, evidenced that all of these exhibited relatively high negative values, indicating favorable binding affinities. The docking scores of compounds **3**–**6** fell within a similar range, indicating a relatively stronger and comparable binding affinity between them and with the reference compound, whereas compounds **1** and **2** displayed significantly weaker indices. Since docking scores alone do not provide a complete picture of the compound’s binding features (and in accordance with the results obtained), compounds **3**–**6** were selected for further MD simulations.

Root mean square fluctuation (RMSF) values indicate the degree of flexibility or fluctuation of atoms within a molecule during an MD simulation.

**Figure 4:**
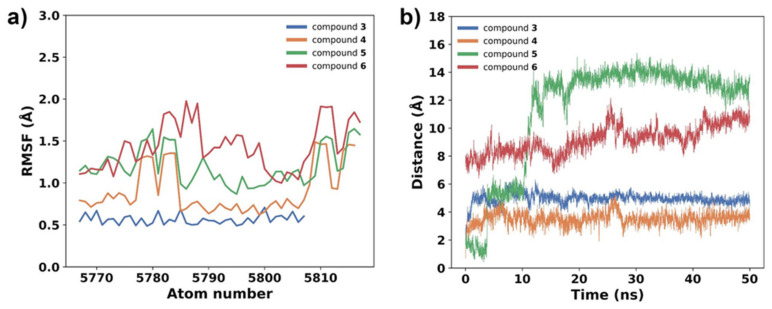
Stability evaluation of the docking complexes. (**a**) Root mean square fluctuations (RMSF) of the compound’s atoms. (**b**) Distance between the docked compound and its position along the MD simulation.

**Figure 5:**
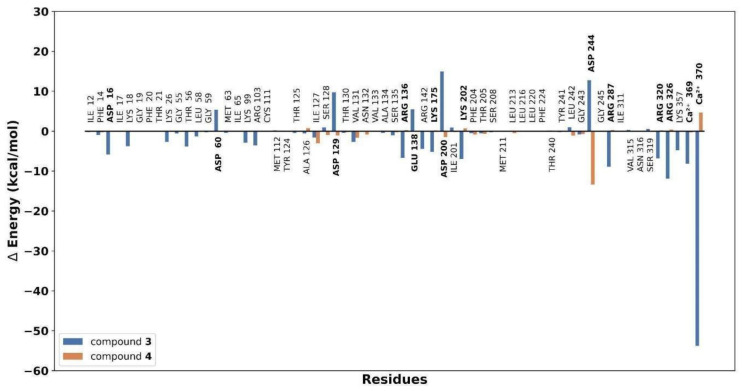
Binding energy decomposition plot showing the contribution of individual residues of the active site to the overall binding energy of the gNC1/compound complex.

As observed in Figure 4a, compounds **3** and **4** have closely related behaviors throughout the simulations, exhibiting reduced flexibility (low RMSF values), which may imply a more constrained conformational space for these metabolites, potentially leading to specific and stable interactions with the active site of gNC1. In contrast, the higher RMSF values observed for compounds **5** and **6** (Figure 4a) showed greater flexibility, with their atoms exhibiting larger fluctuations. These findings are consistent with a greater solvent exposure, indicating a potential influence of the solvent environment on the conformational dynamics of compounds **5** and **6**. The results obtained suggest that these entities are more exposed to solvent molecules, probably outside the active site. In fact, during MD simulations, the docked compounds departed from the active site, as is evidenced by an increase in the measured distance between its initial docked position and its position during the simulation (Figure 4b). This departure showed a potential instability or a weaker binding interaction between the ligand and the receptor. These preliminary results indicated that compounds **3** and **4** might be the most interesting ligands, and therefore these molecules were further analyzed by employing an analysis per residue for the complexes of compound **3**/gNC1 and compound **4**/gNC1. This study allowed us to evaluate in more detail which amino acids would be involved in the formation of the respective complexes, as well as the putative strength of such interactions. These results are presented in Figure 5.

Upon analysis, the strongest interaction observed in the complex of compound **3**/gNC1 compared to compound **4**/gNC1 arises from the formation of an ionic bond between the carboxylate group of compound **3** and a structural cation, specifically calcium. The presence of Arg136 and Lys202 helped to balance the electrostatic interactions within the active site, leading to a more favorable binding environment for compound **3**. Nevertheless, it should be noted that there are repulsive interactions between compound **3** and the acidic side chains present in the active site of the enzyme, which can hinder the binding affinity and disturb the stability of the compound **3**/gNC1 complex. On the other hand, analyzing the **4**/gNC1 complex, the absence of an ionic bond in this complex suggests that this structure interacted with gNC1 through a different mechanism compared to compound **3** or at least by different types of interactions. In fact, compound **4** establishes strong hydrogen bonds with an Asp244 side chain.

**Figure 6:**
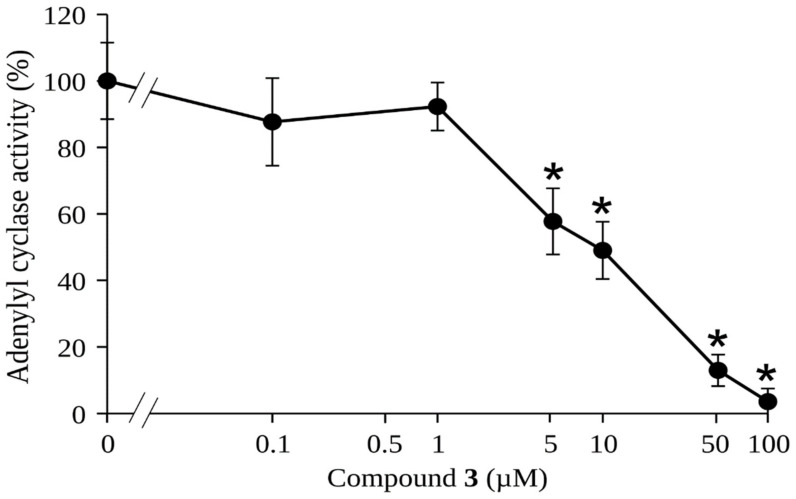
Determination of adenylyl cyclase activity of the catalytic domain of gNC1 incubated in the presence of the indicated concentrations of compound **3** expressed on a log scale. Significant differences between treatments and the negative control were determined by using the two-tailed paired Student’s *t*-test (* *p* < 0.001). Data are representative of three independent experiments.

The combined study using docking and MD simulations predicts that compounds **3** and compound **4** might be the best candidates to bind properly to the gNC1 binding site; however, the analysis per residue indicated that compound **3** exhibited stronger interactions with the target enzyme, suggesting a higher likelihood of being an effective inhibitor. Although our exploratory study does not allow us to rule out the possibility that compound **4** shows inhibitory activity on gNC1, it clearly indicates that compound **3** is the most promising. According to this, compound **3** was subjected to a gNC1 inhibitory assay in order to experimentally confirm the in silico findings. The results revealed compound **3** as a promising anti-gNC1 entity (Figure 6) with an IC_50_ value of 10.1 ± 1.3 µM.

As observed in Figure 1 and Table 2, both *L. floribunda* and *L. meyenii* proved to be highly effective in inhibiting gNC1. To determine whether the activity of the first species was also attributed to compound **3**, the presence of this metabolite was determined and quantified by HPLC. Its yield expressed in mg per 100 g of *L. floribunda* extract was 6647.3 mg, while in *L. meyenii*, the yield was 32.7 mg per 100 g of extract [42]. Although there is not a proportional relationship between the quantities of compound **3** present in each extract and their activity (see Table 2), this finding would explain the highest effectiveness exerted by *L. floribunda*. Compound **3** is a hydroxycinnamic acid highly distributed in numerous species [43,44], many of which are used in foodstuffs [45] or possess medicinal and cosmetic value [43,46,47], such as *Salvia rosmarinus* (homotypic synonym, *Rosmarinus officinalis*), *Melissa officinalis*, *Origanum* spp., *Salvia officinalis* and others [43,44,48]. Among the botanical families containing compound **3**, Lamiaceae is the most prominent, with abundant yields of this compound [49]. This ample presence of significant quantities of compound **3** in species with wide human consumption suggests a great number of sources of the molecule and the safe use of the products containing it. In connection with the latter, it is important to mention that in a previous study, a rosmarinic acid concentrate extract was considered GRAS (generally recognized as safe) as determined by tests employed in the food industry [50]. On the other hand, the compound was submitted to clinical trials at two opportunities, with the aim of treating atopic dermatitis and allergic rhinoconjunctivitis [43,51].

### 2.3. Molecular Modeling

With the aim of deepening our understanding of the interaction between compound **3** and gNC1, further simulations were performed following the same computational techniques and the same steps as those reported for 2-CE and compound **8** in a previous work [36] (see Methods section).

**Figure 7:**
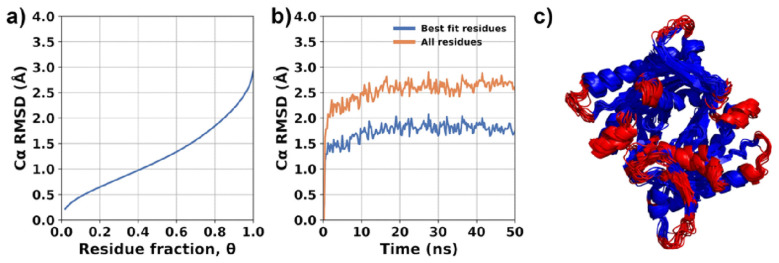
“RMSD_mdlovofit”. Flexibility analysis of compound **3**/gNC1 complex in molecular dynamics simulations. (**a**) Fractional alignment plot. Blue line represents the RMSD value as a function of the fraction of the atoms (θ) considered in the alignment. (**b**) Time-dependent structural deviation; the RMSD of the 75% least mobile atoms is represented by the blue line, while the RMSD of all residues is denoted by the orange line. (**c**) Molecular superposition of aligned trajectory frames colored according to the algorithm automatic classification; least mobile regions are shown in blue and most mobile regions in red.

The evaluation of enzyme flexibility was conducted using MDLovoFit version 16.264. The analysis focused on studying the structural fluctuations of the enzyme by performing structural alignments on subsets of amino acids corresponding to different fractions (θ) of the full protein length. The results are shown in Figure 7. In these simulations, it was observed that a majority of the enzyme structure (75%) remained preserved, with a root mean square deviation (RMSD) value lesser than 2Å. The Cα RMSD plots specifically for θ = 0.75 are depicted in Figure 7b. These plots indicated that the enzyme structure was stabilized and did not show significant divergence from the initial structure. The majority of mobile atoms are situated within the loops and external regions of the enzyme. Furthermore, similar to our previous simulations [36], the presence of compound **3** in the active site was found to slightly destabilize the structure of the enzyme dimer. This destabilization resulted in increased atomic fluctuations, approximately to the same extent observed in the absence of the compound (*apo* form) and compared to 2-CE and compound **8** [36]. Despite the latter, interactions between the monomers and the overall secondary structure of the enzyme remained uncompromised. This indicates that while there may be some local structural changes or fluctuations induced by the compound, the overall integrity of the enzyme’s secondary structure, including its interactions between monomers, remained intact. To expand our analysis, the enzyme was further examined by modeling it with compound **3** bound to both active sites. Compound **3** was observed to bind to both active sites of the enzyme, adopting nearly the same “L-shaped” conformation in both instances.

**Figure 8:**
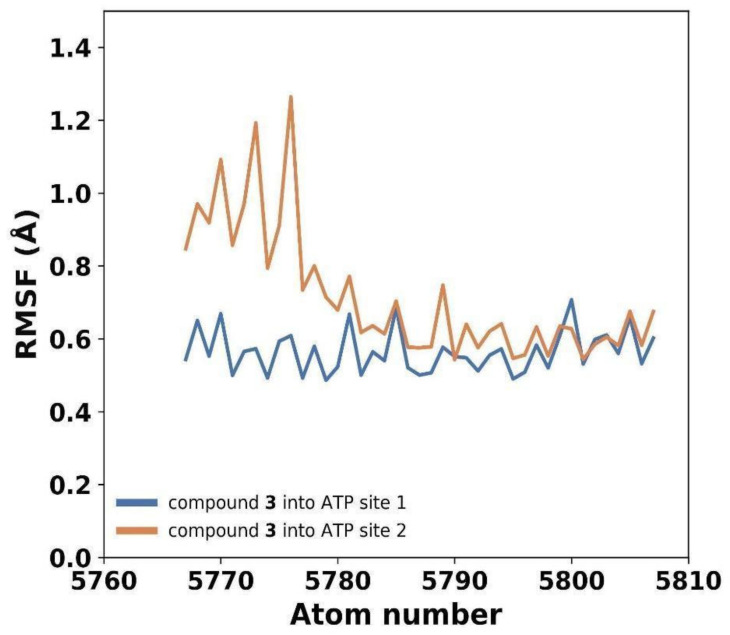
Atomic fluctuations of compound **3** bound to both gNC1 active sites.

**Figure 9:**
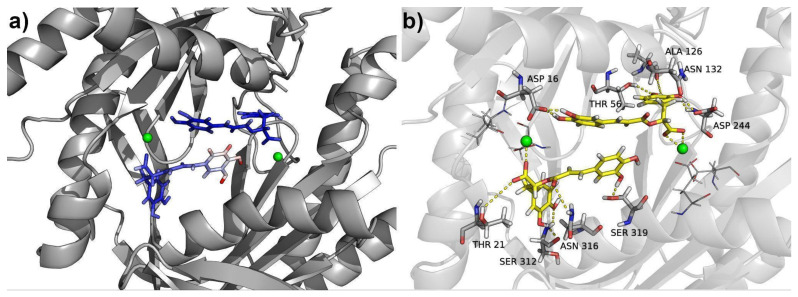
Molecular structure of compound **3**/gNC1 complex with both active sites occupied. (**a**) Compound **3** is shown as sticks colored according to the root mean square fluctuation (RMSF) values. (**b**) Polar interactions (hydrogen and ionic bonds). Calcium atoms are represented as green spheres. Interacting residues are depicted as gray sticks, while the molecules of compound **3** located into both ATP sites are shown as yellow sticks.

While the entire structure of compound **3** located in the ATP site 1 displayed minimal fluctuations, the compound bound to the other active site (ATP site 2) exhibited two distinct sets of atoms with slightly different RMSF values (Figure 8). The first set, comprising atoms involved in crucial interactions, exhibited low RMSF values, indicating a stable binding. However, the other part of the molecule displayed higher fluctuations, suggesting some degree of flexibility in that region (Figure 9a). Although compound **3** does not penetrate deeply into the ATP binding site of the enzyme, it effectively binds to the entrance of the active site, obstructing the binding of ATP molecules. This indicates that compound **3** acts through a competitive mechanism, as it competes with ATP for the access to the active site.

**Figure 10:**
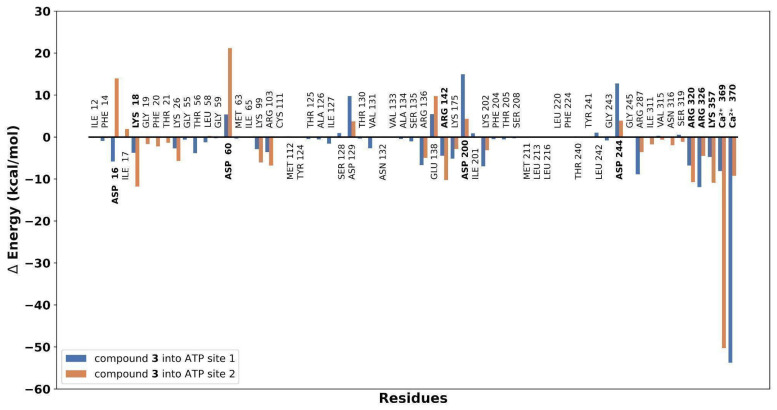
Residue decomposition analysis highlighting the most significant contributions to inhibitor binding at each active site.

The molecular simulations indicated that the primary interaction driving this binding is the ionic bond formed between the carboxylate group of compound **3** and the calcium cation of each active site, as was mentioned above. Furthermore, the presence of basic residues, mainly Lys and Arg, surrounding the entrance of the active site played a crucial role in stabilizing the binding, as observed in Figure 10. These residues contributed to the overall stability of the compound **3**/gNC1 complex.

In addition to the strong ionic interactions, multiple hydrogen bonds were observed to provide stability and, consequently, to improve the binding affinity of the compound. Most prominent hydrogen bonds are presented in Table 3 and Figure 9b. The cooperative effect of these hydrogen bonds, in conjunction with the ionic interaction (Figure 10), amplifies the overall stability of the complex and enhances the affinity of compound **3** for the enzyme’s active sites.

In summary, the molecular simulations performed predicted that compound **3** will bind to the active site of gNC1 from *G. lamblia* as a competitive inhibitor. To corroborate these results experimentally, the next stage was to study the mechanism of action of the target molecule.

### 2.4. Experimental Corroboration for the Mechanism of Action of Rosmarinic Acid (***3***)

**Figure 11:**
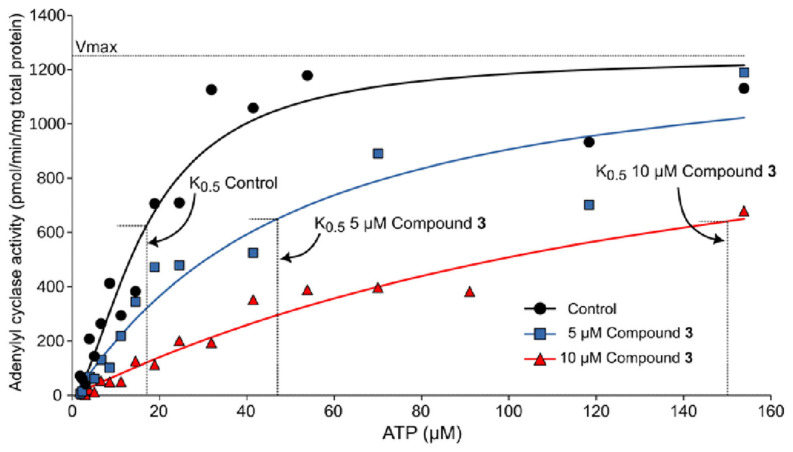
Inhibitory effect of rosmarinic acid (**3**) on gNC1 adenylyl cyclase activity. The assay was performed by increasing the concentrations of ATP in the presence of the dissolution solvent as a control (black filled circles), 5 µM of compound **3** (cyan filled squares), and 10 µM of compound **3** (red filled triangles). The values of K_0.5_, V*max* and Hill coefficient are indicated in Table 4. The data are representative of three different experiments with similar results.

In order to confirm the results obtained through the molecular simulation studies, assays were performed, aiming to determine the adenylyl cyclase activity of different concentrations of compound **3**. As shown in Figure 11 and Table 4, the best fit curve for the control reaction showed a K_0.5_ value of 17.0 ± 1.9 µM and a V*_max_* of 1249 ± 48 pmol of cAMP min^−1^ mg of total proteins^−1^, where at the same time, the K_0.5_ values of the reactions in the presence of 5 and 10 µM of compound **3** increased to 47 ± 8.9 µM and 150 ± 16.9 µM, respectively; their V*_max_* did not change significantly with respect to the control (1300 ± 100 and 1283 ± 93 cAMP min^−1^ mg of total protein^−1^, respectively). These results showed that compound **3** behaves as a competitive inhibitor of gNC1-301 activity by increasing the K_0.5_ value of the reaction without changing V*_max_*, as was predicted by molecular modeling.

## 3. Materials and Methods

### 3.1. Materials, Reagents and Equipment

ATP was purchased from Sigma-Aldrich (St Louis, MO, USA), while EDTA and rosmarinic acid, CAS number 20283-92-5 (96%), were obtained from Merck (Darmstadt, Germany). We obtained 2-CE from Abcam (Cambridge, UK) and dissolved it in dimethyl sulfoxide (DMSO, Sintorgan S.A, Buenos Aires, Argentina). All solvents were HPLC grade.

### 3.2. Plant Material and Extract Preparation

Plants (Table 1) were harvested from December to March in the hills of Córdoba Province, Argentina, between −30.773428 to −31.797760 latitude and −64.109384 to −64.546803 longitude. The species were chosen according to their availability, accessibility, and previous information regarding active principles with catechol moieties. The identification of the selected plants was confirmed by Ing. G. Ruiz, affiliated with the Marcelino Sayago Herbarium of the School of Agricultural Science, Universidad Católica de Córdoba, where a voucher of each plant was deposited and a UCCOR (Universidad Católica de Córdoba) number was given. After drying the aerial parts of each plant, these were grinded in a mechanical grinder and the resulting vegetal material was extracted by maceration with 96% ethanol at a proportion 3:1 for 48 h. The yield of each extract obtained after exhaustive solvent removal is expressed as percentage weight of plant material (Table 1).

### 3.3. Quantification of Rosmarinic Acid (***3***) in Lepechinia Floribunda

For the quantification of compound **3** in *L. floribunda*, HPLC-DAD was performed on a Shimadzu LC-10 AS (Shimadzu Corp., Tokyo, Japan), equipment with a Phenomenex Prodigy 5 μm ODS 250 × 4.6 mm reversed-phase column, followed by elution with 40% methanol in water acidified with perchloric acid (pH 2.4) as a mobile phase and UV detection at 210 nm and 320 nm. Compound **3** at 1 mg/mL and the extract at 20 mg/mL were prepared prior to use dissolved in ethanol.

### 3.4. Expression and Enrichment of gNC1 Catalytic Domain

The expression and enrichment of the catalytic domain of gNC1 enzyme (construct gNC1-301) was carried out as described by Saraullo [28].

### 3.5. Adenylyl Cyclase Inhibitory Assays

The reactions were performed by incubating 5 μL of the enriched enzyme gNC1-301 with 5 μL of each extract or compound **3** solutions to reach a maximum final concentration of 250 µg/mL or 50 µM, respectively, in a 50 μL final reaction mix containing 25 mM Tris-HCl pH 8.0, 4 mM MnCl_2_, 4 mM CaCl_2_ and 100 μM ATP (unless otherwise indicated) during 30 min at 32 °C. The reactions were stopped by the addition of 1 mL of 100% (*v*/*v*) ethanol, and the cAMP formed was quantified by a radiobinding protein assay. In all cases, the vegetal products were dissolved in DMSO just prior to use. A negative control test was performed with DMSO at 10%, since at this concentration, no significant effect on the enzymatic activity of gNC1 was observed. Simultaneously, 2-CE was used as a positive control. All experiments were repeated at least three times.

### 3.6. cAMP Radiobinding Protein (RBP) Assay

In all cases, ethanol from each adenylyl cyclase assay sample was evaporated in a water bath, and residues resuspended in a radiobinding protein (RBP) buffer (50 mM TrisHCl, 4 mM EDTA, pH 7.4, 0.1% BSA). cAMP content was determined by a competitive radiobinding assay for PKA using [^3^H]-cAMP as previously described in Saraullo [28]. Briefly, tritiated PKA (100 μL) was incubated in equilibrium conditions (2 h, 4 °C) with 50 μL of different samples of cAMP standards (0.1 to 90 pmol) in the presence of 50 μL 8 nM [^3^H]-cAMP (PerkinElmer, NET1161250UC, Waltham, MA, USA) in an RBP buffer. The bound fraction was separated by carbon-dextran precipitation, followed by centrifugation (1000× *g*, 15 min, 4 °C) and supernatants added to Optiphase HiSafe scintilliation cocktail (Perkin Elmer, cat 1200.437) and quantified in a Hidex/300 SL liquid scintilliation counter (Hidex, Turku, Finland). Sample cAMP concentrations were determined by interpolating the displacement curves obtained from cAMP standards. Duplicate samples in at least three independent experiments were analyzed. The presence of the studied inhibitors, up to the maximum concentration studied, showed no interference with cAMP as observed in the radiobinding protein assay (see Figure S1, Supplementary Materials).

### 3.7. Determination of the Half-Maximal Inhibitory Concentration (IC_50_)

To determine the IC_50_ value, a concentration–response experiment was performed where the adenylyl cyclase activity of the gNC1 enzyme was measured in the presence of 100 μM ATP and increasing concentrations of the target samples, from 0.1 to 250 µg/mL in the case of the plant extracts and from 0.1 to 100 µM for compound **3**. The control group contained only DMSO. In all cases, the best fit curve of each experiment was obtained with a script of Python developed by us using the matplotlib version 3.5 (https://matplotlib.org/) and numpy version 1.23.0 (https://numpy.org/) libraries. This script fit the sum of the least squares of differences method to the data according to the following equation:

y = Min + (Max − Min)/(1 + (x/IC_50_)^n^)

where ‘y’ is the enzymatic activity calculated; ‘Min’ is the minimal enzymatic activity; ‘Max’ is the maximal enzymatic activity; ‘x’ is the inhibitor concentration; ‘IC_50_’ is the half-maximal inhibitory concentration; and ‘n’ is the Hill coefficient.

### 3.8. Kinetic Behavior of Rosmarinic Acid (***3***)

In order to determine the kinetic behavior of compound **3** on gNC1, increasing concentrations of ATP (from 1.8 to 150 µM) were added in the presence of 5 µL of compound **3** dissolved in DMSO to reach final concentrations of 5 and 10 µM. The control group contained only DMSO. In all cases, the best fit curve of each experiment was obtained with a script of Python developed by us using the matplotlib (https://matplotlib.org/) and numpy (https://numpy.org/) libraries. This script fit the sum of the least squares of differences method to the data according to the following equation:

v = V_max_[S]^n^/(K_0.5_^n^ + [S]^n^)

where ‘v’ is the enzymatic activity; ‘V*_max_*’ is the maximal enzymatic activity; ‘[S]’ is the substrate concentration; ‘K_0.5_’ is the substrate concentration for half-saturation; and ‘n’ is the Hill coefficient.

### 3.9. cAMP-Dependent Protein Kinase (PKA) Purification

PKA purification was adapted from the protocol previously described by Saraullo [28]. In all cAMP protein radiobinding experiments, 100 μL of PKA dissolved in RBP buffer (50 mM Tris-HCl, 4 mM EDTA, pH 7.4, 0.1% BSA) was used and was able to bind between 35 and 50% of 2 nM cAMP.

### 3.10. Protein Structure and Energy Minimization

In the present study, we used the three-dimensional structure of the enzyme gNC1 from our previous work [36]. The Ramachandran plot (Figure S2) and atomic coordinates are available in the Supplementary Materials.

The energy minimization process was carried out with the AMBER 16 molecular dynamics package [52]. The purpose of energy minimization is to relax the system and remove any steric clashes or unfavorable interactions between atoms. The steepest descent method was initially employed for 500 steps, followed by a switch to the conjugate gradient method. This combination of optimization algorithms allows for an efficient and effective energy minimization. The protein backbone atoms were subjected to positional restraint by applying a soft force constant of 0.5 kcal/mol/Å^2^.

### 3.11. Docking

Docking simulations were carried out using Autodock Vina version 1.2 [53,54]. In docking experiments, the simulation box was centered on the catalytic pocket of gNC1, which includes both active sites. The exhaustiveness parameter, which controls the thoroughness of the docking search, was raised from a value of 8 (the default) to 32. By setting the exhaustiveness to 32, Autodock Vina was used to perform a relatively comprehensive exploration of the conformational space during the docking process. It generated a sufficient number of docking poses to adequately sample the ligand’s potential binding modes within the receptor’s binding site.

### 3.12. Molecular Dynamics

All molecular dynamics simulations and subsequent analysis were performed with AMBER 16 software package [52]. The force field parameters employed for the ATP molecule and divalent cations Ca^2+^ and Mn^2+^ were those reported by Meagher [55] and Bradbrook [56], respectively. The gNC1 protein was parameterized within an amber14SB force field, a force field widely used for proteins, while the parameters for all other compounds were derived from the GAFF (General Amber Force Field), which is commonly used for small organic molecules. Each molecular model was solvated in a truncated octahedral periodic box using the TIP3P water model. The minimum distance between the edges of the water box and the closest solute atom was maintained at 10 Å, ensuring sufficient space for molecular dynamics simulations. To neutralize the overall system charge, chloride ions were added accordingly. Prior to the production runs, the entire system underwent an energy minimization step, keeping the protein backbone fixed with harmonic constraint using a soft force of 1 kcal/mol Å^2^. Subsequently, the minimized systems were equilibrated under the NVT ensemble at 305 K for 500 ps. Constant temperature was achieved using a Langevin thermostat [57] with a collision frequency of 1.0 ps^−1^. After the equilibration phase, production runs were conducted under the NVT ensemble for a duration of 50 ns. Throughout each production run, structural coordinates, velocities, and energies were recorded at regular intervals, allowing for detailed analysis of the molecular dynamics trajectories.

### 3.13. Structural Mobility

The structural mobility analysis was conducted using the MDLovoFit program version 16.264 [58], which utilizes the Low-Order-Value-Optimization (LOVO) strategy algorithm [59,60]. This algorithm offers an automated and reliable approach to identifying and quantifying mobile and rigid substructures within a molecular dynamics simulation.

### 3.14. Binding Free Energies and Residue Decomposition Analysis

The binding free energy was calculated using the molecular mechanics Poisson–Boltzmann surface area (MM-PBSA) approach, which combines molecular mechanics and continuum solvent models to estimate binding affinities. The MM-PBSA method utilizes the following equation:

ΔG_binding_ = G_PL_ − G_P_ − G_L_

where G_PL_, G_P_, and G_L_ represent the free energy of the protein–ligand complex, protein, and ligand, respectively. These values are estimated using the following expression:

G = E_bond_ + E_elec_ + E_vander_ + G_polar_ + G_non-polar_ − TS

The terms E_bond_, E_elec_, and E_vander_ correspond to the classical molecular mechanics energy contributions from bonded interactions, electrostatic interactions, and van der Waals interactions, respectively. The terms G_polar_ and G_non-polar_ represent the polar and non-polar solvation energy contributions. The last term, TS, accounts for the temperature and entropy effects of the complex upon ligand binding. The MMPBSA.py script was utilized for performing the MM-PBSA calculations. This tool calculates the binding energy components and provides individual energy contributions of amino acids. For the calculation of polar solvation energy contributions, the Poisson–Boltzmann solver method was employed as implemented in the SANDER program [52]. The non-polar contributions of solvation energy were determined using the solvent-accessible surface area (SASA) approach. Due to the high computational cost and large standard error related to the entropic term in the MM-PBSA calculations, it was not included in the models presented in this work. By excluding the entropic term, the computational demand was reduced, and the estimation of the binding free energy was still informative for the investigated systems.

Following the completion of the MM-PBSA calculations, the energy contributions of individual amino acids were analyzed to identify crucial residues involved in the binding.

## 4. Conclusions

The results obtained allowed us to draw interesting conclusions from different aspects. On one hand, the strong inhibitory activity of *L. floribunda* and *L. meyenii* extracts on gNC1 from *G. lamblia* opens up the possibility of using these vegetal products as a source of candidates for treating giardiasis. This activity might be attributed, at least in part, to the presence of rosmarinic acid (**3**). From a pharmacological point of view, compound **3** has been described as a noticeable antioxidant agent [46], an effect attributed to the presence of the catechol moiety [51,61], which also plays a key role in its interaction with gNC1. In addition, compound **3** shows anticancer, anti-inflammatory, antiviral, antibacterial, neuroprotective and hepatoprotective properties, among other benefits [43,46,62].

On the other hand, attractive results were obtained from the medicinal chemistry point of view, since both theoretical and experimental results showed compound **3** to be a promising starting structure for the search of novel inhibitors of gNC1 from *G. lamblia*. In addition, molecular simulations shed light the mechanism of action of this inhibitor at the molecular level, determining the main molecular interactions that stabilize the complex of this ligand at the binding site of the enzyme.

Finally, it is also possible to obtain interesting conclusions from the methodological point of view, since the results presented demonstrated once again that both the in silico and experimental models are very useful tools for identifying and designing new inhibitors of gNC1 from *G. lamblia.* These are very interesting findings, particularly considering the importance of finding new therapeutic agents to counteract this parasite.

## Figures and Tables

**Table 1:** Plants from central Argentina screened for the inhibition of adenylyl cyclase gNC1 from *Giardia lamblia*.

Plant Species	Family	Status ^a^	Common Name	Yield (%) ^b^	Voucher UCCOR Number
*Achyrocline satureioides* (Lam.) DC.	Asteraceae	N	marcela hembra	4.5	140
*Baccharis coridifolia* DC.	Asteraceae	N	mio-mio	2.2	147
*Calceolaria parviflora* Benth.	Calceolariaceae	E	zapatito	6.1	451
*Dimerostemma aspilioides* (Griseb.) M.D. Moraes	Asteraceae	E	-	5.5	246
*Flourensia oolepis* S.F. Blake	Asteraceae	E	chilca	23.0	135
*Gaillardia megapotamica* (Spreng.) Baker	Asteraceae	E	topasaire	13.76	127
*Lepechinia floribunda* (Benth.) *Epling*	Lamiaceae	N	salvia blanca	3.7	195
*Lepechinia meyenii* (Walp.) Epling	Lamiaceae	N	-	3.8	233
*Ligaria cuneifolia* (Ruiz & Pav.) Tiegh. (parasitizing *Vachellia* sp.)	Loranthaceae	N	liga	4.4	518
*Lithrea molleoides* (Vell.) Engl.	Anacardiaceae	N	molle	10.9	183
*Melia azedarach* L	Meliaceae	Nat	paraíso	8.7	229 ^c^
*Prosopis alba* Griseb.	Fabaceae	N	algarrobo blanco	8.5	255
*Senecio viravira* Hieron	Asteraceae	N	viravira	3.9	181
*Solanum atriplicifolium* Gillies ex Nees	Solanaceae	N	-	18.08	528

^a^ E: endemic; Nat: naturalized; N: native. ^b^ Yield on a wet basis. ^c^ Cord number.

**Table 2:** Inhibitory effect of the target extracts on the adenylyl cyclase gNC1-301 activity.

Plant Extract	IC_50_ (µg/mL) ^a^
*Achyrocline satureioides*	51.7 ± 2.5
*Baccharis coridifolia*	35.1 ± 6.4
*Lepechinia floribunda*	9.4 ± 0.8
*Lepechinia meyenii*	31.3 ± 3.2
*Lithrea molleoides*	67.0 ± 4.8

^a^ Results are expressed as the mean ± standard error.

**Table 3:** Hydrogen bond analysis of compound **3**/gNC1’s interactions.

Hydrogen Bond (Acceptor ||| Donor)	Fraction	Average Distance (Å)
Compound **3**_(site 1)_@O_1_ ||| THR56@OG_1_HG_1_	0.6746	2.7662
ASP 16@OD_2_ ||| compound **3**_(site 1)_@O_4_H_13_	0.6384	2.6005
ASP 16@OD_2_ ||| compound **3**_(site 1)_@O_3_H_12_	0.6326	2.5826
ASP 244@OD_1_ ||| compound **3**_(site 1)_@O_2_H_11_	0.5018	2.6209
ALA 126@O ||| compound **3**_(site 1)_@O_1_H_10_	0.3572	2.7751
compound **3**_(site 1)_@O_6_ ||| ASN 132@ND_2_HD_21_	0.104	2.8439
ASP 244@OD_2_ ||| compound **3**_(site 1)_@O_2_H_11_	0.0934	2.6451
SER 312@O ||| compound **3**_(site 2)_@O_2_H_11_	0.6446	2.8028
Compound **3**_(site 2)_ @O_3_ ||| SER 319@OGHG	0.2506	2.7949
Compound **3**_(site 2)_@O_7_ ||| THR 21@OG_1_HG_1_	0.1232	2.7127
Compound **3**_(site 2)_@O_1_ ||| SER 312@NH	0.1078	2.9284
SER 319@OG ||| compound **3**_(site 2)_@O_3_H_12_	0.0904	2.8027

**Table 4:** Inhibitory effect of rosmarinic acid (**3**) on gNC1 adenylyl cyclase activity.

Rosmarinic Acid (3)	K_0.5_	V*max* (pmol/min/mg Total Protein)	Hill Coefficient
0 (DMSO control)	17.0 ± 1.9	1249 ± 48	1.64 ± 0.32
5 µM	47.0 ± 8.9	1300 ± 100	1.10 ± 0.08
10 µM	150.0 ± 16.9	1283 ± 93	1.04 ± 0.04

## Data Availability

The data are available on request from the correspondent authors A.Z., M.C.C. or R.D.E.

## Supplementary Materials

The following supporting information can be downloaded at: https://www.mdpi.com/article/10.3390/plants13050646/s1, Figure S1. The controls for the Radiobinding-Protein (RBP) assay were conducted under the specified conditions; Figure S2. PROCHECK’s Ramachandran plot that validates the predicted 3D model of gNC1. Reference [63] is cited in Supplementary Materials.

## Abbreviations

2-CE: 2-catechol estrogen; AC, adenylyl cyclase; ATP, adenosine triphosphate; BSA, bovine serum albumin; cAMP, cyclic adenosine monophosphate; DMSO, dimethyl sulfoxide; gNC1, Giardia nucleotidyl cyclase 1; gNC2, Giardia nucleotidyl cyclase 2; gPDE, Giardia phosphodiesterase; MD, molecular dynamic; PDE, phosphodiesterase; PKA, protein kinase A; RBP, radiobinding protein; RMSD, root mean square deviation; RMSF, root mean square fluctuation.
