# Supplementary material for: Rosmarinic Acid Present in Lepechinia floribunda and Lepechinia meyenii as a Potent Inhibitor of the Adenylyl Cyclase gNC1 from Giardia lamblia
Source: Plants (Basel). 2024 Feb 26;13(5):646. doi: 10.3390/plants13050646 (PMC10935199; doi:10.3390/plants13050646)
Supplement: Supplementary file 1 [file plants-13-00646-s001.zip › plants-2872480-supplementary.pdf]

Supplementary Materials

Rosmarinic Acid Present in *Lepechinia floribunda* and *Lepechinia meyenii* As A Potent Inhibitor of the Adenylyl Cyclase gNC1 from *Giardia lamblia*

Adolfo Zurita [1,†,*], Esteban Vega Hissi [1], Agostina Cianci Romero [1], Adela María Luján [2], Sofía Salido [3], Agustín Yaneff [4], Carlos Davio [4], Justo Cobo [3], María Cecilia Carpinella [2,†,*] and Ricardo Daniel Enriz [1,†,*]

[1] Facultad de Química, Bioquímica y Farmacia, Universidad Nacional de San Luis, Instituto Multidisciplinario de Investigaciones Biológicas (IMIBIO-SL), Ejército de los Andes 950, San Luis 5700, Argentina; egvega@gmail.com (E.V.H.); agostinacianci.acr@gmail.com (A.C.R.)

[2] Laboratorio de Química Fina y Productos Naturales, Centro de Investigación y Desarrollo en Inmunología y Enfermedades Infecciosas (CIDIE) CONICET-UCC, Universidad Católica de Córdoba, Avda. Armada Argentina 3555, Córdoba X5016DHK, Argentina; adem.lujan@gmail.com

[3] Departamento de Química Inorgánica y Orgánica, Universidad de Jaén, Campus Las Lagunillas s/n, 23071 Jaén, Spain; ssalido@ujaen.es (S.S.); jcobo@ujaen.es (J.C.)

[4] Instituto de Investigaciones Farmacológicas (ININFA-UBA-CONICET), Facultad de Farmacia y Bioquímica, Universidad de Buenos Aires, Junín 956, Buenos Aires C1113AAD, Argentina; agustinyaneff@hotmail.com (A.Y.); carlosdavio@hotmail.com (C.D.)

* Correspondence: azurita1974@gmail.com (A.Z.); ceciliacarpinella@ucc.edu.ar (M.C.C.); danielenriz@gmail.com (R.D.E.)

† These authors contributed equally to this work.

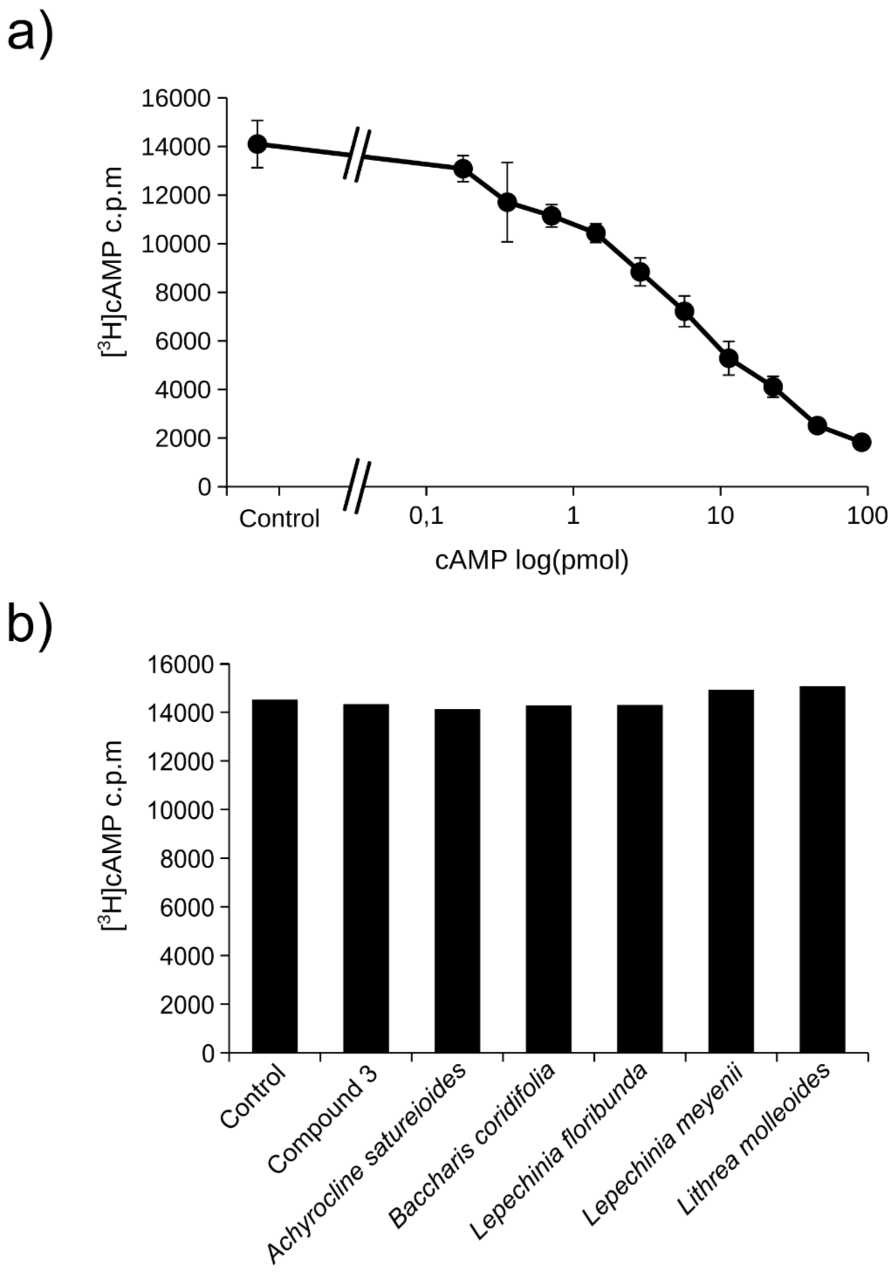

Figure S1. The controls for the Radiobinding-Protein (RBP) assay were conducted under the specified conditions: (a) in the presence of increasing concentrations of cAMP, and (b) in the presence of DMSO (negative control), 50 µM of rosmarinic acid (**3**) or 250 µg/mL of the target plant extracts. The results obtained showed that these inhibitors did not interfere with the binding between cAMP and the protein kinase A used in the RBP buffer.

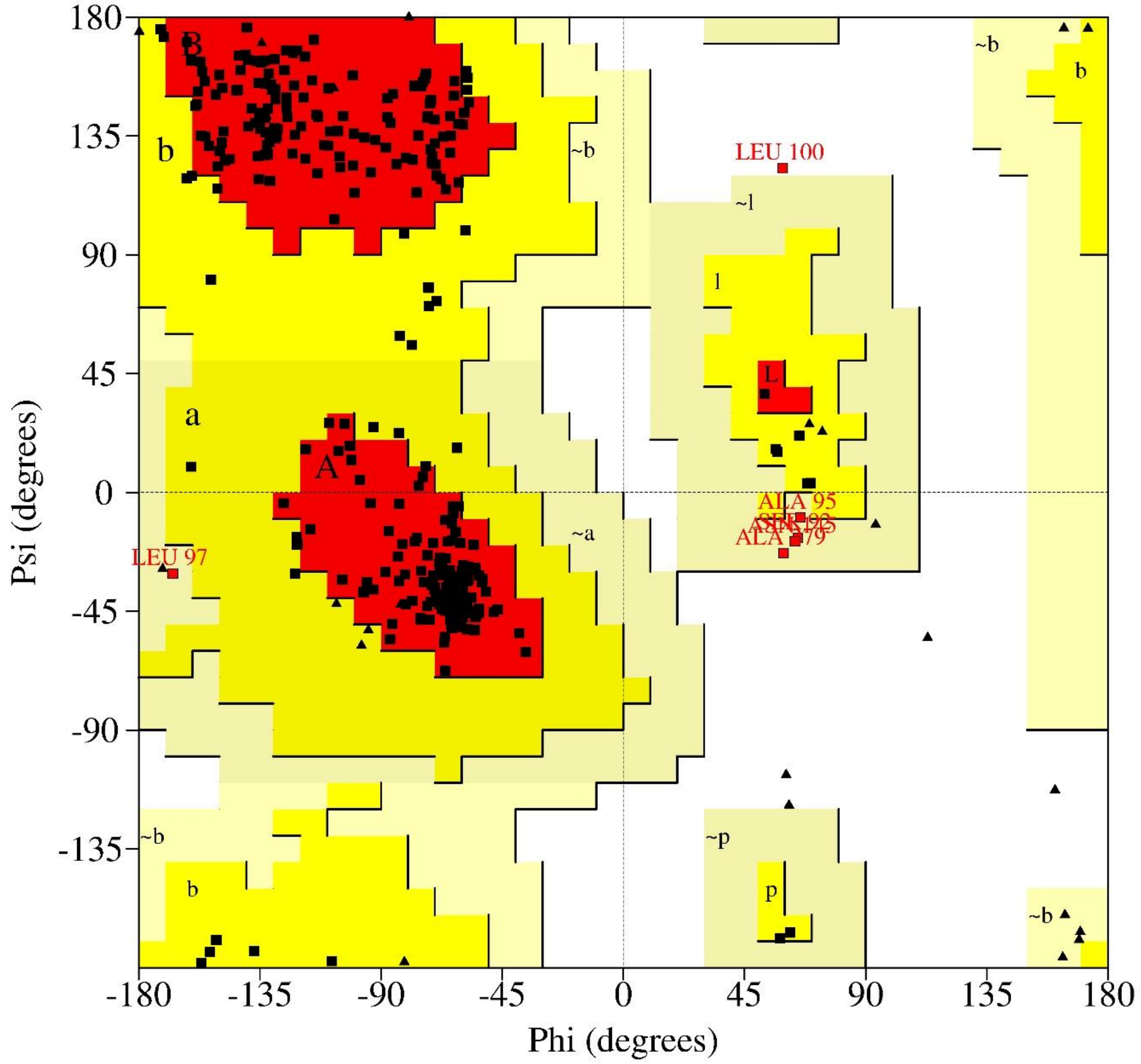

Figure S2. PROCHECK's[1] Ramachandran plot that validates the predicted 3D model of gNC1. A good quality model has been obtained since most residues (87.7%) are situated in the most favored regions and 10.5% are in additional allowed regions. A 1.5% of residues are found in generously allowed regions, while only one residue (0.3%) is located in disallowed regions; these latter correspond to residues located in external regions associated to high protein flexibility.

1. Laskowski, R.A.; MacArthur, M.W.; Moss, D.S.; Thornton, J.M. PROCHECK: a program to check the stereochemical quality of protein structures. J. Appl. Cryst. 1993, 26, 283-291, doi:10.1107/S0021889892009944

Molecular structure of gNC1 from *Giardia lamblia*

ATOM	1	N	LEU	1	25.530	34.611	37.113	1.00	0.00	N
ATOM	2	H1	LEU	1	26.040	33.997	37.726	1.00	0.00	H
ATOM	3	H2	LEU	1	25.444	35.527	37.538	1.00	0.00	H
ATOM	4	H3	LEU	1	24.587	34.254	37.025	1.00	0.00	H
ATOM	5	CA	LEU	1	26.169	34.712	35.782	1.00	0.00	C
ATOM	6	HA	LEU	1	27.171	35.117	35.922	1.00	0.00	H
ATOM	7	CB	LEU	1	26.317	33.345	35.083	1.00	0.00	C
ATOM	8	HB2	LEU	1	25.328	32.941	34.870	1.00	0.00	H
ATOM	9	HB3	LEU	1	26.820	33.501	34.127	1.00	0.00	H
ATOM	10	CG	LEU	1	27.114	32.302	35.893	1.00	0.00	C
ATOM	11	HG	LEU	1	26.618	32.134	36.849	1.00	0.00	H
ATOM	12	CD1	LEU	1	27.171	30.973	35.142	1.00	0.00	C
ATOM	13	HD11	LEU	1	26.160	30.605	34.964	1.00	0.00	H
ATOM	14	HD12	LEU	1	27.711	30.235	35.735	1.00	0.00	H
ATOM	15	HD13	LEU	1	27.683	31.099	34.186	1.00	0.00	H
ATOM	16	CD2	LEU	1	28.545	32.771	36.163	1.00	0.00	C
ATOM	17	HD21	LEU	1	29.050	32.976	35.218	1.00	0.00	H
ATOM	18	HD22	LEU	1	28.557	33.665	36.784	1.00	0.00	H
ATOM	19	HD23	LEU	1	29.093	31.985	36.686	1.00	0.00	H
ATOM	20	C	LEU	1	25.452	35.716	34.880	1.00	0.00	C
ATOM	21	O	LEU	1	26.129	36.573	34.325	1.00	0.00	O
ATOM	22	N	LYS	2	24.124	35.626	34.712	1.00	0.00	N
ATOM	23	H	LYS	2	23.619	34.904	35.223	1.00	0.00	H
ATOM	24	CA	LYS	2	23.337	36.396	33.724	1.00	0.00	C
ATOM	25	HA	LYS	2	23.770	36.225	32.737	1.00	0.00	H
ATOM	26	CB	LYS	2	21.894	35.854	33.718	1.00	0.00	C
ATOM	27	HB2	LYS	2	21.521	35.779	34.742	1.00	0.00	H
ATOM	28	HB3	LYS	2	21.252	36.549	33.174	1.00	0.00	H
ATOM	29	CG	LYS	2	21.805	34.485	33.023	1.00	0.00	C
ATOM	30	HG2	LYS	2	22.166	34.596	31.999	1.00	0.00	H
ATOM	31	HG3	LYS	2	22.435	33.758	33.535	1.00	0.00	H
ATOM	32	CD	LYS	2	20.361	33.970	32.990	1.00	0.00	C
ATOM	33	HD2	LYS	2	20.034	33.709	33.999	1.00	0.00	H
ATOM	34	HD3	LYS	2	19.721	34.762	32.602	1.00	0.00	H
ATOM	35	CE	LYS	2	20.277	32.743	32.073	1.00	0.00	C
ATOM	36	HE2	LYS	2	20.880	32.950	31.185	1.00	0.00	H
ATOM	37	HE3	LYS	2	20.706	31.876	32.581	1.00	0.00	H
ATOM	38	NZ	LYS	2	18.888	32.467	31.637	1.00	0.00	N
ATOM	39	HZ1	LYS	2	18.885	31.883	30.804	1.00	0.00	H
ATOM	40	HZ2	LYS	2	18.420	33.333	31.374	1.00	0.00	H
ATOM	41	HZ3	LYS	2	18.318	32.005	32.339	1.00	0.00	H
ATOM	42	C	LYS	2	23.336	37.930	33.874	1.00	0.00	C
ATOM	43	O	LYS	2	22.890	38.607	32.947	1.00	0.00	O
ATOM	44	N	SER	3	23.827	38.506	34.976	1.00	0.00	N
ATOM	45	H	SER	3	24.094	37.910	35.751	1.00	0.00	H
ATOM	46	CA	SER	3	24.011	39.961	35.120	1.00	0.00	C
ATOM	47	HA	SER	3	23.088	40.451	34.809	1.00	0.00	H
ATOM	48	CB	SER	3	24.235	40.334	36.594	1.00	0.00	C
ATOM	49	HB2	SER	3	23.353	40.066	37.178	1.00	0.00	H
ATOM	50	HB3	SER	3	25.093	39.782	36.979	1.00	0.00	H

ATOM	51	OG	SER	3	24.478	41.731	36.714	1.00	0.00	O
ATOM	52	HG	SER	3	25.134	41.946	36.031	1.00	0.00	H
ATOM	53	C	SER	3	25.142	40.509	34.225	1.00	0.00	C
ATOM	54	O	SER	3	26.228	40.846	34.709	1.00	0.00	O
ATOM	55	N	HID	4	24.861	40.675	32.929	1.00	0.00	N
ATOM	56	H	HID	4	23.982	40.302	32.593	1.00	0.00	H
ATOM	57	CA	HID	4	25.693	41.453	32.002	1.00	0.00	C
ATOM	58	HA	HID	4	26.667	40.966	31.938	1.00	0.00	H
ATOM	59	CB	HID	4	25.065	41.433	30.599	1.00	0.00	C
ATOM	60	HB2	HID	4	24.027	41.765	30.662	1.00	0.00	H
ATOM	61	HB3	HID	4	25.600	42.138	29.962	1.00	0.00	H
ATOM	62	CG	HID	4	25.119	40.075	29.935	1.00	0.00	C
ATOM	63	ND1	HID	4	26.205	39.532	29.281	1.00	0.00	N
ATOM	64	HD1	HID	4	27.109	39.981	29.117	1.00	0.00	H
ATOM	65	CE1	HID	4	25.882	38.287	28.895	1.00	0.00	C
ATOM	66	HE1	HID	4	26.543	37.614	28.359	1.00	0.00	H
ATOM	67	NE2	HID	4	24.626	37.988	29.269	1.00	0.00	N
ATOM	68	CD2	HID	4	24.139	39.117	29.941	1.00	0.00	C
ATOM	69	HD2	HID	4	23.166	39.210	30.404	1.00	0.00	H
ATOM	70	C	HID	4	25.931	42.890	32.517	1.00	0.00	C
ATOM	71	O	HID	4	25.148	43.427	33.302	1.00	0.00	O
ATOM	72	N	ALA	5	27.056	43.492	32.123	1.00	0.00	N
ATOM	73	H	ALA	5	27.641	43.028	31.447	1.00	0.00	H
ATOM	74	CA	ALA	5	27.580	44.695	32.770	1.00	0.00	C
ATOM	75	HA	ALA	5	27.572	44.506	33.844	1.00	0.00	H
ATOM	76	CB	ALA	5	29.037	44.880	32.351	1.00	0.00	C
ATOM	77	HB1	ALA	5	29.097	45.072	31.279	1.00	0.00	H
ATOM	78	HB2	ALA	5	29.587	43.972	32.594	1.00	0.00	H
ATOM	79	HB3	ALA	5	29.478	45.717	32.894	1.00	0.00	H
ATOM	80	C	ALA	5	26.750	45.973	32.538	1.00	0.00	C
ATOM	81	O	ALA	5	26.171	46.192	31.475	1.00	0.00	O
ATOM	82	N	THR	6	26.756	46.844	33.547	1.00	0.00	N
ATOM	83	H	THR	6	27.335	46.615	34.348	1.00	0.00	H
ATOM	84	CA	THR	6	25.916	48.049	33.663	1.00	0.00	C
ATOM	85	HA	THR	6	25.384	48.209	32.725	1.00	0.00	H
ATOM	86	CB	THR	6	24.868	47.872	34.780	1.00	0.00	C
ATOM	87	HB	THR	6	24.264	48.779	34.830	1.00	0.00	H
ATOM	88	CG2	THR	6	23.928	46.692	34.539	1.00	0.00	C
ATOM	89	HG21	THR	6	23.148	46.689	35.299	1.00	0.00	H
ATOM	90	HG22	THR	6	24.473	45.749	34.585	1.00	0.00	H
ATOM	91	HG23	THR	6	23.464	46.786	33.557	1.00	0.00	H
ATOM	92	OG1	THR	6	25.481	47.676	36.039	1.00	0.00	O
ATOM	93	HG1	THR	6	26.081	46.923	35.956	1.00	0.00	H
ATOM	94	C	THR	6	26.757	49.295	33.946	1.00	0.00	C
ATOM	95	O	THR	6	27.871	49.182	34.452	1.00	0.00	O
ATOM	96	N	VAL	7	26.237	50.486	33.630	1.00	0.00	N
ATOM	97	H	VAL	7	25.311	50.512	33.231	1.00	0.00	H
ATOM	98	CA	VAL	7	26.774	51.768	34.124	1.00	0.00	C
ATOM	99	HA	VAL	7	27.840	51.652	34.320	1.00	0.00	H
ATOM	100	CB	VAL	7	26.632	52.907	33.090	1.00	0.00	C
ATOM	101	HB	VAL	7	27.231	52.640	32.229	1.00	0.00	H
ATOM	102	CG1	VAL	7	25.201	53.133	32.583	1.00	0.00	C

```
ATOM    103 HG11 VAL     7      24.805  52.224  32.133  1.00  0.00           H
ATOM    104 HG12 VAL     7      24.552  53.440  33.400  1.00  0.00           H
ATOM    105 HG13 VAL     7      25.202  53.917  31.824  1.00  0.00           H
ATOM    106  CG2 VAL     7      27.184  54.237  33.614  1.00  0.00           C
ATOM    107 HG21 VAL     7      28.201  54.101  33.988  1.00  0.00           H
ATOM    108 HG22 VAL     7      26.559  54.634  34.414  1.00  0.00           H
ATOM    109 HG23 VAL     7      27.218  54.967  32.805  1.00  0.00           H
ATOM    110  C   VAL     7      26.095  52.124  35.444  1.00  0.00           C
ATOM    111  O   VAL     7      24.871  52.001  35.572  1.00  0.00           O
ATOM    112  N   ARG     8      26.868  52.597  36.425  1.00  0.00           N
ATOM    113  H   ARG     8      27.875  52.661  36.272  1.00  0.00           H
ATOM    114  CA  ARG     8      26.323  53.095  37.691  1.00  0.00           C
ATOM    115  HA  ARG     8      25.333  53.507  37.489  1.00  0.00           H
ATOM    116  CB  ARG     8      26.184  51.932  38.694  1.00  0.00           C
ATOM    117  HB2 ARG     8      25.955  51.013  38.152  1.00  0.00           H
ATOM    118  HB3 ARG     8      27.130  51.791  39.222  1.00  0.00           H
ATOM    119  CG  ARG     8      25.053  52.192  39.703  1.00  0.00           C
ATOM    120  HG2 ARG     8      25.188  53.169  40.161  1.00  0.00           H
ATOM    121  HG3 ARG     8      24.098  52.195  39.174  1.00  0.00           H
ATOM    122  CD  ARG     8      24.995  51.155  40.829  1.00  0.00           C
ATOM    123  HD2 ARG     8      25.908  51.222  41.425  1.00  0.00           H
ATOM    124  HD3 ARG     8      24.151  51.415  41.471  1.00  0.00           H
ATOM    125  NE  ARG     8      24.825  49.774  40.332  1.00  0.00           N
ATOM    126  HE  ARG     8      24.903  49.629  39.340  1.00  0.00           H
ATOM    127  CZ  ARG     8      24.571  48.718  41.085  1.00  0.00           C
ATOM    128  NH1 ARG     8      24.433  48.796  42.374  1.00  0.00           N
ATOM    129 HH11 ARG     8      24.452  49.715  42.835  1.00  0.00           H
ATOM    130 HH12 ARG     8      24.187  48.002  42.947  1.00  0.00           H
ATOM    131  NH2 ARG     8      24.450  47.538  40.544  1.00  0.00           N
ATOM    132 HH21 ARG     8      24.547  47.422  39.550  1.00  0.00           H
ATOM    133 HH22 ARG     8      24.225  46.756  41.132  1.00  0.00           H
ATOM    134  C   ARG     8      27.180  54.217  38.261  1.00  0.00           C
ATOM    135  O   ARG     8      28.404  54.162  38.213  1.00  0.00           O
ATOM    136  N   PHE     9      26.527  55.197  38.876  1.00  0.00           N
ATOM    137  H   PHE     9      25.521  55.203  38.850  1.00  0.00           H
ATOM    138  CA  PHE     9      27.185  56.084  39.825  1.00  0.00           C
ATOM    139  HA  PHE     9      28.192  56.294  39.471  1.00  0.00           H
ATOM    140  CB  PHE     9      26.428  57.420  39.891  1.00  0.00           C
ATOM    141  HB2 PHE     9      25.453  57.248  40.350  1.00  0.00           H
ATOM    142  HB3 PHE     9      26.986  58.102  40.531  1.00  0.00           H
ATOM    143  CG  PHE     9      26.224  58.108  38.551  1.00  0.00           C
ATOM    144  CD1 PHE     9      24.923  58.362  38.072  1.00  0.00           C
ATOM    145  HD1 PHE     9      24.059  58.068  38.651  1.00  0.00           H
ATOM    146  CE1 PHE     9      24.739  59.009  36.837  1.00  0.00           C
ATOM    147  HE1 PHE     9      23.740  59.209  36.476  1.00  0.00           H
ATOM    148  CZ  PHE     9      25.853  59.401  36.073  1.00  0.00           C
ATOM    149  HZ  PHE     9      25.709  59.904  35.125  1.00  0.00           H
ATOM    150  CE2 PHE     9      27.152  59.146  36.545  1.00  0.00           C
ATOM    151  HE2 PHE     9      28.010  59.444  35.957  1.00  0.00           H
ATOM    152  CD2 PHE     9      27.337  58.506  37.784  1.00  0.00           C
ATOM    153  HD2 PHE     9      28.338  58.321  38.143  1.00  0.00           H
ATOM    154  C   PHE     9      27.278  55.378  41.191  1.00  0.00           C
```

ATOM	155	O	PHE	9	26.255	55.011	41.775	1.00	0.00	O
ATOM	156	N	ALA	10	28.491	55.199	41.709	1.00	0.00	N
ATOM	157	H	ALA	10	29.289	55.476	41.147	1.00	0.00	H
ATOM	158	CA	ALA	10	28.784	54.575	43.001	1.00	0.00	C
ATOM	159	HA	ALA	10	27.856	54.252	43.472	1.00	0.00	H
ATOM	160	CB	ALA	10	29.627	53.321	42.733	1.00	0.00	C
ATOM	161	HB1	ALA	10	30.562	53.601	42.245	1.00	0.00	H
ATOM	162	HB2	ALA	10	29.077	52.638	42.084	1.00	0.00	H
ATOM	163	HB3	ALA	10	29.849	52.812	43.672	1.00	0.00	H
ATOM	164	C	ALA	10	29.485	55.543	43.975	1.00	0.00	C
ATOM	165	O	ALA	10	30.043	56.560	43.568	1.00	0.00	O
ATOM	166	N	THR	11	29.500	55.201	45.264	1.00	0.00	N
ATOM	167	H	THR	11	29.025	54.339	45.515	1.00	0.00	H
ATOM	168	CA	THR	11	30.471	55.732	46.238	1.00	0.00	C
ATOM	169	HA	THR	11	31.015	56.568	45.798	1.00	0.00	H
ATOM	170	CB	THR	11	29.809	56.221	47.538	1.00	0.00	C
ATOM	171	HB	THR	11	29.556	55.366	48.162	1.00	0.00	H
ATOM	172	CG2	THR	11	30.763	57.130	48.312	1.00	0.00	C
ATOM	173	HG21	THR	11	31.642	56.571	48.631	1.00	0.00	H
ATOM	174	HG22	THR	11	30.264	57.517	49.196	1.00	0.00	H
ATOM	175	HG23	THR	11	31.073	57.969	47.687	1.00	0.00	H
ATOM	176	OG1	THR	11	28.633	56.963	47.304	1.00	0.00	O
ATOM	177	HG1	THR	11	27.933	56.349	47.042	1.00	0.00	H
ATOM	178	C	THR	11	31.467	54.617	46.559	1.00	0.00	C
ATOM	179	O	THR	11	31.049	53.475	46.742	1.00	0.00	O
ATOM	180	N	ILE	12	32.765	54.913	46.619	1.00	0.00	N
ATOM	181	H	ILE	12	33.044	55.879	46.480	1.00	0.00	H
ATOM	182	CA	ILE	12	33.839	53.909	46.625	1.00	0.00	C
ATOM	183	HA	ILE	12	33.407	52.908	46.653	1.00	0.00	H
ATOM	184	CB	ILE	12	34.651	54.040	45.319	1.00	0.00	C
ATOM	185	HB	ILE	12	35.039	55.060	45.273	1.00	0.00	H
ATOM	186	CG2	ILE	12	35.870	53.109	45.319	1.00	0.00	C
ATOM	187	HG21	ILE	12	36.561	53.371	46.120	1.00	0.00	H
ATOM	188	HG22	ILE	12	36.398	53.240	44.382	1.00	0.00	H
ATOM	189	HG23	ILE	12	35.569	52.068	45.441	1.00	0.00	H
ATOM	190	CG1	ILE	12	33.808	53.823	44.038	1.00	0.00	C
ATOM	191	HG12	ILE	12	33.038	54.593	43.978	1.00	0.00	H
ATOM	192	HG13	ILE	12	34.456	53.955	43.173	1.00	0.00	H
ATOM	193	CD1	ILE	12	33.133	52.454	43.897	1.00	0.00	C
ATOM	194	HD11	ILE	12	33.877	51.660	43.923	1.00	0.00	H
ATOM	195	HD12	ILE	12	32.614	52.408	42.939	1.00	0.00	H
ATOM	196	HD13	ILE	12	32.406	52.308	44.694	1.00	0.00	H
ATOM	197	C	ILE	12	34.741	54.049	47.856	1.00	0.00	C
ATOM	198	O	ILE	12	35.262	55.133	48.117	1.00	0.00	O
ATOM	199	N	MET	13	34.965	52.937	48.564	1.00	0.00	N
ATOM	200	H	MET	13	34.505	52.091	48.241	1.00	0.00	H
ATOM	201	CA	MET	13	35.983	52.778	49.615	1.00	0.00	C
ATOM	202	HA	MET	13	36.122	53.722	50.145	1.00	0.00	H
ATOM	203	CB	MET	13	35.503	51.703	50.615	1.00	0.00	C
ATOM	204	HB2	MET	13	34.677	52.101	51.200	1.00	0.00	H
ATOM	205	HB3	MET	13	35.126	50.874	50.022	1.00	0.00	H
ATOM	206	CG	MET	13	36.544	51.102	51.583	1.00	0.00	C

ATOM	207	HG2	MET	13	37.478	50.927	51.057	1.00	0.00	H
ATOM	208	HG3	MET	13	36.744	51.802	52.393	1.00	0.00	H
ATOM	209	SD	MET	13	36.086	49.482	52.262	1.00	0.00	S
ATOM	210	CE	MET	13	34.865	49.947	53.513	1.00	0.00	C
ATOM	211	HE1	MET	13	34.409	49.045	53.919	1.00	0.00	H
ATOM	212	HE2	MET	13	35.358	50.485	54.321	1.00	0.00	H
ATOM	213	HE3	MET	13	34.090	50.572	53.072	1.00	0.00	H
ATOM	214	C	MET	13	37.331	52.343	49.026	1.00	0.00	C
ATOM	215	O	MET	13	37.379	51.430	48.198	1.00	0.00	O
ATOM	216	N	PHE	14	38.420	52.860	49.595	1.00	0.00	N
ATOM	217	H	PHE	14	38.317	53.684	50.183	1.00	0.00	H
ATOM	218	CA	PHE	14	39.684	52.127	49.719	1.00	0.00	C
ATOM	219	HA	PHE	14	39.493	51.063	49.571	1.00	0.00	H
ATOM	220	CB	PHE	14	40.658	52.593	48.624	1.00	0.00	C
ATOM	221	HB2	PHE	14	40.088	52.835	47.726	1.00	0.00	H
ATOM	222	HB3	PHE	14	41.148	53.513	48.942	1.00	0.00	H
ATOM	223	CG	PHE	14	41.705	51.566	48.233	1.00	0.00	C
ATOM	224	CD1	PHE	14	42.952	51.519	48.884	1.00	0.00	C
ATOM	225	HD1	PHE	14	43.181	52.207	49.684	1.00	0.00	H
ATOM	226	CE1	PHE	14	43.918	50.575	48.496	1.00	0.00	C
ATOM	227	HE1	PHE	14	44.872	50.551	49.010	1.00	0.00	H
ATOM	228	CZ	PHE	14	43.641	49.666	47.460	1.00	0.00	C
ATOM	229	HZ	PHE	14	44.382	48.933	47.172	1.00	0.00	H
ATOM	230	CE2	PHE	14	42.397	49.710	46.804	1.00	0.00	C
ATOM	231	HE2	PHE	14	42.177	49.014	46.006	1.00	0.00	H
ATOM	232	CD2	PHE	14	41.433	50.660	47.189	1.00	0.00	C
ATOM	233	HD2	PHE	14	40.479	50.693	46.683	1.00	0.00	H
ATOM	234	C	PHE	14	40.224	52.319	51.149	1.00	0.00	C
ATOM	235	O	PHE	14	40.171	53.433	51.669	1.00	0.00	O
ATOM	236	N	CYS	15	40.693	51.265	51.821	1.00	0.00	N
ATOM	237	H	CYS	15	40.680	50.349	51.384	1.00	0.00	H
ATOM	238	CA	CYS	15	41.201	51.342	53.202	1.00	0.00	C
ATOM	239	HA	CYS	15	41.683	52.309	53.340	1.00	0.00	H
ATOM	240	CB	CYS	15	40.023	51.265	54.188	1.00	0.00	C
ATOM	241	HB2	CYS	15	40.391	51.458	55.197	1.00	0.00	H
ATOM	242	HB3	CYS	15	39.292	52.036	53.936	1.00	0.00	H
ATOM	243	SG	CYS	15	39.219	49.638	54.154	1.00	0.00	S
ATOM	244	HG	CYS	15	38.922	49.623	52.846	1.00	0.00	H
ATOM	245	C	CYS	15	42.278	50.283	53.484	1.00	0.00	C
ATOM	246	O	CYS	15	42.324	49.264	52.797	1.00	0.00	O
ATOM	247	N	ASP	16	43.162	50.540	54.453	1.00	0.00	N
ATOM	248	H	ASP	16	43.052	51.397	54.988	1.00	0.00	H
ATOM	249	CA	ASP	16	44.401	49.775	54.686	1.00	0.00	C
ATOM	250	HA	ASP	16	44.176	48.713	54.577	1.00	0.00	H
ATOM	251	CB	ASP	16	45.384	50.162	53.537	1.00	0.00	C
ATOM	252	HB2	ASP	16	45.132	49.577	52.651	1.00	0.00	H
ATOM	253	HB3	ASP	16	45.224	51.211	53.284	1.00	0.00	H
ATOM	254	CG	ASP	16	46.872	50.017	53.825	1.00	0.00	C
ATOM	255	OD1	ASP	16	47.655	49.351	53.082	1.00	0.00	O
ATOM	256	OD2	ASP	16	47.339	50.526	54.860	1.00	0.00	O
ATOM	257	C	ASP	16	44.923	49.963	56.147	1.00	0.00	C
ATOM	258	O	ASP	16	44.650	50.975	56.799	1.00	0.00	O

ATOM	259	N	ILE	17	45.659	48.977	56.692	1.00	0.00	N
ATOM	260	H	ILE	17	45.887	48.183	56.105	1.00	0.00	H
ATOM	261	CA	ILE	17	46.224	48.987	58.064	1.00	0.00	C
ATOM	262	HA	ILE	17	45.499	49.452	58.734	1.00	0.00	H
ATOM	263	CB	ILE	17	46.475	47.536	58.560	1.00	0.00	C
ATOM	264	HB	ILE	17	47.222	47.084	57.908	1.00	0.00	H
ATOM	265	CG2	ILE	17	47.038	47.542	59.997	1.00	0.00	C
ATOM	266	HG21	ILE	17	47.966	48.111	60.054	1.00	0.00	H
ATOM	267	HG22	ILE	17	47.258	46.531	60.330	1.00	0.00	H
ATOM	268	HG23	ILE	17	46.306	47.980	60.674	1.00	0.00	H
ATOM	269	CG1	ILE	17	45.203	46.658	58.485	1.00	0.00	C
ATOM	270	HG12	ILE	17	44.442	47.060	59.154	1.00	0.00	H
ATOM	271	HG13	ILE	17	44.808	46.685	57.469	1.00	0.00	H
ATOM	272	CD1	ILE	17	45.446	45.179	58.825	1.00	0.00	C
ATOM	273	HD11	ILE	17	46.308	44.805	58.274	1.00	0.00	H
ATOM	274	HD12	ILE	17	44.573	44.594	58.540	1.00	0.00	H
ATOM	275	HD13	ILE	17	45.610	45.053	59.895	1.00	0.00	H
ATOM	276	C	ILE	17	47.535	49.798	58.115	1.00	0.00	C
ATOM	277	O	ILE	17	48.496	49.449	57.423	1.00	0.00	O
ATOM	278	N	LYS	18	47.664	50.848	58.937	1.00	0.00	N
ATOM	279	H	LYS	18	46.916	51.023	59.599	1.00	0.00	H
ATOM	280	CA	LYS	18	48.862	51.727	58.918	1.00	0.00	C
ATOM	281	HA	LYS	18	48.962	52.119	57.905	1.00	0.00	H
ATOM	282	CB	LYS	18	48.679	52.919	59.876	1.00	0.00	C
ATOM	283	HB2	LYS	18	47.686	53.352	59.743	1.00	0.00	H
ATOM	284	HB3	LYS	18	48.755	52.550	60.895	1.00	0.00	H
ATOM	285	CG	LYS	18	49.739	54.019	59.646	1.00	0.00	C
ATOM	286	HG2	LYS	18	50.722	53.579	59.476	1.00	0.00	H
ATOM	287	HG3	LYS	18	49.463	54.579	58.752	1.00	0.00	H
ATOM	288	CD	LYS	18	49.855	54.990	60.829	1.00	0.00	C
ATOM	289	HD2	LYS	18	50.372	55.893	60.498	1.00	0.00	H
ATOM	290	HD3	LYS	18	48.854	55.266	61.163	1.00	0.00	H
ATOM	291	CE	LYS	18	50.655	54.381	61.991	1.00	0.00	C
ATOM	292	HE2	LYS	18	50.315	53.355	62.162	1.00	0.00	H
ATOM	293	HE3	LYS	18	51.712	54.351	61.714	1.00	0.00	H
ATOM	294	NZ	LYS	18	50.473	55.165	63.235	1.00	0.00	N
ATOM	295	HZ1	LYS	18	51.070	54.855	63.985	1.00	0.00	H
ATOM	296	HZ2	LYS	18	49.498	55.080	63.533	1.00	0.00	H
ATOM	297	HZ3	LYS	18	50.591	56.167	63.089	1.00	0.00	H
ATOM	298	C	LYS	18	50.160	50.971	59.271	1.00	0.00	C
ATOM	299	O	LYS	18	50.264	50.408	60.355	1.00	0.00	O
ATOM	300	N	GLY	19	51.173	51.035	58.397	1.00	0.00	N
ATOM	301	H	GLY	19	51.017	51.468	57.500	1.00	0.00	H
ATOM	302	CA	GLY	19	52.535	50.551	58.681	1.00	0.00	C
ATOM	303	HA2	GLY	19	53.182	50.846	57.854	1.00	0.00	H
ATOM	304	HA3	GLY	19	52.903	51.022	59.594	1.00	0.00	H
ATOM	305	C	GLY	19	52.674	49.031	58.843	1.00	0.00	C
ATOM	306	O	GLY	19	53.560	48.565	59.560	1.00	0.00	O
ATOM	307	N	PHE	20	51.806	48.255	58.190	1.00	0.00	N
ATOM	308	H	PHE	20	51.245	48.703	57.477	1.00	0.00	H
ATOM	309	CA	PHE	20	51.728	46.798	58.310	1.00	0.00	C
ATOM	310	HA	PHE	20	51.601	46.558	59.365	1.00	0.00	H

ATOM	311	CB	PHE	20	50.474	46.306	57.577	1.00	0.00	C
ATOM	312	HB2	PHE	20	49.616	46.866	57.948	1.00	0.00	H
ATOM	313	HB3	PHE	20	50.567	46.533	56.519	1.00	0.00	H
ATOM	314	CG	PHE	20	50.184	44.825	57.741	1.00	0.00	C
ATOM	315	CD1	PHE	20	49.296	44.388	58.742	1.00	0.00	C
ATOM	316	HD1	PHE	20	48.819	45.106	59.389	1.00	0.00	H
ATOM	317	CE1	PHE	20	49.020	43.019	58.894	1.00	0.00	C
ATOM	318	HE1	PHE	20	48.329	42.686	59.654	1.00	0.00	H
ATOM	319	CZ	PHE	20	49.633	42.083	58.044	1.00	0.00	C
ATOM	320	HZ	PHE	20	49.411	41.033	58.148	1.00	0.00	H
ATOM	321	CE2	PHE	20	50.521	42.511	57.045	1.00	0.00	C
ATOM	322	HE2	PHE	20	50.987	41.793	56.386	1.00	0.00	H
ATOM	323	CD2	PHE	20	50.795	43.880	56.895	1.00	0.00	C
ATOM	324	HD2	PHE	20	51.481	44.204	56.126	1.00	0.00	H
ATOM	325	C	PHE	20	53.016	46.086	57.852	1.00	0.00	C
ATOM	326	O	PHE	20	53.345	45.032	58.404	1.00	0.00	O
ATOM	327	N	THR	21	53.821	46.715	56.979	1.00	0.00	N
ATOM	328	H	THR	21	53.469	47.541	56.513	1.00	0.00	H
ATOM	329	CA	THR	21	55.207	46.301	56.684	1.00	0.00	C
ATOM	330	HA	THR	21	55.185	45.410	56.056	1.00	0.00	H
ATOM	331	CB	THR	21	55.980	47.402	55.925	1.00	0.00	C
ATOM	332	HB	THR	21	55.891	48.343	56.469	1.00	0.00	H
ATOM	333	CG2	THR	21	57.466	47.092	55.753	1.00	0.00	C
ATOM	334	HG21	THR	21	57.983	47.239	56.700	1.00	0.00	H
ATOM	335	HG22	THR	21	57.899	47.765	55.013	1.00	0.00	H
ATOM	336	HG23	THR	21	57.591	46.060	55.432	1.00	0.00	H
ATOM	337	OG1	THR	21	55.467	47.592	54.633	1.00	0.00	O
ATOM	338	HG1	THR	21	54.819	48.334	54.710	1.00	0.00	H
ATOM	339	C	THR	21	55.963	45.957	57.971	1.00	0.00	C
ATOM	340	O	THR	21	56.409	44.821	58.135	1.00	0.00	O
ATOM	341	N	SER	22	56.011	46.889	58.932	1.00	0.00	N
ATOM	342	H	SER	22	55.508	47.756	58.792	1.00	0.00	H
ATOM	343	CA	SER	22	56.762	46.743	60.188	1.00	0.00	C
ATOM	344	HA	SER	22	57.771	46.418	59.945	1.00	0.00	H
ATOM	345	CB	SER	22	56.874	48.093	60.907	1.00	0.00	C
ATOM	346	HB2	SER	22	55.879	48.436	61.198	1.00	0.00	H
ATOM	347	HB3	SER	22	57.482	47.972	61.805	1.00	0.00	H
ATOM	348	OG	SER	22	57.480	49.060	60.062	1.00	0.00	O
ATOM	349	HG	SER	22	58.223	48.625	59.620	1.00	0.00	H
ATOM	350	C	SER	22	56.195	45.692	61.150	1.00	0.00	C
ATOM	351	O	SER	22	56.880	45.305	62.094	1.00	0.00	O
ATOM	352	N	LEU	23	54.970	45.212	60.919	1.00	0.00	N
ATOM	353	H	LEU	23	54.471	45.557	60.108	1.00	0.00	H
ATOM	354	CA	LEU	23	54.391	44.084	61.650	1.00	0.00	C
ATOM	355	HA	LEU	23	54.771	44.091	62.673	1.00	0.00	H
ATOM	356	CB	LEU	23	52.857	44.243	61.711	1.00	0.00	C
ATOM	357	HB2	LEU	23	52.446	44.046	60.721	1.00	0.00	H
ATOM	358	HB3	LEU	23	52.461	43.491	62.395	1.00	0.00	H
ATOM	359	CG	LEU	23	52.363	45.637	62.158	1.00	0.00	C
ATOM	360	HG	LEU	23	52.674	46.383	61.427	1.00	0.00	H
ATOM	361	CD1	LEU	23	50.837	45.655	62.221	1.00	0.00	C
ATOM	362	HD11	LEU	23	50.425	45.316	61.272	1.00	0.00	H

```
ATOM    363 HD12 LEU    23      50.492  46.672  62.410  1.00  0.00           H
ATOM    364 HD13 LEU    23      50.493  45.004  63.022  1.00  0.00           H
ATOM    365  CD2 LEU    23      52.892  46.054  63.533  1.00  0.00           C
ATOM    366 HD21 LEU    23      52.592  45.329  64.287  1.00  0.00           H
ATOM    367 HD22 LEU    23      53.978  46.130  63.510  1.00  0.00           H
ATOM    368 HD23 LEU    23      52.490  47.032  63.797  1.00  0.00           H
ATOM    369  C   LEU    23      54.845  42.756  61.016  1.00  0.00           C
ATOM    370  O   LEU    23      55.294  41.853  61.727  1.00  0.00           O
ATOM    371  N   SER    24      54.822  42.666  59.679  1.00  0.00           N
ATOM    372  H   SER    24      54.445  43.452  59.158  1.00  0.00           H
ATOM    373  CA  SER    24      55.355  41.516  58.925  1.00  0.00           C
ATOM    374  HA  SER    24      54.892  40.615  59.323  1.00  0.00           H
ATOM    375  CB  SER    24      54.954  41.631  57.445  1.00  0.00           C
ATOM    376  HB2 SER    24      55.055  40.647  56.984  1.00  0.00           H
ATOM    377  HB3 SER    24      53.906  41.928  57.380  1.00  0.00           H
ATOM    378  OG  SER    24      55.743  42.545  56.704  1.00  0.00           O
ATOM    379  HG  SER    24      55.908  43.350  57.228  1.00  0.00           H
ATOM    380  C   SER    24      56.875  41.332  59.089  1.00  0.00           C
ATOM    381  O   SER    24      57.370  40.205  59.125  1.00  0.00           O
ATOM    382  N   ASP    25      57.605  42.431  59.296  1.00  0.00           N
ATOM    383  H   ASP    25      57.152  43.326  59.151  1.00  0.00           H
ATOM    384  CA  ASP    25      59.045  42.451  59.584  1.00  0.00           C
ATOM    385  HA  ASP    25      59.576  41.931  58.785  1.00  0.00           H
ATOM    386  CB  ASP    25      59.536  43.912  59.635  1.00  0.00           C
ATOM    387  HB2 ASP    25      58.943  44.452  60.374  1.00  0.00           H
ATOM    388  HB3 ASP    25      60.571  43.922  59.980  1.00  0.00           H
ATOM    389  CG  ASP    25      59.509  44.686  58.318  1.00  0.00           C
ATOM    390  OD1 ASP    25      59.528  44.074  57.227  1.00  0.00           O
ATOM    391  OD2 ASP    25      59.574  45.935  58.383  1.00  0.00           O
ATOM    392  C   ASP    25      59.440  41.784  60.917  1.00  0.00           C
ATOM    393  O   ASP    25      60.634  41.583  61.150  1.00  0.00           O
ATOM    394  N   LYS    26      58.489  41.520  61.831  1.00  0.00           N
ATOM    395  H   LYS    26      57.529  41.716  61.574  1.00  0.00           H
ATOM    396  CA  LYS    26      58.807  41.185  63.235  1.00  0.00           C
ATOM    397  HA  LYS    26      59.844  40.851  63.284  1.00  0.00           H
ATOM    398  CB  LYS    26      58.667  42.448  64.116  1.00  0.00           C
ATOM    399  HB2 LYS    26      57.618  42.750  64.117  1.00  0.00           H
ATOM    400  HB3 LYS    26      58.937  42.195  65.143  1.00  0.00           H
ATOM    401  CG  LYS    26      59.506  43.669  63.683  1.00  0.00           C
ATOM    402  HG2 LYS    26      59.245  43.931  62.659  1.00  0.00           H
ATOM    403  HG3 LYS    26      59.222  44.518  64.307  1.00  0.00           H
ATOM    404  CD  LYS    26      61.030  43.485  63.792  1.00  0.00           C
ATOM    405  HD2 LYS    26      61.327  43.572  64.838  1.00  0.00           H
ATOM    406  HD3 LYS    26      61.324  42.501  63.428  1.00  0.00           H
ATOM    407  CE  LYS    26      61.734  44.556  62.947  1.00  0.00           C
ATOM    408  HE2 LYS    26      61.450  44.405  61.901  1.00  0.00           H
ATOM    409  HE3 LYS    26      61.378  45.542  63.256  1.00  0.00           H
ATOM    410  NZ  LYS    26      63.209  44.491  63.077  1.00  0.00           N
ATOM    411  HZ1 LYS    26      63.670  45.183  62.489  1.00  0.00           H
ATOM    412  HZ2 LYS    26      63.571  43.572  62.839  1.00  0.00           H
ATOM    413  HZ3 LYS    26      63.497  44.657  64.038  1.00  0.00           H
ATOM    414  C   LYS    26      58.006  40.029  63.841  1.00  0.00           C
```

ATOM	415	O	LYS	26	58.559	39.341	64.706	1.00	0.00	O
ATOM	416	N	MET	27	56.743	39.823	63.455	1.00	0.00	N
ATOM	417	H	MET	27	56.368	40.394	62.707	1.00	0.00	H
ATOM	418	CA	MET	27	55.794	39.010	64.238	1.00	0.00	C
ATOM	419	HA	MET	27	56.241	38.856	65.215	1.00	0.00	H
ATOM	420	CB	MET	27	54.501	39.802	64.476	1.00	0.00	C
ATOM	421	HB2	MET	27	53.985	39.947	63.526	1.00	0.00	H
ATOM	422	HB3	MET	27	53.846	39.227	65.132	1.00	0.00	H
ATOM	423	CG	MET	27	54.762	41.176	65.110	1.00	0.00	C
ATOM	424	HG2	MET	27	55.260	41.809	64.376	1.00	0.00	H
ATOM	425	HG3	MET	27	53.802	41.634	65.321	1.00	0.00	H
ATOM	426	SD	MET	27	55.757	41.205	66.634	1.00	0.00	S
ATOM	427	CE	MET	27	54.681	40.312	67.791	1.00	0.00	C
ATOM	428	HE1	MET	27	53.732	40.836	67.901	1.00	0.00	H
ATOM	429	HE2	MET	27	54.500	39.299	67.433	1.00	0.00	H
ATOM	430	HE3	MET	27	55.170	40.260	68.764	1.00	0.00	H
ATOM	431	C	MET	27	55.489	37.618	63.642	1.00	0.00	C
ATOM	432	O	MET	27	55.473	37.473	62.420	1.00	0.00	O
ATOM	433	N	PRO	28	55.199	36.589	64.474	1.00	0.00	N
ATOM	434	CD	PRO	28	55.270	36.581	65.932	1.00	0.00	C
ATOM	435	HD2	PRO	28	54.331	36.953	66.344	1.00	0.00	H
ATOM	436	HD3	PRO	28	56.108	37.163	66.314	1.00	0.00	H
ATOM	437	CG	PRO	28	55.465	35.119	66.325	1.00	0.00	C
ATOM	438	HG2	PRO	28	55.084	34.913	67.325	1.00	0.00	H
ATOM	439	HG3	PRO	28	56.521	34.856	66.253	1.00	0.00	H
ATOM	440	CB	PRO	28	54.675	34.380	65.250	1.00	0.00	C
ATOM	441	HB2	PRO	28	53.621	34.368	65.529	1.00	0.00	H
ATOM	442	HB3	PRO	28	55.042	33.363	65.108	1.00	0.00	H
ATOM	443	CA	PRO	28	54.879	35.240	63.997	1.00	0.00	C
ATOM	444	HA	PRO	28	55.742	34.863	63.446	1.00	0.00	H
ATOM	445	C	PRO	28	53.641	35.184	63.092	1.00	0.00	C
ATOM	446	O	PRO	28	52.660	35.897	63.313	1.00	0.00	O
ATOM	447	N	LEU	29	53.652	34.279	62.108	1.00	0.00	N
ATOM	448	H	LEU	29	54.481	33.716	61.998	1.00	0.00	H
ATOM	449	CA	LEU	29	52.633	34.208	61.053	1.00	0.00	C
ATOM	450	HA	LEU	29	52.654	35.170	60.548	1.00	0.00	H
ATOM	451	CB	LEU	29	53.029	33.132	60.019	1.00	0.00	C
ATOM	452	HB2	LEU	29	52.434	33.320	59.126	1.00	0.00	H
ATOM	453	HB3	LEU	29	54.071	33.294	59.750	1.00	0.00	H
ATOM	454	CG	LEU	29	52.832	31.643	60.379	1.00	0.00	C
ATOM	455	HG	LEU	29	51.780	31.457	60.593	1.00	0.00	H
ATOM	456	CD1	LEU	29	53.226	30.788	59.173	1.00	0.00	C
ATOM	457	HD11	LEU	29	52.661	31.100	58.296	1.00	0.00	H
ATOM	458	HD12	LEU	29	52.994	29.741	59.377	1.00	0.00	H
ATOM	459	HD13	LEU	29	54.292	30.883	58.974	1.00	0.00	H
ATOM	460	CD2	LEU	29	53.679	31.167	61.564	1.00	0.00	C
ATOM	461	HD21	LEU	29	54.733	31.384	61.385	1.00	0.00	H
ATOM	462	HD22	LEU	29	53.356	31.646	62.486	1.00	0.00	H
ATOM	463	HD23	LEU	29	53.553	30.092	61.686	1.00	0.00	H
ATOM	464	C	LEU	29	51.192	34.058	61.566	1.00	0.00	C
ATOM	465	O	LEU	29	50.302	34.790	61.136	1.00	0.00	O
ATOM	466	N	THR	30	50.964	33.183	62.546	1.00	0.00	N

ATOM	467	H	THR	30	51.714	32.580	62.850	1.00	0.00	H
ATOM	468	CA	THR	30	49.641	32.973	63.155	1.00	0.00	C
ATOM	469	HA	THR	30	48.918	32.767	62.366	1.00	0.00	H
ATOM	470	CB	THR	30	49.693	31.754	64.094	1.00	0.00	C
ATOM	471	HB	THR	30	50.327	31.988	64.947	1.00	0.00	H
ATOM	472	CG2	THR	30	48.324	31.310	64.608	1.00	0.00	C
ATOM	473	HG21	THR	30	47.654	31.124	63.768	1.00	0.00	H
ATOM	474	HG22	THR	30	47.896	32.080	65.249	1.00	0.00	H
ATOM	475	HG23	THR	30	48.429	30.396	65.194	1.00	0.00	H
ATOM	476	OG1	THR	30	50.248	30.652	63.414	1.00	0.00	O
ATOM	477	HG1	THR	30	50.285	29.926	64.045	1.00	0.00	H
ATOM	478	C	THR	30	49.157	34.212	63.921	1.00	0.00	C
ATOM	479	O	THR	30	47.954	34.432	64.047	1.00	0.00	O
ATOM	480	N	HID	31	50.078	35.041	64.426	1.00	0.00	N
ATOM	481	H	HID	31	51.052	34.870	64.213	1.00	0.00	H
ATOM	482	CA	HID	31	49.754	36.272	65.153	1.00	0.00	C
ATOM	483	HA	HID	31	48.856	36.106	65.750	1.00	0.00	H
ATOM	484	CB	HID	31	50.897	36.666	66.120	1.00	0.00	C
ATOM	485	HB2	HID	31	51.760	36.975	65.529	1.00	0.00	H
ATOM	486	HB3	HID	31	50.568	37.544	66.679	1.00	0.00	H
ATOM	487	CG	HID	31	51.387	35.634	67.130	1.00	0.00	C
ATOM	488	ND1	HID	31	52.215	35.895	68.206	1.00	0.00	N
ATOM	489	HD1	HID	31	52.495	36.803	68.581	1.00	0.00	H
ATOM	490	CE1	HID	31	52.523	34.732	68.803	1.00	0.00	C
ATOM	491	HE1	HID	31	53.137	34.641	69.691	1.00	0.00	H
ATOM	492	NE2	HID	31	51.953	33.705	68.157	1.00	0.00	N
ATOM	493	CD2	HID	31	51.212	34.271	67.117	1.00	0.00	C
ATOM	494	HD2	HID	31	50.631	33.702	66.411	1.00	0.00	H
ATOM	495	C	HID	31	49.438	37.403	64.156	1.00	0.00	C
ATOM	496	O	HID	31	48.494	38.163	64.362	1.00	0.00	O
ATOM	497	N	LEU	32	50.185	37.482	63.046	1.00	0.00	N
ATOM	498	H	LEU	32	50.964	36.838	62.950	1.00	0.00	H
ATOM	499	CA	LEU	32	49.991	38.485	61.998	1.00	0.00	C
ATOM	500	HA	LEU	32	49.916	39.458	62.484	1.00	0.00	H
ATOM	501	CB	LEU	32	51.230	38.481	61.082	1.00	0.00	C
ATOM	502	HB2	LEU	32	52.141	38.440	61.683	1.00	0.00	H
ATOM	503	HB3	LEU	32	51.197	37.584	60.462	1.00	0.00	H
ATOM	504	CG	LEU	32	51.279	39.726	60.180	1.00	0.00	C
ATOM	505	HG	LEU	32	50.283	39.935	59.805	1.00	0.00	H
ATOM	506	CD1	LEU	32	51.786	40.948	60.944	1.00	0.00	C
ATOM	507	HD11	LEU	32	51.134	41.165	61.786	1.00	0.00	H
ATOM	508	HD12	LEU	32	51.789	41.810	60.277	1.00	0.00	H
ATOM	509	HD13	LEU	32	52.796	40.763	61.308	1.00	0.00	H
ATOM	510	CD2	LEU	32	52.196	39.505	58.981	1.00	0.00	C
ATOM	511	HD21	LEU	32	53.181	39.190	59.321	1.00	0.00	H
ATOM	512	HD22	LEU	32	51.768	38.739	58.337	1.00	0.00	H
ATOM	513	HD23	LEU	32	52.279	40.426	58.406	1.00	0.00	H
ATOM	514	C	LEU	32	48.688	38.273	61.204	1.00	0.00	C
ATOM	515	O	LEU	32	47.943	39.227	60.979	1.00	0.00	O
ATOM	516	N	LEU	33	48.365	37.029	60.830	1.00	0.00	N
ATOM	517	H	LEU	33	49.034	36.284	61.006	1.00	0.00	H
ATOM	518	CA	LEU	33	47.117	36.700	60.123	1.00	0.00	C

ATOM	519	HA	LEU	33	47.058	37.308	59.220	1.00	0.00	H
ATOM	520	CB	LEU	33	47.128	35.214	59.720	1.00	0.00	C
ATOM	521	HB2	LEU	33	47.378	34.621	60.600	1.00	0.00	H
ATOM	522	HB3	LEU	33	46.128	34.921	59.394	1.00	0.00	H
ATOM	523	CG	LEU	33	48.125	34.903	58.588	1.00	0.00	C
ATOM	524	HG	LEU	33	49.066	35.412	58.783	1.00	0.00	H
ATOM	525	CD1	LEU	33	48.403	33.404	58.522	1.00	0.00	C
ATOM	526	HD11	LEU	33	48.810	33.063	59.473	1.00	0.00	H
ATOM	527	HD12	LEU	33	49.136	33.218	57.739	1.00	0.00	H
ATOM	528	HD13	LEU	33	47.481	32.864	58.300	1.00	0.00	H
ATOM	529	CD2	LEU	33	47.602	35.345	57.218	1.00	0.00	C
ATOM	530	HD21	LEU	33	46.670	34.828	56.984	1.00	0.00	H
ATOM	531	HD22	LEU	33	47.425	36.417	57.208	1.00	0.00	H
ATOM	532	HD23	LEU	33	48.340	35.112	56.450	1.00	0.00	H
ATOM	533	C	LEU	33	45.868	37.043	60.951	1.00	0.00	C
ATOM	534	O	LEU	33	44.888	37.537	60.396	1.00	0.00	O
ATOM	535	N	LYS	34	45.920	36.885	62.282	1.00	0.00	N
ATOM	536	H	LYS	34	46.756	36.479	62.681	1.00	0.00	H
ATOM	537	CA	LYS	34	44.837	37.312	63.187	1.00	0.00	C
ATOM	538	HA	LYS	34	43.901	36.875	62.831	1.00	0.00	H
ATOM	539	CB	LYS	34	45.099	36.799	64.615	1.00	0.00	C
ATOM	540	HB2	LYS	34	46.143	36.967	64.884	1.00	0.00	H
ATOM	541	HB3	LYS	34	44.473	37.359	65.313	1.00	0.00	H
ATOM	542	CG	LYS	34	44.751	35.307	64.758	1.00	0.00	C
ATOM	543	HG2	LYS	34	43.694	35.170	64.527	1.00	0.00	H
ATOM	544	HG3	LYS	34	45.337	34.723	64.048	1.00	0.00	H
ATOM	545	CD	LYS	34	45.020	34.797	66.182	1.00	0.00	C
ATOM	546	HD2	LYS	34	46.087	34.885	66.399	1.00	0.00	H
ATOM	547	HD3	LYS	34	44.460	35.407	66.894	1.00	0.00	H
ATOM	548	CE	LYS	34	44.583	33.332	66.312	1.00	0.00	C
ATOM	549	HE2	LYS	34	43.528	33.255	66.028	1.00	0.00	H
ATOM	550	HE3	LYS	34	45.163	32.730	65.607	1.00	0.00	H
ATOM	551	NZ	LYS	34	44.767	32.820	67.694	1.00	0.00	N
ATOM	552	HZ1	LYS	34	45.727	32.874	68.000	1.00	0.00	H
ATOM	553	HZ2	LYS	34	44.446	31.867	67.791	1.00	0.00	H
ATOM	554	HZ3	LYS	34	44.241	33.365	68.381	1.00	0.00	H
ATOM	555	C	LYS	34	44.587	38.829	63.176	1.00	0.00	C
ATOM	556	O	LYS	34	43.469	39.241	63.479	1.00	0.00	O
ATOM	557	N	LEU	35	45.565	39.662	62.793	1.00	0.00	N
ATOM	558	H	LEU	35	46.463	39.271	62.540	1.00	0.00	H
ATOM	559	CA	LEU	35	45.315	41.089	62.530	1.00	0.00	C
ATOM	560	HA	LEU	35	44.743	41.512	63.358	1.00	0.00	H
ATOM	561	CB	LEU	35	46.615	41.891	62.358	1.00	0.00	C
ATOM	562	HB2	LEU	35	47.076	41.608	61.412	1.00	0.00	H
ATOM	563	HB3	LEU	35	46.339	42.944	62.276	1.00	0.00	H
ATOM	564	CG	LEU	35	47.683	41.754	63.445	1.00	0.00	C
ATOM	565	HG	LEU	35	48.066	40.735	63.459	1.00	0.00	H
ATOM	566	CD1	LEU	35	48.813	42.708	63.058	1.00	0.00	C
ATOM	567	HD11	LEU	35	49.125	42.522	62.031	1.00	0.00	H
ATOM	568	HD12	LEU	35	49.673	42.550	63.695	1.00	0.00	H
ATOM	569	HD13	LEU	35	48.478	43.741	63.149	1.00	0.00	H
ATOM	570	CD2	LEU	35	47.136	42.110	64.828	1.00	0.00	C

ATOM	571	HD21	LEU	35	46.761	43.133	64.834	1.00	0.00	H
ATOM	572	HD22	LEU	35	46.337	41.420	65.093	1.00	0.00	H
ATOM	573	HD23	LEU	35	47.922	42.008	65.570	1.00	0.00	H
ATOM	574	C	LEU	35	44.494	41.284	61.255	1.00	0.00	C
ATOM	575	O	LEU	35	43.502	42.006	61.265	1.00	0.00	O
ATOM	576	N	LEU	36	44.925	40.642	60.165	1.00	0.00	N
ATOM	577	H	LEU	36	45.730	40.037	60.263	1.00	0.00	H
ATOM	578	CA	LEU	36	44.316	40.771	58.842	1.00	0.00	C
ATOM	579	HA	LEU	36	44.302	41.823	58.556	1.00	0.00	H
ATOM	580	CB	LEU	36	45.148	39.971	57.823	1.00	0.00	C
ATOM	581	HB2	LEU	36	45.317	38.969	58.220	1.00	0.00	H
ATOM	582	HB3	LEU	36	44.575	39.861	56.907	1.00	0.00	H
ATOM	583	CG	LEU	36	46.498	40.605	57.458	1.00	0.00	C
ATOM	584	HG	LEU	36	47.011	40.918	58.366	1.00	0.00	H
ATOM	585	CD1	LEU	36	47.364	39.569	56.740	1.00	0.00	C
ATOM	586	HD11	LEU	36	47.633	38.777	57.437	1.00	0.00	H
ATOM	587	HD12	LEU	36	48.276	40.033	56.368	1.00	0.00	H
ATOM	588	HD13	LEU	36	46.810	39.145	55.903	1.00	0.00	H
ATOM	589	CD2	LEU	36	46.324	41.810	56.530	1.00	0.00	C
ATOM	590	HD21	LEU	36	45.825	41.506	55.609	1.00	0.00	H
ATOM	591	HD22	LEU	36	45.734	42.582	57.020	1.00	0.00	H
ATOM	592	HD23	LEU	36	47.299	42.231	56.284	1.00	0.00	H
ATOM	593	C	LEU	36	42.860	40.297	58.851	1.00	0.00	C
ATOM	594	O	LEU	36	41.985	41.012	58.368	1.00	0.00	O
ATOM	595	N	GLU	37	42.586	39.126	59.432	1.00	0.00	N
ATOM	596	H	GLU	37	43.354	38.565	59.790	1.00	0.00	H
ATOM	597	CA	GLU	37	41.222	38.592	59.512	1.00	0.00	C
ATOM	598	HA	GLU	37	40.765	38.670	58.524	1.00	0.00	H
ATOM	599	CB	GLU	37	41.234	37.093	59.876	1.00	0.00	C
ATOM	600	HB2	GLU	37	41.718	36.943	60.842	1.00	0.00	H
ATOM	601	HB3	GLU	37	40.197	36.761	59.951	1.00	0.00	H
ATOM	602	CG	GLU	37	41.949	36.255	58.795	1.00	0.00	C
ATOM	603	HG2	GLU	37	41.850	36.765	57.833	1.00	0.00	H
ATOM	604	HG3	GLU	37	43.014	36.208	59.024	1.00	0.00	H
ATOM	605	CD	GLU	37	41.400	34.828	58.632	1.00	0.00	C
ATOM	606	OE1	GLU	37	41.125	34.132	59.645	1.00	0.00	O
ATOM	607	OE2	GLU	37	41.287	34.378	57.464	1.00	0.00	O
ATOM	608	C	GLU	37	40.321	39.421	60.445	1.00	0.00	C
ATOM	609	O	GLU	37	39.171	39.663	60.088	1.00	0.00	O
ATOM	610	N	GLN	38	40.817	39.946	61.580	1.00	0.00	N
ATOM	611	H	GLN	38	41.771	39.745	61.848	1.00	0.00	H
ATOM	612	CA	GLN	38	40.006	40.840	62.428	1.00	0.00	C
ATOM	613	HA	GLN	38	39.023	40.380	62.527	1.00	0.00	H
ATOM	614	CB	GLN	38	40.579	40.926	63.856	1.00	0.00	C
ATOM	615	HB2	GLN	38	40.674	39.913	64.250	1.00	0.00	H
ATOM	616	HB3	GLN	38	41.576	41.367	63.818	1.00	0.00	H
ATOM	617	CG	GLN	38	39.692	41.765	64.809	1.00	0.00	C
ATOM	618	HG2	GLN	38	40.211	42.692	65.055	1.00	0.00	H
ATOM	619	HG3	GLN	38	38.756	42.028	64.319	1.00	0.00	H
ATOM	620	CD	GLN	38	39.312	41.047	66.101	1.00	0.00	C
ATOM	621	OE1	GLN	38	39.856	41.304	67.172	1.00	0.00	O
ATOM	622	NE2	GLN	38	38.365	40.137	66.059	1.00	0.00	N

```
ATOM    623 HE21 GLN    38      38.085  39.655  66.899  1.00  0.00           H
ATOM    624 HE22 GLN    38      37.855  39.965  65.183  1.00  0.00           H
ATOM    625  C   GLN    38      39.784  42.230  61.791  1.00  0.00           C
ATOM    626  O   GLN    38      38.703  42.800  61.929  1.00  0.00           O
ATOM    627  N   PHE    39      40.752  42.782  61.053  1.00  0.00           N
ATOM    628  H   PHE    39      41.648  42.310  60.979  1.00  0.00           H
ATOM    629  CA  PHE    39      40.547  44.021  60.289  1.00  0.00           C
ATOM    630  HA  PHE    39      40.173  44.793  60.962  1.00  0.00           H
ATOM    631  CB  PHE    39      41.883  44.497  59.704  1.00  0.00           C
ATOM    632  HB2 PHE    39      42.565  44.725  60.525  1.00  0.00           H
ATOM    633  HB3 PHE    39      42.326  43.684  59.127  1.00  0.00           H
ATOM    634  CG  PHE    39      41.766  45.725  58.818  1.00  0.00           C
ATOM    635  CD1 PHE    39      41.435  46.971  59.385  1.00  0.00           C
ATOM    636  HD1 PHE    39      41.260  47.055  60.447  1.00  0.00           H
ATOM    637  CE1 PHE    39      41.327  48.111  58.570  1.00  0.00           C
ATOM    638  HE1 PHE    39      41.066  49.064  59.005  1.00  0.00           H
ATOM    639  CZ  PHE    39      41.554  48.015  57.186  1.00  0.00           C
ATOM    640  HZ  PHE    39      41.476  48.894  56.560  1.00  0.00           H
ATOM    641  CE2 PHE    39      41.880  46.773  56.616  1.00  0.00           C
ATOM    642  HE2 PHE    39      42.059  46.701  55.551  1.00  0.00           H
ATOM    643  CD2 PHE    39      41.982  45.629  57.429  1.00  0.00           C
ATOM    644  HD2 PHE    39      42.239  44.680  56.980  1.00  0.00           H
ATOM    645  C   PHE    39      39.489  43.835  59.190  1.00  0.00           C
ATOM    646  O   PHE    39      38.534  44.611  59.110  1.00  0.00           O
ATOM    647  N   PHE    40      39.609  42.757  58.406  1.00  0.00           N
ATOM    648  H   PHE    40      40.419  42.159  58.535  1.00  0.00           H
ATOM    649  CA  PHE    40      38.622  42.354  57.399  1.00  0.00           C
ATOM    650  HA  PHE    40      38.537  43.147  56.655  1.00  0.00           H
ATOM    651  CB  PHE    40      39.145  41.077  56.711  1.00  0.00           C
ATOM    652  HB2 PHE    40      40.187  41.261  56.447  1.00  0.00           H
ATOM    653  HB3 PHE    40      39.150  40.267  57.441  1.00  0.00           H
ATOM    654  CG  PHE    40      38.448  40.557  55.457  1.00  0.00           C
ATOM    655  CD1 PHE    40      39.228  40.231  54.331  1.00  0.00           C
ATOM    656  HD1 PHE    40      40.283  40.450  54.328  1.00  0.00           H
ATOM    657  CE1 PHE    40      38.661  39.571  53.227  1.00  0.00           C
ATOM    658  HE1 PHE    40      39.282  39.316  52.380  1.00  0.00           H
ATOM    659  CZ  PHE    40      37.294  39.252  53.227  1.00  0.00           C
ATOM    660  HZ  PHE    40      36.855  38.740  52.382  1.00  0.00           H
ATOM    661  CE2 PHE    40      36.490  39.641  54.310  1.00  0.00           C
ATOM    662  HE2 PHE    40      35.432  39.426  54.295  1.00  0.00           H
ATOM    663  CD2 PHE    40      37.068  40.273  55.427  1.00  0.00           C
ATOM    664  HD2 PHE    40      36.446  40.505  56.273  1.00  0.00           H
ATOM    665  C   PHE    40      37.240  42.160  58.043  1.00  0.00           C
ATOM    666  O   PHE    40      36.251  42.663  57.518  1.00  0.00           O
ATOM    667  N   GLU    41      37.161  41.474  59.188  1.00  0.00           N
ATOM    668  H   GLU    41      38.010  41.037  59.540  1.00  0.00           H
ATOM    669  CA  GLU    41      35.935  41.267  59.969  1.00  0.00           C
ATOM    670  HA  GLU    41      35.216  40.719  59.360  1.00  0.00           H
ATOM    671  CB  GLU    41      36.269  40.405  61.202  1.00  0.00           C
ATOM    672  HB2 GLU    41      36.575  39.414  60.863  1.00  0.00           H
ATOM    673  HB3 GLU    41      37.105  40.868  61.714  1.00  0.00           H
ATOM    674  CG  GLU    41      35.134  40.242  62.224  1.00  0.00           C
```

ATOM	675	HG2	GLU	41	34.808	41.224	62.572	1.00	0.00	H
ATOM	676	HG3	GLU	41	34.288	39.757	61.732	1.00	0.00	H
ATOM	677	CD	GLU	41	35.563	39.423	63.452	1.00	0.00	C
ATOM	678	OE1	GLU	41	36.742	39.481	63.883	1.00	0.00	O
ATOM	679	OE2	GLU	41	34.696	38.713	64.021	1.00	0.00	O
ATOM	680	C	GLU	41	35.265	42.588	60.368	1.00	0.00	C
ATOM	681	O	GLU	41	34.087	42.770	60.070	1.00	0.00	O
ATOM	682	N	ILE	42	36.000	43.517	60.994	1.00	0.00	N
ATOM	683	H	ILE	42	36.974	43.301	61.191	1.00	0.00	H
ATOM	684	CA	ILE	42	35.454	44.801	61.469	1.00	0.00	C
ATOM	685	HA	ILE	42	34.597	44.597	62.112	1.00	0.00	H
ATOM	686	CB	ILE	42	36.515	45.567	62.299	1.00	0.00	C
ATOM	687	HB	ILE	42	37.431	45.622	61.707	1.00	0.00	H
ATOM	688	CG2	ILE	42	36.058	47.006	62.613	1.00	0.00	C
ATOM	689	HG21	ILE	42	36.023	47.602	61.700	1.00	0.00	H
ATOM	690	HG22	ILE	42	36.752	47.491	63.296	1.00	0.00	H
ATOM	691	HG23	ILE	42	35.066	46.993	63.065	1.00	0.00	H
ATOM	692	CG1	ILE	42	36.825	44.835	63.627	1.00	0.00	C
ATOM	693	HG12	ILE	42	36.043	45.048	64.357	1.00	0.00	H
ATOM	694	HG13	ILE	42	36.831	43.758	63.466	1.00	0.00	H
ATOM	695	CD1	ILE	42	38.192	45.217	64.216	1.00	0.00	C
ATOM	696	HD11	ILE	42	38.980	45.026	63.487	1.00	0.00	H
ATOM	697	HD12	ILE	42	38.383	44.613	65.103	1.00	0.00	H
ATOM	698	HD13	ILE	42	38.212	46.269	64.496	1.00	0.00	H
ATOM	699	C	ILE	42	34.932	45.642	60.295	1.00	0.00	C
ATOM	700	O	ILE	42	33.814	46.157	60.351	1.00	0.00	O
ATOM	701	N	VAL	43	35.715	45.753	59.216	1.00	0.00	N
ATOM	702	H	VAL	43	36.623	45.296	59.228	1.00	0.00	H
ATOM	703	CA	VAL	43	35.317	46.502	58.013	1.00	0.00	C
ATOM	704	HA	VAL	43	34.989	47.496	58.319	1.00	0.00	H
ATOM	705	CB	VAL	43	36.526	46.672	57.067	1.00	0.00	C
ATOM	706	HB	VAL	43	36.960	45.692	56.861	1.00	0.00	H
ATOM	707	CG1	VAL	43	36.147	47.319	55.730	1.00	0.00	C
ATOM	708	HG11	VAL	43	37.044	47.496	55.135	1.00	0.00	H
ATOM	709	HG12	VAL	43	35.494	46.658	55.165	1.00	0.00	H
ATOM	710	HG13	VAL	43	35.643	48.269	55.906	1.00	0.00	H
ATOM	711	CG2	VAL	43	37.597	47.565	57.711	1.00	0.00	C
ATOM	712	HG21	VAL	43	37.195	48.560	57.902	1.00	0.00	H
ATOM	713	HG22	VAL	43	38.457	47.650	57.045	1.00	0.00	H
ATOM	714	HG23	VAL	43	37.943	47.133	58.649	1.00	0.00	H
ATOM	715	C	VAL	43	34.117	45.841	57.318	1.00	0.00	C
ATOM	716	O	VAL	43	33.213	46.539	56.858	1.00	0.00	O
ATOM	717	N	CYS	44	34.066	44.507	57.264	1.00	0.00	N
ATOM	718	H	CYS	44	34.833	43.985	57.676	1.00	0.00	H
ATOM	719	CA	CYS	44	32.977	43.742	56.648	1.00	0.00	C
ATOM	720	HA	CYS	44	32.776	44.152	55.660	1.00	0.00	H
ATOM	721	CB	CYS	44	33.447	42.285	56.497	1.00	0.00	C
ATOM	722	HB2	CYS	44	34.361	42.263	55.902	1.00	0.00	H
ATOM	723	HB3	CYS	44	33.679	41.883	57.486	1.00	0.00	H
ATOM	724	SG	CYS	44	32.192	41.233	55.711	1.00	0.00	S
ATOM	725	HG	CYS	44	31.938	42.025	54.665	1.00	0.00	H
ATOM	726	C	CYS	44	31.656	43.844	57.418	1.00	0.00	C

ATOM	727	O	CYS	44	30.633	44.173	56.815	1.00	0.00	O
ATOM	728	N	ASP	45	31.681	43.582	58.726	1.00	0.00	N
ATOM	729	H	ASP	45	32.574	43.362	59.161	1.00	0.00	H
ATOM	730	CA	ASP	45	30.488	43.509	59.575	1.00	0.00	C
ATOM	731	HA	ASP	45	29.800	42.775	59.151	1.00	0.00	H
ATOM	732	CB	ASP	45	30.914	43.025	60.973	1.00	0.00	C
ATOM	733	HB2	ASP	45	31.502	42.113	60.862	1.00	0.00	H
ATOM	734	HB3	ASP	45	31.553	43.781	61.433	1.00	0.00	H
ATOM	735	CG	ASP	45	29.746	42.705	61.912	1.00	0.00	C
ATOM	736	OD1	ASP	45	28.794	41.983	61.533	1.00	0.00	O
ATOM	737	OD2	ASP	45	29.761	43.154	63.080	1.00	0.00	O
ATOM	738	C	ASP	45	29.748	44.855	59.631	1.00	0.00	C
ATOM	739	O	ASP	45	28.524	44.886	59.545	1.00	0.00	O
ATOM	740	N	GLU	46	30.470	45.978	59.702	1.00	0.00	N
ATOM	741	H	GLU	46	31.477	45.906	59.797	1.00	0.00	H
ATOM	742	CA	GLU	46	29.855	47.305	59.612	1.00	0.00	C
ATOM	743	HA	GLU	46	28.913	47.288	60.159	1.00	0.00	H
ATOM	744	CB	GLU	46	30.766	48.352	60.273	1.00	0.00	C
ATOM	745	HB2	GLU	46	31.785	48.240	59.897	1.00	0.00	H
ATOM	746	HB3	GLU	46	30.428	49.341	59.984	1.00	0.00	H
ATOM	747	CG	GLU	46	30.780	48.277	61.813	1.00	0.00	C
ATOM	748	HG2	GLU	46	31.337	47.386	62.111	1.00	0.00	H
ATOM	749	HG3	GLU	46	31.325	49.142	62.197	1.00	0.00	H
ATOM	750	CD	GLU	46	29.380	48.236	62.454	1.00	0.00	C
ATOM	751	OE1	GLU	46	28.626	49.237	62.406	1.00	0.00	O
ATOM	752	OE2	GLU	46	29.000	47.187	63.022	1.00	0.00	O
ATOM	753	C	GLU	46	29.457	47.687	58.175	1.00	0.00	C
ATOM	754	O	GLU	46	28.320	48.111	57.967	1.00	0.00	O
ATOM	755	N	THR	47	30.307	47.481	57.158	1.00	0.00	N
ATOM	756	H	THR	47	31.220	47.084	57.349	1.00	0.00	H
ATOM	757	CA	THR	47	29.958	47.871	55.773	1.00	0.00	C
ATOM	758	HA	THR	47	29.739	48.939	55.772	1.00	0.00	H
ATOM	759	CB	THR	47	31.108	47.637	54.781	1.00	0.00	C
ATOM	760	HB	THR	47	31.354	46.575	54.747	1.00	0.00	H
ATOM	761	CG2	THR	47	30.770	48.114	53.368	1.00	0.00	C
ATOM	762	HG21	THR	47	30.357	49.123	53.408	1.00	0.00	H
ATOM	763	HG22	THR	47	30.050	47.439	52.909	1.00	0.00	H
ATOM	764	HG23	THR	47	31.670	48.129	52.763	1.00	0.00	H
ATOM	765	OG1	THR	47	32.255	48.354	55.156	1.00	0.00	O
ATOM	766	HG1	THR	47	32.656	47.859	55.894	1.00	0.00	H
ATOM	767	C	THR	47	28.706	47.151	55.257	1.00	0.00	C
ATOM	768	O	THR	47	27.882	47.775	54.586	1.00	0.00	O
ATOM	769	N	GLU	48	28.528	45.864	55.587	1.00	0.00	N
ATOM	770	H	GLU	48	29.233	45.404	56.161	1.00	0.00	H
ATOM	771	CA	GLU	48	27.337	45.089	55.205	1.00	0.00	C
ATOM	772	HA	GLU	48	27.237	45.130	54.119	1.00	0.00	H
ATOM	773	CB	GLU	48	27.508	43.609	55.617	1.00	0.00	C
ATOM	774	HB2	GLU	48	28.414	43.221	55.148	1.00	0.00	H
ATOM	775	HB3	GLU	48	27.624	43.545	56.700	1.00	0.00	H
ATOM	776	CG	GLU	48	26.318	42.732	55.181	1.00	0.00	C
ATOM	777	HG2	GLU	48	25.422	43.049	55.719	1.00	0.00	H
ATOM	778	HG3	GLU	48	26.143	42.892	54.115	1.00	0.00	H

ATOM	779	CD	GLU	48	26.534	41.230	55.440	1.00	0.00	C
ATOM	780	OE1	GLU	48	26.755	40.821	56.605	1.00	0.00	O
ATOM	781	OE2	GLU	48	26.416	40.421	54.487	1.00	0.00	O
ATOM	782	C	GLU	48	26.050	45.691	55.794	1.00	0.00	C
ATOM	783	O	GLU	48	25.059	45.836	55.073	1.00	0.00	O
ATOM	784	N	LYS	49	26.074	46.091	57.076	1.00	0.00	N
ATOM	785	H	LYS	49	26.943	45.974	57.588	1.00	0.00	H
ATOM	786	CA	LYS	49	24.953	46.769	57.758	1.00	0.00	C
ATOM	787	HA	LYS	49	24.040	46.185	57.635	1.00	0.00	H
ATOM	788	CB	LYS	49	25.264	46.930	59.258	1.00	0.00	C
ATOM	789	HB2	LYS	49	26.189	47.500	59.350	1.00	0.00	H
ATOM	790	HB3	LYS	49	24.469	47.514	59.726	1.00	0.00	H
ATOM	791	CG	LYS	49	25.407	45.609	60.032	1.00	0.00	C
ATOM	792	HG2	LYS	49	24.424	45.164	60.193	1.00	0.00	H
ATOM	793	HG3	LYS	49	26.005	44.909	59.451	1.00	0.00	H
ATOM	794	CD	LYS	49	26.094	45.864	61.384	1.00	0.00	C
ATOM	795	HD2	LYS	49	26.934	46.543	61.229	1.00	0.00	H
ATOM	796	HD3	LYS	49	25.394	46.333	62.078	1.00	0.00	H
ATOM	797	CE	LYS	49	26.646	44.567	61.982	1.00	0.00	C
ATOM	798	HE2	LYS	49	25.828	43.937	62.342	1.00	0.00	H
ATOM	799	HE3	LYS	49	27.171	44.022	61.191	1.00	0.00	H
ATOM	800	NZ	LYS	49	27.606	44.856	63.069	1.00	0.00	N
ATOM	801	HZ1	LYS	49	28.173	44.040	63.292	1.00	0.00	H
ATOM	802	HZ2	LYS	49	27.166	45.211	63.909	1.00	0.00	H
ATOM	803	HZ3	LYS	49	28.270	45.579	62.787	1.00	0.00	H
ATOM	804	C	LYS	49	24.684	48.149	57.158	1.00	0.00	C
ATOM	805	O	LYS	49	23.534	48.544	56.993	1.00	0.00	O
ATOM	806	N	HIE	50	25.735	48.894	56.813	1.00	0.00	N
ATOM	807	H	HIE	50	26.658	48.513	57.009	1.00	0.00	H
ATOM	808	CA	HIE	50	25.656	50.276	56.316	1.00	0.00	C
ATOM	809	HA	HIE	50	24.840	50.775	56.839	1.00	0.00	H
ATOM	810	CB	HIE	50	26.947	51.033	56.665	1.00	0.00	C
ATOM	811	HB2	HIE	50	27.765	50.607	56.082	1.00	0.00	H
ATOM	812	HB3	HIE	50	26.836	52.077	56.368	1.00	0.00	H
ATOM	813	CG	HIE	50	27.364	51.021	58.119	1.00	0.00	C
ATOM	814	ND1	HIE	50	28.614	51.451	58.552	1.00	0.00	N
ATOM	815	CE1	HIE	50	28.663	51.199	59.864	1.00	0.00	C
ATOM	816	HE1	HIE	50	29.534	51.341	60.489	1.00	0.00	H
ATOM	817	NE2	HIE	50	27.524	50.634	60.277	1.00	0.00	N
ATOM	818	HE2	HIE	50	27.447	50.166	61.181	1.00	0.00	H
ATOM	819	CD2	HIE	50	26.674	50.538	59.198	1.00	0.00	C
ATOM	820	HD2	HIE	50	25.695	50.083	59.188	1.00	0.00	H
ATOM	821	C	HIE	50	25.305	50.360	54.819	1.00	0.00	C
ATOM	822	O	HIE	50	25.749	51.274	54.123	1.00	0.00	O
ATOM	823	N	SER	51	24.518	49.408	54.304	1.00	0.00	N
ATOM	824	H	SER	51	24.170	48.718	54.961	1.00	0.00	H
ATOM	825	CA	SER	51	24.072	49.261	52.902	1.00	0.00	C
ATOM	826	HA	SER	51	23.474	48.350	52.873	1.00	0.00	H
ATOM	827	CB	SER	51	23.127	50.410	52.512	1.00	0.00	C
ATOM	828	HB2	SER	51	22.607	50.152	51.587	1.00	0.00	H
ATOM	829	HB3	SER	51	22.384	50.550	53.299	1.00	0.00	H
ATOM	830	OG	SER	51	23.846	51.616	52.316	1.00	0.00	O

ATOM	831	HG	SER	51	24.516	51.649	53.029	1.00	0.00	H
ATOM	832	C	SER	51	25.175	49.048	51.848	1.00	0.00	C
ATOM	833	O	SER	51	24.872	49.012	50.653	1.00	0.00	O
ATOM	834	N	GLY	52	26.438	48.907	52.254	1.00	0.00	N
ATOM	835	H	GLY	52	26.624	48.824	53.246	1.00	0.00	H
ATOM	836	CA	GLY	52	27.570	48.717	51.348	1.00	0.00	C
ATOM	837	HA2	GLY	52	27.398	49.270	50.423	1.00	0.00	H
ATOM	838	HA3	GLY	52	28.469	49.123	51.809	1.00	0.00	H
ATOM	839	C	GLY	52	27.828	47.252	51.000	1.00	0.00	C
ATOM	840	O	GLY	52	27.218	46.333	51.559	1.00	0.00	O
ATOM	841	N	LYS	53	28.775	47.033	50.083	1.00	0.00	N
ATOM	842	H	LYS	53	29.157	47.852	49.612	1.00	0.00	H
ATOM	843	CA	LYS	53	29.222	45.705	49.639	1.00	0.00	C
ATOM	844	HA	LYS	53	28.978	44.971	50.409	1.00	0.00	H
ATOM	845	CB	LYS	53	28.439	45.339	48.363	1.00	0.00	C
ATOM	846	HB2	LYS	53	27.371	45.417	48.578	1.00	0.00	H
ATOM	847	HB3	LYS	53	28.678	46.058	47.578	1.00	0.00	H
ATOM	848	CG	LYS	53	28.728	43.918	47.854	1.00	0.00	C
ATOM	849	HG2	LYS	53	29.787	43.824	47.610	1.00	0.00	H
ATOM	850	HG3	LYS	53	28.478	43.199	48.635	1.00	0.00	H
ATOM	851	CD	LYS	53	27.897	43.618	46.598	1.00	0.00	C
ATOM	852	HD2	LYS	53	26.835	43.732	46.831	1.00	0.00	H
ATOM	853	HD3	LYS	53	28.165	44.328	45.814	1.00	0.00	H
ATOM	854	CE	LYS	53	28.157	42.188	46.109	1.00	0.00	C
ATOM	855	HE2	LYS	53	29.229	42.062	45.934	1.00	0.00	H
ATOM	856	HE3	LYS	53	27.858	41.489	46.896	1.00	0.00	H
ATOM	857	NZ	LYS	53	27.402	41.892	44.868	1.00	0.00	N
ATOM	858	HZ1	LYS	53	26.395	42.005	45.004	1.00	0.00	H
ATOM	859	HZ2	LYS	53	27.538	40.924	44.580	1.00	0.00	H
ATOM	860	HZ3	LYS	53	27.668	42.493	44.106	1.00	0.00	H
ATOM	861	C	LYS	53	30.736	45.698	49.426	1.00	0.00	C
ATOM	862	O	LYS	53	31.233	46.379	48.529	1.00	0.00	O
ATOM	863	N	LEU	54	31.472	44.926	50.236	1.00	0.00	N
ATOM	864	H	LEU	54	31.002	44.393	50.948	1.00	0.00	H
ATOM	865	CA	LEU	54	32.902	44.675	49.996	1.00	0.00	C
ATOM	866	HA	LEU	54	33.419	45.627	49.888	1.00	0.00	H
ATOM	867	CB	LEU	54	33.570	43.912	51.155	1.00	0.00	C
ATOM	868	HB2	LEU	54	33.141	42.910	51.210	1.00	0.00	H
ATOM	869	HB3	LEU	54	34.621	43.805	50.880	1.00	0.00	H
ATOM	870	CG	LEU	54	33.508	44.537	52.564	1.00	0.00	C
ATOM	871	HG	LEU	54	32.513	44.386	52.984	1.00	0.00	H
ATOM	872	CD1	LEU	54	34.542	43.856	53.460	1.00	0.00	C
ATOM	873	HD11	LEU	54	34.287	42.807	53.589	1.00	0.00	H
ATOM	874	HD12	LEU	54	34.573	44.341	54.434	1.00	0.00	H
ATOM	875	HD13	LEU	54	35.531	43.959	53.019	1.00	0.00	H
ATOM	876	CD2	LEU	54	33.826	46.025	52.586	1.00	0.00	C
ATOM	877	HD21	LEU	54	34.801	46.218	52.135	1.00	0.00	H
ATOM	878	HD22	LEU	54	33.050	46.554	52.050	1.00	0.00	H
ATOM	879	HD23	LEU	54	33.821	46.395	53.611	1.00	0.00	H
ATOM	880	C	LEU	54	33.072	43.914	48.674	1.00	0.00	C
ATOM	881	O	LEU	54	32.399	42.902	48.456	1.00	0.00	O
ATOM	882	N	GLY	55	33.942	44.415	47.797	1.00	0.00	N

ATOM	883	H	GLY	55	34.501	45.213	48.081	1.00	0.00	H
ATOM	884	CA	GLY	55	34.090	43.920	46.426	1.00	0.00	C
ATOM	885	HA2	GLY	55	33.306	43.199	46.189	1.00	0.00	H
ATOM	886	HA3	GLY	55	33.984	44.763	45.743	1.00	0.00	H
ATOM	887	C	GLY	55	35.438	43.263	46.143	1.00	0.00	C
ATOM	888	O	GLY	55	35.485	42.265	45.430	1.00	0.00	O
ATOM	889	N	THR	56	36.533	43.800	46.690	1.00	0.00	N
ATOM	890	H	THR	56	36.438	44.581	47.331	1.00	0.00	H
ATOM	891	CA	THR	56	37.897	43.303	46.437	1.00	0.00	C
ATOM	892	HA	THR	56	37.850	42.223	46.297	1.00	0.00	H
ATOM	893	CB	THR	56	38.515	43.905	45.157	1.00	0.00	C
ATOM	894	HB	THR	56	39.512	43.482	45.042	1.00	0.00	H
ATOM	895	CG2	THR	56	37.758	43.638	43.856	1.00	0.00	C
ATOM	896	HG21	THR	56	38.340	44.016	43.016	1.00	0.00	H
ATOM	897	HG22	THR	56	36.790	44.134	43.868	1.00	0.00	H
ATOM	898	HG23	THR	56	37.617	42.564	43.730	1.00	0.00	H
ATOM	899	OG1	THR	56	38.643	45.297	45.267	1.00	0.00	O
ATOM	900	HG1	THR	56	37.762	45.687	45.295	1.00	0.00	H
ATOM	901	C	THR	56	38.833	43.556	47.624	1.00	0.00	C
ATOM	902	O	THR	56	38.551	44.379	48.498	1.00	0.00	O
ATOM	903	N	TYR	57	39.955	42.832	47.639	1.00	0.00	N
ATOM	904	H	TYR	57	40.159	42.241	46.847	1.00	0.00	H
ATOM	905	CA	TYR	57	40.977	42.849	48.688	1.00	0.00	C
ATOM	906	HA	TYR	57	40.895	43.776	49.256	1.00	0.00	H
ATOM	907	CB	TYR	57	40.773	41.666	49.645	1.00	0.00	C
ATOM	908	HB2	TYR	57	41.030	40.741	49.127	1.00	0.00	H
ATOM	909	HB3	TYR	57	41.459	41.773	50.487	1.00	0.00	H
ATOM	910	CG	TYR	57	39.358	41.551	50.172	1.00	0.00	C
ATOM	911	CD1	TYR	57	38.421	40.735	49.508	1.00	0.00	C
ATOM	912	HD1	TYR	57	38.720	40.168	48.638	1.00	0.00	H
ATOM	913	CE1	TYR	57	37.089	40.683	49.960	1.00	0.00	C
ATOM	914	HE1	TYR	57	36.359	40.069	49.457	1.00	0.00	H
ATOM	915	CZ	TYR	57	36.705	41.431	51.090	1.00	0.00	C
ATOM	916	OH	TYR	57	35.443	41.309	51.575	1.00	0.00	O
ATOM	917	HH	TYR	57	35.416	41.630	52.479	1.00	0.00	H
ATOM	918	CE2	TYR	57	37.649	42.243	51.760	1.00	0.00	C
ATOM	919	HE2	TYR	57	37.374	42.791	52.646	1.00	0.00	H
ATOM	920	CD2	TYR	57	38.971	42.309	51.292	1.00	0.00	C
ATOM	921	HD2	TYR	57	39.696	42.937	51.795	1.00	0.00	H
ATOM	922	C	TYR	57	42.366	42.788	48.049	1.00	0.00	C
ATOM	923	O	TYR	57	42.529	42.189	46.986	1.00	0.00	O
ATOM	924	N	LEU	58	43.363	43.400	48.686	1.00	0.00	N
ATOM	925	H	LEU	58	43.145	43.870	49.563	1.00	0.00	H
ATOM	926	CA	LEU	58	44.710	43.569	48.141	1.00	0.00	C
ATOM	927	HA	LEU	58	44.944	42.714	47.504	1.00	0.00	H
ATOM	928	CB	LEU	58	44.719	44.847	47.269	1.00	0.00	C
ATOM	929	HB2	LEU	58	43.869	44.824	46.588	1.00	0.00	H
ATOM	930	HB3	LEU	58	44.595	45.712	47.923	1.00	0.00	H
ATOM	931	CG	LEU	58	46.015	45.000	46.453	1.00	0.00	C
ATOM	932	HG	LEU	58	46.830	44.606	47.041	1.00	0.00	H
ATOM	933	CD1	LEU	58	45.971	44.215	45.139	1.00	0.00	C
ATOM	934	HD11	LEU	58	45.624	43.199	45.323	1.00	0.00	H

ATOM	935	HD12	LEU	58	46.968	44.184	44.701	1.00	0.00	H
ATOM	936	HD13	LEU	58	45.298	44.670	44.424	1.00	0.00	H
ATOM	937	CD2	LEU	58	46.352	46.465	46.169	1.00	0.00	C
ATOM	938	HD21	LEU	58	45.533	46.957	45.654	1.00	0.00	H
ATOM	939	HD22	LEU	58	46.524	46.984	47.113	1.00	0.00	H
ATOM	940	HD23	LEU	58	47.248	46.526	45.553	1.00	0.00	H
ATOM	941	C	LEU	58	45.736	43.595	49.286	1.00	0.00	C
ATOM	942	O	LEU	58	46.184	44.654	49.725	1.00	0.00	O
ATOM	943	N	GLY	59	46.085	42.420	49.811	1.00	0.00	N
ATOM	944	H	GLY	59	45.649	41.584	49.453	1.00	0.00	H
ATOM	945	CA	GLY	59	46.894	42.315	51.028	1.00	0.00	C
ATOM	946	HA2	GLY	59	47.160	41.276	51.210	1.00	0.00	H
ATOM	947	HA3	GLY	59	47.807	42.894	50.907	1.00	0.00	H
ATOM	948	C	GLY	59	46.143	42.843	52.245	1.00	0.00	C
ATOM	949	O	GLY	59	45.031	42.402	52.515	1.00	0.00	O
ATOM	950	N	ASP	60	46.727	43.814	52.951	1.00	0.00	N
ATOM	951	H	ASP	60	47.630	44.162	52.671	1.00	0.00	H
ATOM	952	CA	ASP	60	46.039	44.539	54.028	1.00	0.00	C
ATOM	953	HA	ASP	60	45.507	43.811	54.641	1.00	0.00	H
ATOM	954	CB	ASP	60	47.063	45.251	54.925	1.00	0.00	C
ATOM	955	HB2	ASP	60	46.581	45.463	55.880	1.00	0.00	H
ATOM	956	HB3	ASP	60	47.899	44.580	55.127	1.00	0.00	H
ATOM	957	CG	ASP	60	47.600	46.567	54.352	1.00	0.00	C
ATOM	958	OD1	ASP	60	47.907	46.678	53.126	1.00	0.00	O
ATOM	959	OD2	ASP	60	47.766	47.528	55.150	1.00	0.00	O
ATOM	960	C	ASP	60	44.987	45.550	53.528	1.00	0.00	C
ATOM	961	O	ASP	60	44.305	46.164	54.345	1.00	0.00	O
ATOM	962	N	GLY	61	44.880	45.763	52.211	1.00	0.00	N
ATOM	963	H	GLY	61	45.477	45.235	51.590	1.00	0.00	H
ATOM	964	CA	GLY	61	43.926	46.687	51.607	1.00	0.00	C
ATOM	965	HA2	GLY	61	43.818	47.545	52.263	1.00	0.00	H
ATOM	966	HA3	GLY	61	44.318	47.044	50.656	1.00	0.00	H
ATOM	967	C	GLY	61	42.551	46.059	51.355	1.00	0.00	C
ATOM	968	O	GLY	61	42.460	44.913	50.909	1.00	0.00	O
ATOM	969	N	ALA	62	41.481	46.827	51.560	1.00	0.00	N
ATOM	970	H	ALA	62	41.634	47.754	51.950	1.00	0.00	H
ATOM	971	CA	ALA	62	40.102	46.441	51.256	1.00	0.00	C
ATOM	972	HA	ALA	62	40.118	45.566	50.604	1.00	0.00	H
ATOM	973	CB	ALA	62	39.395	46.029	52.553	1.00	0.00	C
ATOM	974	HB1	ALA	62	39.941	45.213	53.029	1.00	0.00	H
ATOM	975	HB2	ALA	62	38.379	45.700	52.333	1.00	0.00	H
ATOM	976	HB3	ALA	62	39.356	46.875	53.240	1.00	0.00	H
ATOM	977	C	ALA	62	39.348	47.549	50.498	1.00	0.00	C
ATOM	978	O	ALA	62	39.549	48.743	50.736	1.00	0.00	O
ATOM	979	N	MET	63	38.462	47.136	49.586	1.00	0.00	N
ATOM	980	H	MET	63	38.365	46.137	49.432	1.00	0.00	H
ATOM	981	CA	MET	63	37.668	48.013	48.720	1.00	0.00	C
ATOM	982	HA	MET	63	37.696	49.026	49.124	1.00	0.00	H
ATOM	983	CB	MET	63	38.314	48.059	47.322	1.00	0.00	C
ATOM	984	HB2	MET	63	39.201	48.688	47.392	1.00	0.00	H
ATOM	985	HB3	MET	63	38.650	47.060	47.056	1.00	0.00	H
ATOM	986	CG	MET	63	37.431	48.597	46.182	1.00	0.00	C

ATOM 987 HG2 MET 63 36.897 49.482 46.526 1.00 0.00 H
ATOM 988 HG3 MET 63 38.092 48.912 45.375 1.00 0.00 H
ATOM 989 SD MET 63 36.240 47.410 45.476 1.00 0.00 S
ATOM 990 CE MET 63 35.485 48.456 44.201 1.00 0.00 C
ATOM 991 HE1 MET 63 34.686 47.907 43.703 1.00 0.00 H
ATOM 992 HE2 MET 63 35.074 49.356 44.661 1.00 0.00 H
ATOM 993 HE3 MET 63 36.237 48.735 43.463 1.00 0.00 H
ATOM 994 C MET 63 36.195 47.586 48.701 1.00 0.00 C
ATOM 995 O MET 63 35.859 46.396 48.717 1.00 0.00 O
ATOM 996 N CYS 64 35.303 48.574 48.639 1.00 0.00 N
ATOM 997 H CYS 64 35.640 49.524 48.561 1.00 0.00 H
ATOM 998 CA CYS 64 33.856 48.377 48.638 1.00 0.00 C
ATOM 999 HA CYS 64 33.645 47.405 48.193 1.00 0.00 H
ATOM 1000 CB CYS 64 33.344 48.352 50.092 1.00 0.00 C
ATOM 1001 HB2 CYS 64 32.577 47.590 50.176 1.00 0.00 H
ATOM 1002 HB3 CYS 64 34.163 48.097 50.763 1.00 0.00 H
ATOM 1003 SG CYS 64 32.582 49.904 50.628 1.00 0.00 S
ATOM 1004 HG CYS 64 32.416 49.608 51.924 1.00 0.00 H
ATOM 1005 C CYS 64 33.124 49.408 47.775 1.00 0.00 C
ATOM 1006 O CYS 64 33.697 50.428 47.377 1.00 0.00 O
ATOM 1007 N ILE 65 31.840 49.134 47.532 1.00 0.00 N
ATOM 1008 H ILE 65 31.455 48.278 47.916 1.00 0.00 H
ATOM 1009 CA ILE 65 30.902 50.063 46.901 1.00 0.00 C
ATOM 1010 HA ILE 65 31.414 51.016 46.770 1.00 0.00 H
ATOM 1011 CB ILE 65 30.489 49.586 45.484 1.00 0.00 C
ATOM 1012 HB ILE 65 30.031 50.444 44.988 1.00 0.00 H
ATOM 1013 CG2 ILE 65 31.724 49.184 44.658 1.00 0.00 C
ATOM 1014 HG21 ILE 65 32.523 49.907 44.808 1.00 0.00 H
ATOM 1015 HG22 ILE 65 31.465 49.167 43.603 1.00 0.00 H
ATOM 1016 HG23 ILE 65 32.083 48.199 44.961 1.00 0.00 H
ATOM 1017 CG1 ILE 65 29.439 48.447 45.490 1.00 0.00 C
ATOM 1018 HG12 ILE 65 29.848 47.582 46.015 1.00 0.00 H
ATOM 1019 HG13 ILE 65 28.549 48.780 46.023 1.00 0.00 H
ATOM 1020 CD1 ILE 65 28.977 48.005 44.094 1.00 0.00 C
ATOM 1021 HD11 ILE 65 28.624 48.869 43.529 1.00 0.00 H
ATOM 1022 HD12 ILE 65 28.159 47.292 44.196 1.00 0.00 H
ATOM 1023 HD13 ILE 65 29.792 47.523 43.555 1.00 0.00 H
ATOM 1024 C ILE 65 29.687 50.336 47.800 1.00 0.00 C
ATOM 1025 O ILE 65 29.189 49.444 48.492 1.00 0.00 O
ATOM 1026 N TRP 66 29.162 51.554 47.686 1.00 0.00 N
ATOM 1027 H TRP 66 29.704 52.225 47.151 1.00 0.00 H
ATOM 1028 CA TRP 66 27.759 51.912 47.908 1.00 0.00 C
ATOM 1029 HA TRP 66 27.185 51.020 48.165 1.00 0.00 H
ATOM 1030 CB TRP 66 27.617 52.935 49.048 1.00 0.00 C
ATOM 1031 HB2 TRP 66 28.099 53.861 48.742 1.00 0.00 H
ATOM 1032 HB3 TRP 66 26.558 53.162 49.176 1.00 0.00 H
ATOM 1033 CG TRP 66 28.165 52.558 50.389 1.00 0.00 C
ATOM 1034 CD1 TRP 66 27.436 52.078 51.420 1.00 0.00 C
ATOM 1035 HD1 TRP 66 26.369 51.892 51.392 1.00 0.00 H
ATOM 1036 NE1 TRP 66 28.255 51.855 52.508 1.00 0.00 N
ATOM 1037 HE1 TRP 66 27.901 51.517 53.397 1.00 0.00 H
ATOM 1038 CE2 TRP 66 29.566 52.178 52.230 1.00 0.00 C

ATOM	1039	CZ2	TRP	66	30.746	52.141	52.986	1.00	0.00	C
ATOM	1040	HZ2	TRP	66	30.736	51.749	53.992	1.00	0.00	H
ATOM	1041	CH2	TRP	66	31.938	52.628	52.423	1.00	0.00	C
ATOM	1042	HH2	TRP	66	32.850	52.623	53.006	1.00	0.00	H
ATOM	1043	CZ3	TRP	66	31.947	53.092	51.095	1.00	0.00	C
ATOM	1044	HZ3	TRP	66	32.871	53.432	50.647	1.00	0.00	H
ATOM	1045	CE3	TRP	66	30.763	53.089	50.332	1.00	0.00	C
ATOM	1046	HE3	TRP	66	30.784	53.428	49.308	1.00	0.00	H
ATOM	1047	CD2	TRP	66	29.539	52.646	50.881	1.00	0.00	C
ATOM	1048	C	TRP	66	27.188	52.505	46.610	1.00	0.00	C
ATOM	1049	O	TRP	66	27.939	52.867	45.704	1.00	0.00	O
ATOM	1050	N	GLU	67	25.871	52.686	46.526	1.00	0.00	N
ATOM	1051	H	GLU	67	25.285	52.384	47.288	1.00	0.00	H
ATOM	1052	CA	GLU	67	25.270	53.520	45.473	1.00	0.00	C
ATOM	1053	HA	GLU	67	25.613	53.157	44.501	1.00	0.00	H
ATOM	1054	CB	GLU	67	23.730	53.423	45.507	1.00	0.00	C
ATOM	1055	HB2	GLU	67	23.368	54.110	46.274	1.00	0.00	H
ATOM	1056	HB3	GLU	67	23.344	53.778	44.551	1.00	0.00	H
ATOM	1057	CG	GLU	67	23.107	52.044	45.815	1.00	0.00	C
ATOM	1058	HG2	GLU	67	23.374	51.762	46.836	1.00	0.00	H
ATOM	1059	HG3	GLU	67	22.022	52.161	45.796	1.00	0.00	H
ATOM	1060	CD	GLU	67	23.482	50.887	44.872	1.00	0.00	C
ATOM	1061	OE1	GLU	67	24.258	51.067	43.909	1.00	0.00	O
ATOM	1062	OE2	GLU	67	22.975	49.758	45.098	1.00	0.00	O
ATOM	1063	C	GLU	67	25.718	54.995	45.635	1.00	0.00	C
ATOM	1064	O	GLU	67	25.997	55.440	46.755	1.00	0.00	O
ATOM	1065	N	ALA	68	25.767	55.797	44.562	1.00	0.00	N
ATOM	1066	H	ALA	68	25.605	55.414	43.635	1.00	0.00	H
ATOM	1067	CA	ALA	68	26.147	57.217	44.676	1.00	0.00	C
ATOM	1068	HA	ALA	68	27.134	57.276	45.137	1.00	0.00	H
ATOM	1069	CB	ALA	68	26.250	57.855	43.293	1.00	0.00	C
ATOM	1070	HB1	ALA	68	26.379	58.934	43.396	1.00	0.00	H
ATOM	1071	HB2	ALA	68	25.346	57.655	42.717	1.00	0.00	H
ATOM	1072	HB3	ALA	68	27.127	57.463	42.787	1.00	0.00	H
ATOM	1073	C	ALA	68	25.198	58.039	45.567	1.00	0.00	C
ATOM	1074	O	ALA	68	25.652	58.930	46.274	1.00	0.00	O
ATOM	1075	N	SER	69	23.901	57.717	45.618	1.00	0.00	N
ATOM	1076	H	SER	69	23.535	57.038	44.967	1.00	0.00	H
ATOM	1077	CA	SER	69	22.950	58.377	46.532	1.00	0.00	C
ATOM	1078	HA	SER	69	22.978	59.450	46.344	1.00	0.00	H
ATOM	1079	CB	SER	69	21.524	57.886	46.258	1.00	0.00	C
ATOM	1080	HB2	SER	69	21.446	56.822	46.490	1.00	0.00	H
ATOM	1081	HB3	SER	69	20.829	58.437	46.893	1.00	0.00	H
ATOM	1082	OG	SER	69	21.181	58.096	44.905	1.00	0.00	O
ATOM	1083	HG	SER	69	20.208	58.221	44.870	1.00	0.00	H
ATOM	1084	C	SER	69	23.266	58.162	48.023	1.00	0.00	C
ATOM	1085	O	SER	69	22.678	58.827	48.879	1.00	0.00	O
ATOM	1086	N	HID	70	24.171	57.233	48.355	1.00	0.00	N
ATOM	1087	H	HID	70	24.648	56.750	47.601	1.00	0.00	H
ATOM	1088	CA	HID	70	24.516	56.811	49.715	1.00	0.00	C
ATOM	1089	HA	HID	70	23.839	57.313	50.406	1.00	0.00	H
ATOM	1090	CB	HID	70	24.246	55.295	49.866	1.00	0.00	C

```
ATOM   1091  HB2 HID    70      24.391  54.797  48.907  1.00  0.00           H
ATOM   1092  HB3 HID    70      24.962  54.853  50.554  1.00  0.00           H
ATOM   1093  CG  HID    70      22.862  54.960  50.386  1.00  0.00           C
ATOM   1094  ND1 HID    70      22.504  53.792  51.024  1.00  0.00           N
ATOM   1095  HD1 HID    70      23.114  53.010  51.257  1.00  0.00           H
ATOM   1096  CE1 HID    70      21.226  53.897  51.419  1.00  0.00           C
ATOM   1097  HE1 HID    70      20.688  53.141  51.979  1.00  0.00           H
ATOM   1098  NE2 HID    70      20.718  55.084  51.054  1.00  0.00           N
ATOM   1099  CD2 HID    70      21.742  55.753  50.374  1.00  0.00           C
ATOM   1100  HD2 HID    70      21.666  56.746  49.958  1.00  0.00           H
ATOM   1101  C   HID    70      25.903  57.296  50.191  1.00  0.00           C
ATOM   1102  O   HID    70      26.496  56.693  51.087  1.00  0.00           O
ATOM   1103  N   VAL    71      26.381  58.454  49.707  1.00  0.00           N
ATOM   1104  H   VAL    71      25.933  58.857  48.891  1.00  0.00           H
ATOM   1105  CA  VAL    71      27.591  59.123  50.248  1.00  0.00           C
ATOM   1106  HA  VAL    71      28.449  58.497  50.013  1.00  0.00           H
ATOM   1107  CB  VAL    71      27.827  60.518  49.626  1.00  0.00           C
ATOM   1108  HB  VAL    71      27.135  61.228  50.083  1.00  0.00           H
ATOM   1109  CG1 VAL    71      29.253  60.999  49.901  1.00  0.00           C
ATOM   1110 HG11 VAL    71      29.454  61.032  50.970  1.00  0.00           H
ATOM   1111 HG12 VAL    71      29.975  60.339  49.418  1.00  0.00           H
ATOM   1112 HG13 VAL    71      29.365  62.007  49.508  1.00  0.00           H
ATOM   1113  CG2 VAL    71      27.604  60.581  48.115  1.00  0.00           C
ATOM   1114 HG21 VAL    71      27.968  61.527  47.715  1.00  0.00           H
ATOM   1115 HG22 VAL    71      26.538  60.532  47.907  1.00  0.00           H
ATOM   1116 HG23 VAL    71      28.120  59.757  47.618  1.00  0.00           H
ATOM   1117  C   VAL    71      27.528  59.283  51.777  1.00  0.00           C
ATOM   1118  O   VAL    71      28.512  59.050  52.475  1.00  0.00           O
ATOM   1119  N   ARG    72      26.339  59.593  52.317  1.00  0.00           N
ATOM   1120  H   ARG    72      25.578  59.760  51.675  1.00  0.00           H
ATOM   1121  CA  ARG    72      26.075  59.669  53.767  1.00  0.00           C
ATOM   1122  HA  ARG    72      26.757  60.403  54.200  1.00  0.00           H
ATOM   1123  CB  ARG    72      24.622  60.144  54.011  1.00  0.00           C
ATOM   1124  HB2 ARG    72      24.517  60.420  55.060  1.00  0.00           H
ATOM   1125  HB3 ARG    72      24.421  61.026  53.400  1.00  0.00           H
ATOM   1126  CG  ARG    72      23.595  59.048  53.691  1.00  0.00           C
ATOM   1127  HG2 ARG    72      23.831  58.620  52.716  1.00  0.00           H
ATOM   1128  HG3 ARG    72      23.685  58.280  54.459  1.00  0.00           H
ATOM   1129  CD  ARG    72      22.125  59.456  53.669  1.00  0.00           C
ATOM   1130  HD2 ARG    72      21.834  59.818  54.657  1.00  0.00           H
ATOM   1131  HD3 ARG    72      21.982  60.248  52.931  1.00  0.00           H
ATOM   1132  NE  ARG    72      21.312  58.281  53.299  1.00  0.00           N
ATOM   1133  HE  ARG    72      21.806  57.402  53.163  1.00  0.00           H
ATOM   1134  CZ  ARG    72      20.013  58.266  53.068  1.00  0.00           C
ATOM   1135  NH1 ARG    72      19.268  59.310  53.293  1.00  0.00           N
ATOM   1136 HH11 ARG    72      19.691  60.157  53.612  1.00  0.00           H
ATOM   1137 HH12 ARG    72      18.283  59.292  53.085  1.00  0.00           H
ATOM   1138  NH2 ARG    72      19.431  57.206  52.595  1.00  0.00           N
ATOM   1139 HH21 ARG    72      19.985  56.416  52.276  1.00  0.00           H
ATOM   1140 HH22 ARG    72      18.491  57.290  52.242  1.00  0.00           H
ATOM   1141  C   ARG    72      26.379  58.353  54.498  1.00  0.00           C
ATOM   1142  O   ARG    72      26.958  58.377  55.577  1.00  0.00           O
```

```
ATOM   1143  N   ASN    73      26.014  57.212  53.907  1.00  0.00           N
ATOM   1144  H   ASN    73      25.605  57.259  52.986  1.00  0.00           H
ATOM   1145  CA  ASN    73      26.229  55.884  54.475  1.00  0.00           C
ATOM   1146  HA  ASN    73      25.965  55.905  55.534  1.00  0.00           H
ATOM   1147  CB  ASN    73      25.321  54.852  53.778  1.00  0.00           C
ATOM   1148  HB2 ASN    73      25.568  54.795  52.720  1.00  0.00           H
ATOM   1149  HB3 ASN    73      25.518  53.873  54.213  1.00  0.00           H
ATOM   1150  CG  ASN    73      23.836  55.158  53.915  1.00  0.00           C
ATOM   1151  OD1 ASN    73      23.305  56.085  53.318  1.00  0.00           O
ATOM   1152  ND2 ASN    73      23.128  54.417  54.730  1.00  0.00           N
ATOM   1153 HD21 ASN    73      23.519  53.617  55.212  1.00  0.00           H
ATOM   1154 HD22 ASN    73      22.151  54.632  54.885  1.00  0.00           H
ATOM   1155  C   ASN    73      27.710  55.502  54.387  1.00  0.00           C
ATOM   1156  O   ASN    73      28.247  54.960  55.345  1.00  0.00           O
ATOM   1157  N   SER    74      28.385  55.870  53.293  1.00  0.00           N
ATOM   1158  H   SER    74      27.861  56.285  52.531  1.00  0.00           H
ATOM   1159  CA  SER    74      29.836  55.716  53.136  1.00  0.00           C
ATOM   1160  HA  SER    74      30.086  54.664  53.245  1.00  0.00           H
ATOM   1161  CB  SER    74      30.226  56.165  51.726  1.00  0.00           C
ATOM   1162  HB2 SER    74      29.648  55.604  50.991  1.00  0.00           H
ATOM   1163  HB3 SER    74      30.007  57.225  51.604  1.00  0.00           H
ATOM   1164  OG  SER    74      31.598  55.958  51.500  1.00  0.00           O
ATOM   1165  HG  SER    74      31.747  55.001  51.489  1.00  0.00           H
ATOM   1166  C   SER    74      30.630  56.487  54.200  1.00  0.00           C
ATOM   1167  O   SER    74      31.481  55.914  54.881  1.00  0.00           O
ATOM   1168  N   ILE    75      30.294  57.764  54.413  1.00  0.00           N
ATOM   1169  H   ILE    75      29.603  58.182  53.799  1.00  0.00           H
ATOM   1170  CA  ILE    75      30.925  58.621  55.428  1.00  0.00           C
ATOM   1171  HA  ILE    75      32.007  58.538  55.319  1.00  0.00           H
ATOM   1172  CB  ILE    75      30.537  60.096  55.170  1.00  0.00           C
ATOM   1173  HB  ILE    75      29.468  60.143  54.951  1.00  0.00           H
ATOM   1174  CG2 ILE    75      30.799  60.994  56.393  1.00  0.00           C
ATOM   1175 HG21 ILE    75      30.157  60.704  57.225  1.00  0.00           H
ATOM   1176 HG22 ILE    75      30.574  62.033  56.161  1.00  0.00           H
ATOM   1177 HG23 ILE    75      31.841  60.906  56.702  1.00  0.00           H
ATOM   1178  CG1 ILE    75      31.325  60.592  53.932  1.00  0.00           C
ATOM   1179 HG12 ILE    75      32.389  60.631  54.170  1.00  0.00           H
ATOM   1180 HG13 ILE    75      31.194  59.881  53.115  1.00  0.00           H
ATOM   1181  CD1 ILE    75      30.888  61.962  53.407  1.00  0.00           C
ATOM   1182 HD11 ILE    75      29.810  61.970  53.258  1.00  0.00           H
ATOM   1183 HD12 ILE    75      31.381  62.155  52.454  1.00  0.00           H
ATOM   1184 HD13 ILE    75      31.171  62.747  54.106  1.00  0.00           H
ATOM   1185  C   ILE    75      30.612  58.141  56.854  1.00  0.00           C
ATOM   1186  O   ILE    75      31.538  58.021  57.659  1.00  0.00           O
ATOM   1187  N   ASN    76      29.353  57.789  57.159  1.00  0.00           N
ATOM   1188  H   ASN    76      28.619  57.952  56.479  1.00  0.00           H
ATOM   1189  CA  ASN    76      28.998  57.137  58.427  1.00  0.00           C
ATOM   1190  HA  ASN    76      29.219  57.811  59.258  1.00  0.00           H
ATOM   1191  CB  ASN    76      27.500  56.757  58.450  1.00  0.00           C
ATOM   1192  HB2 ASN    76      27.165  56.504  57.447  1.00  0.00           H
ATOM   1193  HB3 ASN    76      27.381  55.862  59.062  1.00  0.00           H
ATOM   1194  CG  ASN    76      26.560  57.793  59.047  1.00  0.00           C
```

ATOM	1195	OD1	ASN	76	26.938	58.833	59.562	1.00	0.00	O
ATOM	1196	ND2	ASN	76	25.281	57.490	59.067	1.00	0.00	N
ATOM	1197	HD21	ASN	76	24.966	56.609	58.710	1.00	0.00	H
ATOM	1198	HD22	ASN	76	24.661	58.100	59.585	1.00	0.00	H
ATOM	1199	C	ASN	76	29.842	55.872	58.643	1.00	0.00	C
ATOM	1200	O	ASN	76	30.373	55.673	59.731	1.00	0.00	O
ATOM	1201	N	SER	77	29.989	55.037	57.612	1.00	0.00	N
ATOM	1202	H	SER	77	29.510	55.246	56.743	1.00	0.00	H
ATOM	1203	CA	SER	77	30.697	53.761	57.706	1.00	0.00	C
ATOM	1204	HA	SER	77	30.300	53.229	58.566	1.00	0.00	H
ATOM	1205	CB	SER	77	30.433	52.930	56.451	1.00	0.00	C
ATOM	1206	HB2	SER	77	29.369	52.947	56.219	1.00	0.00	H
ATOM	1207	HB3	SER	77	30.989	53.348	55.611	1.00	0.00	H
ATOM	1208	OG	SER	77	30.808	51.589	56.662	1.00	0.00	O
ATOM	1209	HG	SER	77	30.198	51.240	57.334	1.00	0.00	H
ATOM	1210	C	SER	77	32.192	53.924	57.962	1.00	0.00	C
ATOM	1211	O	SER	77	32.726	53.258	58.843	1.00	0.00	O
ATOM	1212	N	ALA	78	32.858	54.873	57.298	1.00	0.00	N
ATOM	1213	H	ALA	78	32.377	55.386	56.565	1.00	0.00	H
ATOM	1214	CA	ALA	78	34.255	55.203	57.588	1.00	0.00	C
ATOM	1215	HA	ALA	78	34.861	54.304	57.459	1.00	0.00	H
ATOM	1216	CB	ALA	78	34.721	56.249	56.570	1.00	0.00	C
ATOM	1217	HB1	ALA	78	35.779	56.464	56.724	1.00	0.00	H
ATOM	1218	HB2	ALA	78	34.144	57.168	56.680	1.00	0.00	H
ATOM	1219	HB3	ALA	78	34.582	55.857	55.563	1.00	0.00	H
ATOM	1220	C	ALA	78	34.450	55.690	59.039	1.00	0.00	C
ATOM	1221	O	ALA	78	35.382	55.259	59.722	1.00	0.00	O
ATOM	1222	N	CYS	79	33.547	56.541	59.542	1.00	0.00	N
ATOM	1223	H	CYS	79	32.799	56.865	58.940	1.00	0.00	H
ATOM	1224	CA	CYS	79	33.593	57.031	60.924	1.00	0.00	C
ATOM	1225	HA	CYS	79	34.597	57.404	61.129	1.00	0.00	H
ATOM	1226	CB	CYS	79	32.607	58.199	61.084	1.00	0.00	C
ATOM	1227	HB2	CYS	79	31.590	57.868	60.864	1.00	0.00	H
ATOM	1228	HB3	CYS	79	32.642	58.556	62.115	1.00	0.00	H
ATOM	1229	SG	CYS	79	33.057	59.569	59.975	1.00	0.00	S
ATOM	1230	HG	CYS	79	32.556	59.026	58.854	1.00	0.00	H
ATOM	1231	C	CYS	79	33.311	55.919	61.951	1.00	0.00	C
ATOM	1232	O	CYS	79	34.016	55.807	62.954	1.00	0.00	O
ATOM	1233	N	LEU	80	32.314	55.066	61.697	1.00	0.00	N
ATOM	1234	H	LEU	80	31.755	55.220	60.863	1.00	0.00	H
ATOM	1235	CA	LEU	80	31.960	53.935	62.560	1.00	0.00	C
ATOM	1236	HA	LEU	80	31.916	54.288	63.589	1.00	0.00	H
ATOM	1237	CB	LEU	80	30.572	53.401	62.161	1.00	0.00	C
ATOM	1238	HB2	LEU	80	30.560	53.266	61.079	1.00	0.00	H
ATOM	1239	HB3	LEU	80	30.418	52.423	62.620	1.00	0.00	H
ATOM	1240	CG	LEU	80	29.409	54.328	62.573	1.00	0.00	C
ATOM	1241	HG	LEU	80	29.630	55.354	62.283	1.00	0.00	H
ATOM	1242	CD1	LEU	80	28.116	53.901	61.878	1.00	0.00	C
ATOM	1243	HD11	LEU	80	28.253	53.941	60.797	1.00	0.00	H
ATOM	1244	HD12	LEU	80	27.309	54.581	62.153	1.00	0.00	H
ATOM	1245	HD13	LEU	80	27.850	52.887	62.174	1.00	0.00	H
ATOM	1246	CD2	LEU	80	29.158	54.290	64.084	1.00	0.00	C

ATOM	1247	HD21	LEU	80	28.959	53.268	64.405	1.00	0.00	H
ATOM	1248	HD22	LEU	80	30.023	54.678	64.620	1.00	0.00	H
ATOM	1249	HD23	LEU	80	28.299	54.917	64.327	1.00	0.00	H
ATOM	1250	C	LEU	80	33.024	52.828	62.534	1.00	0.00	C
ATOM	1251	O	LEU	80	33.303	52.247	63.580	1.00	0.00	O
ATOM	1252	N	ALA	81	33.677	52.581	61.395	1.00	0.00	N
ATOM	1253	H	ALA	81	33.367	53.041	60.545	1.00	0.00	H
ATOM	1254	CA	ALA	81	34.836	51.696	61.305	1.00	0.00	C
ATOM	1255	HA	ALA	81	34.565	50.723	61.718	1.00	0.00	H
ATOM	1256	CB	ALA	81	35.212	51.502	59.830	1.00	0.00	C
ATOM	1257	HB1	ALA	81	35.470	52.459	59.376	1.00	0.00	H
ATOM	1258	HB2	ALA	81	34.371	51.066	59.290	1.00	0.00	H
ATOM	1259	HB3	ALA	81	36.066	50.828	59.756	1.00	0.00	H
ATOM	1260	C	ALA	81	36.014	52.241	62.128	1.00	0.00	C
ATOM	1261	O	ALA	81	36.602	51.494	62.907	1.00	0.00	O
ATOM	1262	N	ALA	82	36.318	53.542	62.039	1.00	0.00	N
ATOM	1263	H	ALA	82	35.828	54.114	61.358	1.00	0.00	H
ATOM	1264	CA	ALA	82	37.343	54.175	62.871	1.00	0.00	C
ATOM	1265	HA	ALA	82	38.284	53.646	62.718	1.00	0.00	H
ATOM	1266	CB	ALA	82	37.530	55.624	62.402	1.00	0.00	C
ATOM	1267	HB1	ALA	82	36.609	56.189	62.541	1.00	0.00	H
ATOM	1268	HB2	ALA	82	37.800	55.638	61.345	1.00	0.00	H
ATOM	1269	HB3	ALA	82	38.327	56.094	62.978	1.00	0.00	H
ATOM	1270	C	ALA	82	37.013	54.092	64.378	1.00	0.00	C
ATOM	1271	O	ALA	82	37.890	53.771	65.178	1.00	0.00	O
ATOM	1272	N	PHE	83	35.752	54.308	64.768	1.00	0.00	N
ATOM	1273	H	PHE	83	35.087	54.624	64.068	1.00	0.00	H
ATOM	1274	CA	PHE	83	35.274	54.144	66.149	1.00	0.00	C
ATOM	1275	HA	PHE	83	35.891	54.752	66.811	1.00	0.00	H
ATOM	1276	CB	PHE	83	33.830	54.668	66.219	1.00	0.00	C
ATOM	1277	HB2	PHE	83	33.830	55.726	65.955	1.00	0.00	H
ATOM	1278	HB3	PHE	83	33.232	54.152	65.469	1.00	0.00	H
ATOM	1279	CG	PHE	83	33.143	54.503	67.563	1.00	0.00	C
ATOM	1280	CD1	PHE	83	33.273	55.497	68.551	1.00	0.00	C
ATOM	1281	HD1	PHE	83	33.873	56.375	68.361	1.00	0.00	H
ATOM	1282	CE1	PHE	83	32.606	55.361	69.783	1.00	0.00	C
ATOM	1283	HE1	PHE	83	32.696	56.133	70.534	1.00	0.00	H
ATOM	1284	CZ	PHE	83	31.810	54.228	70.031	1.00	0.00	C
ATOM	1285	HZ	PHE	83	31.288	54.130	70.974	1.00	0.00	H
ATOM	1286	CE2	PHE	83	31.684	53.230	69.049	1.00	0.00	C
ATOM	1287	HE2	PHE	83	31.068	52.363	69.239	1.00	0.00	H
ATOM	1288	CD2	PHE	83	32.347	53.368	67.816	1.00	0.00	C
ATOM	1289	HD2	PHE	83	32.238	52.604	67.058	1.00	0.00	H
ATOM	1290	C	PHE	83	35.375	52.689	66.642	1.00	0.00	C
ATOM	1291	O	PHE	83	35.871	52.433	67.742	1.00	0.00	O
ATOM	1292	N	ALA	84	34.964	51.720	65.819	1.00	0.00	N
ATOM	1293	H	ALA	84	34.539	51.980	64.935	1.00	0.00	H
ATOM	1294	CA	ALA	84	35.078	50.296	66.127	1.00	0.00	C
ATOM	1295	HA	ALA	84	34.584	50.106	67.081	1.00	0.00	H
ATOM	1296	CB	ALA	84	34.353	49.496	65.037	1.00	0.00	C
ATOM	1297	HB1	ALA	84	33.305	49.795	64.991	1.00	0.00	H
ATOM	1298	HB2	ALA	84	34.407	48.431	65.265	1.00	0.00	H

ATOM 1299 HB3 ALA 84 34.815 49.676 64.065 1.00 0.00 H
ATOM 1300 C ALA 84 36.546 49.864 66.268 1.00 0.00 C
ATOM 1301 O ALA 84 36.887 49.169 67.225 1.00 0.00 O
ATOM 1302 N ILE 85 37.423 50.322 65.370 1.00 0.00 N
ATOM 1303 H ILE 85 37.065 50.869 64.591 1.00 0.00 H
ATOM 1304 CA ILE 85 38.872 50.092 65.432 1.00 0.00 C
ATOM 1305 HA ILE 85 39.043 49.021 65.526 1.00 0.00 H
ATOM 1306 CB ILE 85 39.542 50.549 64.112 1.00 0.00 C
ATOM 1307 HB ILE 85 39.152 51.533 63.847 1.00 0.00 H
ATOM 1308 CG2 ILE 85 41.070 50.671 64.240 1.00 0.00 C
ATOM 1309 HG21 ILE 85 41.316 51.474 64.933 1.00 0.00 H
ATOM 1310 HG22 ILE 85 41.518 50.926 63.281 1.00 0.00 H
ATOM 1311 HG23 ILE 85 41.497 49.734 64.600 1.00 0.00 H
ATOM 1312 CG1 ILE 85 39.177 49.543 62.992 1.00 0.00 C
ATOM 1313 HG12 ILE 85 39.674 48.590 63.179 1.00 0.00 H
ATOM 1314 HG13 ILE 85 38.104 49.361 63.010 1.00 0.00 H
ATOM 1315 CD1 ILE 85 39.520 50.013 61.573 1.00 0.00 C
ATOM 1316 HD11 ILE 85 39.113 51.009 61.399 1.00 0.00 H
ATOM 1317 HD12 ILE 85 39.084 49.322 60.851 1.00 0.00 H
ATOM 1318 HD13 ILE 85 40.598 50.024 61.429 1.00 0.00 H
ATOM 1319 C ILE 85 39.469 50.732 66.694 1.00 0.00 C
ATOM 1320 O ILE 85 40.224 50.064 67.389 1.00 0.00 O
ATOM 1321 N LEU 86 39.073 51.955 67.067 1.00 0.00 N
ATOM 1322 H LEU 86 38.470 52.480 66.441 1.00 0.00 H
ATOM 1323 CA LEU 86 39.505 52.603 68.314 1.00 0.00 C
ATOM 1324 HA LEU 86 40.593 52.660 68.300 1.00 0.00 H
ATOM 1325 CB LEU 86 38.953 54.041 68.349 1.00 0.00 C
ATOM 1326 HB2 LEU 86 39.339 54.581 67.483 1.00 0.00 H
ATOM 1327 HB3 LEU 86 37.869 54.000 68.257 1.00 0.00 H
ATOM 1328 CG LEU 86 39.292 54.840 69.623 1.00 0.00 C
ATOM 1329 HG LEU 86 38.878 54.332 70.494 1.00 0.00 H
ATOM 1330 CD1 LEU 86 40.800 55.016 69.821 1.00 0.00 C
ATOM 1331 HD11 LEU 86 41.270 54.048 69.991 1.00 0.00 H
ATOM 1332 HD12 LEU 86 40.985 55.641 70.695 1.00 0.00 H
ATOM 1333 HD13 LEU 86 41.241 55.487 68.942 1.00 0.00 H
ATOM 1334 CD2 LEU 86 38.658 56.230 69.536 1.00 0.00 C
ATOM 1335 HD21 LEU 86 39.064 56.774 68.684 1.00 0.00 H
ATOM 1336 HD22 LEU 86 37.578 56.132 69.424 1.00 0.00 H
ATOM 1337 HD23 LEU 86 38.866 56.786 70.451 1.00 0.00 H
ATOM 1338 C LEU 86 39.121 51.784 69.559 1.00 0.00 C
ATOM 1339 O LEU 86 39.965 51.557 70.432 1.00 0.00 O
ATOM 1340 N ARG 87 37.891 51.250 69.628 1.00 0.00 N
ATOM 1341 H ARG 87 37.216 51.486 68.904 1.00 0.00 H
ATOM 1342 CA ARG 87 37.534 50.305 70.701 1.00 0.00 C
ATOM 1343 HA ARG 87 37.793 50.778 71.651 1.00 0.00 H
ATOM 1344 CB ARG 87 36.023 50.010 70.717 1.00 0.00 C
ATOM 1345 HB2 ARG 87 35.470 50.950 70.769 1.00 0.00 H
ATOM 1346 HB3 ARG 87 35.731 49.470 69.816 1.00 0.00 H
ATOM 1347 CG ARG 87 35.713 49.167 71.965 1.00 0.00 C
ATOM 1348 HG2 ARG 87 36.220 48.206 71.875 1.00 0.00 H
ATOM 1349 HG3 ARG 87 36.113 49.681 72.841 1.00 0.00 H
ATOM 1350 CD ARG 87 34.234 48.878 72.222 1.00 0.00 C

ATOM	1351	HD2	ARG	87	33.710	49.814	72.427	1.00	0.00	H
ATOM	1352	HD3	ARG	87	33.795	48.412	71.338	1.00	0.00	H
ATOM	1353	NE	ARG	87	34.150	47.991	73.395	1.00	0.00	N
ATOM	1354	HE	ARG	87	34.650	48.308	74.222	1.00	0.00	H
ATOM	1355	CZ	ARG	87	33.912	46.695	73.402	1.00	0.00	C
ATOM	1356	NH1	ARG	87	33.348	46.056	72.420	1.00	0.00	N
ATOM	1357	HH11	ARG	87	32.967	46.553	71.641	1.00	0.00	H
ATOM	1358	HH12	ARG	87	33.285	45.045	72.503	1.00	0.00	H
ATOM	1359	NH2	ARG	87	34.277	45.976	74.412	1.00	0.00	N
ATOM	1360	HH21	ARG	87	34.790	46.382	75.183	1.00	0.00	H
ATOM	1361	HH22	ARG	87	34.177	44.968	74.353	1.00	0.00	H
ATOM	1362	C	ARG	87	38.381	49.029	70.629	1.00	0.00	C
ATOM	1363	O	ARG	87	38.870	48.578	71.658	1.00	0.00	O
ATOM	1364	N	ARG	88	38.615	48.481	69.434	1.00	0.00	N
ATOM	1365	H	ARG	88	38.186	48.936	68.633	1.00	0.00	H
ATOM	1366	CA	ARG	88	39.472	47.304	69.165	1.00	0.00	C
ATOM	1367	HA	ARG	88	39.313	46.579	69.962	1.00	0.00	H
ATOM	1368	CB	ARG	88	39.039	46.660	67.828	1.00	0.00	C
ATOM	1369	HB2	ARG	88	39.147	47.402	67.039	1.00	0.00	H
ATOM	1370	HB3	ARG	88	39.703	45.828	67.586	1.00	0.00	H
ATOM	1371	CG	ARG	88	37.583	46.142	67.824	1.00	0.00	C
ATOM	1372	HG2	ARG	88	36.920	46.872	68.286	1.00	0.00	H
ATOM	1373	HG3	ARG	88	37.256	46.001	66.795	1.00	0.00	H
ATOM	1374	CD	ARG	88	37.440	44.813	68.569	1.00	0.00	C
ATOM	1375	HD2	ARG	88	37.948	44.033	67.997	1.00	0.00	H
ATOM	1376	HD3	ARG	88	37.935	44.917	69.535	1.00	0.00	H
ATOM	1377	NE	ARG	88	36.026	44.434	68.779	1.00	0.00	N
ATOM	1378	HE	ARG	88	35.349	44.749	68.106	1.00	0.00	H
ATOM	1379	CZ	ARG	88	35.552	43.791	69.834	1.00	0.00	C
ATOM	1380	NH1	ARG	88	36.326	43.282	70.749	1.00	0.00	N
ATOM	1381	HH11	ARG	88	37.325	43.218	70.595	1.00	0.00	H
ATOM	1382	HH12	ARG	88	35.896	42.906	71.590	1.00	0.00	H
ATOM	1383	NH2	ARG	88	34.272	43.646	70.016	1.00	0.00	N
ATOM	1384	HH21	ARG	88	33.581	43.954	69.334	1.00	0.00	H
ATOM	1385	HH22	ARG	88	33.972	43.188	70.873	1.00	0.00	H
ATOM	1386	C	ARG	88	40.992	47.593	69.236	1.00	0.00	C
ATOM	1387	O	ARG	88	41.786	46.756	68.817	1.00	0.00	O
ATOM	1388	N	ILE	89	41.378	48.752	69.786	1.00	0.00	N
ATOM	1389	H	ILE	89	40.652	49.438	69.923	1.00	0.00	H
ATOM	1390	CA	ILE	89	42.746	49.146	70.176	1.00	0.00	C
ATOM	1391	HA	ILE	89	43.447	48.363	69.880	1.00	0.00	H
ATOM	1392	CB	ILE	89	43.125	50.451	69.425	1.00	0.00	C
ATOM	1393	HB	ILE	89	42.274	51.128	69.482	1.00	0.00	H
ATOM	1394	CG2	ILE	89	44.332	51.198	70.023	1.00	0.00	C
ATOM	1395	HG21	ILE	89	44.153	51.458	71.065	1.00	0.00	H
ATOM	1396	HG22	ILE	89	44.505	52.128	69.482	1.00	0.00	H
ATOM	1397	HG23	ILE	89	45.225	50.582	69.943	1.00	0.00	H
ATOM	1398	CG1	ILE	89	43.406	50.147	67.937	1.00	0.00	C
ATOM	1399	HG12	ILE	89	44.400	49.714	67.841	1.00	0.00	H
ATOM	1400	HG13	ILE	89	42.697	49.408	67.565	1.00	0.00	H
ATOM	1401	CD1	ILE	89	43.301	51.384	67.036	1.00	0.00	C
ATOM	1402	HD11	ILE	89	42.325	51.851	67.148	1.00	0.00	H

ATOM	1403	HD12	ILE	89	43.430	51.081	65.998	1.00	0.00	H
ATOM	1404	HD13	ILE	89	44.077	52.108	67.282	1.00	0.00	H
ATOM	1405	C	ILE	89	42.865	49.281	71.709	1.00	0.00	C
ATOM	1406	O	ILE	89	43.917	48.982	72.273	1.00	0.00	O
ATOM	1407	N	GLN	90	41.798	49.700	72.410	1.00	0.00	N
ATOM	1408	H	GLN	90	40.982	50.004	71.894	1.00	0.00	H
ATOM	1409	CA	GLN	90	41.724	49.684	73.886	1.00	0.00	C
ATOM	1410	HA	GLN	90	42.746	49.710	74.265	1.00	0.00	H
ATOM	1411	CB	GLN	90	41.036	50.977	74.379	1.00	0.00	C
ATOM	1412	HB2	GLN	90	41.477	51.814	73.834	1.00	0.00	H
ATOM	1413	HB3	GLN	90	39.971	50.945	74.141	1.00	0.00	H
ATOM	1414	CG	GLN	90	41.226	51.245	75.888	1.00	0.00	C
ATOM	1415	HG2	GLN	90	40.475	50.697	76.456	1.00	0.00	H
ATOM	1416	HG3	GLN	90	42.211	50.893	76.196	1.00	0.00	H
ATOM	1417	CD	GLN	90	41.129	52.728	76.249	1.00	0.00	C
ATOM	1418	OE1	GLN	90	42.095	53.355	76.674	1.00	0.00	O
ATOM	1419	NE2	GLN	90	39.980	53.344	76.091	1.00	0.00	N
ATOM	1420	HE21	GLN	90	39.945	54.341	76.264	1.00	0.00	H
ATOM	1421	HE22	GLN	90	39.148	52.822	75.839	1.00	0.00	H
ATOM	1422	C	GLN	90	41.102	48.383	74.455	1.00	0.00	C
ATOM	1423	O	GLN	90	41.050	48.196	75.673	1.00	0.00	O
ATOM	1424	N	GLN	91	40.622	47.490	73.587	1.00	0.00	N
ATOM	1425	H	GLN	91	40.648	47.766	72.612	1.00	0.00	H
ATOM	1426	CA	GLN	91	40.152	46.114	73.813	1.00	0.00	C
ATOM	1427	HA	GLN	91	40.673	45.692	74.674	1.00	0.00	H
ATOM	1428	CB	GLN	91	38.629	46.089	74.086	1.00	0.00	C
ATOM	1429	HB2	GLN	91	38.099	46.637	73.306	1.00	0.00	H
ATOM	1430	HB3	GLN	91	38.292	45.053	74.030	1.00	0.00	H
ATOM	1431	CG	GLN	91	38.216	46.607	75.478	1.00	0.00	C
ATOM	1432	HG2	GLN	91	37.500	45.904	75.898	1.00	0.00	H
ATOM	1433	HG3	GLN	91	39.071	46.619	76.153	1.00	0.00	H
ATOM	1434	CD	GLN	91	37.541	47.977	75.447	1.00	0.00	C
ATOM	1435	OE1	GLN	91	36.365	48.106	75.136	1.00	0.00	O
ATOM	1436	NE2	GLN	91	38.244	49.035	75.790	1.00	0.00	N
ATOM	1437	HE21	GLN	91	37.828	49.954	75.637	1.00	0.00	H
ATOM	1438	HE22	GLN	91	39.233	48.920	75.966	1.00	0.00	H
ATOM	1439	C	GLN	91	40.533	45.252	72.583	1.00	0.00	C
ATOM	1440	O	GLN	91	41.138	45.763	71.646	1.00	0.00	O
ATOM	1441	N	SER	92	40.215	43.950	72.572	1.00	0.00	N
ATOM	1442	H	SER	92	39.650	43.605	73.334	1.00	0.00	H
ATOM	1443	CA	SER	92	40.798	42.910	71.689	1.00	0.00	C
ATOM	1444	HA	SER	92	40.346	41.963	71.988	1.00	0.00	H
ATOM	1445	CB	SER	92	40.447	43.090	70.202	1.00	0.00	C
ATOM	1446	HB2	SER	92	40.573	44.128	69.891	1.00	0.00	H
ATOM	1447	HB3	SER	92	41.129	42.479	69.610	1.00	0.00	H
ATOM	1448	OG	SER	92	39.127	42.653	69.924	1.00	0.00	O
ATOM	1449	HG	SER	92	39.186	42.097	69.129	1.00	0.00	H
ATOM	1450	C	SER	92	42.309	42.696	71.870	1.00	0.00	C
ATOM	1451	O	SER	92	42.720	41.561	72.091	1.00	0.00	O
ATOM	1452	N	GLY	93	43.133	43.744	71.826	1.00	0.00	N
ATOM	1453	H	GLY	93	42.739	44.658	71.622	1.00	0.00	H
ATOM	1454	CA	GLY	93	44.551	43.679	72.187	1.00	0.00	C

ATOM	1455	HA2	GLY	93	44.945	42.689	71.963	1.00	0.00	H
ATOM	1456	HA3	GLY	93	45.108	44.392	71.585	1.00	0.00	H
ATOM	1457	C	GLY	93	44.839	43.991	73.658	1.00	0.00	C
ATOM	1458	O	GLY	93	44.057	44.635	74.362	1.00	0.00	O
ATOM	1459	N	SER	94	46.007	43.552	74.125	1.00	0.00	N
ATOM	1460	H	SER	94	46.489	42.847	73.569	1.00	0.00	H
ATOM	1461	CA	SER	94	46.657	43.988	75.369	1.00	0.00	C
ATOM	1462	HA	SER	94	46.560	45.071	75.466	1.00	0.00	H
ATOM	1463	CB	SER	94	45.990	43.333	76.579	1.00	0.00	C
ATOM	1464	HB2	SER	94	45.627	42.335	76.322	1.00	0.00	H
ATOM	1465	HB3	SER	94	46.702	43.251	77.401	1.00	0.00	H
ATOM	1466	OG	SER	94	44.918	44.171	76.973	1.00	0.00	O
ATOM	1467	HG	SER	94	44.382	44.326	76.172	1.00	0.00	H
ATOM	1468	C	SER	94	48.149	43.655	75.317	1.00	0.00	C
ATOM	1469	O	SER	94	48.544	42.515	75.554	1.00	0.00	O
ATOM	1470	N	ALA	95	48.958	44.657	74.946	1.00	0.00	N
ATOM	1471	H	ALA	95	48.522	45.553	74.803	1.00	0.00	H
ATOM	1472	CA	ALA	95	50.403	44.601	74.651	1.00	0.00	C
ATOM	1473	HA	ALA	95	50.689	45.596	74.305	1.00	0.00	H
ATOM	1474	CB	ALA	95	51.180	44.362	75.952	1.00	0.00	C
ATOM	1475	HB1	ALA	95	50.910	45.116	76.694	1.00	0.00	H
ATOM	1476	HB2	ALA	95	52.250	44.435	75.756	1.00	0.00	H
ATOM	1477	HB3	ALA	95	50.962	43.370	76.349	1.00	0.00	H
ATOM	1478	C	ALA	95	50.845	43.644	73.511	1.00	0.00	C
ATOM	1479	O	ALA	95	51.930	43.822	72.959	1.00	0.00	O
ATOM	1480	N	ASP	96	50.016	42.666	73.136	1.00	0.00	N
ATOM	1481	H	ASP	96	49.204	42.524	73.722	1.00	0.00	H
ATOM	1482	CA	ASP	96	50.329	41.601	72.177	1.00	0.00	C
ATOM	1483	HA	ASP	96	51.213	41.076	72.540	1.00	0.00	H
ATOM	1484	CB	ASP	96	49.180	40.571	72.144	1.00	0.00	C
ATOM	1485	HB2	ASP	96	49.309	39.911	71.284	1.00	0.00	H
ATOM	1486	HB3	ASP	96	49.261	39.957	73.041	1.00	0.00	H
ATOM	1487	CG	ASP	96	47.777	41.193	72.110	1.00	0.00	C
ATOM	1488	OD1	ASP	96	47.064	41.129	73.133	1.00	0.00	O
ATOM	1489	OD2	ASP	96	47.352	41.741	71.065	1.00	0.00	O
ATOM	1490	C	ASP	96	50.691	42.102	70.767	1.00	0.00	C
ATOM	1491	O	ASP	96	51.853	41.993	70.373	1.00	0.00	O
ATOM	1492	N	LEU	97	49.701	42.599	70.012	1.00	0.00	N
ATOM	1493	H	LEU	97	48.775	42.573	70.424	1.00	0.00	H
ATOM	1494	CA	LEU	97	49.823	43.109	68.645	1.00	0.00	C
ATOM	1495	HA	LEU	97	50.642	43.826	68.649	1.00	0.00	H
ATOM	1496	CB	LEU	97	50.169	41.923	67.715	1.00	0.00	C
ATOM	1497	HB2	LEU	97	50.920	41.290	68.185	1.00	0.00	H
ATOM	1498	HB3	LEU	97	49.275	41.313	67.578	1.00	0.00	H
ATOM	1499	CG	LEU	97	50.718	42.328	66.339	1.00	0.00	C
ATOM	1500	HG	LEU	97	49.958	42.886	65.803	1.00	0.00	H
ATOM	1501	CD1	LEU	97	51.966	43.208	66.422	1.00	0.00	C
ATOM	1502	HD11	LEU	97	51.697	44.196	66.793	1.00	0.00	H
ATOM	1503	HD12	LEU	97	52.393	43.332	65.429	1.00	0.00	H
ATOM	1504	HD13	LEU	97	52.701	42.760	67.090	1.00	0.00	H
ATOM	1505	CD2	LEU	97	51.056	41.054	65.556	1.00	0.00	C
ATOM	1506	HD21	LEU	97	51.790	40.463	66.104	1.00	0.00	H

ATOM	1507	HD22	LEU	97	50.153	40.458	65.416	1.00	0.00	H
ATOM	1508	HD23	LEU	97	51.461	41.309	64.578	1.00	0.00	H
ATOM	1509	C	LEU	97	48.558	43.838	68.129	1.00	0.00	C
ATOM	1510	O	LEU	97	48.675	44.692	67.250	1.00	0.00	O
ATOM	1511	N	ARG	98	47.348	43.550	68.651	1.00	0.00	N
ATOM	1512	H	ARG	98	47.304	42.852	69.392	1.00	0.00	H
ATOM	1513	CA	ARG	98	46.086	44.232	68.261	1.00	0.00	C
ATOM	1514	HA	ARG	98	46.109	44.396	67.183	1.00	0.00	H
ATOM	1515	CB	ARG	98	44.853	43.351	68.565	1.00	0.00	C
ATOM	1516	HB2	ARG	98	44.954	42.907	69.555	1.00	0.00	H
ATOM	1517	HB3	ARG	98	43.958	43.977	68.556	1.00	0.00	H
ATOM	1518	CG	ARG	98	44.646	42.245	67.518	1.00	0.00	C
ATOM	1519	HG2	ARG	98	44.625	42.702	66.528	1.00	0.00	H
ATOM	1520	HG3	ARG	98	45.478	41.540	67.556	1.00	0.00	H
ATOM	1521	CD	ARG	98	43.320	41.489	67.713	1.00	0.00	C
ATOM	1522	HD2	ARG	98	42.498	42.206	67.752	1.00	0.00	H
ATOM	1523	HD3	ARG	98	43.166	40.840	66.848	1.00	0.00	H
ATOM	1524	NE	ARG	98	43.333	40.664	68.933	1.00	0.00	N
ATOM	1525	HE	ARG	98	44.129	40.744	69.558	1.00	0.00	H
ATOM	1526	CZ	ARG	98	42.478	39.726	69.289	1.00	0.00	C
ATOM	1527	NH1	ARG	98	41.361	39.499	68.662	1.00	0.00	N
ATOM	1528	HH11	ARG	98	41.039	40.145	67.953	1.00	0.00	H
ATOM	1529	HH12	ARG	98	40.764	38.731	68.943	1.00	0.00	H
ATOM	1530	NH2	ARG	98	42.759	38.988	70.312	1.00	0.00	N
ATOM	1531	HH21	ARG	98	43.597	39.222	70.834	1.00	0.00	H
ATOM	1532	HH22	ARG	98	42.091	38.313	70.665	1.00	0.00	H
ATOM	1533	C	ARG	98	45.976	45.645	68.867	1.00	0.00	C
ATOM	1534	O	ARG	98	45.111	45.926	69.688	1.00	0.00	O
ATOM	1535	N	LYS	99	46.902	46.523	68.475	1.00	0.00	N
ATOM	1536	H	LYS	99	47.601	46.167	67.831	1.00	0.00	H
ATOM	1537	CA	LYS	99	47.015	47.933	68.891	1.00	0.00	C
ATOM	1538	HA	LYS	99	46.012	48.347	69.004	1.00	0.00	H
ATOM	1539	CB	LYS	99	47.722	47.935	70.272	1.00	0.00	C
ATOM	1540	HB2	LYS	99	47.362	47.083	70.853	1.00	0.00	H
ATOM	1541	HB3	LYS	99	48.795	47.796	70.123	1.00	0.00	H
ATOM	1542	CG	LYS	99	47.485	49.184	71.137	1.00	0.00	C
ATOM	1543	HG2	LYS	99	47.835	50.075	70.616	1.00	0.00	H
ATOM	1544	HG3	LYS	99	46.418	49.280	71.329	1.00	0.00	H
ATOM	1545	CD	LYS	99	48.214	49.067	72.485	1.00	0.00	C
ATOM	1546	HD2	LYS	99	47.836	48.196	73.023	1.00	0.00	H
ATOM	1547	HD3	LYS	99	49.284	48.941	72.307	1.00	0.00	H
ATOM	1548	CE	LYS	99	47.983	50.327	73.327	1.00	0.00	C
ATOM	1549	HE2	LYS	99	48.370	51.187	72.774	1.00	0.00	H
ATOM	1550	HE3	LYS	99	46.908	50.471	73.463	1.00	0.00	H
ATOM	1551	NZ	LYS	99	48.652	50.244	74.649	1.00	0.00	N
ATOM	1552	HZ1	LYS	99	48.241	49.529	75.250	1.00	0.00	H
ATOM	1553	HZ2	LYS	99	48.587	51.124	75.139	1.00	0.00	H
ATOM	1554	HZ3	LYS	99	49.639	49.998	74.555	1.00	0.00	H
ATOM	1555	C	LYS	99	47.758	48.786	67.825	1.00	0.00	C
ATOM	1556	O	LYS	99	48.529	49.667	68.184	1.00	0.00	O
ATOM	1557	N	LEU	100	47.798	48.415	66.534	1.00	0.00	N
ATOM	1558	H	LEU	100	48.669	48.735	66.140	1.00	0.00	H

ATOM	1559	CA	LEU	100	46.721	48.415	65.511	1.00	0.00	C
ATOM	1560	HA	LEU	100	47.190	48.020	64.610	1.00	0.00	H
ATOM	1561	CB	LEU	100	45.566	47.446	65.842	1.00	0.00	C
ATOM	1562	HB2	LEU	100	45.982	46.447	65.968	1.00	0.00	H
ATOM	1563	HB3	LEU	100	45.116	47.759	66.783	1.00	0.00	H
ATOM	1564	CG	LEU	100	44.436	47.363	64.798	1.00	0.00	C
ATOM	1565	HG	LEU	100	44.019	48.355	64.623	1.00	0.00	H
ATOM	1566	CD1	LEU	100	44.921	46.777	63.468	1.00	0.00	C
ATOM	1567	HD11	LEU	100	45.671	47.423	63.017	1.00	0.00	H
ATOM	1568	HD12	LEU	100	44.083	46.700	62.774	1.00	0.00	H
ATOM	1569	HD13	LEU	100	45.345	45.785	63.624	1.00	0.00	H
ATOM	1570	CD2	LEU	100	43.320	46.461	65.327	1.00	0.00	C
ATOM	1571	HD21	LEU	100	43.696	45.454	65.503	1.00	0.00	H
ATOM	1572	HD22	LEU	100	42.934	46.866	66.265	1.00	0.00	H
ATOM	1573	HD23	LEU	100	42.504	46.423	64.607	1.00	0.00	H
ATOM	1574	C	LEU	100	46.263	49.841	65.138	1.00	0.00	C
ATOM	1575	O	LEU	100	46.071	50.666	66.021	1.00	0.00	O
ATOM	1576	N	ASP	101	46.129	50.140	63.838	1.00	0.00	N
ATOM	1577	H	ASP	101	46.284	49.421	63.147	1.00	0.00	H
ATOM	1578	CA	ASP	101	45.831	51.483	63.306	1.00	0.00	C
ATOM	1579	HA	ASP	101	45.012	51.917	63.883	1.00	0.00	H
ATOM	1580	CB	ASP	101	47.088	52.351	63.496	1.00	0.00	C
ATOM	1581	HB2	ASP	101	47.493	52.199	64.496	1.00	0.00	H
ATOM	1582	HB3	ASP	101	47.853	52.029	62.793	1.00	0.00	H
ATOM	1583	CG	ASP	101	46.843	53.848	63.350	1.00	0.00	C
ATOM	1584	OD1	ASP	101	45.692	54.297	63.160	1.00	0.00	O
ATOM	1585	OD2	ASP	101	47.824	54.613	63.449	1.00	0.00	O
ATOM	1586	C	ASP	101	45.409	51.426	61.813	1.00	0.00	C
ATOM	1587	O	ASP	101	45.701	50.443	61.132	1.00	0.00	O
ATOM	1588	N	CYS	102	44.747	52.458	61.275	1.00	0.00	N
ATOM	1589	H	CYS	102	44.678	53.307	61.833	1.00	0.00	H
ATOM	1590	CA	CYS	102	44.016	52.412	59.995	1.00	0.00	C
ATOM	1591	HA	CYS	102	44.385	51.579	59.393	1.00	0.00	H
ATOM	1592	CB	CYS	102	42.542	52.131	60.334	1.00	0.00	C
ATOM	1593	HB2	CYS	102	42.495	51.236	60.956	1.00	0.00	H
ATOM	1594	HB3	CYS	102	42.133	52.970	60.900	1.00	0.00	H
ATOM	1595	SG	CYS	102	41.522	51.849	58.855	1.00	0.00	S
ATOM	1596	HG	CYS	102	41.703	53.034	58.257	1.00	0.00	H
ATOM	1597	C	CYS	102	44.166	53.692	59.144	1.00	0.00	C
ATOM	1598	O	CYS	102	44.393	54.783	59.674	1.00	0.00	O
ATOM	1599	N	ARG	103	43.991	53.562	57.822	1.00	0.00	N
ATOM	1600	H	ARG	103	43.891	52.612	57.459	1.00	0.00	H
ATOM	1601	CA	ARG	103	43.919	54.640	56.820	1.00	0.00	C
ATOM	1602	HA	ARG	103	43.687	55.582	57.316	1.00	0.00	H
ATOM	1603	CB	ARG	103	45.278	54.749	56.099	1.00	0.00	C
ATOM	1604	HB2	ARG	103	45.501	53.781	55.645	1.00	0.00	H
ATOM	1605	HB3	ARG	103	45.178	55.468	55.290	1.00	0.00	H
ATOM	1606	CG	ARG	103	46.482	55.147	56.981	1.00	0.00	C
ATOM	1607	HG2	ARG	103	46.620	54.396	57.758	1.00	0.00	H
ATOM	1608	HG3	ARG	103	47.376	55.152	56.356	1.00	0.00	H
ATOM	1609	CD	ARG	103	46.327	56.532	57.628	1.00	0.00	C
ATOM	1610	HD2	ARG	103	46.287	57.273	56.836	1.00	0.00	H

ATOM	1611	HD3	ARG	103	45.386	56.563	58.175	1.00	0.00	H
ATOM	1612	NE	ARG	103	47.418	56.866	58.571	1.00	0.00	N
ATOM	1613	HE	ARG	103	47.926	56.100	58.972	1.00	0.00	H
ATOM	1614	CZ	ARG	103	47.665	58.074	59.055	1.00	0.00	C
ATOM	1615	NH1	ARG	103	47.086	59.139	58.595	1.00	0.00	N
ATOM	1616	HH11	ARG	103	46.492	59.116	57.781	1.00	0.00	H
ATOM	1617	HH12	ARG	103	47.210	60.030	59.057	1.00	0.00	H
ATOM	1618	NH2	ARG	103	48.474	58.265	60.057	1.00	0.00	N
ATOM	1619	HH21	ARG	103	48.887	57.521	60.592	1.00	0.00	H
ATOM	1620	HH22	ARG	103	48.617	59.189	60.428	1.00	0.00	H
ATOM	1621	C	ARG	103	42.773	54.378	55.822	1.00	0.00	C
ATOM	1622	O	ARG	103	42.415	53.224	55.590	1.00	0.00	O
ATOM	1623	N	PHE	104	42.156	55.436	55.284	1.00	0.00	N
ATOM	1624	H	PHE	104	42.446	56.354	55.588	1.00	0.00	H
ATOM	1625	CA	PHE	104	40.895	55.359	54.526	1.00	0.00	C
ATOM	1626	HA	PHE	104	40.851	54.403	54.005	1.00	0.00	H
ATOM	1627	CB	PHE	104	39.729	55.426	55.535	1.00	0.00	C
ATOM	1628	HB2	PHE	104	40.076	55.062	56.503	1.00	0.00	H
ATOM	1629	HB3	PHE	104	39.457	56.470	55.684	1.00	0.00	H
ATOM	1630	CG	PHE	104	38.469	54.651	55.186	1.00	0.00	C
ATOM	1631	CD1	PHE	104	38.109	53.529	55.960	1.00	0.00	C
ATOM	1632	HD1	PHE	104	38.751	53.194	56.762	1.00	0.00	H
ATOM	1633	CE1	PHE	104	36.908	52.844	55.706	1.00	0.00	C
ATOM	1634	HE1	PHE	104	36.635	51.988	56.309	1.00	0.00	H
ATOM	1635	CZ	PHE	104	36.058	53.279	54.676	1.00	0.00	C
ATOM	1636	HZ	PHE	104	35.124	52.767	54.492	1.00	0.00	H
ATOM	1637	CE2	PHE	104	36.420	54.383	53.888	1.00	0.00	C
ATOM	1638	HE2	PHE	104	35.764	54.709	53.095	1.00	0.00	H
ATOM	1639	CD2	PHE	104	37.617	55.076	54.147	1.00	0.00	C
ATOM	1640	HD2	PHE	104	37.869	55.943	53.554	1.00	0.00	H
ATOM	1641	C	PHE	104	40.772	56.492	53.483	1.00	0.00	C
ATOM	1642	O	PHE	104	41.057	57.653	53.785	1.00	0.00	O
ATOM	1643	N	GLY	105	40.293	56.174	52.277	1.00	0.00	N
ATOM	1644	H	GLY	105	40.090	55.195	52.091	1.00	0.00	H
ATOM	1645	CA	GLY	105	39.990	57.118	51.194	1.00	0.00	C
ATOM	1646	HA2	GLY	105	40.061	58.135	51.567	1.00	0.00	H
ATOM	1647	HA3	GLY	105	40.723	57.004	50.397	1.00	0.00	H
ATOM	1648	C	GLY	105	38.596	56.895	50.592	1.00	0.00	C
ATOM	1649	O	GLY	105	38.134	55.753	50.520	1.00	0.00	O
ATOM	1650	N	ILE	106	37.919	57.975	50.168	1.00	0.00	N
ATOM	1651	H	ILE	106	38.345	58.886	50.302	1.00	0.00	H
ATOM	1652	CA	ILE	106	36.559	57.915	49.586	1.00	0.00	C
ATOM	1653	HA	ILE	106	36.384	56.885	49.286	1.00	0.00	H
ATOM	1654	CB	ILE	106	35.448	58.275	50.611	1.00	0.00	C
ATOM	1655	HB	ILE	106	35.467	59.353	50.767	1.00	0.00	H
ATOM	1656	CG2	ILE	106	34.062	57.911	50.037	1.00	0.00	C
ATOM	1657	HG21	ILE	106	33.893	58.385	49.073	1.00	0.00	H
ATOM	1658	HG22	ILE	106	33.270	58.250	50.704	1.00	0.00	H
ATOM	1659	HG23	ILE	106	33.982	56.830	49.920	1.00	0.00	H
ATOM	1660	CG1	ILE	106	35.660	57.592	51.981	1.00	0.00	C
ATOM	1661	HG12	ILE	106	35.731	56.517	51.826	1.00	0.00	H
ATOM	1662	HG13	ILE	106	36.601	57.939	52.408	1.00	0.00	H

ATOM 1663 CD1 ILE 106 34.570 57.869 53.026 1.00 0.00 C
ATOM 1664 HD11 ILE 106 34.341 58.933 53.061 1.00 0.00 H
ATOM 1665 HD12 ILE 106 34.929 57.555 54.004 1.00 0.00 H
ATOM 1666 HD13 ILE 106 33.666 57.309 52.791 1.00 0.00 H
ATOM 1667 C ILE 106 36.420 58.784 48.326 1.00 0.00 C
ATOM 1668 O ILE 106 36.671 59.994 48.362 1.00 0.00 O
ATOM 1669 N ASN 107 35.907 58.186 47.243 1.00 0.00 N
ATOM 1670 H ASN 107 35.660 57.205 47.330 1.00 0.00 H
ATOM 1671 CA ASN 107 35.612 58.848 45.964 1.00 0.00 C
ATOM 1672 HA ASN 107 35.599 59.927 46.119 1.00 0.00 H
ATOM 1673 CB ASN 107 36.726 58.527 44.949 1.00 0.00 C
ATOM 1674 HB2 ASN 107 37.176 57.567 45.179 1.00 0.00 H
ATOM 1675 HB3 ASN 107 36.321 58.460 43.941 1.00 0.00 H
ATOM 1676 CG ASN 107 37.808 59.581 44.954 1.00 0.00 C
ATOM 1677 OD1 ASN 107 38.734 59.564 45.740 1.00 0.00 O
ATOM 1678 ND2 ASN 107 37.716 60.554 44.076 1.00 0.00 N
ATOM 1679 HD21 ASN 107 36.970 60.586 43.406 1.00 0.00 H
ATOM 1680 HD22 ASN 107 38.449 61.239 44.103 1.00 0.00 H
ATOM 1681 C ASN 107 34.229 58.477 45.397 1.00 0.00 C
ATOM 1682 O ASN 107 33.647 57.452 45.746 1.00 0.00 O
ATOM 1683 N ALA 108 33.742 59.297 44.461 1.00 0.00 N
ATOM 1684 H ALA 108 34.265 60.129 44.236 1.00 0.00 H
ATOM 1685 CA ALA 108 32.573 59.033 43.621 1.00 0.00 C
ATOM 1686 HA ALA 108 32.402 57.957 43.598 1.00 0.00 H
ATOM 1687 CB ALA 108 31.342 59.693 44.256 1.00 0.00 C
ATOM 1688 HB1 ALA 108 31.185 59.291 45.258 1.00 0.00 H
ATOM 1689 HB2 ALA 108 30.456 59.481 43.656 1.00 0.00 H
ATOM 1690 HB3 ALA 108 31.484 60.772 44.320 1.00 0.00 H
ATOM 1691 C ALA 108 32.842 59.530 42.177 1.00 0.00 C
ATOM 1692 O ALA 108 33.598 60.487 41.999 1.00 0.00 O
ATOM 1693 N GLY 109 32.282 58.933 41.121 1.00 0.00 N
ATOM 1694 H GLY 109 32.626 59.226 40.220 1.00 0.00 H
ATOM 1695 CA GLY 109 31.337 57.810 41.169 1.00 0.00 C
ATOM 1696 HA2 GLY 109 31.706 57.051 41.852 1.00 0.00 H
ATOM 1697 HA3 GLY 109 30.387 58.178 41.557 1.00 0.00 H
ATOM 1698 C GLY 109 31.035 57.084 39.856 1.00 0.00 C
ATOM 1699 O GLY 109 30.564 55.954 39.920 1.00 0.00 O
ATOM 1700 N ASP 110 31.277 57.678 38.685 1.00 0.00 N
ATOM 1701 H ASP 110 31.700 58.589 38.670 1.00 0.00 H
ATOM 1702 CA ASP 110 30.980 57.054 37.384 1.00 0.00 C
ATOM 1703 HA ASP 110 29.921 56.791 37.355 1.00 0.00 H
ATOM 1704 CB ASP 110 31.254 58.104 36.288 1.00 0.00 C
ATOM 1705 HB2 ASP 110 30.851 59.063 36.620 1.00 0.00 H
ATOM 1706 HB3 ASP 110 32.332 58.224 36.166 1.00 0.00 H
ATOM 1707 CG ASP 110 30.621 57.798 34.927 1.00 0.00 C
ATOM 1708 OD1 ASP 110 29.837 56.834 34.827 1.00 0.00 O
ATOM 1709 OD2 ASP 110 30.880 58.583 33.982 1.00 0.00 O
ATOM 1710 C ASP 110 31.799 55.762 37.185 1.00 0.00 C
ATOM 1711 O ASP 110 33.029 55.785 37.330 1.00 0.00 O
ATOM 1712 N CYS 111 31.145 54.624 36.922 1.00 0.00 N
ATOM 1713 H CYS 111 30.134 54.650 36.841 1.00 0.00 H
ATOM 1714 CA CYS 111 31.799 53.320 36.769 1.00 0.00 C

ATOM	1715	HA	CYS	111	32.699	53.487	36.188	1.00	0.00	H
ATOM	1716	CB	CYS	111	32.189	52.777	38.160	1.00	0.00	C
ATOM	1717	HB2	CYS	111	32.846	51.914	38.042	1.00	0.00	H
ATOM	1718	HB3	CYS	111	32.728	53.545	38.715	1.00	0.00	H
ATOM	1719	SG	CYS	111	30.732	52.281	39.123	1.00	0.00	S
ATOM	1720	HG	CYS	111	30.043	53.426	38.956	1.00	0.00	H
ATOM	1721	C	CYS	111	30.975	52.289	35.979	1.00	0.00	C
ATOM	1722	O	CYS	111	29.751	52.402	35.862	1.00	0.00	O
ATOM	1723	N	MET	112	31.652	51.250	35.467	1.00	0.00	N
ATOM	1724	H	MET	112	32.651	51.197	35.623	1.00	0.00	H
ATOM	1725	CA	MET	112	30.977	50.019	35.040	1.00	0.00	C
ATOM	1726	HA	MET	112	29.957	50.278	34.766	1.00	0.00	H
ATOM	1727	CB	MET	112	31.618	49.411	33.781	1.00	0.00	C
ATOM	1728	HB2	MET	112	30.958	48.618	33.425	1.00	0.00	H
ATOM	1729	HB3	MET	112	31.668	50.172	33.002	1.00	0.00	H
ATOM	1730	CG	MET	112	33.016	48.810	33.970	1.00	0.00	C
ATOM	1731	HG2	MET	112	33.724	49.619	34.143	1.00	0.00	H
ATOM	1732	HG3	MET	112	33.019	48.152	34.838	1.00	0.00	H
ATOM	1733	SD	MET	112	33.571	47.847	32.539	1.00	0.00	S
ATOM	1734	CE	MET	112	35.214	47.345	33.113	1.00	0.00	C
ATOM	1735	HE1	MET	112	35.725	46.802	32.317	1.00	0.00	H
ATOM	1736	HE2	MET	112	35.797	48.227	33.379	1.00	0.00	H
ATOM	1737	HE3	MET	112	35.114	46.697	33.983	1.00	0.00	H
ATOM	1738	C	MET	112	30.904	49.022	36.204	1.00	0.00	C
ATOM	1739	O	MET	112	31.834	48.937	37.005	1.00	0.00	O
ATOM	1740	N	VAL	113	29.819	48.249	36.280	1.00	0.00	N
ATOM	1741	H	VAL	113	29.084	48.413	35.598	1.00	0.00	H
ATOM	1742	CA	VAL	113	29.562	47.241	37.322	1.00	0.00	C
ATOM	1743	HA	VAL	113	30.492	47.033	37.846	1.00	0.00	H
ATOM	1744	CB	VAL	113	28.527	47.736	38.358	1.00	0.00	C
ATOM	1745	HB	VAL	113	27.542	47.768	37.892	1.00	0.00	H
ATOM	1746	CG1	VAL	113	28.455	46.780	39.557	1.00	0.00	C
ATOM	1747	HG11	VAL	113	28.147	45.787	39.229	1.00	0.00	H
ATOM	1748	HG12	VAL	113	29.431	46.714	40.039	1.00	0.00	H
ATOM	1749	HG13	VAL	113	27.723	47.144	40.278	1.00	0.00	H
ATOM	1750	CG2	VAL	113	28.826	49.145	38.885	1.00	0.00	C
ATOM	1751	HG21	VAL	113	28.703	49.877	38.086	1.00	0.00	H
ATOM	1752	HG22	VAL	113	29.849	49.198	39.249	1.00	0.00	H
ATOM	1753	HG23	VAL	113	28.140	49.404	39.691	1.00	0.00	H
ATOM	1754	C	VAL	113	29.083	45.944	36.672	1.00	0.00	C
ATOM	1755	O	VAL	113	28.098	45.960	35.929	1.00	0.00	O
ATOM	1756	N	GLY	114	29.762	44.826	36.938	1.00	0.00	N
ATOM	1757	H	GLY	114	30.563	44.865	37.565	1.00	0.00	H
ATOM	1758	CA	GLY	114	29.428	43.537	36.330	1.00	0.00	C
ATOM	1759	HA2	GLY	114	28.459	43.206	36.701	1.00	0.00	H
ATOM	1760	HA3	GLY	114	29.341	43.674	35.253	1.00	0.00	H
ATOM	1761	C	GLY	114	30.449	42.426	36.581	1.00	0.00	C
ATOM	1762	O	GLY	114	31.297	42.514	37.471	1.00	0.00	O
ATOM	1763	N	VAL	115	30.341	41.372	35.769	1.00	0.00	N
ATOM	1764	H	VAL	115	29.617	41.393	35.067	1.00	0.00	H
ATOM	1765	CA	VAL	115	31.268	40.231	35.727	1.00	0.00	C
ATOM	1766	HA	VAL	115	31.425	39.884	36.744	1.00	0.00	H

ATOM	1767	CB	VAL	115	30.646	39.074	34.913	1.00	0.00	C
ATOM	1768	HB	VAL	115	30.414	39.431	33.909	1.00	0.00	H
ATOM	1769	CG1	VAL	115	31.584	37.868	34.792	1.00	0.00	C
ATOM	1770	HG11	VAL	115	31.877	37.533	35.784	1.00	0.00	H
ATOM	1771	HG12	VAL	115	31.077	37.060	34.265	1.00	0.00	H
ATOM	1772	HG13	VAL	115	32.469	38.132	34.212	1.00	0.00	H
ATOM	1773	CG2	VAL	115	29.345	38.572	35.559	1.00	0.00	C
ATOM	1774	HG21	VAL	115	28.609	39.374	35.612	1.00	0.00	H
ATOM	1775	HG22	VAL	115	29.545	38.192	36.560	1.00	0.00	H
ATOM	1776	HG23	VAL	115	28.917	37.773	34.950	1.00	0.00	H
ATOM	1777	C	VAL	115	32.624	40.642	35.131	1.00	0.00	C
ATOM	1778	O	VAL	115	32.660	41.332	34.116	1.00	0.00	O
ATOM	1779	N	PHE	116	33.735	40.188	35.722	1.00	0.00	N
ATOM	1780	H	PHE	116	33.636	39.676	36.592	1.00	0.00	H
ATOM	1781	CA	PHE	116	35.086	40.335	35.160	1.00	0.00	C
ATOM	1782	HA	PHE	116	35.000	40.245	34.075	1.00	0.00	H
ATOM	1783	CB	PHE	116	35.641	41.741	35.467	1.00	0.00	C
ATOM	1784	HB2	PHE	116	36.293	42.020	34.638	1.00	0.00	H
ATOM	1785	HB3	PHE	116	34.828	42.469	35.476	1.00	0.00	H
ATOM	1786	CG	PHE	116	36.432	41.871	36.760	1.00	0.00	C
ATOM	1787	CD1	PHE	116	37.807	42.166	36.709	1.00	0.00	C
ATOM	1788	HD1	PHE	116	38.287	42.312	35.754	1.00	0.00	H
ATOM	1789	CE1	PHE	116	38.552	42.295	37.896	1.00	0.00	C
ATOM	1790	HE1	PHE	116	39.600	42.550	37.853	1.00	0.00	H
ATOM	1791	CZ	PHE	116	37.931	42.104	39.140	1.00	0.00	C
ATOM	1792	HZ	PHE	116	38.507	42.194	40.052	1.00	0.00	H
ATOM	1793	CE2	PHE	116	36.560	41.805	39.194	1.00	0.00	C
ATOM	1794	HE2	PHE	116	36.081	41.666	40.153	1.00	0.00	H
ATOM	1795	CD2	PHE	116	35.807	41.698	38.009	1.00	0.00	C
ATOM	1796	HD2	PHE	116	34.749	41.479	38.056	1.00	0.00	H
ATOM	1797	C	PHE	116	36.051	39.231	35.641	1.00	0.00	C
ATOM	1798	O	PHE	116	35.863	38.642	36.710	1.00	0.00	O
ATOM	1799	N	GLY	117	37.107	38.956	34.872	1.00	0.00	N
ATOM	1800	H	GLY	117	37.197	39.432	33.984	1.00	0.00	H
ATOM	1801	CA	GLY	117	38.134	37.953	35.184	1.00	0.00	C
ATOM	1802	HA2	GLY	117	39.002	38.443	35.626	1.00	0.00	H
ATOM	1803	HA3	GLY	117	37.750	37.223	35.897	1.00	0.00	H
ATOM	1804	C	GLY	117	38.586	37.221	33.923	1.00	0.00	C
ATOM	1805	O	GLY	117	38.863	37.863	32.913	1.00	0.00	O
ATOM	1806	N	CYS	118	38.610	35.888	33.975	1.00	0.00	N
ATOM	1807	H	CYS	118	38.370	35.430	34.847	1.00	0.00	H
ATOM	1808	CA	CYS	118	38.744	35.019	32.802	1.00	0.00	C
ATOM	1809	HA	CYS	118	38.512	35.591	31.900	1.00	0.00	H
ATOM	1810	CB	CYS	118	40.191	34.520	32.686	1.00	0.00	C
ATOM	1811	HB2	CYS	118	40.307	33.979	31.744	1.00	0.00	H
ATOM	1812	HB3	CYS	118	40.872	35.373	32.679	1.00	0.00	H
ATOM	1813	SG	CYS	118	40.596	33.412	34.068	1.00	0.00	S
ATOM	1814	HG	CYS	118	41.109	34.368	34.860	1.00	0.00	H
ATOM	1815	C	CYS	118	37.740	33.855	32.872	1.00	0.00	C
ATOM	1816	O	CYS	118	37.129	33.597	33.912	1.00	0.00	O
ATOM	1817	N	SER	119	37.604	33.101	31.781	1.00	0.00	N
ATOM	1818	H	SER	119	38.148	33.358	30.957	1.00	0.00	H

ATOM	1819	CA	SER	119	36.617	32.018	31.619	1.00	0.00	C
ATOM	1820	HA	SER	119	35.616	32.443	31.698	1.00	0.00	H
ATOM	1821	CB	SER	119	36.769	31.402	30.223	1.00	0.00	C
ATOM	1822	HB2	SER	119	37.736	30.902	30.145	1.00	0.00	H
ATOM	1823	HB3	SER	119	35.983	30.661	30.078	1.00	0.00	H
ATOM	1824	OG	SER	119	36.682	32.400	29.217	1.00	0.00	O
ATOM	1825	HG	SER	119	37.582	32.828	29.178	1.00	0.00	H
ATOM	1826	C	SER	119	36.728	30.892	32.660	1.00	0.00	C
ATOM	1827	O	SER	119	35.757	30.168	32.897	1.00	0.00	O
ATOM	1828	N	LYS	120	37.907	30.742	33.283	1.00	0.00	N
ATOM	1829	H	LYS	120	38.639	31.387	33.013	1.00	0.00	H
ATOM	1830	CA	LYS	120	38.220	29.751	34.329	1.00	0.00	C
ATOM	1831	HA	LYS	120	37.642	28.842	34.149	1.00	0.00	H
ATOM	1832	CB	LYS	120	39.724	29.403	34.268	1.00	0.00	C
ATOM	1833	HB2	LYS	120	40.298	30.259	34.628	1.00	0.00	H
ATOM	1834	HB3	LYS	120	39.911	28.566	34.944	1.00	0.00	H
ATOM	1835	CG	LYS	120	40.241	29.031	32.863	1.00	0.00	C
ATOM	1836	HG2	LYS	120	39.659	28.198	32.466	1.00	0.00	H
ATOM	1837	HG3	LYS	120	40.129	29.889	32.199	1.00	0.00	H
ATOM	1838	CD	LYS	120	41.726	28.641	32.901	1.00	0.00	C
ATOM	1839	HD2	LYS	120	42.281	29.393	33.468	1.00	0.00	H
ATOM	1840	HD3	LYS	120	41.837	27.675	33.396	1.00	0.00	H
ATOM	1841	CE	LYS	120	42.309	28.572	31.483	1.00	0.00	C
ATOM	1842	HE2	LYS	120	41.756	27.838	30.887	1.00	0.00	H
ATOM	1843	HE3	LYS	120	42.193	29.553	31.015	1.00	0.00	H
ATOM	1844	NZ	LYS	120	43.743	28.215	31.517	1.00	0.00	N
ATOM	1845	HZ1	LYS	120	43.888	27.219	31.666	1.00	0.00	H
ATOM	1846	HZ2	LYS	120	44.235	28.718	32.251	1.00	0.00	H
ATOM	1847	HZ3	LYS	120	44.214	28.443	30.644	1.00	0.00	H
ATOM	1848	C	LYS	120	37.865	30.231	35.747	1.00	0.00	C
ATOM	1849	O	LYS	120	37.611	29.407	36.625	1.00	0.00	O
ATOM	1850	N	LYS	121	37.880	31.553	35.976	1.00	0.00	N
ATOM	1851	H	LYS	121	38.045	32.151	35.174	1.00	0.00	H
ATOM	1852	CA	LYS	121	37.634	32.218	37.266	1.00	0.00	C
ATOM	1853	HA	LYS	121	36.816	31.697	37.766	1.00	0.00	H
ATOM	1854	CB	LYS	121	38.888	32.097	38.162	1.00	0.00	C
ATOM	1855	HB2	LYS	121	38.994	31.055	38.469	1.00	0.00	H
ATOM	1856	HB3	LYS	121	39.769	32.354	37.569	1.00	0.00	H
ATOM	1857	CG	LYS	121	38.907	32.982	39.424	1.00	0.00	C
ATOM	1858	HG2	LYS	121	39.788	32.719	40.012	1.00	0.00	H
ATOM	1859	HG3	LYS	121	39.033	34.019	39.109	1.00	0.00	H
ATOM	1860	CD	LYS	121	37.668	32.893	40.334	1.00	0.00	C
ATOM	1861	HD2	LYS	121	36.765	33.041	39.744	1.00	0.00	H
ATOM	1862	HD3	LYS	121	37.620	31.917	40.818	1.00	0.00	H
ATOM	1863	CE	LYS	121	37.766	34.007	41.385	1.00	0.00	C
ATOM	1864	HE2	LYS	121	38.435	33.700	42.194	1.00	0.00	H
ATOM	1865	HE3	LYS	121	38.207	34.883	40.904	1.00	0.00	H
ATOM	1866	NZ	LYS	121	36.448	34.408	41.928	1.00	0.00	N
ATOM	1867	HZ1	LYS	121	36.532	35.324	42.355	1.00	0.00	H
ATOM	1868	HZ2	LYS	121	36.126	33.803	42.680	1.00	0.00	H
ATOM	1869	HZ3	LYS	121	35.750	34.496	41.193	1.00	0.00	H
ATOM	1870	C	LYS	121	37.206	33.672	37.034	1.00	0.00	C

ATOM	1871	O	LYS	121	38.017	34.552	36.738	1.00	0.00	O
ATOM	1872	N	LEU	122	35.912	33.907	37.233	1.00	0.00	N
ATOM	1873	H	LEU	122	35.328	33.118	37.456	1.00	0.00	H
ATOM	1874	CA	LEU	122	35.266	35.217	37.182	1.00	0.00	C
ATOM	1875	HA	LEU	122	35.978	35.936	36.773	1.00	0.00	H
ATOM	1876	CB	LEU	122	34.093	35.158	36.183	1.00	0.00	C
ATOM	1877	HB2	LEU	122	33.665	36.153	36.079	1.00	0.00	H
ATOM	1878	HB3	LEU	122	34.528	34.905	35.213	1.00	0.00	H
ATOM	1879	CG	LEU	122	32.968	34.143	36.484	1.00	0.00	C
ATOM	1880	HG	LEU	122	33.401	33.203	36.825	1.00	0.00	H
ATOM	1881	CD1	LEU	122	31.974	34.637	37.540	1.00	0.00	C
ATOM	1882	HD11	LEU	122	32.461	34.728	38.508	1.00	0.00	H
ATOM	1883	HD12	LEU	122	31.166	33.912	37.642	1.00	0.00	H
ATOM	1884	HD13	LEU	122	31.557	35.599	37.246	1.00	0.00	H
ATOM	1885	CD2	LEU	122	32.183	33.854	35.205	1.00	0.00	C
ATOM	1886	HD21	LEU	122	31.692	34.757	34.845	1.00	0.00	H
ATOM	1887	HD22	LEU	122	32.856	33.480	34.433	1.00	0.00	H
ATOM	1888	HD23	LEU	122	31.437	33.084	35.398	1.00	0.00	H
ATOM	1889	C	LEU	122	34.901	35.738	38.583	1.00	0.00	C
ATOM	1890	O	LEU	122	34.966	35.017	39.583	1.00	0.00	O
ATOM	1891	N	ASN	123	34.554	37.019	38.642	1.00	0.00	N
ATOM	1892	H	ASN	123	34.562	37.539	37.770	1.00	0.00	H
ATOM	1893	CA	ASN	123	34.307	37.823	39.838	1.00	0.00	C
ATOM	1894	HA	ASN	123	33.955	37.182	40.645	1.00	0.00	H
ATOM	1895	CB	ASN	123	35.614	38.525	40.265	1.00	0.00	C
ATOM	1896	HB2	ASN	123	35.748	39.392	39.627	1.00	0.00	H
ATOM	1897	HB3	ASN	123	35.525	38.870	41.294	1.00	0.00	H
ATOM	1898	CG	ASN	123	36.873	37.688	40.137	1.00	0.00	C
ATOM	1899	OD1	ASN	123	37.289	37.003	41.057	1.00	0.00	O
ATOM	1900	ND2	ASN	123	37.500	37.694	38.981	1.00	0.00	N
ATOM	1901	HD21	ASN	123	37.098	38.215	38.207	1.00	0.00	H
ATOM	1902	HD22	ASN	123	38.298	37.099	38.870	1.00	0.00	H
ATOM	1903	C	ASN	123	33.232	38.875	39.504	1.00	0.00	C
ATOM	1904	O	ASN	123	33.041	39.172	38.324	1.00	0.00	O
ATOM	1905	N	TYR	124	32.576	39.486	40.495	1.00	0.00	N
ATOM	1906	H	TYR	124	32.722	39.172	41.446	1.00	0.00	H
ATOM	1907	CA	TYR	124	31.588	40.554	40.262	1.00	0.00	C
ATOM	1908	HA	TYR	124	31.599	40.832	39.210	1.00	0.00	H
ATOM	1909	CB	TYR	124	30.186	40.013	40.566	1.00	0.00	C
ATOM	1910	HB2	TYR	124	30.083	39.032	40.099	1.00	0.00	H
ATOM	1911	HB3	TYR	124	30.076	39.870	41.642	1.00	0.00	H
ATOM	1912	CG	TYR	124	29.063	40.899	40.063	1.00	0.00	C
ATOM	1913	CD1	TYR	124	28.650	42.029	40.797	1.00	0.00	C
ATOM	1914	HD1	TYR	124	29.155	42.283	41.718	1.00	0.00	H
ATOM	1915	CE1	TYR	124	27.585	42.824	40.326	1.00	0.00	C
ATOM	1916	HE1	TYR	124	27.253	43.690	40.877	1.00	0.00	H
ATOM	1917	CZ	TYR	124	26.929	42.487	39.123	1.00	0.00	C
ATOM	1918	OH	TYR	124	25.879	43.235	38.692	1.00	0.00	O
ATOM	1919	HH	TYR	124	25.317	42.738	38.077	1.00	0.00	H
ATOM	1920	CE2	TYR	124	27.365	41.371	38.377	1.00	0.00	C
ATOM	1921	HE2	TYR	124	26.888	41.120	37.444	1.00	0.00	H
ATOM	1922	CD2	TYR	124	28.428	40.582	38.847	1.00	0.00	C

ATOM	1923	HD2	TYR	124	28.751	39.724	38.275	1.00	0.00	H
ATOM	1924	C	TYR	124	31.906	41.821	41.073	1.00	0.00	C
ATOM	1925	O	TYR	124	31.884	41.783	42.303	1.00	0.00	O
ATOM	1926	N	THR	125	32.193	42.949	40.409	1.00	0.00	N
ATOM	1927	H	THR	125	32.106	42.954	39.395	1.00	0.00	H
ATOM	1928	CA	THR	125	32.555	44.219	41.079	1.00	0.00	C
ATOM	1929	HA	THR	125	31.897	44.344	41.938	1.00	0.00	H
ATOM	1930	CB	THR	125	33.997	44.148	41.632	1.00	0.00	C
ATOM	1931	HB	THR	125	34.156	43.172	42.093	1.00	0.00	H
ATOM	1932	CG2	THR	125	35.080	44.379	40.580	1.00	0.00	C
ATOM	1933	HG21	THR	125	36.044	44.054	40.970	1.00	0.00	H
ATOM	1934	HG22	THR	125	35.149	45.432	40.314	1.00	0.00	H
ATOM	1935	HG23	THR	125	34.843	43.813	39.681	1.00	0.00	H
ATOM	1936	OG1	THR	125	34.182	45.138	42.621	1.00	0.00	O
ATOM	1937	HG1	THR	125	35.101	45.087	42.900	1.00	0.00	H
ATOM	1938	C	THR	125	32.351	45.454	40.183	1.00	0.00	C
ATOM	1939	O	THR	125	31.885	45.339	39.048	1.00	0.00	O
ATOM	1940	N	ALA	126	32.681	46.641	40.702	1.00	0.00	N
ATOM	1941	H	ALA	126	33.116	46.623	41.616	1.00	0.00	H
ATOM	1942	CA	ALA	126	32.739	47.907	39.969	1.00	0.00	C
ATOM	1943	HA	ALA	126	32.127	47.822	39.076	1.00	0.00	H
ATOM	1944	CB	ALA	126	32.152	49.011	40.852	1.00	0.00	C
ATOM	1945	HB1	ALA	126	32.093	49.939	40.284	1.00	0.00	H
ATOM	1946	HB2	ALA	126	32.796	49.171	41.717	1.00	0.00	H
ATOM	1947	HB3	ALA	126	31.151	48.733	41.185	1.00	0.00	H
ATOM	1948	C	ALA	126	34.177	48.269	39.546	1.00	0.00	C
ATOM	1949	O	ALA	126	35.113	48.041	40.315	1.00	0.00	O
ATOM	1950	N	ILE	127	34.357	48.869	38.361	1.00	0.00	N
ATOM	1951	H	ILE	127	33.533	49.025	37.785	1.00	0.00	H
ATOM	1952	CA	ILE	127	35.666	49.253	37.797	1.00	0.00	C
ATOM	1953	HA	ILE	127	36.363	49.399	38.619	1.00	0.00	H
ATOM	1954	CB	ILE	127	36.227	48.130	36.879	1.00	0.00	C
ATOM	1955	HB	ILE	127	35.558	48.029	36.023	1.00	0.00	H
ATOM	1956	CG2	ILE	127	37.618	48.529	36.344	1.00	0.00	C
ATOM	1957	HG21	ILE	127	37.557	49.449	35.766	1.00	0.00	H
ATOM	1958	HG22	ILE	127	38.007	47.763	35.673	1.00	0.00	H
ATOM	1959	HG23	ILE	127	38.318	48.671	37.167	1.00	0.00	H
ATOM	1960	CG1	ILE	127	36.273	46.757	37.596	1.00	0.00	C
ATOM	1961	HG12	ILE	127	36.851	46.844	38.514	1.00	0.00	H
ATOM	1962	HG13	ILE	127	35.258	46.463	37.859	1.00	0.00	H
ATOM	1963	CD1	ILE	127	36.851	45.597	36.785	1.00	0.00	C
ATOM	1964	HD11	ILE	127	36.367	45.541	35.810	1.00	0.00	H
ATOM	1965	HD12	ILE	127	36.668	44.671	37.326	1.00	0.00	H
ATOM	1966	HD13	ILE	127	37.927	45.718	36.667	1.00	0.00	H
ATOM	1967	C	ILE	127	35.570	50.590	37.038	1.00	0.00	C
ATOM	1968	O	ILE	127	34.745	50.722	36.135	1.00	0.00	O
ATOM	1969	N	SER	128	36.427	51.565	37.382	1.00	0.00	N
ATOM	1970	H	SER	128	37.034	51.412	38.175	1.00	0.00	H
ATOM	1971	CA	SER	128	36.704	52.797	36.611	1.00	0.00	C
ATOM	1972	HA	SER	128	37.046	52.526	35.612	1.00	0.00	H
ATOM	1973	CB	SER	128	35.449	53.674	36.474	1.00	0.00	C
ATOM	1974	HB2	SER	128	35.647	54.484	35.771	1.00	0.00	H

ATOM	1975	HB3	SER	128	34.640	53.086	36.057	1.00	0.00	H
ATOM	1976	OG	SER	128	35.068	54.234	37.717	1.00	0.00	O
ATOM	1977	HG	SER	128	34.270	54.784	37.546	1.00	0.00	H
ATOM	1978	C	SER	128	37.783	53.669	37.275	1.00	0.00	C
ATOM	1979	O	SER	128	38.256	53.371	38.377	1.00	0.00	O
ATOM	1980	N	ASP	129	38.075	54.816	36.647	1.00	0.00	N
ATOM	1981	H	ASP	129	37.693	54.961	35.721	1.00	0.00	H
ATOM	1982	CA	ASP	129	38.843	55.938	37.207	1.00	0.00	C
ATOM	1983	HA	ASP	129	39.893	55.651	37.227	1.00	0.00	H
ATOM	1984	CB	ASP	129	38.693	57.134	36.240	1.00	0.00	C
ATOM	1985	HB2	ASP	129	38.888	56.790	35.223	1.00	0.00	H
ATOM	1986	HB3	ASP	129	37.665	57.501	36.281	1.00	0.00	H
ATOM	1987	CG	ASP	129	39.653	58.291	36.536	1.00	0.00	C
ATOM	1988	OD1	ASP	129	40.608	58.107	37.318	1.00	0.00	O
ATOM	1989	OD2	ASP	129	39.485	59.421	36.004	1.00	0.00	O
ATOM	1990	C	ASP	129	38.416	56.338	38.645	1.00	0.00	C
ATOM	1991	O	ASP	129	39.239	56.759	39.458	1.00	0.00	O
ATOM	1992	N	THR	130	37.147	56.143	39.031	1.00	0.00	N
ATOM	1993	H	THR	130	36.494	55.733	38.375	1.00	0.00	H
ATOM	1994	CA	THR	130	36.696	56.453	40.401	1.00	0.00	C
ATOM	1995	HA	THR	130	36.883	57.510	40.590	1.00	0.00	H
ATOM	1996	CB	THR	130	35.186	56.209	40.572	1.00	0.00	C
ATOM	1997	HB	THR	130	34.954	55.161	40.377	1.00	0.00	H
ATOM	1998	CG2	THR	130	34.717	56.576	41.980	1.00	0.00	C
ATOM	1999	HG21	THR	130	33.668	56.315	42.086	1.00	0.00	H
ATOM	2000	HG22	THR	130	34.860	57.641	42.145	1.00	0.00	H
ATOM	2001	HG23	THR	130	35.269	56.028	42.739	1.00	0.00	H
ATOM	2002	OG1	THR	130	34.438	57.033	39.701	1.00	0.00	O
ATOM	2003	HG1	THR	130	34.220	56.538	38.897	1.00	0.00	H
ATOM	2004	C	THR	130	37.479	55.661	41.457	1.00	0.00	C
ATOM	2005	O	THR	130	37.866	56.218	42.486	1.00	0.00	O
ATOM	2006	N	VAL	131	37.740	54.371	41.211	1.00	0.00	N
ATOM	2007	H	VAL	131	37.495	53.995	40.301	1.00	0.00	H
ATOM	2008	CA	VAL	131	38.438	53.496	42.171	1.00	0.00	C
ATOM	2009	HA	VAL	131	38.146	53.804	43.172	1.00	0.00	H
ATOM	2010	CB	VAL	131	38.035	52.019	42.001	1.00	0.00	C
ATOM	2011	HB	VAL	131	38.597	51.611	41.168	1.00	0.00	H
ATOM	2012	CG1	VAL	131	38.391	51.246	43.278	1.00	0.00	C
ATOM	2013	HG11	VAL	131	39.471	51.216	43.412	1.00	0.00	H
ATOM	2014	HG12	VAL	131	37.958	51.715	44.156	1.00	0.00	H
ATOM	2015	HG13	VAL	131	38.009	50.232	43.221	1.00	0.00	H
ATOM	2016	CG2	VAL	131	36.550	51.783	41.679	1.00	0.00	C
ATOM	2017	HG21	VAL	131	35.918	52.208	42.453	1.00	0.00	H
ATOM	2018	HG22	VAL	131	36.354	50.712	41.612	1.00	0.00	H
ATOM	2019	HG23	VAL	131	36.294	52.229	40.718	1.00	0.00	H
ATOM	2020	C	VAL	131	39.962	53.659	42.095	1.00	0.00	C
ATOM	2021	O	VAL	131	40.644	53.581	43.116	1.00	0.00	O
ATOM	2022	N	ASN	132	40.485	53.986	40.909	1.00	0.00	N
ATOM	2023	H	ASN	132	39.864	54.009	40.110	1.00	0.00	H
ATOM	2024	CA	ASN	132	41.860	54.456	40.710	1.00	0.00	C
ATOM	2025	HA	ASN	132	42.557	53.665	40.980	1.00	0.00	H
ATOM	2026	CB	ASN	132	42.005	54.795	39.214	1.00	0.00	C

ATOM	2027	HB2	ASN	132	41.915	53.885	38.623	1.00	0.00	H
ATOM	2028	HB3	ASN	132	41.190	55.444	38.927	1.00	0.00	H
ATOM	2029	CG	ASN	132	43.281	55.505	38.799	1.00	0.00	C
ATOM	2030	OD1	ASN	132	44.336	55.406	39.410	1.00	0.00	O
ATOM	2031	ND2	ASN	132	43.226	56.260	37.731	1.00	0.00	N
ATOM	2032	HD21	ASN	132	42.348	56.402	37.240	1.00	0.00	H
ATOM	2033	HD22	ASN	132	44.082	56.402	37.223	1.00	0.00	H
ATOM	2034	C	ASN	132	42.166	55.661	41.613	1.00	0.00	C
ATOM	2035	O	ASN	132	43.117	55.632	42.398	1.00	0.00	O
ATOM	2036	N	VAL	133	41.315	56.694	41.569	1.00	0.00	N
ATOM	2037	H	VAL	133	40.553	56.673	40.895	1.00	0.00	H
ATOM	2038	CA	VAL	133	41.465	57.860	42.447	1.00	0.00	C
ATOM	2039	HA	VAL	133	42.499	58.195	42.368	1.00	0.00	H
ATOM	2040	CB	VAL	133	40.579	59.040	42.003	1.00	0.00	C
ATOM	2041	HB	VAL	133	39.530	58.743	42.033	1.00	0.00	H
ATOM	2042	CG1	VAL	133	40.782	60.273	42.892	1.00	0.00	C
ATOM	2043	HG11	VAL	133	41.831	60.569	42.896	1.00	0.00	H
ATOM	2044	HG12	VAL	133	40.174	61.102	42.529	1.00	0.00	H
ATOM	2045	HG13	VAL	133	40.482	60.046	43.912	1.00	0.00	H
ATOM	2046	CG2	VAL	133	40.938	59.500	40.582	1.00	0.00	C
ATOM	2047	HG21	VAL	133	41.974	59.837	40.552	1.00	0.00	H
ATOM	2048	HG22	VAL	133	40.269	60.307	40.285	1.00	0.00	H
ATOM	2049	HG23	VAL	133	40.813	58.675	39.882	1.00	0.00	H
ATOM	2050	C	VAL	133	41.261	57.475	43.916	1.00	0.00	C
ATOM	2051	O	VAL	133	42.100	57.857	44.723	1.00	0.00	O
ATOM	2052	N	ALA	134	40.261	56.654	44.271	1.00	0.00	N
ATOM	2053	H	ALA	134	39.593	56.363	43.567	1.00	0.00	H
ATOM	2054	CA	ALA	134	40.044	56.217	45.661	1.00	0.00	C
ATOM	2055	HA	ALA	134	39.759	57.089	46.252	1.00	0.00	H
ATOM	2056	CB	ALA	134	38.892	55.200	45.706	1.00	0.00	C
ATOM	2057	HB1	ALA	134	38.025	55.569	45.165	1.00	0.00	H
ATOM	2058	HB2	ALA	134	38.612	55.019	46.745	1.00	0.00	H
ATOM	2059	HB3	ALA	134	39.205	54.255	45.261	1.00	0.00	H
ATOM	2060	C	ALA	134	41.308	55.609	46.305	1.00	0.00	C
ATOM	2061	O	ALA	134	41.672	55.969	47.424	1.00	0.00	O
ATOM	2062	N	SER	135	42.009	54.730	45.579	1.00	0.00	N
ATOM	2063	H	SER	135	41.647	54.473	44.666	1.00	0.00	H
ATOM	2064	CA	SER	135	43.245	54.089	46.048	1.00	0.00	C
ATOM	2065	HA	SER	135	43.066	53.691	47.046	1.00	0.00	H
ATOM	2066	CB	SER	135	43.564	52.911	45.120	1.00	0.00	C
ATOM	2067	HB2	SER	135	42.688	52.266	45.039	1.00	0.00	H
ATOM	2068	HB3	SER	135	43.810	53.287	44.125	1.00	0.00	H
ATOM	2069	OG	SER	135	44.648	52.153	45.612	1.00	0.00	O
ATOM	2070	HG	SER	135	44.393	51.770	46.463	1.00	0.00	H
ATOM	2071	C	SER	135	44.425	55.070	46.162	1.00	0.00	C
ATOM	2072	O	SER	135	45.146	55.067	47.166	1.00	0.00	O
ATOM	2073	N	ARG	136	44.596	55.985	45.192	1.00	0.00	N
ATOM	2074	H	ARG	136	43.969	55.961	44.392	1.00	0.00	H
ATOM	2075	CA	ARG	136	45.623	57.048	45.271	1.00	0.00	C
ATOM	2076	HA	ARG	136	46.560	56.582	45.583	1.00	0.00	H
ATOM	2077	CB	ARG	136	45.867	57.691	43.882	1.00	0.00	C
ATOM	2078	HB2	ARG	136	44.937	58.136	43.524	1.00	0.00	H

ATOM 2079 HB3 ARG 136 46.602 58.489 44.002 1.00 0.00 H
ATOM 2080 CG ARG 136 46.399 56.700 42.815 1.00 0.00 C
ATOM 2081 HG2 ARG 136 47.246 56.148 43.223 1.00 0.00 H
ATOM 2082 HG3 ARG 136 45.615 55.981 42.587 1.00 0.00 H
ATOM 2083 CD ARG 136 46.851 57.385 41.504 1.00 0.00 C
ATOM 2084 HD2 ARG 136 46.201 58.241 41.317 1.00 0.00 H
ATOM 2085 HD3 ARG 136 47.872 57.752 41.629 1.00 0.00 H
ATOM 2086 NE ARG 136 46.754 56.480 40.334 1.00 0.00 N
ATOM 2087 HE ARG 136 45.872 55.984 40.229 1.00 0.00 H
ATOM 2088 CZ ARG 136 47.598 56.279 39.329 1.00 0.00 C
ATOM 2089 NH1 ARG 136 48.759 56.857 39.205 1.00 0.00 N
ATOM 2090 HH11 ARG 136 49.108 57.517 39.893 1.00 0.00 H
ATOM 2091 HH12 ARG 136 49.314 56.597 38.406 1.00 0.00 H
ATOM 2092 NH2 ARG 136 47.273 55.470 38.366 1.00 0.00 N
ATOM 2093 HH21 ARG 136 46.378 55.004 38.361 1.00 0.00 H
ATOM 2094 HH22 ARG 136 47.905 55.294 37.575 1.00 0.00 H
ATOM 2095 C ARG 136 45.313 58.085 46.375 1.00 0.00 C
ATOM 2096 O ARG 136 46.245 58.612 46.980 1.00 0.00 O
ATOM 2097 N ILE 137 44.038 58.339 46.694 1.00 0.00 N
ATOM 2098 H ILE 137 43.318 57.907 46.124 1.00 0.00 H
ATOM 2099 CA ILE 137 43.606 59.190 47.820 1.00 0.00 C
ATOM 2100 HA ILE 137 44.234 60.079 47.837 1.00 0.00 H
ATOM 2101 CB ILE 137 42.131 59.638 47.646 1.00 0.00 C
ATOM 2102 HB ILE 137 41.529 58.759 47.409 1.00 0.00 H
ATOM 2103 CG2 ILE 137 41.544 60.237 48.939 1.00 0.00 C
ATOM 2104 HG21 ILE 137 41.428 59.463 49.698 1.00 0.00 H
ATOM 2105 HG22 ILE 137 40.554 60.654 48.747 1.00 0.00 H
ATOM 2106 HG23 ILE 137 42.203 61.011 49.330 1.00 0.00 H
ATOM 2107 CG1 ILE 137 41.954 60.648 46.487 1.00 0.00 C
ATOM 2108 HG12 ILE 137 42.098 60.135 45.544 1.00 0.00 H
ATOM 2109 HG13 ILE 137 40.930 61.021 46.496 1.00 0.00 H
ATOM 2110 CD1 ILE 137 42.910 61.843 46.485 1.00 0.00 C
ATOM 2111 HD11 ILE 137 42.993 62.270 47.483 1.00 0.00 H
ATOM 2112 HD12 ILE 137 42.542 62.601 45.793 1.00 0.00 H
ATOM 2113 HD13 ILE 137 43.895 61.518 46.151 1.00 0.00 H
ATOM 2114 C ILE 137 43.837 58.518 49.176 1.00 0.00 C
ATOM 2115 O ILE 137 44.375 59.162 50.077 1.00 0.00 O
ATOM 2116 N GLU 138 43.498 57.234 49.329 1.00 0.00 N
ATOM 2117 H GLU 138 43.014 56.752 48.576 1.00 0.00 H
ATOM 2118 CA GLU 138 43.836 56.480 50.544 1.00 0.00 C
ATOM 2119 HA GLU 138 43.372 56.972 51.400 1.00 0.00 H
ATOM 2120 CB GLU 138 43.273 55.051 50.460 1.00 0.00 C
ATOM 2121 HB2 GLU 138 42.197 55.126 50.309 1.00 0.00 H
ATOM 2122 HB3 GLU 138 43.691 54.539 49.593 1.00 0.00 H
ATOM 2123 CG GLU 138 43.510 54.218 51.734 1.00 0.00 C
ATOM 2124 HG2 GLU 138 43.192 54.803 52.600 1.00 0.00 H
ATOM 2125 HG3 GLU 138 42.878 53.333 51.686 1.00 0.00 H
ATOM 2126 CD GLU 138 44.959 53.751 51.912 1.00 0.00 C
ATOM 2127 OE1 GLU 138 45.510 53.927 53.021 1.00 0.00 O
ATOM 2128 OE2 GLU 138 45.568 53.279 50.934 1.00 0.00 O
ATOM 2129 C GLU 138 45.352 56.504 50.774 1.00 0.00 C
ATOM 2130 O GLU 138 45.796 56.859 51.866 1.00 0.00 O

ATOM	2131	N	THR	139	46.149	56.267	49.725	1.00	0.00	N
ATOM	2132	H	THR	139	45.710	55.929	48.874	1.00	0.00	H
ATOM	2133	CA	THR	139	47.618	56.341	49.784	1.00	0.00	C
ATOM	2134	HA	THR	139	47.974	55.528	50.413	1.00	0.00	H
ATOM	2135	CB	THR	139	48.235	56.134	48.390	1.00	0.00	C
ATOM	2136	HB	THR	139	47.852	56.886	47.705	1.00	0.00	H
ATOM	2137	CG2	THR	139	49.763	56.219	48.399	1.00	0.00	C
ATOM	2138	HG21	THR	139	50.085	57.233	48.636	1.00	0.00	H
ATOM	2139	HG22	THR	139	50.144	55.956	47.412	1.00	0.00	H
ATOM	2140	HG23	THR	139	50.165	55.525	49.137	1.00	0.00	H
ATOM	2141	OG1	THR	139	47.926	54.859	47.881	1.00	0.00	O
ATOM	2142	HG1	THR	139	46.962	54.765	47.818	1.00	0.00	H
ATOM	2143	C	THR	139	48.091	57.673	50.391	1.00	0.00	C
ATOM	2144	O	THR	139	48.907	57.683	51.319	1.00	0.00	O
ATOM	2145	N	ALA	140	47.526	58.799	49.936	1.00	0.00	N
ATOM	2146	H	ALA	140	46.815	58.712	49.218	1.00	0.00	H
ATOM	2147	CA	ALA	140	47.899	60.157	50.347	1.00	0.00	C
ATOM	2148	HA	ALA	140	48.968	60.276	50.166	1.00	0.00	H
ATOM	2149	CB	ALA	140	47.158	61.147	49.436	1.00	0.00	C
ATOM	2150	HB1	ALA	140	47.345	60.905	48.389	1.00	0.00	H
ATOM	2151	HB2	ALA	140	47.512	62.160	49.628	1.00	0.00	H
ATOM	2152	HB3	ALA	140	46.085	61.106	49.626	1.00	0.00	H
ATOM	2153	C	ALA	140	47.662	60.481	51.840	1.00	0.00	C
ATOM	2154	O	ALA	140	48.293	61.402	52.366	1.00	0.00	O
ATOM	2155	N	THR	141	46.836	59.712	52.561	1.00	0.00	N
ATOM	2156	H	THR	141	46.343	58.965	52.080	1.00	0.00	H
ATOM	2157	CA	THR	141	46.618	59.867	54.020	1.00	0.00	C
ATOM	2158	HA	THR	141	46.147	60.828	54.217	1.00	0.00	H
ATOM	2159	CB	THR	141	45.697	58.767	54.556	1.00	0.00	C
ATOM	2160	HB	THR	141	45.613	58.892	55.629	1.00	0.00	H
ATOM	2161	CG2	THR	141	44.281	58.771	53.985	1.00	0.00	C
ATOM	2162	HG21	THR	141	44.295	58.781	52.896	1.00	0.00	H
ATOM	2163	HG22	THR	141	43.751	59.653	54.341	1.00	0.00	H
ATOM	2164	HG23	THR	141	43.747	57.885	54.328	1.00	0.00	H
ATOM	2165	OG1	THR	141	46.290	57.512	54.347	1.00	0.00	O
ATOM	2166	HG1	THR	141	46.031	57.203	53.453	1.00	0.00	H
ATOM	2167	C	THR	141	47.915	59.826	54.842	1.00	0.00	C
ATOM	2168	O	THR	141	48.014	60.483	55.877	1.00	0.00	O
ATOM	2169	N	ARG	142	48.937	59.106	54.355	1.00	0.00	N
ATOM	2170	H	ARG	142	48.753	58.591	53.502	1.00	0.00	H
ATOM	2171	CA	ARG	142	50.286	59.013	54.946	1.00	0.00	C
ATOM	2172	HA	ARG	142	50.207	58.725	55.995	1.00	0.00	H
ATOM	2173	CB	ARG	142	51.081	57.931	54.178	1.00	0.00	C
ATOM	2174	HB2	ARG	142	51.081	58.193	53.118	1.00	0.00	H
ATOM	2175	HB3	ARG	142	52.118	57.929	54.523	1.00	0.00	H
ATOM	2176	CG	ARG	142	50.506	56.508	54.356	1.00	0.00	C
ATOM	2177	HG2	ARG	142	50.900	56.072	55.275	1.00	0.00	H
ATOM	2178	HG3	ARG	142	49.427	56.573	54.470	1.00	0.00	H
ATOM	2179	CD	ARG	142	50.834	55.578	53.166	1.00	0.00	C
ATOM	2180	HD2	ARG	142	50.626	56.098	52.231	1.00	0.00	H
ATOM	2181	HD3	ARG	142	51.900	55.345	53.186	1.00	0.00	H
ATOM	2182	NE	ARG	142	50.067	54.312	53.180	1.00	0.00	N

ATOM	2183	HE	ARG	142	50.574	53.428	53.058	1.00	0.00	H
ATOM	2184	CZ	ARG	142	48.753	54.173	53.116	1.00	0.00	C
ATOM	2185	NH1	ARG	142	47.941	55.165	52.937	1.00	0.00	N
ATOM	2186	HH11	ARG	142	48.274	56.077	52.695	1.00	0.00	H
ATOM	2187	HH12	ARG	142	46.947	54.927	52.892	1.00	0.00	H
ATOM	2188	NH2	ARG	142	48.181	53.025	53.240	1.00	0.00	N
ATOM	2189	HH21	ARG	142	48.745	52.199	53.448	1.00	0.00	H
ATOM	2190	HH22	ARG	142	47.169	52.983	53.130	1.00	0.00	H
ATOM	2191	C	ARG	142	51.025	60.362	54.925	1.00	0.00	C
ATOM	2192	O	ARG	142	51.756	60.674	55.861	1.00	0.00	O
ATOM	2193	N	ILE	143	50.815	61.165	53.878	1.00	0.00	N
ATOM	2194	H	ILE	143	50.132	60.864	53.196	1.00	0.00	H
ATOM	2195	CA	ILE	143	51.453	62.478	53.672	1.00	0.00	C
ATOM	2196	HA	ILE	143	52.462	62.456	54.087	1.00	0.00	H
ATOM	2197	CB	ILE	143	51.561	62.787	52.154	1.00	0.00	C
ATOM	2198	HB	ILE	143	50.562	62.721	51.721	1.00	0.00	H
ATOM	2199	CG2	ILE	143	52.086	64.214	51.899	1.00	0.00	C
ATOM	2200	HG21	ILE	143	51.404	64.957	52.316	1.00	0.00	H
ATOM	2201	HG22	ILE	143	52.153	64.416	50.832	1.00	0.00	H
ATOM	2202	HG23	ILE	143	53.071	64.340	52.351	1.00	0.00	H
ATOM	2203	CG1	ILE	143	52.464	61.746	51.446	1.00	0.00	C
ATOM	2204	HG12	ILE	143	53.496	61.874	51.777	1.00	0.00	H
ATOM	2205	HG13	ILE	143	52.149	60.741	51.724	1.00	0.00	H
ATOM	2206	CD1	ILE	143	52.417	61.813	49.913	1.00	0.00	C
ATOM	2207	HD11	ILE	143	51.384	61.758	49.568	1.00	0.00	H
ATOM	2208	HD12	ILE	143	52.973	60.971	49.500	1.00	0.00	H
ATOM	2209	HD13	ILE	143	52.876	62.734	49.553	1.00	0.00	H
ATOM	2210	C	ILE	143	50.688	63.583	54.412	1.00	0.00	C
ATOM	2211	O	ILE	143	51.287	64.413	55.089	1.00	0.00	O
ATOM	2212	N	PHE	144	49.356	63.591	54.301	1.00	0.00	N
ATOM	2213	H	PHE	144	48.926	62.889	53.711	1.00	0.00	H
ATOM	2214	CA	PHE	144	48.500	64.653	54.852	1.00	0.00	C
ATOM	2215	HA	PHE	144	49.111	65.552	54.952	1.00	0.00	H
ATOM	2216	CB	PHE	144	47.404	64.989	53.832	1.00	0.00	C
ATOM	2217	HB2	PHE	144	46.820	64.094	53.625	1.00	0.00	H
ATOM	2218	HB3	PHE	144	46.733	65.739	54.248	1.00	0.00	H
ATOM	2219	CG	PHE	144	47.980	65.533	52.536	1.00	0.00	C
ATOM	2220	CD1	PHE	144	48.429	66.866	52.474	1.00	0.00	C
ATOM	2221	HD1	PHE	144	48.311	67.521	53.326	1.00	0.00	H
ATOM	2222	CE1	PHE	144	49.051	67.349	51.309	1.00	0.00	C
ATOM	2223	HE1	PHE	144	49.396	68.373	51.264	1.00	0.00	H
ATOM	2224	CZ	PHE	144	49.232	66.499	50.205	1.00	0.00	C
ATOM	2225	HZ	PHE	144	49.717	66.873	49.313	1.00	0.00	H
ATOM	2226	CE2	PHE	144	48.774	65.171	50.258	1.00	0.00	C
ATOM	2227	HE2	PHE	144	48.908	64.519	49.407	1.00	0.00	H
ATOM	2228	CD2	PHE	144	48.141	64.693	51.419	1.00	0.00	C
ATOM	2229	HD2	PHE	144	47.792	63.674	51.457	1.00	0.00	H
ATOM	2230	C	PHE	144	47.990	64.372	56.279	1.00	0.00	C
ATOM	2231	O	PHE	144	47.099	65.065	56.765	1.00	0.00	O
ATOM	2232	N	ASN	145	48.572	63.379	56.958	1.00	0.00	N
ATOM	2233	H	ASN	145	49.282	62.865	56.455	1.00	0.00	H
ATOM	2234	CA	ASN	145	48.371	62.968	58.355	1.00	0.00	C

ATOM	2235	HA	ASN	145	48.979	62.071	58.485	1.00	0.00	H
ATOM	2236	CB	ASN	145	48.962	64.026	59.307	1.00	0.00	C
ATOM	2237	HB2	ASN	145	48.479	64.986	59.126	1.00	0.00	H
ATOM	2238	HB3	ASN	145	48.751	63.740	60.337	1.00	0.00	H
ATOM	2239	CG	ASN	145	50.472	64.183	59.192	1.00	0.00	C
ATOM	2240	OD1	ASN	145	51.209	63.223	59.007	1.00	0.00	O
ATOM	2241	ND2	ASN	145	50.965	65.390	59.333	1.00	0.00	N
ATOM	2242	HD21	ASN	145	50.360	66.191	59.443	1.00	0.00	H
ATOM	2243	HD22	ASN	145	51.975	65.507	59.374	1.00	0.00	H
ATOM	2244	C	ASN	145	46.956	62.518	58.784	1.00	0.00	C
ATOM	2245	O	ASN	145	46.870	61.682	59.686	1.00	0.00	O
ATOM	2246	N	SER	146	45.863	62.973	58.158	1.00	0.00	N
ATOM	2247	H	SER	146	45.990	63.694	57.459	1.00	0.00	H
ATOM	2248	CA	SER	146	44.501	62.491	58.459	1.00	0.00	C
ATOM	2249	HA	SER	146	44.313	62.657	59.521	1.00	0.00	H
ATOM	2250	CB	SER	146	43.432	63.261	57.678	1.00	0.00	C
ATOM	2251	HB2	SER	146	43.489	64.326	57.909	1.00	0.00	H
ATOM	2252	HB3	SER	146	43.589	63.109	56.610	1.00	0.00	H
ATOM	2253	OG	SER	146	42.163	62.754	58.050	1.00	0.00	O
ATOM	2254	HG	SER	146	41.552	62.797	57.288	1.00	0.00	H
ATOM	2255	C	SER	146	44.340	60.994	58.173	1.00	0.00	C
ATOM	2256	O	SER	146	44.927	60.465	57.228	1.00	0.00	O
ATOM	2257	N	LYS	147	43.529	60.295	58.976	1.00	0.00	N
ATOM	2258	H	LYS	147	43.053	60.806	59.707	1.00	0.00	H
ATOM	2259	CA	LYS	147	43.227	58.861	58.796	1.00	0.00	C
ATOM	2260	HA	LYS	147	44.076	58.393	58.301	1.00	0.00	H
ATOM	2261	CB	LYS	147	43.069	58.171	60.165	1.00	0.00	C
ATOM	2262	HB2	LYS	147	42.264	58.636	60.736	1.00	0.00	H
ATOM	2263	HB3	LYS	147	42.835	57.116	60.012	1.00	0.00	H
ATOM	2264	CG	LYS	147	44.397	58.286	60.928	1.00	0.00	C
ATOM	2265	HG2	LYS	147	45.191	57.937	60.270	1.00	0.00	H
ATOM	2266	HG3	LYS	147	44.583	59.335	61.166	1.00	0.00	H
ATOM	2267	CD	LYS	147	44.489	57.477	62.221	1.00	0.00	C
ATOM	2268	HD2	LYS	147	43.708	57.777	62.922	1.00	0.00	H
ATOM	2269	HD3	LYS	147	44.403	56.412	61.994	1.00	0.00	H
ATOM	2270	CE	LYS	147	45.875	57.794	62.790	1.00	0.00	C
ATOM	2271	HE2	LYS	147	46.607	57.685	61.986	1.00	0.00	H
ATOM	2272	HE3	LYS	147	45.911	58.836	63.121	1.00	0.00	H
ATOM	2273	NZ	LYS	147	46.280	56.898	63.888	1.00	0.00	N
ATOM	2274	HZ1	LYS	147	46.227	55.920	63.587	1.00	0.00	H
ATOM	2275	HZ2	LYS	147	45.726	57.009	64.719	1.00	0.00	H
ATOM	2276	HZ3	LYS	147	47.268	57.052	64.086	1.00	0.00	H
ATOM	2277	C	LYS	147	42.058	58.591	57.845	1.00	0.00	C
ATOM	2278	O	LYS	147	41.933	57.469	57.364	1.00	0.00	O
ATOM	2279	N	VAL	148	41.270	59.618	57.517	1.00	0.00	N
ATOM	2280	H	VAL	148	41.475	60.516	57.932	1.00	0.00	H
ATOM	2281	CA	VAL	148	40.226	59.590	56.479	1.00	0.00	C
ATOM	2282	HA	VAL	148	40.360	58.700	55.867	1.00	0.00	H
ATOM	2283	CB	VAL	148	38.803	59.536	57.080	1.00	0.00	C
ATOM	2284	HB	VAL	148	38.603	60.479	57.592	1.00	0.00	H
ATOM	2285	CG1	VAL	148	37.743	59.354	55.983	1.00	0.00	C
ATOM	2286	HG11	VAL	148	37.928	58.433	55.429	1.00	0.00	H

ATOM	2287	HG12	VAL	148	36.751	59.305	56.433	1.00	0.00	H
ATOM	2288	HG13	VAL	148	37.758	60.198	55.296	1.00	0.00	H
ATOM	2289	CG2	VAL	148	38.625	58.405	58.104	1.00	0.00	C
ATOM	2290	HG21	VAL	148	38.871	57.444	57.657	1.00	0.00	H
ATOM	2291	HG22	VAL	148	37.592	58.380	58.454	1.00	0.00	H
ATOM	2292	HG23	VAL	148	39.268	58.573	58.967	1.00	0.00	H
ATOM	2293	C	VAL	148	40.388	60.820	55.584	1.00	0.00	C
ATOM	2294	O	VAL	148	40.493	61.937	56.096	1.00	0.00	O
ATOM	2295	N	LEU	149	40.407	60.634	54.262	1.00	0.00	N
ATOM	2296	H	LEU	149	40.383	59.684	53.904	1.00	0.00	H
ATOM	2297	CA	LEU	149	40.404	61.730	53.283	1.00	0.00	C
ATOM	2298	HA	LEU	149	40.096	62.647	53.785	1.00	0.00	H
ATOM	2299	CB	LEU	149	41.811	61.947	52.696	1.00	0.00	C
ATOM	2300	HB2	LEU	149	42.180	60.987	52.330	1.00	0.00	H
ATOM	2301	HB3	LEU	149	41.734	62.618	51.840	1.00	0.00	H
ATOM	2302	CG	LEU	149	42.833	62.541	53.685	1.00	0.00	C
ATOM	2303	HG	LEU	149	42.864	61.933	54.588	1.00	0.00	H
ATOM	2304	CD1	LEU	149	44.230	62.530	53.068	1.00	0.00	C
ATOM	2305	HD11	LEU	149	44.456	61.537	52.678	1.00	0.00	H
ATOM	2306	HD12	LEU	149	44.958	62.787	53.835	1.00	0.00	H
ATOM	2307	HD13	LEU	149	44.293	63.252	52.256	1.00	0.00	H
ATOM	2308	CD2	LEU	149	42.511	63.991	54.064	1.00	0.00	C
ATOM	2309	HD21	LEU	149	42.392	64.597	53.166	1.00	0.00	H
ATOM	2310	HD22	LEU	149	41.587	64.030	54.641	1.00	0.00	H
ATOM	2311	HD23	LEU	149	43.313	64.407	54.671	1.00	0.00	H
ATOM	2312	C	LEU	149	39.372	61.476	52.182	1.00	0.00	C
ATOM	2313	O	LEU	149	39.257	60.363	51.666	1.00	0.00	O
ATOM	2314	N	ILE	150	38.622	62.517	51.815	1.00	0.00	N
ATOM	2315	H	ILE	150	38.761	63.403	52.294	1.00	0.00	H
ATOM	2316	CA	ILE	150	37.519	62.417	50.850	1.00	0.00	C
ATOM	2317	HA	ILE	150	37.606	61.457	50.341	1.00	0.00	H
ATOM	2318	CB	ILE	150	36.138	62.400	51.554	1.00	0.00	C
ATOM	2319	HB	ILE	150	35.424	62.045	50.811	1.00	0.00	H
ATOM	2320	CG2	ILE	150	36.122	61.388	52.717	1.00	0.00	C
ATOM	2321	HG21	ILE	150	36.618	60.465	52.422	1.00	0.00	H
ATOM	2322	HG22	ILE	150	35.097	61.150	52.998	1.00	0.00	H
ATOM	2323	HG23	ILE	150	36.639	61.801	53.585	1.00	0.00	H
ATOM	2324	CG1	ILE	150	35.656	63.790	52.032	1.00	0.00	C
ATOM	2325	HG12	ILE	150	36.304	64.139	52.838	1.00	0.00	H
ATOM	2326	HG13	ILE	150	35.716	64.504	51.213	1.00	0.00	H
ATOM	2327	CD1	ILE	150	34.202	63.802	52.519	1.00	0.00	C
ATOM	2328	HD11	ILE	150	33.530	63.563	51.695	1.00	0.00	H
ATOM	2329	HD12	ILE	150	33.957	64.792	52.902	1.00	0.00	H
ATOM	2330	HD13	ILE	150	34.058	63.082	53.319	1.00	0.00	H
ATOM	2331	C	ILE	150	37.638	63.483	49.758	1.00	0.00	C
ATOM	2332	O	ILE	150	38.004	64.632	50.020	1.00	0.00	O
ATOM	2333	N	HIE	151	37.343	63.096	48.516	1.00	0.00	N
ATOM	2334	H	HIE	151	37.086	62.126	48.368	1.00	0.00	H
ATOM	2335	CA	HIE	151	37.336	64.004	47.363	1.00	0.00	C
ATOM	2336	HA	HIE	151	38.239	64.616	47.393	1.00	0.00	H
ATOM	2337	CB	HIE	151	37.377	63.157	46.084	1.00	0.00	C
ATOM	2338	HB2	HIE	151	38.309	62.589	46.068	1.00	0.00	H

```
ATOM   2339  HB3 HIE   151      36.549  62.447  46.105  1.00  0.00           H
ATOM   2340  CG  HIE   151      37.276  63.959  44.813  1.00  0.00           C
ATOM   2341  ND1 HIE   151      36.122  64.095  44.043  1.00  0.00           N
ATOM   2342  CE1 HIE   151      36.429  64.949  43.054  1.00  0.00           C
ATOM   2343  HE1 HIE   151      35.748  65.253  42.266  1.00  0.00           H
ATOM   2344  NE2 HIE   151      37.695  65.379  43.191  1.00  0.00           N
ATOM   2345  HE2 HIE   151      38.153  66.037  42.573  1.00  0.00           H
ATOM   2346  CD2 HIE   151      38.244  64.771  44.301  1.00  0.00           C
ATOM   2347  HD2 HIE   151      39.231  64.926  44.719  1.00  0.00           H
ATOM   2348  C   HIE   151      36.143  64.976  47.383  1.00  0.00           C
ATOM   2349  O   HIE   151      35.111  64.698  47.991  1.00  0.00           O
ATOM   2350  N   SER   152      36.261  66.102  46.670  1.00  0.00           N
ATOM   2351  H   SER   152      37.155  66.282  46.231  1.00  0.00           H
ATOM   2352  CA  SER   152      35.228  67.150  46.578  1.00  0.00           C
ATOM   2353  HA  SER   152      35.128  67.603  47.564  1.00  0.00           H
ATOM   2354  CB  SER   152      35.694  68.227  45.588  1.00  0.00           C
ATOM   2355  HB2 SER   152      36.781  68.208  45.536  1.00  0.00           H
ATOM   2356  HB3 SER   152      35.306  68.016  44.590  1.00  0.00           H
ATOM   2357  OG  SER   152      35.292  69.522  46.001  1.00  0.00           O
ATOM   2358  HG  SER   152      34.391  69.683  45.629  1.00  0.00           H
ATOM   2359  C   SER   152      33.832  66.640  46.192  1.00  0.00           C
ATOM   2360  O   SER   152      32.844  67.129  46.734  1.00  0.00           O
ATOM   2361  N   HID   153      33.723  65.604  45.349  1.00  0.00           N
ATOM   2362  H   HID   153      34.567  65.203  44.955  1.00  0.00           H
ATOM   2363  CA  HID   153      32.444  64.939  45.055  1.00  0.00           C
ATOM   2364  HA  HID   153      31.754  65.651  44.600  1.00  0.00           H
ATOM   2365  CB  HID   153      32.690  63.777  44.080  1.00  0.00           C
ATOM   2366  HB2 HID   153      33.496  63.148  44.462  1.00  0.00           H
ATOM   2367  HB3 HID   153      31.793  63.161  44.048  1.00  0.00           H
ATOM   2368  CG  HID   153      33.011  64.171  42.660  1.00  0.00           C
ATOM   2369  ND1 HID   153      33.359  63.296  41.656  1.00  0.00           N
ATOM   2370  HD1 HID   153      33.525  62.296  41.761  1.00  0.00           H
ATOM   2371  CE1 HID   153      33.416  63.977  40.502  1.00  0.00           C
ATOM   2372  HE1 HID   153      33.655  63.549  39.536  1.00  0.00           H
ATOM   2373  NE2 HID   153      33.109  65.266  40.712  1.00  0.00           N
ATOM   2374  CD2 HID   153      32.860  65.404  42.080  1.00  0.00           C
ATOM   2375  HD2 HID   153      32.538  66.312  42.572  1.00  0.00           H
ATOM   2376  C   HID   153      31.754  64.396  46.315  1.00  0.00           C
ATOM   2377  O   HID   153      30.554  64.587  46.502  1.00  0.00           O
ATOM   2378  N   CYS   154      32.514  63.762  47.208  1.00  0.00           N
ATOM   2379  H   CYS   154      33.511  63.711  47.050  1.00  0.00           H
ATOM   2380  CA  CYS   154      32.009  63.179  48.451  1.00  0.00           C
ATOM   2381  HA  CYS   154      31.146  62.557  48.217  1.00  0.00           H
ATOM   2382  CB  CYS   154      33.106  62.289  49.047  1.00  0.00           C
ATOM   2383  HB2 CYS   154      33.945  62.918  49.347  1.00  0.00           H
ATOM   2384  HB3 CYS   154      32.719  61.771  49.928  1.00  0.00           H
ATOM   2385  SG  CYS   154      33.668  61.079  47.814  1.00  0.00           S
ATOM   2386  HG  CYS   154      34.795  60.701  48.440  1.00  0.00           H
ATOM   2387  C   CYS   154      31.550  64.241  49.467  1.00  0.00           C
ATOM   2388  O   CYS   154      30.771  63.929  50.363  1.00  0.00           O
ATOM   2389  N   VAL   155      32.004  65.491  49.328  1.00  0.00           N
ATOM   2390  H   VAL   155      32.679  65.668  48.595  1.00  0.00           H
```

ATOM	2391	CA	VAL	155	31.462	66.642	50.070	1.00	0.00	C
ATOM	2392	HA	VAL	155	31.132	66.311	51.056	1.00	0.00	H
ATOM	2393	CB	VAL	155	32.540	67.729	50.274	1.00	0.00	C
ATOM	2394	HB	VAL	155	32.943	68.023	49.304	1.00	0.00	H
ATOM	2395	CG1	VAL	155	31.992	68.987	50.962	1.00	0.00	C
ATOM	2396	HG11	VAL	155	31.249	69.474	50.333	1.00	0.00	H
ATOM	2397	HG12	VAL	155	31.538	68.722	51.918	1.00	0.00	H
ATOM	2398	HG13	VAL	155	32.803	69.696	51.137	1.00	0.00	H
ATOM	2399	CG2	VAL	155	33.691	67.199	51.132	1.00	0.00	C
ATOM	2400	HG21	VAL	155	34.181	66.363	50.634	1.00	0.00	H
ATOM	2401	HG22	VAL	155	33.314	66.879	52.104	1.00	0.00	H
ATOM	2402	HG23	VAL	155	34.425	67.987	51.282	1.00	0.00	H
ATOM	2403	C	VAL	155	30.236	67.224	49.363	1.00	0.00	C
ATOM	2404	O	VAL	155	29.194	67.422	49.977	1.00	0.00	O
ATOM	2405	N	GLN	156	30.341	67.525	48.068	1.00	0.00	N
ATOM	2406	H	GLN	156	31.214	67.319	47.595	1.00	0.00	H
ATOM	2407	CA	GLN	156	29.351	68.344	47.356	1.00	0.00	C
ATOM	2408	HA	GLN	156	29.024	69.141	48.026	1.00	0.00	H
ATOM	2409	CB	GLN	156	30.025	69.006	46.143	1.00	0.00	C
ATOM	2410	HB2	GLN	156	30.497	68.238	45.527	1.00	0.00	H
ATOM	2411	HB3	GLN	156	29.270	69.515	45.542	1.00	0.00	H
ATOM	2412	CG	GLN	156	31.077	70.041	46.585	1.00	0.00	C
ATOM	2413	HG2	GLN	156	30.576	70.874	47.079	1.00	0.00	H
ATOM	2414	HG3	GLN	156	31.771	69.596	47.297	1.00	0.00	H
ATOM	2415	CD	GLN	156	31.894	70.560	45.411	1.00	0.00	C
ATOM	2416	OE1	GLN	156	32.901	69.983	45.025	1.00	0.00	O
ATOM	2417	NE2	GLN	156	31.515	71.669	44.820	1.00	0.00	N
ATOM	2418	HE21	GLN	156	32.054	71.987	44.036	1.00	0.00	H
ATOM	2419	HE22	GLN	156	30.725	72.198	45.180	1.00	0.00	H
ATOM	2420	C	GLN	156	28.076	67.583	46.957	1.00	0.00	C
ATOM	2421	O	GLN	156	27.015	68.202	46.833	1.00	0.00	O
ATOM	2422	N	TYR	157	28.142	66.259	46.784	1.00	0.00	N
ATOM	2423	H	TYR	157	29.050	65.805	46.829	1.00	0.00	H
ATOM	2424	CA	TYR	157	26.970	65.406	46.535	1.00	0.00	C
ATOM	2425	HA	TYR	157	26.208	65.998	46.030	1.00	0.00	H
ATOM	2426	CB	TYR	157	27.356	64.263	45.578	1.00	0.00	C
ATOM	2427	HB2	TYR	157	28.018	63.582	46.115	1.00	0.00	H
ATOM	2428	HB3	TYR	157	26.454	63.705	45.324	1.00	0.00	H
ATOM	2429	CG	TYR	157	28.034	64.673	44.270	1.00	0.00	C
ATOM	2430	CD1	TYR	157	27.718	65.890	43.625	1.00	0.00	C
ATOM	2431	HD1	TYR	157	26.973	66.555	44.035	1.00	0.00	H
ATOM	2432	CE1	TYR	157	28.380	66.258	42.437	1.00	0.00	C
ATOM	2433	HE1	TYR	157	28.152	67.191	41.945	1.00	0.00	H
ATOM	2434	CZ	TYR	157	29.350	65.407	41.870	1.00	0.00	C
ATOM	2435	OH	TYR	157	30.003	65.792	40.742	1.00	0.00	O
ATOM	2436	HH	TYR	157	30.892	65.425	40.661	1.00	0.00	H
ATOM	2437	CE2	TYR	157	29.636	64.171	42.487	1.00	0.00	C
ATOM	2438	HE2	TYR	157	30.352	63.504	42.033	1.00	0.00	H
ATOM	2439	CD2	TYR	157	28.983	63.811	43.684	1.00	0.00	C
ATOM	2440	HD2	TYR	157	29.211	62.867	44.160	1.00	0.00	H
ATOM	2441	C	TYR	157	26.295	64.905	47.830	1.00	0.00	C
ATOM	2442	O	TYR	157	25.299	64.187	47.772	1.00	0.00	O

ATOM	2443	N	LEU	158	26.807	65.293	49.004	1.00	0.00	N
ATOM	2444	H	LEU	158	27.598	65.922	48.998	1.00	0.00	H
ATOM	2445	CA	LEU	158	26.242	64.954	50.309	1.00	0.00	C
ATOM	2446	HA	LEU	158	25.896	63.922	50.255	1.00	0.00	H
ATOM	2447	CB	LEU	158	27.381	65.045	51.335	1.00	0.00	C
ATOM	2448	HB2	LEU	158	28.251	64.514	50.947	1.00	0.00	H
ATOM	2449	HB3	LEU	158	27.649	66.096	51.443	1.00	0.00	H
ATOM	2450	CG	LEU	158	27.064	64.498	52.732	1.00	0.00	C
ATOM	2451	HG	LEU	158	26.195	65.007	53.141	1.00	0.00	H
ATOM	2452	CD1	LEU	158	26.815	62.989	52.724	1.00	0.00	C
ATOM	2453	HD11	LEU	158	25.931	62.750	52.140	1.00	0.00	H
ATOM	2454	HD12	LEU	158	26.672	62.648	53.748	1.00	0.00	H
ATOM	2455	HD13	LEU	158	27.679	62.481	52.296	1.00	0.00	H
ATOM	2456	CD2	LEU	158	28.244	64.780	53.644	1.00	0.00	C
ATOM	2457	HD21	LEU	158	29.144	64.307	53.252	1.00	0.00	H
ATOM	2458	HD22	LEU	158	28.404	65.857	53.685	1.00	0.00	H
ATOM	2459	HD23	LEU	158	28.036	64.414	54.648	1.00	0.00	H
ATOM	2460	C	LEU	158	25.040	65.846	50.692	1.00	0.00	C
ATOM	2461	O	LEU	158	24.957	67.017	50.313	1.00	0.00	O
ATOM	2462	N	GLY	159	24.117	65.288	51.484	1.00	0.00	N
ATOM	2463	H	GLY	159	24.239	64.319	51.722	1.00	0.00	H
ATOM	2464	CA	GLY	159	22.992	66.011	52.099	1.00	0.00	C
ATOM	2465	HA2	GLY	159	22.772	66.911	51.521	1.00	0.00	H
ATOM	2466	HA3	GLY	159	22.105	65.379	52.062	1.00	0.00	H
ATOM	2467	C	GLY	159	23.222	66.430	53.566	1.00	0.00	C
ATOM	2468	O	GLY	159	23.011	67.604	53.878	1.00	0.00	O
ATOM	2469	N	PRO	160	23.625	65.506	54.471	1.00	0.00	N
ATOM	2470	CD	PRO	160	23.541	64.061	54.289	1.00	0.00	C
ATOM	2471	HD2	PRO	160	24.482	63.686	53.895	1.00	0.00	H
ATOM	2472	HD3	PRO	160	22.718	63.781	53.631	1.00	0.00	H
ATOM	2473	CG	PRO	160	23.306	63.477	55.681	1.00	0.00	C
ATOM	2474	HG2	PRO	160	23.676	62.457	55.765	1.00	0.00	H
ATOM	2475	HG3	PRO	160	22.245	63.527	55.931	1.00	0.00	H
ATOM	2476	CB	PRO	160	24.094	64.438	56.564	1.00	0.00	C
ATOM	2477	HB2	PRO	160	25.159	64.205	56.503	1.00	0.00	H
ATOM	2478	HB3	PRO	160	23.751	64.411	57.599	1.00	0.00	H
ATOM	2479	CA	PRO	160	23.808	65.788	55.901	1.00	0.00	C
ATOM	2480	HA	PRO	160	22.848	66.146	56.275	1.00	0.00	H
ATOM	2481	C	PRO	160	24.874	66.831	56.279	1.00	0.00	C
ATOM	2482	O	PRO	160	25.875	67.034	55.593	1.00	0.00	O
ATOM	2483	N	LYS	161	24.641	67.462	57.435	1.00	0.00	N
ATOM	2484	H	LYS	161	23.850	67.131	57.965	1.00	0.00	H
ATOM	2485	CA	LYS	161	25.322	68.649	57.982	1.00	0.00	C
ATOM	2486	HA	LYS	161	25.545	69.337	57.164	1.00	0.00	H
ATOM	2487	CB	LYS	161	24.342	69.347	58.961	1.00	0.00	C
ATOM	2488	HB2	LYS	161	24.127	68.656	59.779	1.00	0.00	H
ATOM	2489	HB3	LYS	161	24.830	70.226	59.388	1.00	0.00	H
ATOM	2490	CG	LYS	161	23.008	69.799	58.327	1.00	0.00	C
ATOM	2491	HG2	LYS	161	23.196	70.652	57.673	1.00	0.00	H
ATOM	2492	HG3	LYS	161	22.587	68.992	57.726	1.00	0.00	H
ATOM	2493	CD	LYS	161	21.963	70.183	59.391	1.00	0.00	C
ATOM	2494	HD2	LYS	161	21.777	69.320	60.033	1.00	0.00	H

```
ATOM   2495  HD3 LYS   161      22.344  71.004  60.001  1.00  0.00           H
ATOM   2496  CE  LYS   161      20.649  70.607  58.717  1.00  0.00           C
ATOM   2497  HE2 LYS   161      20.814  71.551  58.188  1.00  0.00           H
ATOM   2498  HE3 LYS   161      20.375  69.861  57.965  1.00  0.00           H
ATOM   2499  NZ  LYS   161      19.532  70.762  59.682  1.00  0.00           N
ATOM   2500  HZ1 LYS   161      19.736  71.395  60.448  1.00  0.00           H
ATOM   2501  HZ2 LYS   161      19.226  69.887  60.106  1.00  0.00           H
ATOM   2502  HZ3 LYS   161      18.704  71.150  59.238  1.00  0.00           H
ATOM   2503  C   LYS   161      26.675  68.318  58.650  1.00  0.00           C
ATOM   2504  O   LYS   161      26.861  68.588  59.836  1.00  0.00           O
ATOM   2505  N   ILE   162      27.591  67.655  57.940  1.00  0.00           N
ATOM   2506  H   ILE   162      27.367  67.427  56.980  1.00  0.00           H
ATOM   2507  CA  ILE   162      28.894  67.232  58.498  1.00  0.00           C
ATOM   2508  HA  ILE   162      28.701  66.819  59.489  1.00  0.00           H
ATOM   2509  CB  ILE   162      29.537  66.100  57.668  1.00  0.00           C
ATOM   2510  HB  ILE   162      30.452  65.807  58.186  1.00  0.00           H
ATOM   2511  CG2 ILE   162      28.611  64.870  57.651  1.00  0.00           C
ATOM   2512 HG21 ILE   162      28.310  64.620  58.670  1.00  0.00           H
ATOM   2513 HG22 ILE   162      29.132  64.009  57.234  1.00  0.00           H
ATOM   2514 HG23 ILE   162      27.719  65.071  57.058  1.00  0.00           H
ATOM   2515  CG1 ILE   162      29.930  66.559  56.247  1.00  0.00           C
ATOM   2516 HG12 ILE   162      29.028  66.716  55.657  1.00  0.00           H
ATOM   2517 HG13 ILE   162      30.460  67.508  56.294  1.00  0.00           H
ATOM   2518  CD1 ILE   162      30.858  65.573  55.531  1.00  0.00           C
ATOM   2519 HD11 ILE   162      31.781  65.475  56.097  1.00  0.00           H
ATOM   2520 HD12 ILE   162      31.091  65.949  54.535  1.00  0.00           H
ATOM   2521 HD13 ILE   162      30.391  64.595  55.441  1.00  0.00           H
ATOM   2522  C   ILE   162      29.870  68.406  58.704  1.00  0.00           C
ATOM   2523  O   ILE   162      29.867  69.376  57.949  1.00  0.00           O
ATOM   2524  N   LYS   163      30.761  68.286  59.697  1.00  0.00           N
ATOM   2525  H   LYS   163      30.701  67.449  60.260  1.00  0.00           H
ATOM   2526  CA  LYS   163      31.715  69.328  60.148  1.00  0.00           C
ATOM   2527  HA  LYS   163      31.183  70.283  60.165  1.00  0.00           H
ATOM   2528  CB  LYS   163      32.161  69.011  61.594  1.00  0.00           C
ATOM   2529  HB2 LYS   163      32.728  68.078  61.601  1.00  0.00           H
ATOM   2530  HB3 LYS   163      32.817  69.810  61.944  1.00  0.00           H
ATOM   2531  CG  LYS   163      30.985  68.900  62.580  1.00  0.00           C
ATOM   2532  HG2 LYS   163      30.392  69.814  62.526  1.00  0.00           H
ATOM   2533  HG3 LYS   163      30.352  68.054  62.307  1.00  0.00           H
ATOM   2534  CD  LYS   163      31.472  68.703  64.023  1.00  0.00           C
ATOM   2535  HD2 LYS   163      32.047  67.777  64.096  1.00  0.00           H
ATOM   2536  HD3 LYS   163      32.103  69.547  64.307  1.00  0.00           H
ATOM   2537  CE  LYS   163      30.253  68.637  64.948  1.00  0.00           C
ATOM   2538  HE2 LYS   163      29.640  69.525  64.765  1.00  0.00           H
ATOM   2539  HE3 LYS   163      29.651  67.760  64.690  1.00  0.00           H
ATOM   2540  NZ  LYS   163      30.631  68.591  66.379  1.00  0.00           N
ATOM   2541  HZ1 LYS   163      31.064  67.714  66.654  1.00  0.00           H
ATOM   2542  HZ2 LYS   163      29.798  68.688  66.952  1.00  0.00           H
ATOM   2543  HZ3 LYS   163      31.251  69.354  66.640  1.00  0.00           H
ATOM   2544  C   LYS   163      32.934  69.565  59.226  1.00  0.00           C
ATOM   2545  O   LYS   163      33.953  70.085  59.684  1.00  0.00           O
ATOM   2546  N   ALA   164      32.854  69.158  57.957  1.00  0.00           N
```

ATOM	2547	H	ALA	164	31.939	68.903	57.621	1.00	0.00	H
ATOM	2548	CA	ALA	164	34.003	68.927	57.073	1.00	0.00	C
ATOM	2549	HA	ALA	164	34.578	68.111	57.513	1.00	0.00	H
ATOM	2550	CB	ALA	164	33.491	68.451	55.709	1.00	0.00	C
ATOM	2551	HB1	ALA	164	32.900	67.547	55.832	1.00	0.00	H
ATOM	2552	HB2	ALA	164	34.337	68.224	55.058	1.00	0.00	H
ATOM	2553	HB3	ALA	164	32.880	69.228	55.246	1.00	0.00	H
ATOM	2554	C	ALA	164	34.965	70.123	56.915	1.00	0.00	C
ATOM	2555	O	ALA	164	34.551	71.286	56.885	1.00	0.00	O
ATOM	2556	N	ARG	165	36.258	69.821	56.752	1.00	0.00	N
ATOM	2557	H	ARG	165	36.497	68.830	56.739	1.00	0.00	H
ATOM	2558	CA	ARG	165	37.370	70.785	56.688	1.00	0.00	C
ATOM	2559	HA	ARG	165	36.966	71.795	56.610	1.00	0.00	H
ATOM	2560	CB	ARG	165	38.148	70.687	58.019	1.00	0.00	C
ATOM	2561	HB2	ARG	165	37.465	70.938	58.833	1.00	0.00	H
ATOM	2562	HB3	ARG	165	38.475	69.655	58.164	1.00	0.00	H
ATOM	2563	CG	ARG	165	39.377	71.603	58.115	1.00	0.00	C
ATOM	2564	HG2	ARG	165	40.105	71.287	57.367	1.00	0.00	H
ATOM	2565	HG3	ARG	165	39.086	72.634	57.910	1.00	0.00	H
ATOM	2566	CD	ARG	165	40.015	71.527	59.516	1.00	0.00	C
ATOM	2567	HD2	ARG	165	39.358	72.027	60.230	1.00	0.00	H
ATOM	2568	HD3	ARG	165	40.097	70.482	59.822	1.00	0.00	H
ATOM	2569	NE	ARG	165	41.352	72.159	59.547	1.00	0.00	N
ATOM	2570	HE	ARG	165	41.428	73.088	59.941	1.00	0.00	H
ATOM	2571	CZ	ARG	165	42.446	71.643	59.010	1.00	0.00	C
ATOM	2572	NH1	ARG	165	42.504	70.421	58.570	1.00	0.00	N
ATOM	2573	HH11	ARG	165	41.769	69.744	58.814	1.00	0.00	H
ATOM	2574	HH12	ARG	165	43.317	70.072	58.102	1.00	0.00	H
ATOM	2575	NH2	ARG	165	43.526	72.358	58.892	1.00	0.00	N
ATOM	2576	HH21	ARG	165	43.589	73.279	59.302	1.00	0.00	H
ATOM	2577	HH22	ARG	165	44.317	72.011	58.365	1.00	0.00	H
ATOM	2578	C	ARG	165	38.232	70.525	55.447	1.00	0.00	C
ATOM	2579	O	ARG	165	38.640	69.396	55.196	1.00	0.00	O
ATOM	2580	N	PHE	166	38.501	71.554	54.644	1.00	0.00	N
ATOM	2581	H	PHE	166	38.139	72.463	54.882	1.00	0.00	H
ATOM	2582	CA	PHE	166	39.387	71.445	53.476	1.00	0.00	C
ATOM	2583	HA	PHE	166	39.088	70.578	52.884	1.00	0.00	H
ATOM	2584	CB	PHE	166	39.215	72.691	52.600	1.00	0.00	C
ATOM	2585	HB2	PHE	166	38.200	72.699	52.201	1.00	0.00	H
ATOM	2586	HB3	PHE	166	39.328	73.577	53.226	1.00	0.00	H
ATOM	2587	CG	PHE	166	40.189	72.796	51.442	1.00	0.00	C
ATOM	2588	CD1	PHE	166	39.956	72.087	50.249	1.00	0.00	C
ATOM	2589	HD1	PHE	166	39.090	71.446	50.159	1.00	0.00	H
ATOM	2590	CE1	PHE	166	40.846	72.220	49.168	1.00	0.00	C
ATOM	2591	HE1	PHE	166	40.669	71.685	48.247	1.00	0.00	H
ATOM	2592	CZ	PHE	166	41.974	73.051	49.281	1.00	0.00	C
ATOM	2593	HZ	PHE	166	42.653	73.156	48.448	1.00	0.00	H
ATOM	2594	CE2	PHE	166	42.227	73.731	50.484	1.00	0.00	C
ATOM	2595	HE2	PHE	166	43.107	74.349	50.585	1.00	0.00	H
ATOM	2596	CD2	PHE	166	41.333	73.608	51.561	1.00	0.00	C
ATOM	2597	HD2	PHE	166	41.528	74.144	52.480	1.00	0.00	H
ATOM	2598	C	PHE	166	40.852	71.236	53.894	1.00	0.00	C

ATOM	2599	O	PHE	166	41.324	71.885	54.829	1.00	0.00	O
ATOM	2600	N	MET	167	41.566	70.351	53.187	1.00	0.00	N
ATOM	2601	H	MET	167	41.100	69.865	52.427	1.00	0.00	H
ATOM	2602	CA	MET	167	42.954	69.962	53.486	1.00	0.00	C
ATOM	2603	HA	MET	167	43.305	70.534	54.345	1.00	0.00	H
ATOM	2604	CB	MET	167	42.997	68.467	53.852	1.00	0.00	C
ATOM	2605	HB2	MET	167	42.215	67.929	53.314	1.00	0.00	H
ATOM	2606	HB3	MET	167	43.953	68.037	53.553	1.00	0.00	H
ATOM	2607	CG	MET	167	42.817	68.250	55.358	1.00	0.00	C
ATOM	2608	HG2	MET	167	41.897	68.743	55.675	1.00	0.00	H
ATOM	2609	HG3	MET	167	42.703	67.181	55.537	1.00	0.00	H
ATOM	2610	SD	MET	167	44.187	68.850	56.398	1.00	0.00	S
ATOM	2611	CE	MET	167	45.516	67.724	55.905	1.00	0.00	C
ATOM	2612	HE1	MET	167	45.807	67.923	54.873	1.00	0.00	H
ATOM	2613	HE2	MET	167	46.379	67.874	56.553	1.00	0.00	H
ATOM	2614	HE3	MET	167	45.174	66.693	55.999	1.00	0.00	H
ATOM	2615	C	MET	167	43.963	70.266	52.369	1.00	0.00	C
ATOM	2616	O	MET	167	45.153	70.395	52.667	1.00	0.00	O
ATOM	2617	N	GLY	168	43.541	70.397	51.107	1.00	0.00	N
ATOM	2618	H	GLY	168	42.563	70.261	50.886	1.00	0.00	H
ATOM	2619	CA	GLY	168	44.446	70.822	50.033	1.00	0.00	C
ATOM	2620	HA2	GLY	168	44.608	71.895	50.126	1.00	0.00	H
ATOM	2621	HA3	GLY	168	45.412	70.331	50.159	1.00	0.00	H
ATOM	2622	C	GLY	168	43.985	70.534	48.605	1.00	0.00	C
ATOM	2623	O	GLY	168	42.958	69.892	48.374	1.00	0.00	O
ATOM	2624	N	GLN	169	44.785	71.011	47.648	1.00	0.00	N
ATOM	2625	H	GLN	169	45.611	71.523	47.953	1.00	0.00	H
ATOM	2626	CA	GLN	169	44.764	70.592	46.244	1.00	0.00	C
ATOM	2627	HA	GLN	169	43.858	70.023	46.051	1.00	0.00	H
ATOM	2628	CB	GLN	169	44.791	71.811	45.306	1.00	0.00	C
ATOM	2629	HB2	GLN	169	45.481	72.568	45.685	1.00	0.00	H
ATOM	2630	HB3	GLN	169	45.141	71.489	44.325	1.00	0.00	H
ATOM	2631	CG	GLN	169	43.390	72.404	45.138	1.00	0.00	C
ATOM	2632	HG2	GLN	169	42.718	71.591	44.863	1.00	0.00	H
ATOM	2633	HG3	GLN	169	43.051	72.825	46.084	1.00	0.00	H
ATOM	2634	CD	GLN	169	43.286	73.474	44.053	1.00	0.00	C
ATOM	2635	OE1	GLN	169	44.254	74.014	43.538	1.00	0.00	O
ATOM	2636	NE2	GLN	169	42.083	73.817	43.657	1.00	0.00	N
ATOM	2637	HE21	GLN	169	42.013	74.488	42.915	1.00	0.00	H
ATOM	2638	HE22	GLN	169	41.286	73.336	44.068	1.00	0.00	H
ATOM	2639	C	GLN	169	45.959	69.682	45.949	1.00	0.00	C
ATOM	2640	O	GLN	169	47.090	70.028	46.284	1.00	0.00	O
ATOM	2641	N	ILE	170	45.722	68.541	45.294	1.00	0.00	N
ATOM	2642	H	ILE	170	44.764	68.310	45.055	1.00	0.00	H
ATOM	2643	CA	ILE	170	46.780	67.577	44.942	1.00	0.00	C
ATOM	2644	HA	ILE	170	47.737	68.099	44.991	1.00	0.00	H
ATOM	2645	CB	ILE	170	46.863	66.418	45.964	1.00	0.00	C
ATOM	2646	HB	ILE	170	47.739	65.827	45.690	1.00	0.00	H
ATOM	2647	CG2	ILE	170	47.110	66.948	47.387	1.00	0.00	C
ATOM	2648	HG21	ILE	170	47.939	67.658	47.383	1.00	0.00	H
ATOM	2649	HG22	ILE	170	47.364	66.131	48.057	1.00	0.00	H
ATOM	2650	HG23	ILE	170	46.220	67.449	47.770	1.00	0.00	H

ATOM	2651	CG1	ILE	170	45.640	65.477	45.921	1.00	0.00	C
ATOM	2652	HG12	ILE	170	44.770	65.981	46.344	1.00	0.00	H
ATOM	2653	HG13	ILE	170	45.412	65.216	44.888	1.00	0.00	H
ATOM	2654	CD1	ILE	170	45.887	64.166	46.678	1.00	0.00	C
ATOM	2655	HD11	ILE	170	46.765	63.660	46.274	1.00	0.00	H
ATOM	2656	HD12	ILE	170	45.023	63.521	46.554	1.00	0.00	H
ATOM	2657	HD13	ILE	170	46.030	64.351	47.741	1.00	0.00	H
ATOM	2658	C	ILE	170	46.667	67.080	43.495	1.00	0.00	C
ATOM	2659	O	ILE	170	45.604	67.150	42.878	1.00	0.00	O
ATOM	2660	N	GLU	171	47.777	66.569	42.959	1.00	0.00	N
ATOM	2661	H	GLU	171	48.597	66.493	43.540	1.00	0.00	H
ATOM	2662	CA	GLU	171	47.925	66.128	41.566	1.00	0.00	C
ATOM	2663	HA	GLU	171	46.962	66.204	41.058	1.00	0.00	H
ATOM	2664	CB	GLU	171	48.921	67.035	40.816	1.00	0.00	C
ATOM	2665	HB2	GLU	171	49.868	67.047	41.359	1.00	0.00	H
ATOM	2666	HB3	GLU	171	49.096	66.617	39.824	1.00	0.00	H
ATOM	2667	CG	GLU	171	48.410	68.477	40.656	1.00	0.00	C
ATOM	2668	HG2	GLU	171	47.508	68.458	40.041	1.00	0.00	H
ATOM	2669	HG3	GLU	171	48.142	68.868	41.639	1.00	0.00	H
ATOM	2670	CD	GLU	171	49.431	69.435	40.023	1.00	0.00	C
ATOM	2671	OE1	GLU	171	49.236	70.666	40.166	1.00	0.00	O
ATOM	2672	OE2	GLU	171	50.419	68.983	39.395	1.00	0.00	O
ATOM	2673	C	GLU	171	48.381	64.661	41.483	1.00	0.00	C
ATOM	2674	O	GLU	171	49.247	64.220	42.238	1.00	0.00	O
ATOM	2675	N	ALA	172	47.800	63.920	40.540	1.00	0.00	N
ATOM	2676	H	ALA	172	47.112	64.366	39.948	1.00	0.00	H
ATOM	2677	CA	ALA	172	48.141	62.550	40.148	1.00	0.00	C
ATOM	2678	HA	ALA	172	49.225	62.448	40.069	1.00	0.00	H
ATOM	2679	CB	ALA	172	47.606	61.573	41.209	1.00	0.00	C
ATOM	2680	HB1	ALA	172	48.067	61.787	42.174	1.00	0.00	H
ATOM	2681	HB2	ALA	172	47.852	60.548	40.930	1.00	0.00	H
ATOM	2682	HB3	ALA	172	46.524	61.674	41.299	1.00	0.00	H
ATOM	2683	C	ALA	172	47.508	62.271	38.769	1.00	0.00	C
ATOM	2684	O	ALA	172	46.681	63.071	38.312	1.00	0.00	O
ATOM	2685	N	LYS	173	47.804	61.114	38.140	1.00	0.00	N
ATOM	2686	H	LYS	173	48.501	60.514	38.558	1.00	0.00	H
ATOM	2687	CA	LYS	173	47.261	60.755	36.801	1.00	0.00	C
ATOM	2688	HA	LYS	173	47.627	59.764	36.529	1.00	0.00	H
ATOM	2689	CB	LYS	173	45.719	60.693	36.954	1.00	0.00	C
ATOM	2690	HB2	LYS	173	45.474	60.794	38.014	1.00	0.00	H
ATOM	2691	HB3	LYS	173	45.285	61.559	36.458	1.00	0.00	H
ATOM	2692	CG	LYS	173	44.986	59.418	36.512	1.00	0.00	C
ATOM	2693	HG2	LYS	173	44.880	59.392	35.432	1.00	0.00	H
ATOM	2694	HG3	LYS	173	45.544	58.536	36.832	1.00	0.00	H
ATOM	2695	CD	LYS	173	43.577	59.363	37.122	1.00	0.00	C
ATOM	2696	HD2	LYS	173	42.999	58.637	36.552	1.00	0.00	H
ATOM	2697	HD3	LYS	173	43.648	59.017	38.155	1.00	0.00	H
ATOM	2698	CE	LYS	173	42.848	60.712	37.095	1.00	0.00	C
ATOM	2699	HE2	LYS	173	43.389	61.426	37.723	1.00	0.00	H
ATOM	2700	HE3	LYS	173	42.859	61.074	36.068	1.00	0.00	H
ATOM	2701	NZ	LYS	173	41.459	60.577	37.570	1.00	0.00	N
ATOM	2702	HZ1	LYS	173	41.411	60.101	38.460	1.00	0.00	H

ATOM	2703	HZ2	LYS	173	40.953	59.906	36.976	1.00	0.00	H
ATOM	2704	HZ3	LYS	173	40.934	61.435	37.573	1.00	0.00	H
ATOM	2705	C	LYS	173	47.803	61.741	35.722	1.00	0.00	C
ATOM	2706	O	LYS	173	48.612	62.603	36.034	1.00	0.00	O
ATOM	2707	N	GLY	174	47.461	61.701	34.432	1.00	0.00	N
ATOM	2708	H	GLY	174	48.032	62.314	33.866	1.00	0.00	H
ATOM	2709	CA	GLY	174	46.463	60.898	33.710	1.00	0.00	C
ATOM	2710	HA2	GLY	174	46.834	60.752	32.694	1.00	0.00	H
ATOM	2711	HA3	GLY	174	46.386	59.907	34.150	1.00	0.00	H
ATOM	2712	C	GLY	174	45.067	61.545	33.578	1.00	0.00	C
ATOM	2713	O	GLY	174	44.160	60.932	33.030	1.00	0.00	O
ATOM	2714	N	LYS	175	44.900	62.787	34.063	1.00	0.00	N
ATOM	2715	H	LYS	175	45.711	63.166	34.526	1.00	0.00	H
ATOM	2716	CA	LYS	175	43.779	63.719	33.761	1.00	0.00	C
ATOM	2717	HA	LYS	175	43.395	63.481	32.767	1.00	0.00	H
ATOM	2718	CB	LYS	175	42.619	63.583	34.780	1.00	0.00	C
ATOM	2719	HB2	LYS	175	43.044	63.418	35.771	1.00	0.00	H
ATOM	2720	HB3	LYS	175	42.071	64.527	34.824	1.00	0.00	H
ATOM	2721	CG	LYS	175	41.587	62.471	34.481	1.00	0.00	C
ATOM	2722	HG2	LYS	175	42.106	61.541	34.267	1.00	0.00	H
ATOM	2723	HG3	LYS	175	40.981	62.316	35.373	1.00	0.00	H
ATOM	2724	CD	LYS	175	40.630	62.789	33.322	1.00	0.00	C
ATOM	2725	HD2	LYS	175	39.915	63.554	33.630	1.00	0.00	H
ATOM	2726	HD3	LYS	175	41.204	63.189	32.485	1.00	0.00	H
ATOM	2727	CE	LYS	175	39.878	61.552	32.804	1.00	0.00	C
ATOM	2728	HE2	LYS	175	39.371	61.842	31.879	1.00	0.00	H
ATOM	2729	HE3	LYS	175	40.599	60.777	32.532	1.00	0.00	H
ATOM	2730	NZ	LYS	175	38.856	60.994	33.728	1.00	0.00	N
ATOM	2731	HZ1	LYS	175	38.298	60.307	33.213	1.00	0.00	H
ATOM	2732	HZ2	LYS	175	38.202	61.708	34.032	1.00	0.00	H
ATOM	2733	HZ3	LYS	175	39.240	60.499	34.534	1.00	0.00	H
ATOM	2734	C	LYS	175	44.242	65.189	33.658	1.00	0.00	C
ATOM	2735	O	LYS	175	43.593	65.967	32.967	1.00	0.00	O
ATOM	2736	N	ALA	176	45.388	65.526	34.268	1.00	0.00	N
ATOM	2737	H	ALA	176	45.817	64.842	34.863	1.00	0.00	H
ATOM	2738	CA	ALA	176	46.128	66.791	34.124	1.00	0.00	C
ATOM	2739	HA	ALA	176	47.024	66.663	34.732	1.00	0.00	H
ATOM	2740	CB	ALA	176	46.639	66.936	32.680	1.00	0.00	C
ATOM	2741	HB1	ALA	176	47.145	66.022	32.365	1.00	0.00	H
ATOM	2742	HB2	ALA	176	47.349	67.763	32.622	1.00	0.00	H
ATOM	2743	HB3	ALA	176	45.808	67.145	32.005	1.00	0.00	H
ATOM	2744	C	ALA	176	45.475	68.068	34.712	1.00	0.00	C
ATOM	2745	O	ALA	176	45.715	69.169	34.215	1.00	0.00	O
ATOM	2746	N	GLU	177	44.708	67.940	35.801	1.00	0.00	N
ATOM	2747	H	GLU	177	44.554	67.021	36.184	1.00	0.00	H
ATOM	2748	CA	GLU	177	44.161	69.060	36.591	1.00	0.00	C
ATOM	2749	HA	GLU	177	44.829	69.914	36.488	1.00	0.00	H
ATOM	2750	CB	GLU	177	42.786	69.455	36.007	1.00	0.00	C
ATOM	2751	HB2	GLU	177	42.892	69.656	34.939	1.00	0.00	H
ATOM	2752	HB3	GLU	177	42.130	68.588	36.104	1.00	0.00	H
ATOM	2753	CG	GLU	177	42.062	70.651	36.655	1.00	0.00	C
ATOM	2754	HG2	GLU	177	41.006	70.572	36.386	1.00	0.00	H

ATOM	2755	HG3	GLU	177	42.114	70.570	37.742	1.00	0.00	H
ATOM	2756	CD	GLU	177	42.554	72.043	36.214	1.00	0.00	C
ATOM	2757	OE1	GLU	177	43.700	72.193	35.729	1.00	0.00	O
ATOM	2758	OE2	GLU	177	41.786	73.020	36.397	1.00	0.00	O
ATOM	2759	C	GLU	177	44.089	68.651	38.087	1.00	0.00	C
ATOM	2760	O	GLU	177	43.774	67.487	38.365	1.00	0.00	O
ATOM	2761	N	PRO	178	44.412	69.537	39.056	1.00	0.00	N
ATOM	2762	CD	PRO	178	44.891	70.901	38.866	1.00	0.00	C
ATOM	2763	HD2	PRO	178	44.274	71.461	38.168	1.00	0.00	H
ATOM	2764	HD3	PRO	178	45.922	70.874	38.512	1.00	0.00	H
ATOM	2765	CG	PRO	178	44.843	71.562	40.241	1.00	0.00	C
ATOM	2766	HG2	PRO	178	43.840	71.950	40.430	1.00	0.00	H
ATOM	2767	HG3	PRO	178	45.588	72.353	40.337	1.00	0.00	H
ATOM	2768	CB	PRO	178	45.130	70.390	41.175	1.00	0.00	C
ATOM	2769	HB2	PRO	178	44.747	70.572	42.179	1.00	0.00	H
ATOM	2770	HB3	PRO	178	46.204	70.214	41.209	1.00	0.00	H
ATOM	2771	CA	PRO	178	44.431	69.210	40.488	1.00	0.00	C
ATOM	2772	HA	PRO	178	45.028	68.310	40.638	1.00	0.00	H
ATOM	2773	C	PRO	178	43.039	68.972	41.095	1.00	0.00	C
ATOM	2774	O	PRO	178	42.035	69.522	40.643	1.00	0.00	O
ATOM	2775	N	LEU	179	42.991	68.190	42.180	1.00	0.00	N
ATOM	2776	H	LEU	179	43.869	67.798	42.510	1.00	0.00	H
ATOM	2777	CA	LEU	179	41.764	67.798	42.883	1.00	0.00	C
ATOM	2778	HA	LEU	179	40.903	68.193	42.343	1.00	0.00	H
ATOM	2779	CB	LEU	179	41.659	66.260	42.901	1.00	0.00	C
ATOM	2780	HB2	LEU	179	42.468	65.874	43.524	1.00	0.00	H
ATOM	2781	HB3	LEU	179	40.719	65.991	43.378	1.00	0.00	H
ATOM	2782	CG	LEU	179	41.739	65.557	41.530	1.00	0.00	C
ATOM	2783	HG	LEU	179	42.702	65.764	41.064	1.00	0.00	H
ATOM	2784	CD1	LEU	179	41.624	64.043	41.733	1.00	0.00	C
ATOM	2785	HD11	LEU	179	42.427	63.703	42.388	1.00	0.00	H
ATOM	2786	HD12	LEU	179	41.722	63.539	40.773	1.00	0.00	H
ATOM	2787	HD13	LEU	179	40.662	63.793	42.181	1.00	0.00	H
ATOM	2788	CD2	LEU	179	40.620	65.986	40.579	1.00	0.00	C
ATOM	2789	HD21	LEU	179	39.645	65.781	41.013	1.00	0.00	H
ATOM	2790	HD22	LEU	179	40.711	67.050	40.359	1.00	0.00	H
ATOM	2791	HD23	LEU	179	40.719	65.449	39.636	1.00	0.00	H
ATOM	2792	C	LEU	179	41.710	68.357	44.317	1.00	0.00	C
ATOM	2793	O	LEU	179	42.661	68.188	45.079	1.00	0.00	O
ATOM	2794	N	ASN	180	40.577	68.963	44.699	1.00	0.00	N
ATOM	2795	H	ASN	180	39.864	69.107	44.004	1.00	0.00	H
ATOM	2796	CA	ASN	180	40.285	69.389	46.079	1.00	0.00	C
ATOM	2797	HA	ASN	180	41.151	69.934	46.463	1.00	0.00	H
ATOM	2798	CB	ASN	180	39.056	70.328	46.104	1.00	0.00	C
ATOM	2799	HB2	ASN	180	38.207	69.799	45.681	1.00	0.00	H
ATOM	2800	HB3	ASN	180	38.812	70.566	47.139	1.00	0.00	H
ATOM	2801	CG	ASN	180	39.193	71.650	45.365	1.00	0.00	C
ATOM	2802	OD1	ASN	180	40.270	72.162	45.120	1.00	0.00	O
ATOM	2803	ND2	ASN	180	38.092	72.256	44.988	1.00	0.00	N
ATOM	2804	HD21	ASN	180	37.190	71.866	45.190	1.00	0.00	H
ATOM	2805	HD22	ASN	180	38.190	73.168	44.551	1.00	0.00	H
ATOM	2806	C	ASN	180	40.030	68.179	47.009	1.00	0.00	C

ATOM	2807	O	ASN	180	39.288	67.259	46.636	1.00	0.00	O
ATOM	2808	N	LEU	181	40.575	68.220	48.232	1.00	0.00	N
ATOM	2809	H	LEU	181	41.201	68.992	48.445	1.00	0.00	H
ATOM	2810	CA	LEU	181	40.551	67.121	49.207	1.00	0.00	C
ATOM	2811	HA	LEU	181	39.766	66.428	48.910	1.00	0.00	H
ATOM	2812	CB	LEU	181	41.918	66.412	49.129	1.00	0.00	C
ATOM	2813	HB2	LEU	181	42.237	66.359	48.086	1.00	0.00	H
ATOM	2814	HB3	LEU	181	42.650	67.024	49.660	1.00	0.00	H
ATOM	2815	CG	LEU	181	41.936	64.988	49.705	1.00	0.00	C
ATOM	2816	HG	LEU	181	41.517	64.983	50.710	1.00	0.00	H
ATOM	2817	CD1	LEU	181	41.143	64.032	48.815	1.00	0.00	C
ATOM	2818	HD11	LEU	181	40.112	64.349	48.740	1.00	0.00	H
ATOM	2819	HD12	LEU	181	41.158	63.035	49.246	1.00	0.00	H
ATOM	2820	HD13	LEU	181	41.568	64.016	47.813	1.00	0.00	H
ATOM	2821	CD2	LEU	181	43.374	64.476	49.766	1.00	0.00	C
ATOM	2822	HD21	LEU	181	43.827	64.527	48.778	1.00	0.00	H
ATOM	2823	HD22	LEU	181	43.953	65.089	50.457	1.00	0.00	H
ATOM	2824	HD23	LEU	181	43.386	63.442	50.109	1.00	0.00	H
ATOM	2825	C	LEU	181	40.219	67.591	50.642	1.00	0.00	C
ATOM	2826	O	LEU	181	40.620	68.686	51.045	1.00	0.00	O
ATOM	2827	N	TYR	182	39.491	66.766	51.410	1.00	0.00	N
ATOM	2828	H	TYR	182	39.195	65.881	51.014	1.00	0.00	H
ATOM	2829	CA	TYR	182	38.828	67.165	52.665	1.00	0.00	C
ATOM	2830	HA	TYR	182	39.306	68.071	53.038	1.00	0.00	H
ATOM	2831	CB	TYR	182	37.349	67.504	52.389	1.00	0.00	C
ATOM	2832	HB2	TYR	182	36.835	66.584	52.107	1.00	0.00	H
ATOM	2833	HB3	TYR	182	36.892	67.845	53.319	1.00	0.00	H
ATOM	2834	CG	TYR	182	37.071	68.541	51.309	1.00	0.00	C
ATOM	2835	CD1	TYR	182	36.655	69.845	51.649	1.00	0.00	C
ATOM	2836	HD1	TYR	182	36.563	70.128	52.689	1.00	0.00	H
ATOM	2837	CE1	TYR	182	36.311	70.769	50.639	1.00	0.00	C
ATOM	2838	HE1	TYR	182	35.959	71.757	50.898	1.00	0.00	H
ATOM	2839	CZ	TYR	182	36.403	70.394	49.281	1.00	0.00	C
ATOM	2840	OH	TYR	182	36.011	71.239	48.288	1.00	0.00	O
ATOM	2841	HH	TYR	182	35.782	70.731	47.494	1.00	0.00	H
ATOM	2842	CE2	TYR	182	36.864	69.106	48.945	1.00	0.00	C
ATOM	2843	HE2	TYR	182	36.978	68.829	47.913	1.00	0.00	H
ATOM	2844	CD2	TYR	182	37.155	68.173	49.952	1.00	0.00	C
ATOM	2845	HD2	TYR	182	37.441	67.166	49.682	1.00	0.00	H
ATOM	2846	C	TYR	182	38.918	66.116	53.797	1.00	0.00	C
ATOM	2847	O	TYR	182	39.101	64.921	53.557	1.00	0.00	O
ATOM	2848	N	GLU	183	38.751	66.606	55.027	1.00	0.00	N
ATOM	2849	H	GLU	183	38.580	67.605	55.079	1.00	0.00	H
ATOM	2850	CA	GLU	183	38.744	65.945	56.345	1.00	0.00	C
ATOM	2851	HA	GLU	183	38.988	64.888	56.238	1.00	0.00	H
ATOM	2852	CB	GLU	183	39.835	66.648	57.188	1.00	0.00	C
ATOM	2853	HB2	GLU	183	40.755	66.677	56.604	1.00	0.00	H
ATOM	2854	HB3	GLU	183	39.503	67.678	57.316	1.00	0.00	H
ATOM	2855	CG	GLU	183	40.195	66.065	58.573	1.00	0.00	C
ATOM	2856	HG2	GLU	183	39.378	65.459	58.955	1.00	0.00	H
ATOM	2857	HG3	GLU	183	41.047	65.395	58.449	1.00	0.00	H
ATOM	2858	CD	GLU	183	40.544	67.139	59.627	1.00	0.00	C

ATOM 2859 OE1 GLU 183 41.007 68.241 59.246 1.00 0.00 O
ATOM 2860 OE2 GLU 183 40.383 66.852 60.840 1.00 0.00 O
ATOM 2861 C GLU 183 37.349 66.081 57.012 1.00 0.00 C
ATOM 2862 O GLU 183 36.550 66.931 56.603 1.00 0.00 O
ATOM 2863 N ILE 184 37.060 65.281 58.051 1.00 0.00 N
ATOM 2864 H ILE 184 37.796 64.682 58.402 1.00 0.00 H
ATOM 2865 CA ILE 184 35.831 65.297 58.880 1.00 0.00 C
ATOM 2866 HA ILE 184 35.324 66.252 58.756 1.00 0.00 H
ATOM 2867 CB ILE 184 34.866 64.163 58.448 1.00 0.00 C
ATOM 2868 HB ILE 184 35.392 63.210 58.551 1.00 0.00 H
ATOM 2869 CG2 ILE 184 33.624 64.123 59.363 1.00 0.00 C
ATOM 2870 HG21 ILE 184 33.922 63.955 60.397 1.00 0.00 H
ATOM 2871 HG22 ILE 184 32.968 63.296 59.096 1.00 0.00 H
ATOM 2872 HG23 ILE 184 33.083 65.067 59.296 1.00 0.00 H
ATOM 2873 CG1 ILE 184 34.453 64.336 56.968 1.00 0.00 C
ATOM 2874 HG12 ILE 184 33.986 65.312 56.840 1.00 0.00 H
ATOM 2875 HG13 ILE 184 35.342 64.305 56.339 1.00 0.00 H
ATOM 2876 CD1 ILE 184 33.507 63.251 56.442 1.00 0.00 C
ATOM 2877 HD11 ILE 184 33.952 62.267 56.588 1.00 0.00 H
ATOM 2878 HD12 ILE 184 33.332 63.414 55.382 1.00 0.00 H
ATOM 2879 HD13 ILE 184 32.545 63.305 56.948 1.00 0.00 H
ATOM 2880 C ILE 184 36.191 65.222 60.368 1.00 0.00 C
ATOM 2881 O ILE 184 37.166 64.515 60.712 1.00 0.00 O
ATOM 2882 OXT ILE 184 35.485 65.836 61.194 1.00 0.00 O
TER 2883 ILE 184
ATOM 2883 N LEU 185 40.791 23.668 52.151 1.00 0.00 N
ATOM 2884 H1 LEU 185 40.219 23.897 51.346 1.00 0.00 H
ATOM 2885 H2 LEU 185 41.746 23.614 51.829 1.00 0.00 H
ATOM 2886 H3 LEU 185 40.512 22.754 52.487 1.00 0.00 H
ATOM 2887 CA LEU 185 40.644 24.689 53.219 1.00 0.00 C
ATOM 2888 HA LEU 185 40.801 25.664 52.758 1.00 0.00 H
ATOM 2889 CB LEU 185 39.242 24.721 53.865 1.00 0.00 C
ATOM 2890 HB2 LEU 185 39.074 23.794 54.412 1.00 0.00 H
ATOM 2891 HB3 LEU 185 39.224 25.533 54.595 1.00 0.00 H
ATOM 2892 CG LEU 185 38.078 24.929 52.876 1.00 0.00 C
ATOM 2893 HG LEU 185 38.067 24.109 52.157 1.00 0.00 H
ATOM 2894 CD1 LEU 185 36.748 24.932 53.628 1.00 0.00 C
ATOM 2895 HD11 LEU 185 36.620 23.989 54.161 1.00 0.00 H
ATOM 2896 HD12 LEU 185 35.920 25.049 52.928 1.00 0.00 H
ATOM 2897 HD13 LEU 185 36.719 25.751 54.348 1.00 0.00 H
ATOM 2898 CD2 LEU 185 38.207 26.249 52.112 1.00 0.00 C
ATOM 2899 HD21 LEU 185 38.260 27.081 52.816 1.00 0.00 H
ATOM 2900 HD22 LEU 185 39.092 26.247 51.477 1.00 0.00 H
ATOM 2901 HD23 LEU 185 37.331 26.384 51.476 1.00 0.00 H
ATOM 2902 C LEU 185 41.738 24.573 54.280 1.00 0.00 C
ATOM 2903 O LEU 185 42.472 25.539 54.434 1.00 0.00 O
ATOM 2904 N LYS 186 41.896 23.426 54.962 1.00 0.00 N
ATOM 2905 H LYS 186 41.267 22.659 54.727 1.00 0.00 H
ATOM 2906 CA LYS 186 42.746 23.190 56.163 1.00 0.00 C
ATOM 2907 HA LYS 186 42.454 23.912 56.929 1.00 0.00 H
ATOM 2908 CB LYS 186 42.424 21.775 56.703 1.00 0.00 C
ATOM 2909 HB2 LYS 186 42.611 21.057 55.903 1.00 0.00 H

ATOM	2910	HB3	LYS	186	43.090	21.528	57.532	1.00	0.00	H
ATOM	2911	CG	LYS	186	40.979	21.617	57.216	1.00	0.00	C
ATOM	2912	HG2	LYS	186	40.896	22.067	58.206	1.00	0.00	H
ATOM	2913	HG3	LYS	186	40.290	22.137	56.552	1.00	0.00	H
ATOM	2914	CD	LYS	186	40.564	20.137	57.281	1.00	0.00	C
ATOM	2915	HD2	LYS	186	40.764	19.674	56.313	1.00	0.00	H
ATOM	2916	HD3	LYS	186	41.140	19.618	58.049	1.00	0.00	H
ATOM	2917	CE	LYS	186	39.065	20.037	57.584	1.00	0.00	C
ATOM	2918	HE2	LYS	186	38.898	20.220	58.649	1.00	0.00	H
ATOM	2919	HE3	LYS	186	38.550	20.822	57.023	1.00	0.00	H
ATOM	2920	NZ	LYS	186	38.496	18.728	57.184	1.00	0.00	N
ATOM	2921	HZ1	LYS	186	38.874	17.946	57.716	1.00	0.00	H
ATOM	2922	HZ2	LYS	186	38.612	18.571	56.186	1.00	0.00	H
ATOM	2923	HZ3	LYS	186	37.487	18.741	57.323	1.00	0.00	H
ATOM	2924	C	LYS	186	44.278	23.381	56.005	1.00	0.00	C
ATOM	2925	O	LYS	186	45.046	22.915	56.843	1.00	0.00	O
ATOM	2926	N	SER	187	44.753	24.058	54.958	1.00	0.00	N
ATOM	2927	H	SER	187	44.073	24.555	54.402	1.00	0.00	H
ATOM	2928	CA	SER	187	46.168	24.412	54.785	1.00	0.00	C
ATOM	2929	HA	SER	187	46.782	23.514	54.871	1.00	0.00	H
ATOM	2930	CB	SER	187	46.393	25.021	53.392	1.00	0.00	C
ATOM	2931	HB2	SER	187	46.201	24.260	52.634	1.00	0.00	H
ATOM	2932	HB3	SER	187	45.683	25.836	53.246	1.00	0.00	H
ATOM	2933	OG	SER	187	47.715	25.520	53.224	1.00	0.00	O
ATOM	2934	HG	SER	187	47.971	26.048	53.996	1.00	0.00	H
ATOM	2935	C	SER	187	46.601	25.423	55.847	1.00	0.00	C
ATOM	2936	O	SER	187	46.202	26.586	55.790	1.00	0.00	O
ATOM	2937	N	HID	188	47.497	25.026	56.752	1.00	0.00	N
ATOM	2938	H	HID	188	47.770	24.054	56.785	1.00	0.00	H
ATOM	2939	CA	HID	188	48.217	25.986	57.591	1.00	0.00	C
ATOM	2940	HA	HID	188	47.477	26.571	58.140	1.00	0.00	H
ATOM	2941	CB	HID	188	49.117	25.271	58.613	1.00	0.00	C
ATOM	2942	HB2	HID	188	50.021	24.931	58.106	1.00	0.00	H
ATOM	2943	HB3	HID	188	49.425	25.990	59.374	1.00	0.00	H
ATOM	2944	CG	HID	188	48.490	24.082	59.299	1.00	0.00	C
ATOM	2945	ND1	HID	188	47.271	24.048	59.937	1.00	0.00	N
ATOM	2946	HD1	HID	188	46.621	24.816	60.034	1.00	0.00	H
ATOM	2947	CE1	HID	188	47.054	22.795	60.362	1.00	0.00	C
ATOM	2948	HE1	HID	188	46.161	22.470	60.885	1.00	0.00	H
ATOM	2949	NE2	HID	188	48.093	21.999	60.040	1.00	0.00	N
ATOM	2950	CD2	HID	188	49.000	22.812	59.342	1.00	0.00	C
ATOM	2951	HD2	HID	188	49.939	22.494	58.905	1.00	0.00	H
ATOM	2952	C	HID	188	49.035	26.948	56.712	1.00	0.00	C
ATOM	2953	O	HID	188	49.493	26.566	55.628	1.00	0.00	O
ATOM	2954	N	ALA	189	49.208	28.189	57.166	1.00	0.00	N
ATOM	2955	H	ALA	189	48.827	28.440	58.063	1.00	0.00	H
ATOM	2956	CA	ALA	189	50.016	29.185	56.468	1.00	0.00	C
ATOM	2957	HA	ALA	189	49.713	29.195	55.420	1.00	0.00	H
ATOM	2958	CB	ALA	189	49.713	30.560	57.071	1.00	0.00	C
ATOM	2959	HB1	ALA	189	50.273	31.328	56.537	1.00	0.00	H
ATOM	2960	HB2	ALA	189	49.999	30.579	58.124	1.00	0.00	H
ATOM	2961	HB3	ALA	189	48.648	30.777	56.979	1.00	0.00	H

ATOM	2962	C	ALA	189	51.523	28.860	56.517	1.00	0.00	C
ATOM	2963	O	ALA	189	51.993	28.176	57.428	1.00	0.00	O
ATOM	2964	N	THR	190	52.292	29.405	55.571	1.00	0.00	N
ATOM	2965	H	THR	190	51.833	29.958	54.851	1.00	0.00	H
ATOM	2966	CA	THR	190	53.762	29.322	55.547	1.00	0.00	C
ATOM	2967	HA	THR	190	54.115	29.024	56.534	1.00	0.00	H
ATOM	2968	CB	THR	190	54.305	28.284	54.541	1.00	0.00	C
ATOM	2969	HB	THR	190	55.390	28.260	54.649	1.00	0.00	H
ATOM	2970	CG2	THR	190	53.782	26.871	54.778	1.00	0.00	C
ATOM	2971	HG21	THR	190	53.959	26.581	55.814	1.00	0.00	H
ATOM	2972	HG22	THR	190	54.314	26.183	54.124	1.00	0.00	H
ATOM	2973	HG23	THR	190	52.713	26.821	54.567	1.00	0.00	H
ATOM	2974	OG1	THR	190	54.014	28.629	53.197	1.00	0.00	O
ATOM	2975	HG1	THR	190	54.014	29.602	53.172	1.00	0.00	H
ATOM	2976	C	THR	190	54.386	30.672	55.216	1.00	0.00	C
ATOM	2977	O	THR	190	54.059	31.263	54.187	1.00	0.00	O
ATOM	2978	N	VAL	191	55.353	31.100	56.027	1.00	0.00	N
ATOM	2979	H	VAL	191	55.567	30.545	56.841	1.00	0.00	H
ATOM	2980	CA	VAL	191	56.391	32.056	55.608	1.00	0.00	C
ATOM	2981	HA	VAL	191	55.929	32.942	55.175	1.00	0.00	H
ATOM	2982	CB	VAL	191	57.226	32.484	56.832	1.00	0.00	C
ATOM	2983	HB	VAL	191	57.577	31.590	57.351	1.00	0.00	H
ATOM	2984	CG1	VAL	191	58.439	33.341	56.486	1.00	0.00	C
ATOM	2985	HG11	VAL	191	58.121	34.249	55.973	1.00	0.00	H
ATOM	2986	HG12	VAL	191	58.977	33.619	57.391	1.00	0.00	H
ATOM	2987	HG13	VAL	191	59.127	32.795	55.840	1.00	0.00	H
ATOM	2988	CG2	VAL	191	56.360	33.301	57.799	1.00	0.00	C
ATOM	2989	HG21	VAL	191	55.498	32.719	58.109	1.00	0.00	H
ATOM	2990	HG22	VAL	191	56.023	34.216	57.312	1.00	0.00	H
ATOM	2991	HG23	VAL	191	56.943	33.560	58.684	1.00	0.00	H
ATOM	2992	C	VAL	191	57.252	31.376	54.534	1.00	0.00	C
ATOM	2993	O	VAL	191	57.660	30.228	54.734	1.00	0.00	O
ATOM	2994	N	ARG	192	57.458	32.016	53.377	1.00	0.00	N
ATOM	2995	H	ARG	192	57.099	32.964	53.283	1.00	0.00	H
ATOM	2996	CA	ARG	192	58.191	31.445	52.237	1.00	0.00	C
ATOM	2997	HA	ARG	192	59.065	30.927	52.636	1.00	0.00	H
ATOM	2998	CB	ARG	192	57.285	30.441	51.505	1.00	0.00	C
ATOM	2999	HB2	ARG	192	56.686	29.901	52.239	1.00	0.00	H
ATOM	3000	HB3	ARG	192	56.601	30.982	50.848	1.00	0.00	H
ATOM	3001	CG	ARG	192	58.089	29.412	50.692	1.00	0.00	C
ATOM	3002	HG2	ARG	192	58.820	29.917	50.061	1.00	0.00	H
ATOM	3003	HG3	ARG	192	58.620	28.753	51.380	1.00	0.00	H
ATOM	3004	CD	ARG	192	57.186	28.573	49.784	1.00	0.00	C
ATOM	3005	HD2	ARG	192	56.841	29.207	48.965	1.00	0.00	H
ATOM	3006	HD3	ARG	192	57.776	27.758	49.356	1.00	0.00	H
ATOM	3007	NE	ARG	192	56.019	28.029	50.505	1.00	0.00	N
ATOM	3008	HE	ARG	192	55.967	28.199	51.496	1.00	0.00	H
ATOM	3009	CZ	ARG	192	54.997	27.406	49.959	1.00	0.00	C
ATOM	3010	NH1	ARG	192	54.970	27.089	48.695	1.00	0.00	N
ATOM	3011	HH11	ARG	192	55.781	27.331	48.103	1.00	0.00	H
ATOM	3012	HH12	ARG	192	54.187	26.639	48.278	1.00	0.00	H
ATOM	3013	NH2	ARG	192	53.967	27.108	50.701	1.00	0.00	N

ATOM	3014	HH21	ARG	192	53.942	27.434	51.664	1.00	0.00	H
ATOM	3015	HH22	ARG	192	53.194	26.597	50.327	1.00	0.00	H
ATOM	3016	C	ARG	192	58.668	32.512	51.249	1.00	0.00	C
ATOM	3017	O	ARG	192	57.905	33.386	50.844	1.00	0.00	O
ATOM	3018	N	PHE	193	59.893	32.359	50.752	1.00	0.00	N
ATOM	3019	H	PHE	193	60.487	31.655	51.154	1.00	0.00	H
ATOM	3020	CA	PHE	193	60.437	33.191	49.678	1.00	0.00	C
ATOM	3021	HA	PHE	193	60.294	34.229	49.963	1.00	0.00	H
ATOM	3022	CB	PHE	193	61.951	32.953	49.560	1.00	0.00	C
ATOM	3023	HB2	PHE	193	62.117	31.972	49.114	1.00	0.00	H
ATOM	3024	HB3	PHE	193	62.371	33.702	48.889	1.00	0.00	H
ATOM	3025	CG	PHE	193	62.695	33.025	50.883	1.00	0.00	C
ATOM	3026	CD1	PHE	193	63.279	31.867	51.433	1.00	0.00	C
ATOM	3027	HD1	PHE	193	63.241	30.928	50.899	1.00	0.00	H
ATOM	3028	CE1	PHE	193	63.907	31.923	52.690	1.00	0.00	C
ATOM	3029	HE1	PHE	193	64.342	31.032	53.119	1.00	0.00	H
ATOM	3030	CZ	PHE	193	63.955	33.134	53.400	1.00	0.00	C
ATOM	3031	HZ	PHE	193	64.420	33.173	54.376	1.00	0.00	H
ATOM	3032	CE2	PHE	193	63.384	34.294	52.849	1.00	0.00	C
ATOM	3033	HE2	PHE	193	63.406	35.219	53.408	1.00	0.00	H
ATOM	3034	CD2	PHE	193	62.760	34.240	51.590	1.00	0.00	C
ATOM	3035	HD2	PHE	193	62.310	35.130	51.179	1.00	0.00	H
ATOM	3036	C	PHE	193	59.704	32.973	48.340	1.00	0.00	C
ATOM	3037	O	PHE	193	59.660	31.858	47.812	1.00	0.00	O
ATOM	3038	N	ALA	194	59.189	34.055	47.757	1.00	0.00	N
ATOM	3039	H	ALA	194	59.293	34.947	48.232	1.00	0.00	H
ATOM	3040	CA	ALA	194	58.441	34.071	46.502	1.00	0.00	C
ATOM	3041	HA	ALA	194	58.503	33.094	46.027	1.00	0.00	H
ATOM	3042	CB	ALA	194	56.966	34.319	46.823	1.00	0.00	C
ATOM	3043	HB1	ALA	194	56.862	35.268	47.350	1.00	0.00	H
ATOM	3044	HB2	ALA	194	56.588	33.512	47.452	1.00	0.00	H
ATOM	3045	HB3	ALA	194	56.380	34.351	45.904	1.00	0.00	H
ATOM	3046	C	ALA	194	58.991	35.108	45.509	1.00	0.00	C
ATOM	3047	O	ALA	194	59.592	36.114	45.897	1.00	0.00	O
ATOM	3048	N	THR	195	58.747	34.871	44.219	1.00	0.00	N
ATOM	3049	H	THR	195	58.192	34.048	44.006	1.00	0.00	H
ATOM	3050	CA	THR	195	58.829	35.893	43.167	1.00	0.00	C
ATOM	3051	HA	THR	195	59.507	36.688	43.476	1.00	0.00	H
ATOM	3052	CB	THR	195	59.325	35.322	41.829	1.00	0.00	C
ATOM	3053	HB	THR	195	58.522	34.761	41.357	1.00	0.00	H
ATOM	3054	CG2	THR	195	59.753	36.446	40.888	1.00	0.00	C
ATOM	3055	HG21	THR	195	60.191	36.020	39.988	1.00	0.00	H
ATOM	3056	HG22	THR	195	60.493	37.082	41.374	1.00	0.00	H
ATOM	3057	HG23	THR	195	58.890	37.048	40.604	1.00	0.00	H
ATOM	3058	OG1	THR	195	60.432	34.463	41.991	1.00	0.00	O
ATOM	3059	HG1	THR	195	60.110	33.603	42.298	1.00	0.00	H
ATOM	3060	C	THR	195	57.429	36.469	42.974	1.00	0.00	C
ATOM	3061	O	THR	195	56.480	35.706	42.799	1.00	0.00	O
ATOM	3062	N	ILE	196	57.280	37.790	43.010	1.00	0.00	N
ATOM	3063	H	ILE	196	58.113	38.367	43.073	1.00	0.00	H
ATOM	3064	CA	ILE	196	55.993	38.489	43.007	1.00	0.00	C
ATOM	3065	HA	ILE	196	55.186	37.765	42.891	1.00	0.00	H

ATOM	3066	CB	ILE	196	55.780	39.235	44.340	1.00	0.00	C
ATOM	3067	HB	ILE	196	56.534	40.021	44.405	1.00	0.00	H
ATOM	3068	CG2	ILE	196	54.406	39.917	44.316	1.00	0.00	C
ATOM	3069	HG21	ILE	196	54.394	40.739	43.599	1.00	0.00	H
ATOM	3070	HG22	ILE	196	54.205	40.321	45.297	1.00	0.00	H
ATOM	3071	HG23	ILE	196	53.626	39.202	44.051	1.00	0.00	H
ATOM	3072	CG1	ILE	196	55.935	38.371	45.615	1.00	0.00	C
ATOM	3073	HG12	ILE	196	56.938	37.945	45.644	1.00	0.00	H
ATOM	3074	HG13	ILE	196	55.843	39.025	46.483	1.00	0.00	H
ATOM	3075	CD1	ILE	196	54.922	37.236	45.786	1.00	0.00	C
ATOM	3076	HD11	ILE	196	53.909	37.635	45.818	1.00	0.00	H
ATOM	3077	HD12	ILE	196	55.113	36.723	46.728	1.00	0.00	H
ATOM	3078	HD13	ILE	196	55.020	36.522	44.969	1.00	0.00	H
ATOM	3079	C	ILE	196	55.924	39.467	41.834	1.00	0.00	C
ATOM	3080	O	ILE	196	56.836	40.272	41.640	1.00	0.00	O
ATOM	3081	N	MET	197	54.816	39.431	41.096	1.00	0.00	N
ATOM	3082	H	MET	197	54.095	38.761	41.346	1.00	0.00	H
ATOM	3083	CA	MET	197	54.566	40.241	39.906	1.00	0.00	C
ATOM	3084	HA	MET	197	55.400	40.925	39.754	1.00	0.00	H
ATOM	3085	CB	MET	197	54.508	39.292	38.704	1.00	0.00	C
ATOM	3086	HB2	MET	197	55.450	38.743	38.687	1.00	0.00	H
ATOM	3087	HB3	MET	197	53.697	38.582	38.853	1.00	0.00	H
ATOM	3088	CG	MET	197	54.354	39.949	37.329	1.00	0.00	C
ATOM	3089	HG2	MET	197	55.101	40.740	37.252	1.00	0.00	H
ATOM	3090	HG3	MET	197	54.601	39.193	36.585	1.00	0.00	H
ATOM	3091	SD	MET	197	52.752	40.661	36.849	1.00	0.00	S
ATOM	3092	CE	MET	197	51.628	39.247	36.848	1.00	0.00	C
ATOM	3093	HE1	MET	197	50.673	39.542	36.414	1.00	0.00	H
ATOM	3094	HE2	MET	197	52.052	38.437	36.255	1.00	0.00	H
ATOM	3095	HE3	MET	197	51.457	38.926	37.874	1.00	0.00	H
ATOM	3096	C	MET	197	53.292	41.075	40.056	1.00	0.00	C
ATOM	3097	O	MET	197	52.288	40.591	40.586	1.00	0.00	O
ATOM	3098	N	PHE	198	53.327	42.305	39.544	1.00	0.00	N
ATOM	3099	H	PHE	198	54.194	42.622	39.115	1.00	0.00	H
ATOM	3100	CA	PHE	198	52.148	43.139	39.316	1.00	0.00	C
ATOM	3101	HA	PHE	198	51.254	42.519	39.377	1.00	0.00	H
ATOM	3102	CB	PHE	198	52.069	44.214	40.409	1.00	0.00	C
ATOM	3103	HB2	PHE	198	52.284	43.751	41.373	1.00	0.00	H
ATOM	3104	HB3	PHE	198	52.849	44.953	40.224	1.00	0.00	H
ATOM	3105	CG	PHE	198	50.722	44.906	40.523	1.00	0.00	C
ATOM	3106	CD1	PHE	198	50.537	46.223	40.063	1.00	0.00	C
ATOM	3107	HD1	PHE	198	51.349	46.760	39.602	1.00	0.00	H
ATOM	3108	CE1	PHE	198	49.291	46.857	40.199	1.00	0.00	C
ATOM	3109	HE1	PHE	198	49.169	47.868	39.828	1.00	0.00	H
ATOM	3110	CZ	PHE	198	48.218	46.175	40.801	1.00	0.00	C
ATOM	3111	HZ	PHE	198	47.257	46.658	40.905	1.00	0.00	H
ATOM	3112	CE2	PHE	198	48.397	44.860	41.266	1.00	0.00	C
ATOM	3113	HE2	PHE	198	47.574	44.332	41.727	1.00	0.00	H
ATOM	3114	CD2	PHE	198	49.647	44.230	41.127	1.00	0.00	C
ATOM	3115	HD2	PHE	198	49.789	43.221	41.480	1.00	0.00	H
ATOM	3116	C	PHE	198	52.207	43.740	37.903	1.00	0.00	C
ATOM	3117	O	PHE	198	53.270	44.179	37.461	1.00	0.00	O

ATOM 3118 N CYS 199 51.086 43.768 37.184 1.00 0.00 N
ATOM 3119 H CYS 199 50.240 43.371 37.576 1.00 0.00 H
ATOM 3120 CA CYS 199 51.000 44.351 35.840 1.00 0.00 C
ATOM 3121 HA CYS 199 51.744 45.142 35.748 1.00 0.00 H
ATOM 3122 CB CYS 199 51.317 43.280 34.783 1.00 0.00 C
ATOM 3123 HB2 CYS 199 51.337 43.747 33.798 1.00 0.00 H
ATOM 3124 HB3 CYS 199 52.302 42.859 34.983 1.00 0.00 H
ATOM 3125 SG CYS 199 50.082 41.947 34.775 1.00 0.00 S
ATOM 3126 HG CYS 199 50.328 41.543 36.028 1.00 0.00 H
ATOM 3127 C CYS 199 49.634 45.001 35.612 1.00 0.00 C
ATOM 3128 O CYS 199 48.635 44.534 36.163 1.00 0.00 O
ATOM 3129 N ASP 200 49.582 46.079 34.823 1.00 0.00 N
ATOM 3130 H ASP 200 50.433 46.400 34.371 1.00 0.00 H
ATOM 3131 CA ASP 200 48.334 46.791 34.527 1.00 0.00 C
ATOM 3132 HA ASP 200 47.576 46.017 34.380 1.00 0.00 H
ATOM 3133 CB ASP 200 47.883 47.576 35.782 1.00 0.00 C
ATOM 3134 HB2 ASP 200 46.813 47.422 35.855 1.00 0.00 H
ATOM 3135 HB3 ASP 200 48.296 47.117 36.681 1.00 0.00 H
ATOM 3136 CG ASP 200 48.089 49.086 35.900 1.00 0.00 C
ATOM 3137 OD1 ASP 200 47.638 49.613 36.950 1.00 0.00 O
ATOM 3138 OD2 ASP 200 48.315 49.839 34.931 1.00 0.00 O
ATOM 3139 C ASP 200 48.349 47.574 33.194 1.00 0.00 C
ATOM 3140 O ASP 200 49.383 47.730 32.538 1.00 0.00 O
ATOM 3141 N ILE 201 47.166 47.996 32.740 1.00 0.00 N
ATOM 3142 H ILE 201 46.367 47.893 33.352 1.00 0.00 H
ATOM 3143 CA ILE 201 46.946 48.653 31.443 1.00 0.00 C
ATOM 3144 HA ILE 201 47.571 48.168 30.691 1.00 0.00 H
ATOM 3145 CB ILE 201 45.473 48.471 31.015 1.00 0.00 C
ATOM 3146 HB ILE 201 44.843 48.808 31.839 1.00 0.00 H
ATOM 3147 CG2 ILE 201 45.138 49.328 29.779 1.00 0.00 C
ATOM 3148 HG21 ILE 201 45.172 50.390 30.027 1.00 0.00 H
ATOM 3149 HG22 ILE 201 44.138 49.108 29.413 1.00 0.00 H
ATOM 3150 HG23 ILE 201 45.857 49.127 28.984 1.00 0.00 H
ATOM 3151 CG1 ILE 201 45.175 46.976 30.746 1.00 0.00 C
ATOM 3152 HG12 ILE 201 45.592 46.688 29.781 1.00 0.00 H
ATOM 3153 HG13 ILE 201 45.653 46.355 31.503 1.00 0.00 H
ATOM 3154 CD1 ILE 201 43.681 46.634 30.787 1.00 0.00 C
ATOM 3155 HD11 ILE 201 43.290 46.813 31.789 1.00 0.00 H
ATOM 3156 HD12 ILE 201 43.542 45.584 30.537 1.00 0.00 H
ATOM 3157 HD13 ILE 201 43.128 47.238 30.074 1.00 0.00 H
ATOM 3158 C ILE 201 47.343 50.135 31.516 1.00 0.00 C
ATOM 3159 O ILE 201 46.921 50.853 32.431 1.00 0.00 O
ATOM 3160 N LYS 202 48.108 50.639 30.538 1.00 0.00 N
ATOM 3161 H LYS 202 48.361 50.025 29.767 1.00 0.00 H
ATOM 3162 CA LYS 202 48.525 52.057 30.495 1.00 0.00 C
ATOM 3163 HA LYS 202 49.081 52.280 31.406 1.00 0.00 H
ATOM 3164 CB LYS 202 49.429 52.310 29.274 1.00 0.00 C
ATOM 3165 HB2 LYS 202 50.293 51.647 29.329 1.00 0.00 H
ATOM 3166 HB3 LYS 202 48.865 52.071 28.373 1.00 0.00 H
ATOM 3167 CG LYS 202 49.919 53.772 29.191 1.00 0.00 C
ATOM 3168 HG2 LYS 202 49.084 54.471 29.239 1.00 0.00 H
ATOM 3169 HG3 LYS 202 50.573 53.964 30.043 1.00 0.00 H

ATOM	3170	CD	LYS	202	50.698	54.053	27.901	1.00	0.00	C
ATOM	3171	HD2	LYS	202	51.257	54.984	28.020	1.00	0.00	H
ATOM	3172	HD3	LYS	202	51.406	53.239	27.763	1.00	0.00	H
ATOM	3173	CE	LYS	202	49.804	54.171	26.656	1.00	0.00	C
ATOM	3174	HE2	LYS	202	49.194	53.269	26.577	1.00	0.00	H
ATOM	3175	HE3	LYS	202	49.130	55.026	26.766	1.00	0.00	H
ATOM	3176	NZ	LYS	202	50.615	54.319	25.422	1.00	0.00	N
ATOM	3177	HZ1	LYS	202	50.275	53.695	24.690	1.00	0.00	H
ATOM	3178	HZ2	LYS	202	51.582	54.045	25.589	1.00	0.00	H
ATOM	3179	HZ3	LYS	202	50.642	55.280	25.085	1.00	0.00	H
ATOM	3180	C	LYS	202	47.315	52.999	30.441	1.00	0.00	C
ATOM	3181	O	LYS	202	46.571	52.975	29.462	1.00	0.00	O
ATOM	3182	N	GLY	203	47.156	53.867	31.445	1.00	0.00	N
ATOM	3183	H	GLY	203	47.740	53.786	32.280	1.00	0.00	H
ATOM	3184	CA	GLY	203	46.171	54.954	31.424	1.00	0.00	C
ATOM	3185	HA2	GLY	203	46.193	55.459	32.390	1.00	0.00	H
ATOM	3186	HA3	GLY	203	46.440	55.670	30.646	1.00	0.00	H
ATOM	3187	C	GLY	203	44.735	54.486	31.182	1.00	0.00	C
ATOM	3188	O	GLY	203	44.020	55.108	30.393	1.00	0.00	O
ATOM	3189	N	PHE	204	44.322	53.390	31.829	1.00	0.00	N
ATOM	3190	H	PHE	204	44.926	53.047	32.571	1.00	0.00	H
ATOM	3191	CA	PHE	204	43.007	52.750	31.701	1.00	0.00	C
ATOM	3192	HA	PHE	204	42.903	52.374	30.685	1.00	0.00	H
ATOM	3193	CB	PHE	204	42.940	51.559	32.672	1.00	0.00	C
ATOM	3194	HB2	PHE	204	43.783	50.897	32.472	1.00	0.00	H
ATOM	3195	HB3	PHE	204	43.057	51.924	33.694	1.00	0.00	H
ATOM	3196	CG	PHE	204	41.657	50.749	32.601	1.00	0.00	C
ATOM	3197	CD1	PHE	204	41.567	49.636	31.744	1.00	0.00	C
ATOM	3198	HD1	PHE	204	42.414	49.354	31.139	1.00	0.00	H
ATOM	3199	CE1	PHE	204	40.381	48.882	31.681	1.00	0.00	C
ATOM	3200	HE1	PHE	204	40.320	48.028	31.023	1.00	0.00	H
ATOM	3201	CZ	PHE	204	39.279	49.238	32.478	1.00	0.00	C
ATOM	3202	HZ	PHE	204	38.369	48.658	32.432	1.00	0.00	H
ATOM	3203	CE2	PHE	204	39.363	50.350	33.334	1.00	0.00	C
ATOM	3204	HE2	PHE	204	38.518	50.629	33.944	1.00	0.00	H
ATOM	3205	CD2	PHE	204	40.552	51.099	33.401	1.00	0.00	C
ATOM	3206	HD2	PHE	204	40.617	51.947	34.069	1.00	0.00	H
ATOM	3207	C	PHE	204	41.849	53.728	31.953	1.00	0.00	C
ATOM	3208	O	PHE	204	40.799	53.616	31.316	1.00	0.00	O
ATOM	3209	N	THR	205	42.075	54.758	32.779	1.00	0.00	N
ATOM	3210	H	THR	205	42.939	54.759	33.317	1.00	0.00	H
ATOM	3211	CA	THR	205	41.207	55.942	32.903	1.00	0.00	C
ATOM	3212	HA	THR	205	40.347	55.695	33.524	1.00	0.00	H
ATOM	3213	CB	THR	205	41.991	57.063	33.603	1.00	0.00	C
ATOM	3214	HB	THR	205	42.975	57.158	33.142	1.00	0.00	H
ATOM	3215	CG2	THR	205	41.311	58.427	33.546	1.00	0.00	C
ATOM	3216	HG21	THR	205	41.759	59.087	34.289	1.00	0.00	H
ATOM	3217	HG22	THR	205	40.246	58.315	33.741	1.00	0.00	H
ATOM	3218	HG23	THR	205	41.457	58.863	32.558	1.00	0.00	H
ATOM	3219	OG1	THR	205	42.158	56.722	34.953	1.00	0.00	O
ATOM	3220	HG1	THR	205	42.985	56.169	34.990	1.00	0.00	H
ATOM	3221	C	THR	205	40.694	56.445	31.548	1.00	0.00	C

ATOM	3222	O	THR	205	39.487	56.615	31.365	1.00	0.00	O
ATOM	3223	N	SER	206	41.598	56.597	30.575	1.00	0.00	N
ATOM	3224	H	SER	206	42.556	56.346	30.784	1.00	0.00	H
ATOM	3225	CA	SER	206	41.328	57.119	29.228	1.00	0.00	C
ATOM	3226	HA	SER	206	40.913	58.123	29.315	1.00	0.00	H
ATOM	3227	CB	SER	206	42.622	57.189	28.400	1.00	0.00	C
ATOM	3228	HB2	SER	206	42.871	56.197	28.017	1.00	0.00	H
ATOM	3229	HB3	SER	206	42.464	57.855	27.551	1.00	0.00	H
ATOM	3230	OG	SER	206	43.714	57.653	29.166	1.00	0.00	O
ATOM	3231	HG	SER	206	44.112	56.874	29.585	1.00	0.00	H
ATOM	3232	C	SER	206	40.340	56.251	28.450	1.00	0.00	C
ATOM	3233	O	SER	206	39.516	56.775	27.707	1.00	0.00	O
ATOM	3234	N	LEU	207	40.415	54.930	28.638	1.00	0.00	N
ATOM	3235	H	LEU	207	41.080	54.593	29.323	1.00	0.00	H
ATOM	3236	CA	LEU	207	39.499	53.952	28.051	1.00	0.00	C
ATOM	3237	HA	LEU	207	39.360	54.178	26.992	1.00	0.00	H
ATOM	3238	CB	LEU	207	40.100	52.535	28.196	1.00	0.00	C
ATOM	3239	HB2	LEU	207	40.016	52.232	29.240	1.00	0.00	H
ATOM	3240	HB3	LEU	207	39.494	51.843	27.610	1.00	0.00	H
ATOM	3241	CG	LEU	207	41.578	52.375	27.783	1.00	0.00	C
ATOM	3242	HG	LEU	207	42.197	53.082	28.335	1.00	0.00	H
ATOM	3243	CD1	LEU	207	42.062	50.970	28.132	1.00	0.00	C
ATOM	3244	HD11	LEU	207	41.960	50.804	29.203	1.00	0.00	H
ATOM	3245	HD12	LEU	207	43.111	50.863	27.856	1.00	0.00	H
ATOM	3246	HD13	LEU	207	41.468	50.230	27.601	1.00	0.00	H
ATOM	3247	CD2	LEU	207	41.789	52.594	26.287	1.00	0.00	C
ATOM	3248	HD21	LEU	207	41.238	51.845	25.721	1.00	0.00	H
ATOM	3249	HD22	LEU	207	41.456	53.591	26.002	1.00	0.00	H
ATOM	3250	HD23	LEU	207	42.850	52.504	26.051	1.00	0.00	H
ATOM	3251	C	LEU	207	38.126	54.038	28.736	1.00	0.00	C
ATOM	3252	O	LEU	207	37.094	54.036	28.066	1.00	0.00	O
ATOM	3253	N	SER	208	38.130	54.155	30.071	1.00	0.00	N
ATOM	3254	H	SER	208	39.031	54.143	30.537	1.00	0.00	H
ATOM	3255	CA	SER	208	36.922	54.159	30.905	1.00	0.00	C
ATOM	3256	HA	SER	208	36.310	53.323	30.580	1.00	0.00	H
ATOM	3257	CB	SER	208	37.293	53.907	32.373	1.00	0.00	C
ATOM	3258	HB2	SER	208	36.393	53.606	32.910	1.00	0.00	H
ATOM	3259	HB3	SER	208	38.009	53.085	32.423	1.00	0.00	H
ATOM	3260	OG	SER	208	37.839	55.040	33.025	1.00	0.00	O
ATOM	3261	HG	SER	208	38.373	55.552	32.392	1.00	0.00	H
ATOM	3262	C	SER	208	36.037	55.401	30.751	1.00	0.00	C
ATOM	3263	O	SER	208	34.828	55.289	30.921	1.00	0.00	O
ATOM	3264	N	ASP	209	36.593	56.561	30.378	1.00	0.00	N
ATOM	3265	H	ASP	209	37.603	56.627	30.350	1.00	0.00	H
ATOM	3266	CA	ASP	209	35.783	57.752	30.075	1.00	0.00	C
ATOM	3267	HA	ASP	209	35.109	57.964	30.907	1.00	0.00	H
ATOM	3268	CB	ASP	209	36.674	58.975	29.814	1.00	0.00	C
ATOM	3269	HB2	ASP	209	37.352	58.719	29.001	1.00	0.00	H
ATOM	3270	HB3	ASP	209	36.031	59.786	29.470	1.00	0.00	H
ATOM	3271	CG	ASP	209	37.517	59.559	30.933	1.00	0.00	C
ATOM	3272	OD1	ASP	209	37.203	59.419	32.136	1.00	0.00	O
ATOM	3273	OD2	ASP	209	38.456	60.286	30.535	1.00	0.00	O

ATOM	3274	C	ASP	209	34.947	57.609	28.789	1.00	0.00	C
ATOM	3275	O	ASP	209	33.816	58.095	28.717	1.00	0.00	O
ATOM	3276	N	LYS	210	35.563	57.084	27.717	1.00	0.00	N
ATOM	3277	H	LYS	210	36.463	56.643	27.865	1.00	0.00	H
ATOM	3278	CA	LYS	210	35.142	57.391	26.335	1.00	0.00	C
ATOM	3279	HA	LYS	210	34.490	58.265	26.353	1.00	0.00	H
ATOM	3280	CB	LYS	210	36.369	57.731	25.459	1.00	0.00	C
ATOM	3281	HB2	LYS	210	36.937	56.815	25.291	1.00	0.00	H
ATOM	3282	HB3	LYS	210	36.007	58.067	24.485	1.00	0.00	H
ATOM	3283	CG	LYS	210	37.356	58.792	25.990	1.00	0.00	C
ATOM	3284	HG2	LYS	210	37.866	58.382	26.855	1.00	0.00	H
ATOM	3285	HG3	LYS	210	38.106	58.965	25.218	1.00	0.00	H
ATOM	3286	CD	LYS	210	36.738	60.143	26.385	1.00	0.00	C
ATOM	3287	HD2	LYS	210	36.151	60.514	25.543	1.00	0.00	H
ATOM	3288	HD3	LYS	210	36.058	60.010	27.224	1.00	0.00	H
ATOM	3289	CE	LYS	210	37.797	61.210	26.734	1.00	0.00	C
ATOM	3290	HE2	LYS	210	38.371	61.425	25.828	1.00	0.00	H
ATOM	3291	HE3	LYS	210	37.282	62.133	27.014	1.00	0.00	H
ATOM	3292	NZ	LYS	210	38.745	60.828	27.817	1.00	0.00	N
ATOM	3293	HZ1	LYS	210	39.237	59.976	27.596	1.00	0.00	H
ATOM	3294	HZ2	LYS	210	38.316	60.710	28.737	1.00	0.00	H
ATOM	3295	HZ3	LYS	210	39.436	61.570	27.929	1.00	0.00	H
ATOM	3296	C	LYS	210	34.313	56.290	25.665	1.00	0.00	C
ATOM	3297	O	LYS	210	33.622	56.581	24.686	1.00	0.00	O
ATOM	3298	N	MET	211	34.408	55.048	26.144	1.00	0.00	N
ATOM	3299	H	MET	211	34.922	54.910	27.003	1.00	0.00	H
ATOM	3300	CA	MET	211	34.038	53.852	25.373	1.00	0.00	C
ATOM	3301	HA	MET	211	33.734	54.181	24.382	1.00	0.00	H
ATOM	3302	CB	MET	211	35.274	52.958	25.190	1.00	0.00	C
ATOM	3303	HB2	MET	211	35.564	52.545	26.158	1.00	0.00	H
ATOM	3304	HB3	MET	211	35.021	52.126	24.533	1.00	0.00	H
ATOM	3305	CG	MET	211	36.477	53.714	24.604	1.00	0.00	C
ATOM	3306	HG2	MET	211	36.821	54.443	25.338	1.00	0.00	H
ATOM	3307	HG3	MET	211	37.292	53.011	24.469	1.00	0.00	H
ATOM	3308	SD	MET	211	36.209	54.586	23.031	1.00	0.00	S
ATOM	3309	CE	MET	211	35.730	53.230	21.926	1.00	0.00	C
ATOM	3310	HE1	MET	211	36.504	52.464	21.923	1.00	0.00	H
ATOM	3311	HE2	MET	211	34.785	52.799	22.255	1.00	0.00	H
ATOM	3312	HE3	MET	211	35.608	53.620	20.915	1.00	0.00	H
ATOM	3313	C	MET	211	32.872	53.058	25.992	1.00	0.00	C
ATOM	3314	O	MET	211	32.740	53.029	27.216	1.00	0.00	O
ATOM	3315	N	PRO	212	32.038	52.361	25.187	1.00	0.00	N
ATOM	3316	CD	PRO	212	31.984	52.395	23.729	1.00	0.00	C
ATOM	3317	HD2	PRO	212	32.714	51.695	23.319	1.00	0.00	H
ATOM	3318	HD3	PRO	212	32.154	53.396	23.331	1.00	0.00	H
ATOM	3319	CG	PRO	212	30.571	51.949	23.361	1.00	0.00	C
ATOM	3320	HG2	PRO	212	30.533	51.470	22.384	1.00	0.00	H
ATOM	3321	HG3	PRO	212	29.895	52.804	23.394	1.00	0.00	H
ATOM	3322	CB	PRO	212	30.220	50.972	24.478	1.00	0.00	C
ATOM	3323	HB2	PRO	212	30.644	49.994	24.243	1.00	0.00	H
ATOM	3324	HB3	PRO	212	29.142	50.897	24.622	1.00	0.00	H
ATOM	3325	CA	PRO	212	30.916	51.576	25.703	1.00	0.00	C

ATOM	3326	HA	PRO	212	30.228	52.264	26.195	1.00	0.00	H
ATOM	3327	C	PRO	212	31.330	50.481	26.696	1.00	0.00	C
ATOM	3328	O	PRO	212	32.341	49.806	26.516	1.00	0.00	O
ATOM	3329	N	LEU	213	30.479	50.229	27.694	1.00	0.00	N
ATOM	3330	H	LEU	213	29.680	50.840	27.757	1.00	0.00	H
ATOM	3331	CA	LEU	213	30.732	49.342	28.843	1.00	0.00	C
ATOM	3332	HA	LEU	213	31.504	49.793	29.471	1.00	0.00	H
ATOM	3333	CB	LEU	213	29.429	49.205	29.659	1.00	0.00	C
ATOM	3334	HB2	LEU	213	28.729	48.604	29.076	1.00	0.00	H
ATOM	3335	HB3	LEU	213	29.643	48.654	30.577	1.00	0.00	H
ATOM	3336	CG	LEU	213	28.722	50.523	30.020	1.00	0.00	C
ATOM	3337	HG	LEU	213	28.641	51.163	29.143	1.00	0.00	H
ATOM	3338	CD1	LEU	213	27.298	50.198	30.460	1.00	0.00	C
ATOM	3339	HD11	LEU	213	26.761	49.701	29.651	1.00	0.00	H
ATOM	3340	HD12	LEU	213	26.768	51.112	30.714	1.00	0.00	H
ATOM	3341	HD13	LEU	213	27.329	49.528	31.315	1.00	0.00	H
ATOM	3342	CD2	LEU	213	29.485	51.280	31.105	1.00	0.00	C
ATOM	3343	HD21	LEU	213	29.395	50.768	32.063	1.00	0.00	H
ATOM	3344	HD22	LEU	213	30.538	51.368	30.837	1.00	0.00	H
ATOM	3345	HD23	LEU	213	29.093	52.291	31.191	1.00	0.00	H
ATOM	3346	C	LEU	213	31.226	47.942	28.444	1.00	0.00	C
ATOM	3347	O	LEU	213	32.281	47.485	28.880	1.00	0.00	O
ATOM	3348	N	THR	214	30.490	47.268	27.554	1.00	0.00	N
ATOM	3349	H	THR	214	29.643	47.697	27.217	1.00	0.00	H
ATOM	3350	CA	THR	214	30.882	45.941	27.051	1.00	0.00	C
ATOM	3351	HA	THR	214	31.128	45.306	27.904	1.00	0.00	H
ATOM	3352	CB	THR	214	29.711	45.269	26.310	1.00	0.00	C
ATOM	3353	HB	THR	214	29.274	45.973	25.601	1.00	0.00	H
ATOM	3354	CG2	THR	214	30.074	43.984	25.561	1.00	0.00	C
ATOM	3355	HG21	THR	214	29.165	43.506	25.196	1.00	0.00	H
ATOM	3356	HG22	THR	214	30.595	43.298	26.230	1.00	0.00	H
ATOM	3357	HG23	THR	214	30.709	44.212	24.707	1.00	0.00	H
ATOM	3358	OG1	THR	214	28.736	44.888	27.253	1.00	0.00	O
ATOM	3359	HG1	THR	214	28.296	45.666	27.604	1.00	0.00	H
ATOM	3360	C	THR	214	32.147	46.008	26.189	1.00	0.00	C
ATOM	3361	O	THR	214	32.897	45.041	26.140	1.00	0.00	O
ATOM	3362	N	HID	215	32.447	47.135	25.536	1.00	0.00	N
ATOM	3363	H	HID	215	31.893	47.964	25.707	1.00	0.00	H
ATOM	3364	CA	HID	215	33.696	47.307	24.783	1.00	0.00	C
ATOM	3365	HA	HID	215	33.917	46.371	24.275	1.00	0.00	H
ATOM	3366	CB	HID	215	33.509	48.366	23.678	1.00	0.00	C
ATOM	3367	HB2	HID	215	33.169	49.304	24.116	1.00	0.00	H
ATOM	3368	HB3	HID	215	34.478	48.549	23.212	1.00	0.00	H
ATOM	3369	CG	HID	215	32.543	47.943	22.585	1.00	0.00	C
ATOM	3370	ND1	HID	215	32.207	48.652	21.447	1.00	0.00	N
ATOM	3371	HD1	HID	215	32.487	49.599	21.199	1.00	0.00	H
ATOM	3372	CE1	HID	215	31.414	47.874	20.687	1.00	0.00	C
ATOM	3373	HE1	HID	215	30.998	48.164	19.729	1.00	0.00	H
ATOM	3374	NE2	HID	215	31.212	46.681	21.277	1.00	0.00	N
ATOM	3375	CD2	HID	215	31.902	46.733	22.488	1.00	0.00	C
ATOM	3376	HD2	HID	215	31.950	45.930	23.206	1.00	0.00	H
ATOM	3377	C	HID	215	34.908	47.551	25.701	1.00	0.00	C

ATOM	3378	O	HID	215	36.010	47.115	25.369	1.00	0.00	O
ATOM	3379	N	LEU	216	34.704	48.129	26.891	1.00	0.00	N
ATOM	3380	H	LEU	216	33.792	48.527	27.087	1.00	0.00	H
ATOM	3381	CA	LEU	216	35.719	48.173	27.947	1.00	0.00	C
ATOM	3382	HA	LEU	216	36.647	48.513	27.488	1.00	0.00	H
ATOM	3383	CB	LEU	216	35.288	49.206	29.004	1.00	0.00	C
ATOM	3384	HB2	LEU	216	34.983	50.122	28.495	1.00	0.00	H
ATOM	3385	HB3	LEU	216	34.420	48.819	29.540	1.00	0.00	H
ATOM	3386	CG	LEU	216	36.377	49.562	30.034	1.00	0.00	C
ATOM	3387	HG	LEU	216	36.704	48.662	30.554	1.00	0.00	H
ATOM	3388	CD1	LEU	216	37.588	50.238	29.384	1.00	0.00	C
ATOM	3389	HD11	LEU	216	38.121	49.528	28.752	1.00	0.00	H
ATOM	3390	HD12	LEU	216	38.277	50.584	30.154	1.00	0.00	H
ATOM	3391	HD13	LEU	216	37.262	51.090	28.788	1.00	0.00	H
ATOM	3392	CD2	LEU	216	35.797	50.536	31.058	1.00	0.00	C
ATOM	3393	HD21	LEU	216	35.386	51.405	30.547	1.00	0.00	H
ATOM	3394	HD22	LEU	216	34.991	50.058	31.609	1.00	0.00	H
ATOM	3395	HD23	LEU	216	36.568	50.846	31.763	1.00	0.00	H
ATOM	3396	C	LEU	216	35.992	46.775	28.538	1.00	0.00	C
ATOM	3397	O	LEU	216	37.154	46.406	28.705	1.00	0.00	O
ATOM	3398	N	LEU	217	34.961	45.946	28.762	1.00	0.00	N
ATOM	3399	H	LEU	217	34.020	46.315	28.679	1.00	0.00	H
ATOM	3400	CA	LEU	217	35.151	44.533	29.138	1.00	0.00	C
ATOM	3401	HA	LEU	217	35.873	44.498	29.951	1.00	0.00	H
ATOM	3402	CB	LEU	217	33.840	43.897	29.629	1.00	0.00	C
ATOM	3403	HB2	LEU	217	33.039	44.152	28.935	1.00	0.00	H
ATOM	3404	HB3	LEU	217	33.955	42.811	29.615	1.00	0.00	H
ATOM	3405	CG	LEU	217	33.435	44.304	31.056	1.00	0.00	C
ATOM	3406	HG	LEU	217	33.312	45.384	31.098	1.00	0.00	H
ATOM	3407	CD1	LEU	217	32.103	43.646	31.394	1.00	0.00	C
ATOM	3408	HD11	LEU	217	31.339	43.979	30.694	1.00	0.00	H
ATOM	3409	HD12	LEU	217	31.820	43.920	32.409	1.00	0.00	H
ATOM	3410	HD13	LEU	217	32.198	42.559	31.339	1.00	0.00	H
ATOM	3411	CD2	LEU	217	34.440	43.863	32.128	1.00	0.00	C
ATOM	3412	HD21	LEU	217	34.592	42.784	32.069	1.00	0.00	H
ATOM	3413	HD22	LEU	217	35.389	44.376	32.006	1.00	0.00	H
ATOM	3414	HD23	LEU	217	34.048	44.104	33.117	1.00	0.00	H
ATOM	3415	C	LEU	217	35.774	43.677	28.024	1.00	0.00	C
ATOM	3416	O	LEU	217	36.689	42.910	28.313	1.00	0.00	O
ATOM	3417	N	LYS	218	35.399	43.859	26.746	1.00	0.00	N
ATOM	3418	H	LYS	218	34.592	44.448	26.570	1.00	0.00	H
ATOM	3419	CA	LYS	218	36.073	43.205	25.598	1.00	0.00	C
ATOM	3420	HA	LYS	218	35.960	42.123	25.695	1.00	0.00	H
ATOM	3421	CB	LYS	218	35.477	43.662	24.250	1.00	0.00	C
ATOM	3422	HB2	LYS	218	35.356	44.743	24.275	1.00	0.00	H
ATOM	3423	HB3	LYS	218	36.205	43.445	23.465	1.00	0.00	H
ATOM	3424	CG	LYS	218	34.159	43.000	23.810	1.00	0.00	C
ATOM	3425	HG2	LYS	218	34.301	41.922	23.721	1.00	0.00	H
ATOM	3426	HG3	LYS	218	33.376	43.190	24.541	1.00	0.00	H
ATOM	3427	CD	LYS	218	33.753	43.581	22.442	1.00	0.00	C
ATOM	3428	HD2	LYS	218	33.739	44.667	22.513	1.00	0.00	H
ATOM	3429	HD3	LYS	218	34.510	43.305	21.704	1.00	0.00	H

ATOM	3430	CE	LYS	218	32.386	43.093	21.944	1.00	0.00	C
ATOM	3431	HE2	LYS	218	32.457	42.022	21.728	1.00	0.00	H
ATOM	3432	HE3	LYS	218	31.639	43.228	22.732	1.00	0.00	H
ATOM	3433	NZ	LYS	218	31.975	43.830	20.720	1.00	0.00	N
ATOM	3434	HZ1	LYS	218	31.189	43.389	20.244	1.00	0.00	H
ATOM	3435	HZ2	LYS	218	32.738	43.861	20.043	1.00	0.00	H
ATOM	3436	HZ3	LYS	218	31.719	44.796	20.910	1.00	0.00	H
ATOM	3437	C	LYS	218	37.583	43.484	25.559	1.00	0.00	C
ATOM	3438	O	LYS	218	38.311	42.728	24.914	1.00	0.00	O
ATOM	3439	N	LEU	219	38.055	44.562	26.184	1.00	0.00	N
ATOM	3440	H	LEU	219	37.385	45.171	26.633	1.00	0.00	H
ATOM	3441	CA	LEU	219	39.475	44.866	26.362	1.00	0.00	C
ATOM	3442	HA	LEU	219	40.044	44.415	25.547	1.00	0.00	H
ATOM	3443	CB	LEU	219	39.627	46.392	26.244	1.00	0.00	C
ATOM	3444	HB2	LEU	219	39.244	46.667	25.260	1.00	0.00	H
ATOM	3445	HB3	LEU	219	38.994	46.891	26.978	1.00	0.00	H
ATOM	3446	CG	LEU	219	41.066	46.925	26.377	1.00	0.00	C
ATOM	3447	HG	LEU	219	41.771	46.200	25.969	1.00	0.00	H
ATOM	3448	CD1	LEU	219	41.182	48.216	25.571	1.00	0.00	C
ATOM	3449	HD11	LEU	219	41.000	48.007	24.518	1.00	0.00	H
ATOM	3450	HD12	LEU	219	42.182	48.639	25.664	1.00	0.00	H
ATOM	3451	HD13	LEU	219	40.451	48.942	25.925	1.00	0.00	H
ATOM	3452	CD2	LEU	219	41.434	47.247	27.826	1.00	0.00	C
ATOM	3453	HD21	LEU	219	40.687	47.904	28.272	1.00	0.00	H
ATOM	3454	HD22	LEU	219	41.507	46.333	28.411	1.00	0.00	H
ATOM	3455	HD23	LEU	219	42.403	47.743	27.856	1.00	0.00	H
ATOM	3456	C	LEU	219	40.028	44.262	27.665	1.00	0.00	C
ATOM	3457	O	LEU	219	41.051	43.580	27.633	1.00	0.00	O
ATOM	3458	N	LEU	220	39.348	44.476	28.795	1.00	0.00	N
ATOM	3459	H	LEU	220	38.511	45.048	28.749	1.00	0.00	H
ATOM	3460	CA	LEU	220	39.814	44.054	30.119	1.00	0.00	C
ATOM	3461	HA	LEU	220	40.850	44.381	30.218	1.00	0.00	H
ATOM	3462	CB	LEU	220	38.971	44.779	31.183	1.00	0.00	C
ATOM	3463	HB2	LEU	220	39.008	45.852	30.993	1.00	0.00	H
ATOM	3464	HB3	LEU	220	37.934	44.455	31.085	1.00	0.00	H
ATOM	3465	CG	LEU	220	39.431	44.515	32.628	1.00	0.00	C
ATOM	3466	HG	LEU	220	39.385	43.448	32.843	1.00	0.00	H
ATOM	3467	CD1	LEU	220	40.852	45.019	32.875	1.00	0.00	C
ATOM	3468	HD11	LEU	220	41.569	44.417	32.318	1.00	0.00	H
ATOM	3469	HD12	LEU	220	41.090	44.916	33.931	1.00	0.00	H
ATOM	3470	HD13	LEU	220	40.943	46.066	32.590	1.00	0.00	H
ATOM	3471	CD2	LEU	220	38.496	45.236	33.598	1.00	0.00	C
ATOM	3472	HD21	LEU	220	38.529	46.310	33.423	1.00	0.00	H
ATOM	3473	HD22	LEU	220	37.478	44.869	33.473	1.00	0.00	H
ATOM	3474	HD23	LEU	220	38.822	45.032	34.618	1.00	0.00	H
ATOM	3475	C	LEU	220	39.811	42.528	30.316	1.00	0.00	C
ATOM	3476	O	LEU	220	40.824	41.971	30.731	1.00	0.00	O
ATOM	3477	N	GLU	221	38.716	41.839	29.973	1.00	0.00	N
ATOM	3478	H	GLU	221	37.925	42.340	29.579	1.00	0.00	H
ATOM	3479	CA	GLU	221	38.650	40.366	29.998	1.00	0.00	C
ATOM	3480	HA	GLU	221	38.892	40.005	31.000	1.00	0.00	H
ATOM	3481	CB	GLU	221	37.243	39.851	29.617	1.00	0.00	C

ATOM	3482	HB2	GLU	221	36.999	40.183	28.606	1.00	0.00	H
ATOM	3483	HB3	GLU	221	37.287	38.761	29.604	1.00	0.00	H
ATOM	3484	CG	GLU	221	36.104	40.270	30.564	1.00	0.00	C
ATOM	3485	HG2	GLU	221	36.405	40.059	31.593	1.00	0.00	H
ATOM	3486	HG3	GLU	221	35.937	41.345	30.480	1.00	0.00	H
ATOM	3487	CD	GLU	221	34.803	39.502	30.255	1.00	0.00	C
ATOM	3488	OE1	GLU	221	33.851	40.085	29.686	1.00	0.00	O
ATOM	3489	OE2	GLU	221	34.717	38.296	30.589	1.00	0.00	O
ATOM	3490	C	GLU	221	39.682	39.760	29.035	1.00	0.00	C
ATOM	3491	O	GLU	221	40.334	38.774	29.363	1.00	0.00	O
ATOM	3492	N	GLN	222	39.893	40.378	27.865	1.00	0.00	N
ATOM	3493	H	GLN	222	39.361	41.212	27.667	1.00	0.00	H
ATOM	3494	CA	GLN	222	40.869	39.900	26.882	1.00	0.00	C
ATOM	3495	HA	GLN	222	40.644	38.851	26.684	1.00	0.00	H
ATOM	3496	CB	GLN	222	40.703	40.689	25.571	1.00	0.00	C
ATOM	3497	HB2	GLN	222	39.691	40.525	25.200	1.00	0.00	H
ATOM	3498	HB3	GLN	222	40.820	41.752	25.781	1.00	0.00	H
ATOM	3499	CG	GLN	222	41.692	40.319	24.453	1.00	0.00	C
ATOM	3500	HG2	GLN	222	41.536	40.998	23.615	1.00	0.00	H
ATOM	3501	HG3	GLN	222	42.718	40.447	24.794	1.00	0.00	H
ATOM	3502	CD	GLN	222	41.493	38.894	23.951	1.00	0.00	C
ATOM	3503	OE1	GLN	222	40.819	38.659	22.962	1.00	0.00	O
ATOM	3504	NE2	GLN	222	42.039	37.903	24.621	1.00	0.00	N
ATOM	3505	HE21	GLN	222	41.836	36.972	24.301	1.00	0.00	H
ATOM	3506	HE22	GLN	222	42.563	38.081	25.471	1.00	0.00	H
ATOM	3507	C	GLN	222	42.309	39.961	27.415	1.00	0.00	C
ATOM	3508	O	GLN	222	43.070	39.016	27.203	1.00	0.00	O
ATOM	3509	N	PHE	223	42.675	41.042	28.113	1.00	0.00	N
ATOM	3510	H	PHE	223	42.001	41.792	28.223	1.00	0.00	H
ATOM	3511	CA	PHE	223	43.963	41.174	28.799	1.00	0.00	C
ATOM	3512	HA	PHE	223	44.767	40.973	28.090	1.00	0.00	H
ATOM	3513	CB	PHE	223	44.100	42.622	29.298	1.00	0.00	C
ATOM	3514	HB2	PHE	223	44.173	43.284	28.434	1.00	0.00	H
ATOM	3515	HB3	PHE	223	43.190	42.896	29.835	1.00	0.00	H
ATOM	3516	CG	PHE	223	45.290	42.883	30.205	1.00	0.00	C
ATOM	3517	CD1	PHE	223	46.599	42.668	29.734	1.00	0.00	C
ATOM	3518	HD1	PHE	223	46.765	42.310	28.729	1.00	0.00	H
ATOM	3519	CE1	PHE	223	47.699	42.912	30.576	1.00	0.00	C
ATOM	3520	HE1	PHE	223	48.703	42.736	30.221	1.00	0.00	H
ATOM	3521	CZ	PHE	223	47.498	43.382	31.885	1.00	0.00	C
ATOM	3522	HZ	PHE	223	48.345	43.575	32.530	1.00	0.00	H
ATOM	3523	CE2	PHE	223	46.193	43.601	32.356	1.00	0.00	C
ATOM	3524	HE2	PHE	223	46.040	43.975	33.359	1.00	0.00	H
ATOM	3525	CD2	PHE	223	45.091	43.345	31.522	1.00	0.00	C
ATOM	3526	HD2	PHE	223	44.092	43.518	31.897	1.00	0.00	H
ATOM	3527	C	PHE	223	44.106	40.157	29.942	1.00	0.00	C
ATOM	3528	O	PHE	223	45.075	39.398	29.986	1.00	0.00	O
ATOM	3529	N	PHE	224	43.114	40.091	30.833	1.00	0.00	N
ATOM	3530	H	PHE	224	42.331	40.731	30.734	1.00	0.00	H
ATOM	3531	CA	PHE	224	43.117	39.201	31.996	1.00	0.00	C
ATOM	3532	HA	PHE	224	44.064	39.309	32.518	1.00	0.00	H
ATOM	3533	CB	PHE	224	41.968	39.621	32.925	1.00	0.00	C

```
ATOM   3534  HB2 PHE   224      41.066  39.754  32.324  1.00  0.00           H
ATOM   3535  HB3 PHE   224      41.755  38.800  33.605  1.00  0.00           H
ATOM   3536  CG  PHE   224      42.182  40.869  33.774  1.00  0.00           C
ATOM   3537  CD1 PHE   224      43.457  41.453  33.968  1.00  0.00           C
ATOM   3538  HD1 PHE   224      44.336  41.081  33.466  1.00  0.00           H
ATOM   3539  CE1 PHE   224      43.615  42.544  34.835  1.00  0.00           C
ATOM   3540  HE1 PHE   224      44.590  42.988  34.979  1.00  0.00           H
ATOM   3541  CZ  PHE   224      42.504  43.068  35.511  1.00  0.00           C
ATOM   3542  HZ  PHE   224      42.622  43.904  36.182  1.00  0.00           H
ATOM   3543  CE2 PHE   224      41.234  42.521  35.298  1.00  0.00           C
ATOM   3544  HE2 PHE   224      40.387  42.973  35.785  1.00  0.00           H
ATOM   3545  CD2 PHE   224      41.072  41.419  34.440  1.00  0.00           C
ATOM   3546  HD2 PHE   224      40.092  40.990  34.291  1.00  0.00           H
ATOM   3547  C   PHE   224      43.041  37.706  31.666  1.00  0.00           C
ATOM   3548  O   PHE   224      43.526  36.902  32.465  1.00  0.00           O
ATOM   3549  N   GLU   225      42.485  37.315  30.519  1.00  0.00           N
ATOM   3550  H   GLU   225      41.962  37.999  29.978  1.00  0.00           H
ATOM   3551  CA  GLU   225      42.522  35.934  30.028  1.00  0.00           C
ATOM   3552  HA  GLU   225      42.365  35.267  30.874  1.00  0.00           H
ATOM   3553  CB  GLU   225      41.351  35.711  29.052  1.00  0.00           C
ATOM   3554  HB2 GLU   225      40.441  36.039  29.558  1.00  0.00           H
ATOM   3555  HB3 GLU   225      41.485  36.326  28.161  1.00  0.00           H
ATOM   3556  CG  GLU   225      41.162  34.243  28.638  1.00  0.00           C
ATOM   3557  HG2 GLU   225      41.743  34.044  27.734  1.00  0.00           H
ATOM   3558  HG3 GLU   225      41.545  33.596  29.431  1.00  0.00           H
ATOM   3559  CD  GLU   225      39.678  33.907  28.416  1.00  0.00           C
ATOM   3560  OE1 GLU   225      39.180  34.021  27.274  1.00  0.00           O
ATOM   3561  OE2 GLU   225      39.000  33.522  29.402  1.00  0.00           O
ATOM   3562  C   GLU   225      43.897  35.566  29.441  1.00  0.00           C
ATOM   3563  O   GLU   225      44.430  34.513  29.792  1.00  0.00           O
ATOM   3564  N   ILE   226      44.526  36.455  28.653  1.00  0.00           N
ATOM   3565  H   ILE   226      44.050  37.323  28.436  1.00  0.00           H
ATOM   3566  CA  ILE   226      45.895  36.254  28.131  1.00  0.00           C
ATOM   3567  HA  ILE   226      45.923  35.310  27.584  1.00  0.00           H
ATOM   3568  CB  ILE   226      46.294  37.393  27.156  1.00  0.00           C
ATOM   3569  HB  ILE   226      46.050  38.346  27.628  1.00  0.00           H
ATOM   3570  CG2 ILE   226      47.808  37.391  26.846  1.00  0.00           C
ATOM   3571 HG21 ILE   226      48.386  37.590  27.749  1.00  0.00           H
ATOM   3572 HG22 ILE   226      48.060  38.170  26.130  1.00  0.00           H
ATOM   3573 HG23 ILE   226      48.106  36.422  26.443  1.00  0.00           H
ATOM   3574  CG1 ILE   226      45.499  37.275  25.835  1.00  0.00           C
ATOM   3575 HG12 ILE   226      45.894  36.448  25.244  1.00  0.00           H
ATOM   3576 HG13 ILE   226      44.457  37.048  26.059  1.00  0.00           H
ATOM   3577  CD1 ILE   226      45.520  38.557  24.988  1.00  0.00           C
ATOM   3578 HD11 ILE   226      45.138  39.396  25.568  1.00  0.00           H
ATOM   3579 HD12 ILE   226      44.893  38.419  24.107  1.00  0.00           H
ATOM   3580 HD13 ILE   226      46.532  38.786  24.660  1.00  0.00           H
ATOM   3581  C   ILE   226      46.899  36.119  29.284  1.00  0.00           C
ATOM   3582  O   ILE   226      47.653  35.147  29.336  1.00  0.00           O
ATOM   3583  N   VAL   227      46.881  37.059  30.237  1.00  0.00           N
ATOM   3584  H   VAL   227      46.235  37.837  30.134  1.00  0.00           H
ATOM   3585  CA  VAL   227      47.780  37.033  31.402  1.00  0.00           C
```

ATOM	3586	HA	VAL	227	48.809	37.003	31.044	1.00	0.00	H
ATOM	3587	CB	VAL	227	47.607	38.314	32.248	1.00	0.00	C
ATOM	3588	HB	VAL	227	46.546	38.463	32.455	1.00	0.00	H
ATOM	3589	CG1	VAL	227	48.351	38.265	33.590	1.00	0.00	C
ATOM	3590	HG11	VAL	227	49.406	38.041	33.428	1.00	0.00	H
ATOM	3591	HG12	VAL	227	48.266	39.227	34.097	1.00	0.00	H
ATOM	3592	HG13	VAL	227	47.916	37.504	34.236	1.00	0.00	H
ATOM	3593	CG2	VAL	227	48.140	39.534	31.486	1.00	0.00	C
ATOM	3594	HG21	VAL	227	47.622	39.647	30.534	1.00	0.00	H
ATOM	3595	HG22	VAL	227	49.208	39.426	31.300	1.00	0.00	H
ATOM	3596	HG23	VAL	227	47.970	40.436	32.074	1.00	0.00	H
ATOM	3597	C	VAL	227	47.575	35.763	32.236	1.00	0.00	C
ATOM	3598	O	VAL	227	48.554	35.176	32.692	1.00	0.00	O
ATOM	3599	N	CYS	228	46.333	35.300	32.415	1.00	0.00	N
ATOM	3600	H	CYS	228	45.565	35.789	31.976	1.00	0.00	H
ATOM	3601	CA	CYS	228	46.035	34.102	33.204	1.00	0.00	C
ATOM	3602	HA	CYS	228	46.566	34.172	34.153	1.00	0.00	H
ATOM	3603	CB	CYS	228	44.528	34.069	33.488	1.00	0.00	C
ATOM	3604	HB2	CYS	228	44.210	35.033	33.874	1.00	0.00	H
ATOM	3605	HB3	CYS	228	43.983	33.865	32.564	1.00	0.00	H
ATOM	3606	SG	CYS	228	44.140	32.793	34.718	1.00	0.00	S
ATOM	3607	HG	CYS	228	44.716	33.396	35.763	1.00	0.00	H
ATOM	3608	C	CYS	228	46.494	32.795	32.535	1.00	0.00	C
ATOM	3609	O	CYS	228	47.100	31.948	33.200	1.00	0.00	O
ATOM	3610	N	ASP	229	46.208	32.613	31.241	1.00	0.00	N
ATOM	3611	H	ASP	229	45.705	33.336	30.732	1.00	0.00	H
ATOM	3612	CA	ASP	229	46.538	31.368	30.536	1.00	0.00	C
ATOM	3613	HA	ASP	229	46.218	30.536	31.160	1.00	0.00	H
ATOM	3614	CB	ASP	229	45.766	31.286	29.205	1.00	0.00	C
ATOM	3615	HB2	ASP	229	44.757	31.671	29.362	1.00	0.00	H
ATOM	3616	HB3	ASP	229	46.256	31.919	28.462	1.00	0.00	H
ATOM	3617	CG	ASP	229	45.625	29.854	28.663	1.00	0.00	C
ATOM	3618	OD1	ASP	229	45.471	28.892	29.457	1.00	0.00	O
ATOM	3619	OD2	ASP	229	45.634	29.668	27.424	1.00	0.00	O
ATOM	3620	C	ASP	229	48.053	31.216	30.337	1.00	0.00	C
ATOM	3621	O	ASP	229	48.580	30.115	30.474	1.00	0.00	O
ATOM	3622	N	GLU	230	48.770	32.314	30.079	1.00	0.00	N
ATOM	3623	H	GLU	230	48.284	33.196	29.947	1.00	0.00	H
ATOM	3624	CA	GLU	230	50.232	32.302	29.983	1.00	0.00	C
ATOM	3625	HA	GLU	230	50.538	31.411	29.436	1.00	0.00	H
ATOM	3626	CB	GLU	230	50.701	33.532	29.189	1.00	0.00	C
ATOM	3627	HB2	GLU	230	50.299	34.438	29.646	1.00	0.00	H
ATOM	3628	HB3	GLU	230	51.780	33.597	29.255	1.00	0.00	H
ATOM	3629	CG	GLU	230	50.310	33.494	27.697	1.00	0.00	C
ATOM	3630	HG2	GLU	230	49.244	33.717	27.613	1.00	0.00	H
ATOM	3631	HG3	GLU	230	50.850	34.287	27.176	1.00	0.00	H
ATOM	3632	CD	GLU	230	50.592	32.148	27.004	1.00	0.00	C
ATOM	3633	OE1	GLU	230	51.706	31.597	27.167	1.00	0.00	O
ATOM	3634	OE2	GLU	230	49.677	31.635	26.309	1.00	0.00	O
ATOM	3635	C	GLU	230	50.923	32.167	31.350	1.00	0.00	C
ATOM	3636	O	GLU	230	51.783	31.302	31.492	1.00	0.00	O
ATOM	3637	N	THR	231	50.504	32.903	32.392	1.00	0.00	N

ATOM 3638 H THR 231 49.770 33.585 32.243 1.00 0.00 H
ATOM 3639 CA THR 231 51.096 32.769 33.746 1.00 0.00 C
ATOM 3640 HA THR 231 52.139 33.085 33.706 1.00 0.00 H
ATOM 3641 CB THR 231 50.368 33.651 34.777 1.00 0.00 C
ATOM 3642 HB THR 231 49.304 33.408 34.769 1.00 0.00 H
ATOM 3643 CG2 THR 231 50.886 33.482 36.204 1.00 0.00 C
ATOM 3644 HG21 THR 231 51.970 33.605 36.222 1.00 0.00 H
ATOM 3645 HG22 THR 231 50.618 32.498 36.580 1.00 0.00 H
ATOM 3646 HG23 THR 231 50.431 34.231 36.847 1.00 0.00 H
ATOM 3647 OG1 THR 231 50.514 35.014 34.470 1.00 0.00 O
ATOM 3648 HG1 THR 231 49.913 35.190 33.725 1.00 0.00 H
ATOM 3649 C THR 231 51.073 31.318 34.240 1.00 0.00 C
ATOM 3650 O THR 231 52.066 30.829 34.779 1.00 0.00 O
ATOM 3651 N GLU 232 49.957 30.611 34.023 1.00 0.00 N
ATOM 3652 H GLU 232 49.187 31.067 33.546 1.00 0.00 H
ATOM 3653 CA GLU 232 49.798 29.202 34.399 1.00 0.00 C
ATOM 3654 HA GLU 232 50.026 29.091 35.461 1.00 0.00 H
ATOM 3655 CB GLU 232 48.333 28.799 34.167 1.00 0.00 C
ATOM 3656 HB2 GLU 232 47.692 29.443 34.771 1.00 0.00 H
ATOM 3657 HB3 GLU 232 48.093 28.967 33.116 1.00 0.00 H
ATOM 3658 CG GLU 232 48.033 27.335 34.526 1.00 0.00 C
ATOM 3659 HG2 GLU 232 48.679 26.673 33.948 1.00 0.00 H
ATOM 3660 HG3 GLU 232 48.248 27.183 35.587 1.00 0.00 H
ATOM 3661 CD GLU 232 46.578 26.952 34.233 1.00 0.00 C
ATOM 3662 OE1 GLU 232 46.026 27.427 33.207 1.00 0.00 O
ATOM 3663 OE2 GLU 232 46.003 26.165 35.020 1.00 0.00 O
ATOM 3664 C GLU 232 50.757 28.277 33.631 1.00 0.00 C
ATOM 3665 O GLU 232 51.474 27.485 34.250 1.00 0.00 O
ATOM 3666 N LYS 233 50.826 28.403 32.296 1.00 0.00 N
ATOM 3667 H LYS 233 50.232 29.096 31.849 1.00 0.00 H
ATOM 3668 CA LYS 233 51.771 27.642 31.452 1.00 0.00 C
ATOM 3669 HA LYS 233 51.578 26.573 31.552 1.00 0.00 H
ATOM 3670 CB LYS 233 51.622 28.060 29.980 1.00 0.00 C
ATOM 3671 HB2 LYS 233 51.720 29.146 29.924 1.00 0.00 H
ATOM 3672 HB3 LYS 233 52.433 27.621 29.395 1.00 0.00 H
ATOM 3673 CG LYS 233 50.291 27.633 29.346 1.00 0.00 C
ATOM 3674 HG2 LYS 233 50.311 26.564 29.133 1.00 0.00 H
ATOM 3675 HG3 LYS 233 49.480 27.821 30.047 1.00 0.00 H
ATOM 3676 CD LYS 233 50.042 28.417 28.048 1.00 0.00 C
ATOM 3677 HD2 LYS 233 50.345 29.455 28.189 1.00 0.00 H
ATOM 3678 HD3 LYS 233 50.625 27.988 27.232 1.00 0.00 H
ATOM 3679 CE LYS 233 48.552 28.405 27.712 1.00 0.00 C
ATOM 3680 HE2 LYS 233 48.229 27.389 27.471 1.00 0.00 H
ATOM 3681 HE3 LYS 233 48.011 28.723 28.609 1.00 0.00 H
ATOM 3682 NZ LYS 233 48.232 29.337 26.606 1.00 0.00 N
ATOM 3683 HZ1 LYS 233 47.233 29.550 26.627 1.00 0.00 H
ATOM 3684 HZ2 LYS 233 48.479 28.958 25.707 1.00 0.00 H
ATOM 3685 HZ3 LYS 233 48.735 30.223 26.706 1.00 0.00 H
ATOM 3686 C LYS 233 53.215 27.882 31.887 1.00 0.00 C
ATOM 3687 O LYS 233 54.039 26.973 31.853 1.00 0.00 O
ATOM 3688 N HIE 234 53.524 29.107 32.311 1.00 0.00 N
ATOM 3689 H HIE 234 52.798 29.817 32.252 1.00 0.00 H

```
ATOM   3690  CA  HIE   234      54.855  29.531  32.745  1.00  0.00           C
ATOM   3691  HA  HIE   234      55.591  28.945  32.192  1.00  0.00           H
ATOM   3692  CB  HIE   234      55.078  31.009  32.368  1.00  0.00           C
ATOM   3693  HB2 HIE   234      54.474  31.621  33.040  1.00  0.00           H
ATOM   3694  HB3 HIE   234      56.124  31.256  32.551  1.00  0.00           H
ATOM   3695  CG  HIE   234      54.743  31.434  30.944  1.00  0.00           C
ATOM   3696  ND1 HIE   234      54.662  32.768  30.548  1.00  0.00           N
ATOM   3697  CE1 HIE   234      54.195  32.765  29.293  1.00  0.00           C
ATOM   3698  HE1 HIE   234      53.941  33.654  28.731  1.00  0.00           H
ATOM   3699  NE2 HIE   234      53.966  31.513  28.877  1.00  0.00           N
ATOM   3700  HE2 HIE   234      53.370  31.291  28.072  1.00  0.00           H
ATOM   3701  CD2 HIE   234      54.354  30.655  29.885  1.00  0.00           C
ATOM   3702  HD2 HIE   234      54.253  29.583  29.879  1.00  0.00           H
ATOM   3703  C   HIE   234      55.111  29.228  34.238  1.00  0.00           C
ATOM   3704  O   HIE   234      56.051  29.767  34.826  1.00  0.00           O
ATOM   3705  N   SER   235      54.327  28.331  34.858  1.00  0.00           N
ATOM   3706  H   SER   235      53.533  27.983  34.330  1.00  0.00           H
ATOM   3707  CA  SER   235      54.462  27.806  36.236  1.00  0.00           C
ATOM   3708  HA  SER   235      53.645  27.093  36.350  1.00  0.00           H
ATOM   3709  CB  SER   235      55.756  26.988  36.394  1.00  0.00           C
ATOM   3710  HB2 SER   235      55.728  26.437  37.335  1.00  0.00           H
ATOM   3711  HB3 SER   235      55.827  26.269  35.576  1.00  0.00           H
ATOM   3712  OG  SER   235      56.896  27.830  36.379  1.00  0.00           O
ATOM   3713  HG  SER   235      56.669  28.559  35.765  1.00  0.00           H
ATOM   3714  C   SER   235      54.241  28.818  37.376  1.00  0.00           C
ATOM   3715  O   SER   235      54.702  28.597  38.498  1.00  0.00           O
ATOM   3716  N   GLY   236      53.545  29.922  37.096  1.00  0.00           N
ATOM   3717  H   GLY   236      53.152  30.017  36.164  1.00  0.00           H
ATOM   3718  CA  GLY   236      53.100  30.901  38.090  1.00  0.00           C
ATOM   3719  HA2 GLY   236      53.694  30.817  39.001  1.00  0.00           H
ATOM   3720  HA3 GLY   236      53.244  31.905  37.691  1.00  0.00           H
ATOM   3721  C   GLY   236      51.622  30.736  38.448  1.00  0.00           C
ATOM   3722  O   GLY   236      50.914  29.896  37.888  1.00  0.00           O
ATOM   3723  N   LYS   237      51.126  31.572  39.366  1.00  0.00           N
ATOM   3724  H   LYS   237      51.780  32.193  39.837  1.00  0.00           H
ATOM   3725  CA  LYS   237      49.710  31.588  39.769  1.00  0.00           C
ATOM   3726  HA  LYS   237      49.119  31.169  38.953  1.00  0.00           H
ATOM   3727  CB  LYS   237      49.535  30.688  41.002  1.00  0.00           C
ATOM   3728  HB2 LYS   237      50.065  29.747  40.839  1.00  0.00           H
ATOM   3729  HB3 LYS   237      49.957  31.178  41.881  1.00  0.00           H
ATOM   3730  CG  LYS   237      48.054  30.374  41.234  1.00  0.00           C
ATOM   3731  HG2 LYS   237      47.487  31.297  41.344  1.00  0.00           H
ATOM   3732  HG3 LYS   237      47.677  29.838  40.363  1.00  0.00           H
ATOM   3733  CD  LYS   237      47.846  29.527  42.489  1.00  0.00           C
ATOM   3734  HD2 LYS   237      48.522  28.672  42.468  1.00  0.00           H
ATOM   3735  HD3 LYS   237      48.046  30.133  43.374  1.00  0.00           H
ATOM   3736  CE  LYS   237      46.398  29.035  42.490  1.00  0.00           C
ATOM   3737  HE2 LYS   237      45.731  29.880  42.298  1.00  0.00           H
ATOM   3738  HE3 LYS   237      46.280  28.325  41.668  1.00  0.00           H
ATOM   3739  NZ  LYS   237      46.023  28.384  43.762  1.00  0.00           N
ATOM   3740  HZ1 LYS   237      46.028  29.065  44.513  1.00  0.00           H
ATOM   3741  HZ2 LYS   237      46.673  27.622  43.965  1.00  0.00           H
```

ATOM	3742	HZ3	LYS	237	45.079	28.020	43.678	1.00	0.00	H
ATOM	3743	C	LYS	237	49.202	33.001	40.039	1.00	0.00	C
ATOM	3744	O	LYS	237	49.842	33.751	40.774	1.00	0.00	O
ATOM	3745	N	LEU	238	48.033	33.356	39.496	1.00	0.00	N
ATOM	3746	H	LEU	238	47.541	32.683	38.932	1.00	0.00	H
ATOM	3747	CA	LEU	238	47.350	34.606	39.855	1.00	0.00	C
ATOM	3748	HA	LEU	238	48.050	35.432	39.733	1.00	0.00	H
ATOM	3749	CB	LEU	238	46.135	34.886	38.952	1.00	0.00	C
ATOM	3750	HB2	LEU	238	45.391	34.103	39.109	1.00	0.00	H
ATOM	3751	HB3	LEU	238	45.696	35.825	39.294	1.00	0.00	H
ATOM	3752	CG	LEU	238	46.400	35.005	37.439	1.00	0.00	C
ATOM	3753	HG	LEU	238	46.577	34.013	37.022	1.00	0.00	H
ATOM	3754	CD1	LEU	238	45.167	35.614	36.775	1.00	0.00	C
ATOM	3755	HD11	LEU	238	44.295	34.982	36.946	1.00	0.00	H
ATOM	3756	HD12	LEU	238	45.333	35.717	35.705	1.00	0.00	H
ATOM	3757	HD13	LEU	238	44.971	36.603	37.190	1.00	0.00	H
ATOM	3758	CD2	LEU	238	47.585	35.902	37.090	1.00	0.00	C
ATOM	3759	HD21	LEU	238	47.472	36.882	37.555	1.00	0.00	H
ATOM	3760	HD22	LEU	238	48.505	35.435	37.424	1.00	0.00	H
ATOM	3761	HD23	LEU	238	47.657	36.018	36.009	1.00	0.00	H
ATOM	3762	C	LEU	238	46.918	34.592	41.331	1.00	0.00	C
ATOM	3763	O	LEU	238	46.355	33.611	41.817	1.00	0.00	O
ATOM	3764	N	GLY	239	47.168	35.698	42.032	1.00	0.00	N
ATOM	3765	H	GLY	239	47.628	36.465	41.553	1.00	0.00	H
ATOM	3766	CA	GLY	239	46.756	35.903	43.421	1.00	0.00	C
ATOM	3767	HA2	GLY	239	46.545	34.945	43.897	1.00	0.00	H
ATOM	3768	HA3	GLY	239	47.574	36.373	43.962	1.00	0.00	H
ATOM	3769	C	GLY	239	45.520	36.793	43.559	1.00	0.00	C
ATOM	3770	O	GLY	239	44.668	36.502	44.394	1.00	0.00	O
ATOM	3771	N	THR	240	45.409	37.845	42.737	1.00	0.00	N
ATOM	3772	H	THR	240	46.160	38.017	42.078	1.00	0.00	H
ATOM	3773	CA	THR	240	44.238	38.741	42.631	1.00	0.00	C
ATOM	3774	HA	THR	240	43.333	38.146	42.753	1.00	0.00	H
ATOM	3775	CB	THR	240	44.219	39.860	43.701	1.00	0.00	C
ATOM	3776	HB	THR	240	43.420	40.556	43.442	1.00	0.00	H
ATOM	3777	CG2	THR	240	43.964	39.387	45.129	1.00	0.00	C
ATOM	3778	HG21	THR	240	44.814	38.822	45.508	1.00	0.00	H
ATOM	3779	HG22	THR	240	43.069	38.766	45.158	1.00	0.00	H
ATOM	3780	HG23	THR	240	43.810	40.255	45.770	1.00	0.00	H
ATOM	3781	OG1	THR	240	45.428	40.577	43.741	1.00	0.00	O
ATOM	3782	HG1	THR	240	46.047	40.046	44.251	1.00	0.00	H
ATOM	3783	C	THR	240	44.180	39.410	41.249	1.00	0.00	C
ATOM	3784	O	THR	240	45.214	39.715	40.655	1.00	0.00	O
ATOM	3785	N	TYR	241	42.966	39.678	40.756	1.00	0.00	N
ATOM	3786	H	TYR	241	42.166	39.382	41.292	1.00	0.00	H
ATOM	3787	CA	TYR	241	42.710	40.775	39.814	1.00	0.00	C
ATOM	3788	HA	TYR	241	43.623	41.039	39.278	1.00	0.00	H
ATOM	3789	CB	TYR	241	41.628	40.399	38.789	1.00	0.00	C
ATOM	3790	HB2	TYR	241	40.692	40.223	39.322	1.00	0.00	H
ATOM	3791	HB3	TYR	241	41.471	41.268	38.150	1.00	0.00	H
ATOM	3792	CG	TYR	241	41.900	39.221	37.870	1.00	0.00	C
ATOM	3793	CD1	TYR	241	41.088	38.071	37.937	1.00	0.00	C

ATOM	3794	HD1	TYR	241	40.335	37.994	38.706	1.00	0.00	H
ATOM	3795	CE1	TYR	241	41.224	37.048	36.976	1.00	0.00	C
ATOM	3796	HE1	TYR	241	40.586	36.177	36.996	1.00	0.00	H
ATOM	3797	CZ	TYR	241	42.180	37.175	35.945	1.00	0.00	C
ATOM	3798	OH	TYR	241	42.249	36.227	34.974	1.00	0.00	O
ATOM	3799	HH	TYR	241	42.746	36.527	34.198	1.00	0.00	H
ATOM	3800	CE2	TYR	241	43.029	38.303	35.909	1.00	0.00	C
ATOM	3801	HE2	TYR	241	43.763	38.400	35.124	1.00	0.00	H
ATOM	3802	CD2	TYR	241	42.878	39.329	36.863	1.00	0.00	C
ATOM	3803	HD2	TYR	241	43.479	40.226	36.791	1.00	0.00	H
ATOM	3804	C	TYR	241	42.231	41.996	40.615	1.00	0.00	C
ATOM	3805	O	TYR	241	41.452	41.830	41.557	1.00	0.00	O
ATOM	3806	N	LEU	242	42.637	43.209	40.236	1.00	0.00	N
ATOM	3807	H	LEU	242	43.280	43.284	39.452	1.00	0.00	H
ATOM	3808	CA	LEU	242	42.210	44.443	40.896	1.00	0.00	C
ATOM	3809	HA	LEU	242	41.208	44.280	41.293	1.00	0.00	H
ATOM	3810	CB	LEU	242	43.153	44.743	42.079	1.00	0.00	C
ATOM	3811	HB2	LEU	242	43.332	43.838	42.663	1.00	0.00	H
ATOM	3812	HB3	LEU	242	44.112	45.081	41.684	1.00	0.00	H
ATOM	3813	CG	LEU	242	42.537	45.828	42.979	1.00	0.00	C
ATOM	3814	HG	LEU	242	42.029	46.552	42.348	1.00	0.00	H
ATOM	3815	CD1	LEU	242	41.533	45.201	43.943	1.00	0.00	C
ATOM	3816	HD11	LEU	242	40.673	44.831	43.390	1.00	0.00	H
ATOM	3817	HD12	LEU	242	41.197	45.941	44.667	1.00	0.00	H
ATOM	3818	HD13	LEU	242	41.992	44.374	44.487	1.00	0.00	H
ATOM	3819	CD2	LEU	242	43.559	46.598	43.806	1.00	0.00	C
ATOM	3820	HD21	LEU	242	43.705	46.093	44.754	1.00	0.00	H
ATOM	3821	HD22	LEU	242	44.505	46.662	43.273	1.00	0.00	H
ATOM	3822	HD23	LEU	242	43.175	47.594	44.019	1.00	0.00	H
ATOM	3823	C	LEU	242	42.131	45.623	39.914	1.00	0.00	C
ATOM	3824	O	LEU	242	43.147	46.202	39.526	1.00	0.00	O
ATOM	3825	N	GLY	243	40.910	46.014	39.543	1.00	0.00	N
ATOM	3826	H	GLY	243	40.111	45.496	39.876	1.00	0.00	H
ATOM	3827	CA	GLY	243	40.686	47.067	38.553	1.00	0.00	C
ATOM	3828	HA2	GLY	243	39.621	47.275	38.471	1.00	0.00	H
ATOM	3829	HA3	GLY	243	41.203	47.971	38.868	1.00	0.00	H
ATOM	3830	C	GLY	243	41.211	46.665	37.178	1.00	0.00	C
ATOM	3831	O	GLY	243	40.761	45.672	36.615	1.00	0.00	O
ATOM	3832	N	ASP	244	42.170	47.429	36.656	1.00	0.00	N
ATOM	3833	H	ASP	244	42.487	48.232	37.180	1.00	0.00	H
ATOM	3834	CA	ASP	244	42.892	47.123	35.414	1.00	0.00	C
ATOM	3835	HA	ASP	244	42.207	46.637	34.720	1.00	0.00	H
ATOM	3836	CB	ASP	244	43.369	48.433	34.771	1.00	0.00	C
ATOM	3837	HB2	ASP	244	43.579	48.238	33.721	1.00	0.00	H
ATOM	3838	HB3	ASP	244	42.566	49.168	34.813	1.00	0.00	H
ATOM	3839	CG	ASP	244	44.624	49.023	35.425	1.00	0.00	C
ATOM	3840	OD1	ASP	244	44.796	48.962	36.681	1.00	0.00	O
ATOM	3841	OD2	ASP	244	45.471	49.595	34.696	1.00	0.00	O
ATOM	3842	C	ASP	244	44.107	46.190	35.600	1.00	0.00	C
ATOM	3843	O	ASP	244	44.780	45.877	34.618	1.00	0.00	O
ATOM	3844	N	GLY	245	44.448	45.823	36.842	1.00	0.00	N
ATOM	3845	H	GLY	245	43.845	46.094	37.609	1.00	0.00	H

ATOM	3846	CA	GLY	245	45.702	45.146	37.177	1.00	0.00	C
ATOM	3847	HA2	GLY	245	46.349	45.158	36.304	1.00	0.00	H
ATOM	3848	HA3	GLY	245	46.209	45.698	37.968	1.00	0.00	H
ATOM	3849	C	GLY	245	45.558	43.693	37.625	1.00	0.00	C
ATOM	3850	O	GLY	245	44.512	43.276	38.127	1.00	0.00	O
ATOM	3851	N	ALA	246	46.638	42.927	37.479	1.00	0.00	N
ATOM	3852	H	ALA	246	47.458	43.348	37.047	1.00	0.00	H
ATOM	3853	CA	ALA	246	46.746	41.541	37.925	1.00	0.00	C
ATOM	3854	HA	ALA	246	45.885	41.298	38.549	1.00	0.00	H
ATOM	3855	CB	ALA	246	46.703	40.615	36.702	1.00	0.00	C
ATOM	3856	HB1	ALA	246	47.556	40.817	36.053	1.00	0.00	H
ATOM	3857	HB2	ALA	246	45.783	40.782	36.141	1.00	0.00	H
ATOM	3858	HB3	ALA	246	46.738	39.575	37.027	1.00	0.00	H
ATOM	3859	C	ALA	246	48.010	41.329	38.774	1.00	0.00	C
ATOM	3860	O	ALA	246	49.114	41.709	38.378	1.00	0.00	O
ATOM	3861	N	MET	247	47.840	40.694	39.937	1.00	0.00	N
ATOM	3862	H	MET	247	46.898	40.392	40.172	1.00	0.00	H
ATOM	3863	CA	MET	247	48.917	40.232	40.815	1.00	0.00	C
ATOM	3864	HA	MET	247	49.840	40.747	40.552	1.00	0.00	H
ATOM	3865	CB	MET	247	48.568	40.581	42.271	1.00	0.00	C
ATOM	3866	HB2	MET	247	48.428	41.658	42.347	1.00	0.00	H
ATOM	3867	HB3	MET	247	47.624	40.106	42.529	1.00	0.00	H
ATOM	3868	CG	MET	247	49.623	40.156	43.303	1.00	0.00	C
ATOM	3869	HG2	MET	247	50.622	40.284	42.884	1.00	0.00	H
ATOM	3870	HG3	MET	247	49.536	40.827	44.157	1.00	0.00	H
ATOM	3871	SD	MET	247	49.434	38.459	43.924	1.00	0.00	S
ATOM	3872	CE	MET	247	50.523	38.521	45.373	1.00	0.00	C
ATOM	3873	HE1	MET	247	50.543	37.545	45.858	1.00	0.00	H
ATOM	3874	HE2	MET	247	51.532	38.791	45.064	1.00	0.00	H
ATOM	3875	HE3	MET	247	50.149	39.257	46.082	1.00	0.00	H
ATOM	3876	C	MET	247	49.129	38.726	40.632	1.00	0.00	C
ATOM	3877	O	MET	247	48.158	37.962	40.622	1.00	0.00	O
ATOM	3878	N	CYS	248	50.380	38.276	40.533	1.00	0.00	N
ATOM	3879	H	CYS	248	51.149	38.940	40.592	1.00	0.00	H
ATOM	3880	CA	CYS	248	50.707	36.850	40.461	1.00	0.00	C
ATOM	3881	HA	CYS	248	49.944	36.311	41.023	1.00	0.00	H
ATOM	3882	CB	CYS	248	50.612	36.370	39.000	1.00	0.00	C
ATOM	3883	HB2	CYS	248	50.170	35.379	38.991	1.00	0.00	H
ATOM	3884	HB3	CYS	248	49.961	37.031	38.425	1.00	0.00	H
ATOM	3885	SG	CYS	248	52.215	36.222	38.182	1.00	0.00	S
ATOM	3886	HG	CYS	248	52.494	37.526	38.108	1.00	0.00	H
ATOM	3887	C	CYS	248	52.032	36.501	41.156	1.00	0.00	C
ATOM	3888	O	CYS	248	52.833	37.386	41.468	1.00	0.00	O
ATOM	3889	N	ILE	249	52.239	35.208	41.420	1.00	0.00	N
ATOM	3890	H	ILE	249	51.539	34.540	41.113	1.00	0.00	H
ATOM	3891	CA	ILE	249	53.373	34.686	42.191	1.00	0.00	C
ATOM	3892	HA	ILE	249	54.138	35.460	42.211	1.00	0.00	H
ATOM	3893	CB	ILE	249	52.968	34.412	43.665	1.00	0.00	C
ATOM	3894	HB	ILE	249	53.895	34.254	44.220	1.00	0.00	H
ATOM	3895	CG2	ILE	249	52.266	35.634	44.289	1.00	0.00	C
ATOM	3896	HG21	ILE	249	52.791	36.546	44.007	1.00	0.00	H
ATOM	3897	HG22	ILE	249	52.272	35.556	45.375	1.00	0.00	H

ATOM	3898	HG23	ILE	249	51.236	35.701	43.938	1.00	0.00	H
ATOM	3899	CG1	ILE	249	52.106	33.137	43.833	1.00	0.00	C
ATOM	3900	HG12	ILE	249	51.194	33.235	43.244	1.00	0.00	H
ATOM	3901	HG13	ILE	249	52.660	32.277	43.459	1.00	0.00	H
ATOM	3902	CD1	ILE	249	51.721	32.822	45.286	1.00	0.00	C
ATOM	3903	HD11	ILE	249	52.616	32.791	45.909	1.00	0.00	H
ATOM	3904	HD12	ILE	249	51.228	31.851	45.324	1.00	0.00	H
ATOM	3905	HD13	ILE	249	51.033	33.575	45.670	1.00	0.00	H
ATOM	3906	C	ILE	249	54.016	33.451	41.538	1.00	0.00	C
ATOM	3907	O	ILE	249	53.340	32.643	40.899	1.00	0.00	O
ATOM	3908	N	TRP	250	55.311	33.276	41.800	1.00	0.00	N
ATOM	3909	H	TRP	250	55.768	34.022	42.317	1.00	0.00	H
ATOM	3910	CA	TRP	250	56.065	32.021	41.700	1.00	0.00	C
ATOM	3911	HA	TRP	250	55.380	31.189	41.531	1.00	0.00	H
ATOM	3912	CB	TRP	250	57.091	32.061	40.549	1.00	0.00	C
ATOM	3913	HB2	TRP	250	57.788	32.875	40.733	1.00	0.00	H
ATOM	3914	HB3	TRP	250	57.670	31.144	40.600	1.00	0.00	H
ATOM	3915	CG	TRP	250	56.618	32.182	39.133	1.00	0.00	C
ATOM	3916	CD1	TRP	250	56.619	31.175	38.229	1.00	0.00	C
ATOM	3917	HD1	TRP	250	56.914	30.153	38.432	1.00	0.00	H
ATOM	3918	NE1	TRP	250	56.178	31.641	37.009	1.00	0.00	N
ATOM	3919	HE1	TRP	250	56.065	31.051	36.186	1.00	0.00	H
ATOM	3920	CE2	TRP	250	55.823	32.965	37.073	1.00	0.00	C
ATOM	3921	CZ2	TRP	250	55.280	33.846	36.131	1.00	0.00	C
ATOM	3922	HZ2	TRP	250	55.023	33.497	35.141	1.00	0.00	H
ATOM	3923	CH2	TRP	250	55.065	35.183	36.495	1.00	0.00	C
ATOM	3924	HH2	TRP	250	54.634	35.873	35.778	1.00	0.00	H
ATOM	3925	CZ3	TRP	250	55.384	35.610	37.796	1.00	0.00	C
ATOM	3926	HZ3	TRP	250	55.186	36.631	38.079	1.00	0.00	H
ATOM	3927	CE3	TRP	250	55.900	34.709	38.747	1.00	0.00	C
ATOM	3928	HE3	TRP	250	56.109	35.046	39.752	1.00	0.00	H
ATOM	3929	CD2	TRP	250	56.125	33.356	38.413	1.00	0.00	C
ATOM	3930	C	TRP	250	56.820	31.779	43.022	1.00	0.00	C
ATOM	3931	O	TRP	250	56.965	32.687	43.840	1.00	0.00	O
ATOM	3932	N	GLU	251	57.400	30.593	43.214	1.00	0.00	N
ATOM	3933	H	GLU	251	57.246	29.868	42.532	1.00	0.00	H
ATOM	3934	CA	GLU	251	58.481	30.396	44.198	1.00	0.00	C
ATOM	3935	HA	GLU	251	58.107	30.605	45.204	1.00	0.00	H
ATOM	3936	CB	GLU	251	58.968	28.936	44.137	1.00	0.00	C
ATOM	3937	HB2	GLU	251	59.162	28.688	43.091	1.00	0.00	H
ATOM	3938	HB3	GLU	251	59.920	28.845	44.661	1.00	0.00	H
ATOM	3939	CG	GLU	251	57.963	27.910	44.698	1.00	0.00	C
ATOM	3940	HG2	GLU	251	56.951	28.175	44.383	1.00	0.00	H
ATOM	3941	HG3	GLU	251	58.194	26.940	44.252	1.00	0.00	H
ATOM	3942	CD	GLU	251	58.029	27.750	46.226	1.00	0.00	C
ATOM	3943	OE1	GLU	251	59.162	27.672	46.758	1.00	0.00	O
ATOM	3944	OE2	GLU	251	56.954	27.654	46.864	1.00	0.00	O
ATOM	3945	C	GLU	251	59.668	31.346	43.916	1.00	0.00	C
ATOM	3946	O	GLU	251	59.924	31.704	42.761	1.00	0.00	O
ATOM	3947	N	ALA	252	60.454	31.727	44.932	1.00	0.00	N
ATOM	3948	H	ALA	252	60.203	31.482	45.887	1.00	0.00	H
ATOM	3949	CA	ALA	252	61.700	32.484	44.706	1.00	0.00	C

ATOM	3950	HA	ALA	252	61.468	33.395	44.152	1.00	0.00	H
ATOM	3951	CB	ALA	252	62.306	32.895	46.048	1.00	0.00	C
ATOM	3952	HB1	ALA	252	61.653	33.615	46.536	1.00	0.00	H
ATOM	3953	HB2	ALA	252	63.272	33.374	45.880	1.00	0.00	H
ATOM	3954	HB3	ALA	252	62.448	32.020	46.683	1.00	0.00	H
ATOM	3955	C	ALA	252	62.721	31.692	43.865	1.00	0.00	C
ATOM	3956	O	ALA	252	63.521	32.269	43.136	1.00	0.00	O
ATOM	3957	N	SER	253	62.640	30.359	43.884	1.00	0.00	N
ATOM	3958	H	SER	253	62.003	29.934	44.542	1.00	0.00	H
ATOM	3959	CA	SER	253	63.419	29.460	43.022	1.00	0.00	C
ATOM	3960	HA	SER	253	64.480	29.653	43.183	1.00	0.00	H
ATOM	3961	CB	SER	253	63.112	28.006	43.416	1.00	0.00	C
ATOM	3962	HB2	SER	253	62.118	27.741	43.052	1.00	0.00	H
ATOM	3963	HB3	SER	253	63.840	27.340	42.951	1.00	0.00	H
ATOM	3964	OG	SER	253	63.133	27.834	44.828	1.00	0.00	O
ATOM	3965	HG	SER	253	64.041	27.959	45.134	1.00	0.00	H
ATOM	3966	C	SER	253	63.135	29.636	41.516	1.00	0.00	C
ATOM	3967	O	SER	253	63.860	29.084	40.692	1.00	0.00	O
ATOM	3968	N	HID	254	62.076	30.368	41.146	1.00	0.00	N
ATOM	3969	H	HID	254	61.541	30.821	41.878	1.00	0.00	H
ATOM	3970	CA	HID	254	61.527	30.473	39.789	1.00	0.00	C
ATOM	3971	HA	HID	254	62.172	29.917	39.112	1.00	0.00	H
ATOM	3972	CB	HID	254	60.139	29.795	39.763	1.00	0.00	C
ATOM	3973	HB2	HID	254	59.601	30.067	40.669	1.00	0.00	H
ATOM	3974	HB3	HID	254	59.562	30.169	38.921	1.00	0.00	H
ATOM	3975	CG	HID	254	60.143	28.284	39.652	1.00	0.00	C
ATOM	3976	ND1	HID	254	59.022	27.482	39.603	1.00	0.00	N
ATOM	3977	HD1	HID	254	58.060	27.789	39.648	1.00	0.00	H
ATOM	3978	CE1	HID	254	59.413	26.218	39.369	1.00	0.00	C
ATOM	3979	HE1	HID	254	58.745	25.371	39.263	1.00	0.00	H
ATOM	3980	NE2	HID	254	60.750	26.159	39.240	1.00	0.00	N
ATOM	3981	CD2	HID	254	61.219	27.463	39.426	1.00	0.00	C
ATOM	3982	HD2	HID	254	62.251	27.771	39.337	1.00	0.00	H
ATOM	3983	C	HID	254	61.560	31.905	39.207	1.00	0.00	C
ATOM	3984	O	HID	254	60.784	32.237	38.310	1.00	0.00	O
ATOM	3985	N	VAL	255	62.520	32.740	39.630	1.00	0.00	N
ATOM	3986	H	VAL	255	63.077	32.464	40.429	1.00	0.00	H
ATOM	3987	CA	VAL	255	62.779	34.065	39.021	1.00	0.00	C
ATOM	3988	HA	VAL	255	61.898	34.689	39.172	1.00	0.00	H
ATOM	3989	CB	VAL	255	63.968	34.754	39.727	1.00	0.00	C
ATOM	3990	HB	VAL	255	64.800	34.051	39.778	1.00	0.00	H
ATOM	3991	CG1	VAL	255	64.457	36.017	39.016	1.00	0.00	C
ATOM	3992	HG11	VAL	255	63.648	36.742	38.924	1.00	0.00	H
ATOM	3993	HG12	VAL	255	65.275	36.457	39.586	1.00	0.00	H
ATOM	3994	HG13	VAL	255	64.838	35.779	38.026	1.00	0.00	H
ATOM	3995	CG2	VAL	255	63.586	35.180	41.149	1.00	0.00	C
ATOM	3996	HG21	VAL	255	62.791	35.924	41.120	1.00	0.00	H
ATOM	3997	HG22	VAL	255	64.453	35.601	41.657	1.00	0.00	H
ATOM	3998	HG23	VAL	255	63.238	34.323	41.720	1.00	0.00	H
ATOM	3999	C	VAL	255	62.996	33.975	37.498	1.00	0.00	C
ATOM	4000	O	VAL	255	62.468	34.804	36.757	1.00	0.00	O
ATOM	4001	N	ARG	256	63.685	32.928	37.004	1.00	0.00	N

ATOM	4002	H	ARG	256	64.085	32.283	37.668	1.00	0.00	H
ATOM	4003	CA	ARG	256	63.839	32.667	35.554	1.00	0.00	C
ATOM	4004	HA	ARG	256	64.249	33.567	35.094	1.00	0.00	H
ATOM	4005	CB	ARG	256	64.833	31.509	35.284	1.00	0.00	C
ATOM	4006	HB2	ARG	256	65.027	31.483	34.211	1.00	0.00	H
ATOM	4007	HB3	ARG	256	65.774	31.737	35.785	1.00	0.00	H
ATOM	4008	CG	ARG	256	64.380	30.102	35.721	1.00	0.00	C
ATOM	4009	HG2	ARG	256	64.144	30.118	36.786	1.00	0.00	H
ATOM	4010	HG3	ARG	256	63.490	29.826	35.159	1.00	0.00	H
ATOM	4011	CD	ARG	256	65.447	29.023	35.462	1.00	0.00	C
ATOM	4012	HD2	ARG	256	65.613	28.938	34.386	1.00	0.00	H
ATOM	4013	HD3	ARG	256	66.382	29.327	35.937	1.00	0.00	H
ATOM	4014	NE	ARG	256	65.035	27.712	36.007	1.00	0.00	N
ATOM	4015	HE	ARG	256	64.147	27.670	36.483	1.00	0.00	H
ATOM	4016	CZ	ARG	256	65.752	26.596	36.012	1.00	0.00	C
ATOM	4017	NH1	ARG	256	66.885	26.478	35.379	1.00	0.00	N
ATOM	4018	HH11	ARG	256	67.201	27.182	34.724	1.00	0.00	H
ATOM	4019	HH12	ARG	256	67.389	25.602	35.379	1.00	0.00	H
ATOM	4020	NH2	ARG	256	65.340	25.545	36.660	1.00	0.00	N
ATOM	4021	HH21	ARG	256	64.439	25.516	37.128	1.00	0.00	H
ATOM	4022	HH22	ARG	256	65.942	24.731	36.717	1.00	0.00	H
ATOM	4023	C	ARG	256	62.495	32.467	34.845	1.00	0.00	C
ATOM	4024	O	ARG	256	62.280	33.011	33.768	1.00	0.00	O
ATOM	4025	N	ASN	257	61.577	31.735	35.472	1.00	0.00	N
ATOM	4026	H	ASN	257	61.820	31.325	36.362	1.00	0.00	H
ATOM	4027	CA	ASN	257	60.236	31.462	34.964	1.00	0.00	C
ATOM	4028	HA	ASN	257	60.318	31.071	33.948	1.00	0.00	H
ATOM	4029	CB	ASN	257	59.528	30.402	35.849	1.00	0.00	C
ATOM	4030	HB2	ASN	257	59.143	30.886	36.744	1.00	0.00	H
ATOM	4031	HB3	ASN	257	58.665	30.038	35.295	1.00	0.00	H
ATOM	4032	CG	ASN	257	60.352	29.198	36.313	1.00	0.00	C
ATOM	4033	OD1	ASN	257	61.513	29.280	36.691	1.00	0.00	O
ATOM	4034	ND2	ASN	257	59.767	28.028	36.374	1.00	0.00	N
ATOM	4035	HD21	ASN	257	58.772	27.934	36.200	1.00	0.00	H
ATOM	4036	HD22	ASN	257	60.279	27.281	36.808	1.00	0.00	H
ATOM	4037	C	ASN	257	59.423	32.769	34.897	1.00	0.00	C
ATOM	4038	O	ASN	257	58.788	33.049	33.884	1.00	0.00	O
ATOM	4039	N	SER	258	59.512	33.596	35.945	1.00	0.00	N
ATOM	4040	H	SER	258	60.047	33.277	36.746	1.00	0.00	H
ATOM	4041	CA	SER	258	58.823	34.887	36.048	1.00	0.00	C
ATOM	4042	HA	SER	258	57.761	34.722	35.884	1.00	0.00	H
ATOM	4043	CB	SER	258	59.013	35.429	37.466	1.00	0.00	C
ATOM	4044	HB2	SER	258	58.730	34.665	38.191	1.00	0.00	H
ATOM	4045	HB3	SER	258	60.062	35.685	37.620	1.00	0.00	H
ATOM	4046	OG	SER	258	58.222	36.577	37.669	1.00	0.00	O
ATOM	4047	HG	SER	258	57.297	36.293	37.636	1.00	0.00	H
ATOM	4048	C	SER	258	59.289	35.914	35.008	1.00	0.00	C
ATOM	4049	O	SER	258	58.467	36.518	34.319	1.00	0.00	O
ATOM	4050	N	ILE	259	60.605	36.076	34.827	1.00	0.00	N
ATOM	4051	H	ILE	259	61.243	35.566	35.430	1.00	0.00	H
ATOM	4052	CA	ILE	259	61.167	37.011	33.838	1.00	0.00	C
ATOM	4053	HA	ILE	259	60.639	37.962	33.932	1.00	0.00	H

ATOM	4054	CB	ILE	259	62.659	37.277	34.150	1.00	0.00	C
ATOM	4055	HB	ILE	259	63.155	36.322	34.337	1.00	0.00	H
ATOM	4056	CG2	ILE	259	63.383	37.968	32.980	1.00	0.00	C
ATOM	4057	HG21	ILE	259	63.387	37.326	32.098	1.00	0.00	H
ATOM	4058	HG22	ILE	259	64.421	38.162	33.238	1.00	0.00	H
ATOM	4059	HG23	ILE	259	62.889	38.909	32.735	1.00	0.00	H
ATOM	4060	CG1	ILE	259	62.733	38.147	35.429	1.00	0.00	C
ATOM	4061	HG12	ILE	259	62.294	39.124	35.225	1.00	0.00	H
ATOM	4062	HG13	ILE	259	62.147	37.677	36.220	1.00	0.00	H
ATOM	4063	CD1	ILE	259	64.144	38.354	35.984	1.00	0.00	C
ATOM	4064	HD11	ILE	259	64.618	37.389	36.156	1.00	0.00	H
ATOM	4065	HD12	ILE	259	64.074	38.886	36.931	1.00	0.00	H
ATOM	4066	HD13	ILE	259	64.748	38.950	35.301	1.00	0.00	H
ATOM	4067	C	ILE	259	60.909	36.535	32.400	1.00	0.00	C
ATOM	4068	O	ILE	259	60.531	37.348	31.553	1.00	0.00	O
ATOM	4069	N	ASN	260	61.013	35.228	32.124	1.00	0.00	N
ATOM	4070	H	ASN	260	61.380	34.596	32.826	1.00	0.00	H
ATOM	4071	CA	ASN	260	60.579	34.671	30.839	1.00	0.00	C
ATOM	4072	HA	ASN	260	61.131	35.164	30.035	1.00	0.00	H
ATOM	4073	CB	ASN	260	60.866	33.158	30.781	1.00	0.00	C
ATOM	4074	HB2	ASN	260	60.529	32.678	31.698	1.00	0.00	H
ATOM	4075	HB3	ASN	260	60.293	32.736	29.955	1.00	0.00	H
ATOM	4076	CG	ASN	260	62.325	32.797	30.542	1.00	0.00	C
ATOM	4077	OD1	ASN	260	63.243	33.593	30.641	1.00	0.00	O
ATOM	4078	ND2	ASN	260	62.588	31.567	30.163	1.00	0.00	N
ATOM	4079	HD21	ASN	260	61.849	30.902	30.026	1.00	0.00	H
ATOM	4080	HD22	ASN	260	63.547	31.364	29.953	1.00	0.00	H
ATOM	4081	C	ASN	260	59.087	34.952	30.595	1.00	0.00	C
ATOM	4082	O	ASN	260	58.719	35.366	29.497	1.00	0.00	O
ATOM	4083	N	SER	261	58.241	34.788	31.618	1.00	0.00	N
ATOM	4084	H	SER	261	58.599	34.416	32.490	1.00	0.00	H
ATOM	4085	CA	SER	261	56.802	35.043	31.525	1.00	0.00	C
ATOM	4086	HA	SER	261	56.425	34.457	30.692	1.00	0.00	H
ATOM	4087	CB	SER	261	56.105	34.580	32.806	1.00	0.00	C
ATOM	4088	HB2	SER	261	56.393	33.552	33.023	1.00	0.00	H
ATOM	4089	HB3	SER	261	56.405	35.212	33.641	1.00	0.00	H
ATOM	4090	OG	SER	261	54.704	34.640	32.659	1.00	0.00	O
ATOM	4091	HG	SER	261	54.468	34.016	31.946	1.00	0.00	H
ATOM	4092	C	SER	261	56.470	36.504	31.218	1.00	0.00	C
ATOM	4093	O	SER	261	55.655	36.768	30.338	1.00	0.00	O
ATOM	4094	N	ALA	262	57.171	37.459	31.836	1.00	0.00	N
ATOM	4095	H	ALA	262	57.807	37.184	32.577	1.00	0.00	H
ATOM	4096	CA	ALA	262	57.046	38.883	31.518	1.00	0.00	C
ATOM	4097	HA	ALA	262	56.021	39.198	31.715	1.00	0.00	H
ATOM	4098	CB	ALA	262	57.990	39.656	32.446	1.00	0.00	C
ATOM	4099	HB1	ALA	262	59.029	39.445	32.197	1.00	0.00	H
ATOM	4100	HB2	ALA	262	57.809	39.355	33.476	1.00	0.00	H
ATOM	4101	HB3	ALA	262	57.808	40.727	32.343	1.00	0.00	H
ATOM	4102	C	ALA	262	57.354	39.184	30.039	1.00	0.00	C
ATOM	4103	O	ALA	262	56.596	39.886	29.365	1.00	0.00	O
ATOM	4104	N	CYS	263	58.439	38.607	29.511	1.00	0.00	N
ATOM	4105	H	CYS	263	59.021	38.040	30.116	1.00	0.00	H

ATOM	4106	CA	CYS	263	58.829	38.759	28.107	1.00	0.00	C
ATOM	4107	HA	CYS	263	58.867	39.822	27.864	1.00	0.00	H
ATOM	4108	CB	CYS	263	60.232	38.164	27.902	1.00	0.00	C
ATOM	4109	HB2	CYS	263	60.224	37.097	28.129	1.00	0.00	H
ATOM	4110	HB3	CYS	263	60.524	38.298	26.859	1.00	0.00	H
ATOM	4111	SG	CYS	263	61.451	39.000	28.962	1.00	0.00	S
ATOM	4112	HG	CYS	263	61.091	38.423	30.121	1.00	0.00	H
ATOM	4113	C	CYS	263	57.809	38.111	27.154	1.00	0.00	C
ATOM	4114	O	CYS	263	57.434	38.708	26.144	1.00	0.00	O
ATOM	4115	N	LEU	264	57.327	36.908	27.482	1.00	0.00	N
ATOM	4116	H	LEU	264	57.678	36.466	28.327	1.00	0.00	H
ATOM	4117	CA	LEU	264	56.320	36.189	26.697	1.00	0.00	C
ATOM	4118	HA	LEU	264	56.635	36.177	25.654	1.00	0.00	H
ATOM	4119	CB	LEU	264	56.218	34.738	27.204	1.00	0.00	C
ATOM	4120	HB2	LEU	264	56.083	34.766	28.285	1.00	0.00	H
ATOM	4121	HB3	LEU	264	55.333	34.270	26.769	1.00	0.00	H
ATOM	4122	CG	LEU	264	57.446	33.866	26.871	1.00	0.00	C
ATOM	4123	HG	LEU	264	58.361	34.398	27.123	1.00	0.00	H
ATOM	4124	CD1	LEU	264	57.406	32.570	27.680	1.00	0.00	C
ATOM	4125	HD11	LEU	264	57.384	32.803	28.745	1.00	0.00	H
ATOM	4126	HD12	LEU	264	58.293	31.973	27.469	1.00	0.00	H
ATOM	4127	HD13	LEU	264	56.515	31.995	27.426	1.00	0.00	H
ATOM	4128	CD2	LEU	264	57.501	33.499	25.385	1.00	0.00	C
ATOM	4129	HD21	LEU	264	56.591	32.973	25.096	1.00	0.00	H
ATOM	4130	HD22	LEU	264	57.608	34.396	24.778	1.00	0.00	H
ATOM	4131	HD23	LEU	264	58.360	32.853	25.200	1.00	0.00	H
ATOM	4132	C	LEU	264	54.954	36.889	26.723	1.00	0.00	C
ATOM	4133	O	LEU	264	54.307	36.970	25.683	1.00	0.00	O
ATOM	4134	N	ALA	265	54.538	37.456	27.859	1.00	0.00	N
ATOM	4135	H	ALA	265	55.090	37.317	28.699	1.00	0.00	H
ATOM	4136	CA	ALA	265	53.330	38.270	27.966	1.00	0.00	C
ATOM	4137	HA	ALA	265	52.482	37.686	27.602	1.00	0.00	H
ATOM	4138	CB	ALA	265	53.087	38.605	29.443	1.00	0.00	C
ATOM	4139	HB1	ALA	265	52.977	37.683	30.017	1.00	0.00	H
ATOM	4140	HB2	ALA	265	52.174	39.193	29.541	1.00	0.00	H
ATOM	4141	HB3	ALA	265	53.926	39.175	29.845	1.00	0.00	H
ATOM	4142	C	ALA	265	53.429	39.536	27.100	1.00	0.00	C
ATOM	4143	O	ALA	265	52.503	39.834	26.347	1.00	0.00	O
ATOM	4144	N	ALA	266	54.566	40.242	27.129	1.00	0.00	N
ATOM	4145	H	ALA	266	55.291	39.972	27.788	1.00	0.00	H
ATOM	4146	CA	ALA	266	54.812	41.391	26.257	1.00	0.00	C
ATOM	4147	HA	ALA	266	54.017	42.121	26.418	1.00	0.00	H
ATOM	4148	CB	ALA	266	56.143	42.035	26.665	1.00	0.00	C
ATOM	4149	HB1	ALA	266	56.322	42.922	26.057	1.00	0.00	H
ATOM	4150	HB2	ALA	266	56.963	41.331	26.519	1.00	0.00	H
ATOM	4151	HB3	ALA	266	56.107	42.325	27.716	1.00	0.00	H
ATOM	4152	C	ALA	266	54.788	41.010	24.761	1.00	0.00	C
ATOM	4153	O	ALA	266	54.166	41.709	23.960	1.00	0.00	O
ATOM	4154	N	PHE	267	55.401	39.884	24.379	1.00	0.00	N
ATOM	4155	H	PHE	267	55.938	39.370	25.073	1.00	0.00	H
ATOM	4156	CA	PHE	267	55.388	39.375	23.001	1.00	0.00	C
ATOM	4157	HA	PHE	267	55.709	40.176	22.333	1.00	0.00	H

ATOM	4158	CB	PHE	267	56.403	38.225	22.888	1.00	0.00	C
ATOM	4159	HB2	PHE	267	57.354	38.557	23.307	1.00	0.00	H
ATOM	4160	HB3	PHE	267	56.061	37.382	23.489	1.00	0.00	H
ATOM	4161	CG	PHE	267	56.650	37.751	21.466	1.00	0.00	C
ATOM	4162	CD1	PHE	267	57.708	38.301	20.716	1.00	0.00	C
ATOM	4163	HD1	PHE	267	58.353	39.051	21.156	1.00	0.00	H
ATOM	4164	CE1	PHE	267	57.928	37.886	19.390	1.00	0.00	C
ATOM	4165	HE1	PHE	267	58.739	38.317	18.819	1.00	0.00	H
ATOM	4166	CZ	PHE	267	57.087	36.922	18.806	1.00	0.00	C
ATOM	4167	HZ	PHE	267	57.251	36.609	17.784	1.00	0.00	H
ATOM	4168	CE2	PHE	267	56.037	36.361	19.556	1.00	0.00	C
ATOM	4169	HE2	PHE	267	55.394	35.616	19.109	1.00	0.00	H
ATOM	4170	CD2	PHE	267	55.822	36.771	20.885	1.00	0.00	C
ATOM	4171	HD2	PHE	267	55.010	36.342	21.455	1.00	0.00	H
ATOM	4172	C	PHE	267	53.980	38.938	22.548	1.00	0.00	C
ATOM	4173	O	PHE	267	53.554	39.249	21.433	1.00	0.00	O
ATOM	4174	N	ALA	268	53.224	38.265	23.420	1.00	0.00	N
ATOM	4175	H	ALA	268	53.631	37.997	24.311	1.00	0.00	H
ATOM	4176	CA	ALA	268	51.836	37.886	23.168	1.00	0.00	C
ATOM	4177	HA	ALA	268	51.792	37.313	22.240	1.00	0.00	H
ATOM	4178	CB	ALA	268	51.345	36.990	24.313	1.00	0.00	C
ATOM	4179	HB1	ALA	268	51.973	36.100	24.382	1.00	0.00	H
ATOM	4180	HB2	ALA	268	50.317	36.680	24.124	1.00	0.00	H
ATOM	4181	HB3	ALA	268	51.387	37.528	25.260	1.00	0.00	H
ATOM	4182	C	ALA	268	50.944	39.123	22.992	1.00	0.00	C
ATOM	4183	O	ALA	268	50.177	39.187	22.034	1.00	0.00	O
ATOM	4184	N	ILE	269	51.092	40.134	23.855	1.00	0.00	N
ATOM	4185	H	ILE	269	51.728	40.004	24.638	1.00	0.00	H
ATOM	4186	CA	ILE	269	50.405	41.426	23.735	1.00	0.00	C
ATOM	4187	HA	ILE	269	49.332	41.239	23.677	1.00	0.00	H
ATOM	4188	CB	ILE	269	50.659	42.283	25.000	1.00	0.00	C
ATOM	4189	HB	ILE	269	51.723	42.241	25.236	1.00	0.00	H
ATOM	4190	CG2	ILE	269	50.283	43.762	24.797	1.00	0.00	C
ATOM	4191	HG21	ILE	269	50.939	44.210	24.051	1.00	0.00	H
ATOM	4192	HG22	ILE	269	50.413	44.322	25.721	1.00	0.00	H
ATOM	4193	HG23	ILE	269	49.248	43.847	24.465	1.00	0.00	H
ATOM	4194	CG1	ILE	269	49.856	41.690	26.184	1.00	0.00	C
ATOM	4195	HG12	ILE	269	48.794	41.900	26.047	1.00	0.00	H
ATOM	4196	HG13	ILE	269	49.973	40.606	26.199	1.00	0.00	H
ATOM	4197	CD1	ILE	269	50.295	42.207	27.560	1.00	0.00	C
ATOM	4198	HD11	ILE	269	51.371	42.084	27.681	1.00	0.00	H
ATOM	4199	HD12	ILE	269	49.786	41.633	28.335	1.00	0.00	H
ATOM	4200	HD13	ILE	269	50.031	43.256	27.675	1.00	0.00	H
ATOM	4201	C	ILE	269	50.787	42.123	22.420	1.00	0.00	C
ATOM	4202	O	ILE	269	49.896	42.593	21.722	1.00	0.00	O
ATOM	4203	N	LEU	270	52.061	42.115	22.010	1.00	0.00	N
ATOM	4204	H	LEU	270	52.767	41.743	22.638	1.00	0.00	H
ATOM	4205	CA	LEU	270	52.501	42.675	20.725	1.00	0.00	C
ATOM	4206	HA	LEU	270	52.205	43.724	20.703	1.00	0.00	H
ATOM	4207	CB	LEU	270	54.039	42.613	20.651	1.00	0.00	C
ATOM	4208	HB2	LEU	270	54.451	43.188	21.482	1.00	0.00	H
ATOM	4209	HB3	LEU	270	54.353	41.579	20.778	1.00	0.00	H

ATOM	4210	CG	LEU	270	54.650	43.133	19.335	1.00	0.00	C
ATOM	4211	HG	LEU	270	54.266	42.549	18.498	1.00	0.00	H
ATOM	4212	CD1	LEU	270	54.339	44.611	19.093	1.00	0.00	C
ATOM	4213	HD11	LEU	270	53.267	44.750	18.952	1.00	0.00	H
ATOM	4214	HD12	LEU	270	54.849	44.951	18.191	1.00	0.00	H
ATOM	4215	HD13	LEU	270	54.677	45.209	19.939	1.00	0.00	H
ATOM	4216	CD2	LEU	270	56.169	42.961	19.375	1.00	0.00	C
ATOM	4217	HD21	LEU	270	56.591	43.540	20.197	1.00	0.00	H
ATOM	4218	HD22	LEU	270	56.417	41.908	19.514	1.00	0.00	H
ATOM	4219	HD23	LEU	270	56.605	43.302	18.436	1.00	0.00	H
ATOM	4220	C	LEU	270	51.815	41.998	19.521	1.00	0.00	C
ATOM	4221	O	LEU	270	51.252	42.695	18.673	1.00	0.00	O
ATOM	4222	N	ARG	271	51.775	40.656	19.463	1.00	0.00	N
ATOM	4223	H	ARG	271	52.271	40.127	20.179	1.00	0.00	H
ATOM	4224	CA	ARG	271	51.002	39.944	18.423	1.00	0.00	C
ATOM	4225	HA	ARG	271	51.319	40.318	17.447	1.00	0.00	H
ATOM	4226	CB	ARG	271	51.267	38.424	18.458	1.00	0.00	C
ATOM	4227	HB2	ARG	271	52.323	38.250	18.246	1.00	0.00	H
ATOM	4228	HB3	ARG	271	51.043	38.032	19.451	1.00	0.00	H
ATOM	4229	CG	ARG	271	50.410	37.680	17.411	1.00	0.00	C
ATOM	4230	HG2	ARG	271	49.366	37.704	17.729	1.00	0.00	H
ATOM	4231	HG3	ARG	271	50.491	38.189	16.449	1.00	0.00	H
ATOM	4232	CD	ARG	271	50.817	36.214	17.220	1.00	0.00	C
ATOM	4233	HD2	ARG	271	51.826	36.187	16.812	1.00	0.00	H
ATOM	4234	HD3	ARG	271	50.821	35.718	18.193	1.00	0.00	H
ATOM	4235	NE	ARG	271	49.877	35.507	16.322	1.00	0.00	N
ATOM	4236	HE	ARG	271	48.954	35.919	16.204	1.00	0.00	H
ATOM	4237	CZ	ARG	271	50.094	34.395	15.638	1.00	0.00	C
ATOM	4238	NH1	ARG	271	51.289	33.897	15.490	1.00	0.00	N
ATOM	4239	HH11	ARG	271	52.095	34.430	15.781	1.00	0.00	H
ATOM	4240	HH12	ARG	271	51.425	33.035	14.980	1.00	0.00	H
ATOM	4241	NH2	ARG	271	49.112	33.750	15.078	1.00	0.00	N
ATOM	4242	HH21	ARG	271	48.153	33.960	15.364	1.00	0.00	H
ATOM	4243	HH22	ARG	271	49.288	32.868	14.609	1.00	0.00	H
ATOM	4244	C	ARG	271	49.510	40.264	18.524	1.00	0.00	C
ATOM	4245	O	ARG	271	48.872	40.472	17.500	1.00	0.00	O
ATOM	4246	N	ARG	272	48.949	40.337	19.733	1.00	0.00	N
ATOM	4247	H	ARG	272	49.543	40.140	20.535	1.00	0.00	H
ATOM	4248	CA	ARG	272	47.526	40.624	19.973	1.00	0.00	C
ATOM	4249	HA	ARG	272	46.944	40.083	19.229	1.00	0.00	H
ATOM	4250	CB	ARG	272	47.127	40.094	21.368	1.00	0.00	C
ATOM	4251	HB2	ARG	272	47.810	40.505	22.106	1.00	0.00	H
ATOM	4252	HB3	ARG	272	46.124	40.437	21.629	1.00	0.00	H
ATOM	4253	CG	ARG	272	47.160	38.550	21.450	1.00	0.00	C
ATOM	4254	HG2	ARG	272	48.064	38.176	20.966	1.00	0.00	H
ATOM	4255	HG3	ARG	272	47.208	38.255	22.498	1.00	0.00	H
ATOM	4256	CD	ARG	272	45.946	37.867	20.811	1.00	0.00	C
ATOM	4257	HD2	ARG	272	45.067	38.051	21.436	1.00	0.00	H
ATOM	4258	HD3	ARG	272	45.750	38.320	19.839	1.00	0.00	H
ATOM	4259	NE	ARG	272	46.151	36.405	20.673	1.00	0.00	N
ATOM	4260	HE	ARG	272	46.252	35.863	21.517	1.00	0.00	H
ATOM	4261	CZ	ARG	272	46.204	35.705	19.549	1.00	0.00	C

ATOM 4262 NH1 ARG 272 46.079 36.249 18.374 1.00 0.00 N
ATOM 4263 HH11 ARG 272 45.899 37.226 18.281 1.00 0.00 H
ATOM 4264 HH12 ARG 272 46.120 35.641 17.559 1.00 0.00 H
ATOM 4265 NH2 ARG 272 46.390 34.419 19.567 1.00 0.00 N
ATOM 4266 HH21 ARG 272 46.524 33.915 20.445 1.00 0.00 H
ATOM 4267 HH22 ARG 272 46.365 33.916 18.686 1.00 0.00 H
ATOM 4268 C ARG 272 47.142 42.094 19.743 1.00 0.00 C
ATOM 4269 O ARG 272 45.954 42.340 19.586 1.00 0.00 O
ATOM 4270 N ILE 273 48.092 43.027 19.635 1.00 0.00 N
ATOM 4271 H ILE 273 49.020 42.772 19.953 1.00 0.00 H
ATOM 4272 CA ILE 273 47.906 44.387 19.084 1.00 0.00 C
ATOM 4273 HA ILE 273 46.928 44.769 19.387 1.00 0.00 H
ATOM 4274 CB ILE 273 48.996 45.331 19.651 1.00 0.00 C
ATOM 4275 HB ILE 273 49.962 44.841 19.528 1.00 0.00 H
ATOM 4276 CG2 ILE 273 49.060 46.681 18.909 1.00 0.00 C
ATOM 4277 HG21 ILE 273 49.384 46.533 17.879 1.00 0.00 H
ATOM 4278 HG22 ILE 273 49.777 47.347 19.388 1.00 0.00 H
ATOM 4279 HG23 ILE 273 48.079 47.154 18.910 1.00 0.00 H
ATOM 4280 CG1 ILE 273 48.763 45.593 21.157 1.00 0.00 C
ATOM 4281 HG12 ILE 273 48.007 46.366 21.281 1.00 0.00 H
ATOM 4282 HG13 ILE 273 48.387 44.693 21.642 1.00 0.00 H
ATOM 4283 CD1 ILE 273 50.044 46.009 21.890 1.00 0.00 C
ATOM 4284 HD11 ILE 273 50.822 45.263 21.741 1.00 0.00 H
ATOM 4285 HD12 ILE 273 49.835 46.089 22.956 1.00 0.00 H
ATOM 4286 HD13 ILE 273 50.396 46.975 21.531 1.00 0.00 H
ATOM 4287 C ILE 273 47.920 44.361 17.543 1.00 0.00 C
ATOM 4288 O ILE 273 47.177 45.098 16.903 1.00 0.00 O
ATOM 4289 N GLN 274 48.713 43.470 16.937 1.00 0.00 N
ATOM 4290 H GLN 274 49.328 42.919 17.523 1.00 0.00 H
ATOM 4291 CA GLN 274 48.678 43.131 15.501 1.00 0.00 C
ATOM 4292 HA GLN 274 48.528 44.058 14.947 1.00 0.00 H
ATOM 4293 CB GLN 274 50.061 42.549 15.126 1.00 0.00 C
ATOM 4294 HB2 GLN 274 50.834 43.128 15.634 1.00 0.00 H
ATOM 4295 HB3 GLN 274 50.129 41.531 15.508 1.00 0.00 H
ATOM 4296 CG GLN 274 50.412 42.512 13.625 1.00 0.00 C
ATOM 4297 HG2 GLN 274 51.411 42.089 13.532 1.00 0.00 H
ATOM 4298 HG3 GLN 274 49.731 41.855 13.089 1.00 0.00 H
ATOM 4299 CD GLN 274 50.433 43.876 12.940 1.00 0.00 C
ATOM 4300 OE1 GLN 274 49.962 44.048 11.827 1.00 0.00 O
ATOM 4301 NE2 GLN 274 50.978 44.903 13.556 1.00 0.00 N
ATOM 4302 HE21 GLN 274 50.999 45.767 13.047 1.00 0.00 H
ATOM 4303 HE22 GLN 274 51.391 44.790 14.464 1.00 0.00 H
ATOM 4304 C GLN 274 47.497 42.190 15.129 1.00 0.00 C
ATOM 4305 O GLN 274 47.473 41.599 14.048 1.00 0.00 O
ATOM 4306 N GLN 275 46.532 42.004 16.036 1.00 0.00 N
ATOM 4307 H GLN 275 46.563 42.601 16.851 1.00 0.00 H
ATOM 4308 CA GLN 275 45.361 41.120 15.949 1.00 0.00 C
ATOM 4309 HA GLN 275 44.930 41.187 14.951 1.00 0.00 H
ATOM 4310 CB GLN 275 45.794 39.651 16.225 1.00 0.00 C
ATOM 4311 HB2 GLN 275 46.658 39.633 16.883 1.00 0.00 H
ATOM 4312 HB3 GLN 275 45.021 39.098 16.754 1.00 0.00 H
ATOM 4313 CG GLN 275 46.082 38.853 14.944 1.00 0.00 C

ATOM	4314	HG2	GLN	275	45.376	38.025	14.879	1.00	0.00	H
ATOM	4315	HG3	GLN	275	45.925	39.469	14.059	1.00	0.00	H
ATOM	4316	CD	GLN	275	47.489	38.271	14.904	1.00	0.00	C
ATOM	4317	OE1	GLN	275	47.782	37.247	15.506	1.00	0.00	O
ATOM	4318	NE2	GLN	275	48.384	38.884	14.167	1.00	0.00	N
ATOM	4319	HE21	GLN	275	49.283	38.441	14.031	1.00	0.00	H
ATOM	4320	HE22	GLN	275	48.161	39.801	13.792	1.00	0.00	H
ATOM	4321	C	GLN	275	44.280	41.632	16.926	1.00	0.00	C
ATOM	4322	O	GLN	275	44.281	42.804	17.294	1.00	0.00	O
ATOM	4323	N	SER	276	43.342	40.775	17.340	1.00	0.00	N
ATOM	4324	H	SER	276	43.378	39.839	16.953	1.00	0.00	H
ATOM	4325	CA	SER	276	42.277	40.993	18.339	1.00	0.00	C
ATOM	4326	HA	SER	276	41.756	40.040	18.429	1.00	0.00	H
ATOM	4327	CB	SER	276	42.848	41.294	19.732	1.00	0.00	C
ATOM	4328	HB2	SER	276	43.076	42.359	19.806	1.00	0.00	H
ATOM	4329	HB3	SER	276	42.099	41.040	20.483	1.00	0.00	H
ATOM	4330	OG	SER	276	44.026	40.568	20.002	1.00	0.00	O
ATOM	4331	HG	SER	276	44.751	41.201	19.819	1.00	0.00	H
ATOM	4332	C	SER	276	41.198	42.023	17.969	1.00	0.00	C
ATOM	4333	O	SER	276	40.052	41.857	18.394	1.00	0.00	O
ATOM	4334	N	GLY	277	41.529	43.036	17.167	1.00	0.00	N
ATOM	4335	H	GLY	277	42.514	43.113	16.926	1.00	0.00	H
ATOM	4336	CA	GLY	277	40.639	44.022	16.551	1.00	0.00	C
ATOM	4337	HA2	GLY	277	39.692	43.553	16.281	1.00	0.00	H
ATOM	4338	HA3	GLY	277	40.435	44.818	17.266	1.00	0.00	H
ATOM	4339	C	GLY	277	41.267	44.634	15.286	1.00	0.00	C
ATOM	4340	O	GLY	277	42.271	44.137	14.768	1.00	0.00	O
ATOM	4341	N	SER	278	40.670	45.699	14.752	1.00	0.00	N
ATOM	4342	H	SER	278	39.790	45.995	15.154	1.00	0.00	H
ATOM	4343	CA	SER	278	41.227	46.563	13.688	1.00	0.00	C
ATOM	4344	HA	SER	278	42.276	46.777	13.901	1.00	0.00	H
ATOM	4345	CB	SER	278	41.127	45.908	12.304	1.00	0.00	C
ATOM	4346	HB2	SER	278	40.160	45.414	12.194	1.00	0.00	H
ATOM	4347	HB3	SER	278	41.228	46.667	11.526	1.00	0.00	H
ATOM	4348	OG	SER	278	42.176	44.969	12.159	1.00	0.00	O
ATOM	4349	HG	SER	278	42.300	44.556	13.031	1.00	0.00	H
ATOM	4350	C	SER	278	40.458	47.882	13.688	1.00	0.00	C
ATOM	4351	O	SER	278	39.227	47.848	13.746	1.00	0.00	O
ATOM	4352	N	ALA	279	41.174	49.012	13.725	1.00	0.00	N
ATOM	4353	H	ALA	279	42.157	48.926	13.488	1.00	0.00	H
ATOM	4354	CA	ALA	279	40.727	50.345	14.186	1.00	0.00	C
ATOM	4355	HA	ALA	279	41.613	50.981	14.195	1.00	0.00	H
ATOM	4356	CB	ALA	279	39.766	50.951	13.153	1.00	0.00	C
ATOM	4357	HB1	ALA	279	38.837	50.382	13.123	1.00	0.00	H
ATOM	4358	HB2	ALA	279	40.227	50.943	12.164	1.00	0.00	H
ATOM	4359	HB3	ALA	279	39.535	51.980	13.429	1.00	0.00	H
ATOM	4360	C	ALA	279	40.164	50.411	15.634	1.00	0.00	C
ATOM	4361	O	ALA	279	40.266	51.450	16.289	1.00	0.00	O
ATOM	4362	N	ASP	280	39.611	49.307	16.137	1.00	0.00	N
ATOM	4363	H	ASP	280	39.412	48.584	15.461	1.00	0.00	H
ATOM	4364	CA	ASP	280	39.051	49.117	17.476	1.00	0.00	C
ATOM	4365	HA	ASP	280	38.244	49.840	17.602	1.00	0.00	H

ATOM	4366	CB	ASP	280	38.450	47.702	17.572	1.00	0.00	C
ATOM	4367	HB2	ASP	280	39.255	46.977	17.440	1.00	0.00	H
ATOM	4368	HB3	ASP	280	38.042	47.562	18.574	1.00	0.00	H
ATOM	4369	CG	ASP	280	37.333	47.378	16.574	1.00	0.00	C
ATOM	4370	OD1	ASP	280	36.690	48.301	16.034	1.00	0.00	O
ATOM	4371	OD2	ASP	280	37.057	46.164	16.401	1.00	0.00	O
ATOM	4372	C	ASP	280	40.047	49.308	18.639	1.00	0.00	C
ATOM	4373	O	ASP	280	41.262	49.156	18.509	1.00	0.00	O
ATOM	4374	N	LEU	281	39.486	49.529	19.833	1.00	0.00	N
ATOM	4375	H	LEU	281	38.481	49.602	19.838	1.00	0.00	H
ATOM	4376	CA	LEU	281	40.163	49.872	21.093	1.00	0.00	C
ATOM	4377	HA	LEU	281	40.639	50.845	20.959	1.00	0.00	H
ATOM	4378	CB	LEU	281	39.043	49.989	22.150	1.00	0.00	C
ATOM	4379	HB2	LEU	281	38.190	50.510	21.710	1.00	0.00	H
ATOM	4380	HB3	LEU	281	38.714	48.979	22.400	1.00	0.00	H
ATOM	4381	CG	LEU	281	39.413	50.724	23.452	1.00	0.00	C
ATOM	4382	HG	LEU	281	40.327	50.319	23.874	1.00	0.00	H
ATOM	4383	CD1	LEU	281	39.609	52.220	23.202	1.00	0.00	C
ATOM	4384	HD11	LEU	281	40.513	52.381	22.615	1.00	0.00	H
ATOM	4385	HD12	LEU	281	39.722	52.747	24.148	1.00	0.00	H
ATOM	4386	HD13	LEU	281	38.758	52.637	22.665	1.00	0.00	H
ATOM	4387	CD2	LEU	281	38.292	50.506	24.470	1.00	0.00	C
ATOM	4388	HD21	LEU	281	37.338	50.829	24.055	1.00	0.00	H
ATOM	4389	HD22	LEU	281	38.224	49.447	24.722	1.00	0.00	H
ATOM	4390	HD23	LEU	281	38.505	51.065	25.381	1.00	0.00	H
ATOM	4391	C	LEU	281	41.258	48.888	21.566	1.00	0.00	C
ATOM	4392	O	LEU	281	42.070	49.232	22.420	1.00	0.00	O
ATOM	4393	N	ARG	282	41.310	47.665	21.024	1.00	0.00	N
ATOM	4394	H	ARG	282	40.683	47.494	20.250	1.00	0.00	H
ATOM	4395	CA	ARG	282	42.277	46.608	21.391	1.00	0.00	C
ATOM	4396	HA	ARG	282	42.326	46.564	22.480	1.00	0.00	H
ATOM	4397	CB	ARG	282	41.758	45.244	20.885	1.00	0.00	C
ATOM	4398	HB2	ARG	282	41.428	45.341	19.848	1.00	0.00	H
ATOM	4399	HB3	ARG	282	42.569	44.515	20.913	1.00	0.00	H
ATOM	4400	CG	ARG	282	40.607	44.700	21.757	1.00	0.00	C
ATOM	4401	HG2	ARG	282	40.995	44.496	22.757	1.00	0.00	H
ATOM	4402	HG3	ARG	282	39.807	45.438	21.832	1.00	0.00	H
ATOM	4403	CD	ARG	282	40.038	43.406	21.160	1.00	0.00	C
ATOM	4404	HD2	ARG	282	39.435	43.660	20.285	1.00	0.00	H
ATOM	4405	HD3	ARG	282	40.870	42.786	20.835	1.00	0.00	H
ATOM	4406	NE	ARG	282	39.229	42.626	22.119	1.00	0.00	N
ATOM	4407	HE	ARG	282	39.137	42.967	23.066	1.00	0.00	H
ATOM	4408	CZ	ARG	282	38.641	41.464	21.878	1.00	0.00	C
ATOM	4409	NH1	ARG	282	38.676	40.898	20.708	1.00	0.00	N
ATOM	4410	HH11	ARG	282	39.142	41.373	19.947	1.00	0.00	H
ATOM	4411	HH12	ARG	282	38.296	39.968	20.566	1.00	0.00	H
ATOM	4412	NH2	ARG	282	37.999	40.869	22.837	1.00	0.00	N
ATOM	4413	HH21	ARG	282	38.020	41.283	23.759	1.00	0.00	H
ATOM	4414	HH22	ARG	282	37.501	39.997	22.686	1.00	0.00	H
ATOM	4415	C	ARG	282	43.738	46.896	20.977	1.00	0.00	C
ATOM	4416	O	ARG	282	44.604	46.058	21.217	1.00	0.00	O
ATOM	4417	N	LYS	283	44.039	48.088	20.440	1.00	0.00	N

ATOM	4418	H	LYS	283	43.253	48.700	20.268	1.00	0.00	H
ATOM	4419	CA	LYS	283	45.389	48.660	20.218	1.00	0.00	C
ATOM	4420	HA	LYS	283	45.997	47.891	19.740	1.00	0.00	H
ATOM	4421	CB	LYS	283	45.243	49.845	19.238	1.00	0.00	C
ATOM	4422	HB2	LYS	283	44.477	49.601	18.499	1.00	0.00	H
ATOM	4423	HB3	LYS	283	44.911	50.734	19.779	1.00	0.00	H
ATOM	4424	CG	LYS	283	46.540	50.147	18.474	1.00	0.00	C
ATOM	4425	HG2	LYS	283	47.350	50.342	19.179	1.00	0.00	H
ATOM	4426	HG3	LYS	283	46.802	49.277	17.870	1.00	0.00	H
ATOM	4427	CD	LYS	283	46.365	51.368	17.557	1.00	0.00	C
ATOM	4428	HD2	LYS	283	45.467	51.249	16.947	1.00	0.00	H
ATOM	4429	HD3	LYS	283	46.253	52.257	18.176	1.00	0.00	H
ATOM	4430	CE	LYS	283	47.581	51.521	16.638	1.00	0.00	C
ATOM	4431	HE2	LYS	283	48.489	51.411	17.238	1.00	0.00	H
ATOM	4432	HE3	LYS	283	47.561	50.716	15.898	1.00	0.00	H
ATOM	4433	NZ	LYS	283	47.608	52.837	15.957	1.00	0.00	N
ATOM	4434	HZ1	LYS	283	47.825	53.574	16.623	1.00	0.00	H
ATOM	4435	HZ2	LYS	283	46.721	53.093	15.525	1.00	0.00	H
ATOM	4436	HZ3	LYS	283	48.346	52.866	15.258	1.00	0.00	H
ATOM	4437	C	LYS	283	46.095	49.029	21.551	1.00	0.00	C
ATOM	4438	O	LYS	283	46.441	50.181	21.817	1.00	0.00	O
ATOM	4439	N	LEU	284	46.208	48.029	22.424	1.00	0.00	N
ATOM	4440	H	LEU	284	45.922	47.123	22.076	1.00	0.00	H
ATOM	4441	CA	LEU	284	46.495	48.103	23.861	1.00	0.00	C
ATOM	4442	HA	LEU	284	45.817	48.840	24.289	1.00	0.00	H
ATOM	4443	CB	LEU	284	46.151	46.704	24.430	1.00	0.00	C
ATOM	4444	HB2	LEU	284	45.174	46.410	24.042	1.00	0.00	H
ATOM	4445	HB3	LEU	284	46.881	45.993	24.039	1.00	0.00	H
ATOM	4446	CG	LEU	284	46.102	46.548	25.963	1.00	0.00	C
ATOM	4447	HG	LEU	284	47.078	46.755	26.397	1.00	0.00	H
ATOM	4448	CD1	LEU	284	45.060	47.480	26.579	1.00	0.00	C
ATOM	4449	HD11	LEU	284	45.364	48.520	26.478	1.00	0.00	H
ATOM	4450	HD12	LEU	284	44.950	47.257	27.639	1.00	0.00	H
ATOM	4451	HD13	LEU	284	44.101	47.332	26.086	1.00	0.00	H
ATOM	4452	CD2	LEU	284	45.725	45.113	26.329	1.00	0.00	C
ATOM	4453	HD21	LEU	284	44.728	44.875	25.956	1.00	0.00	H
ATOM	4454	HD22	LEU	284	46.448	44.421	25.898	1.00	0.00	H
ATOM	4455	HD23	LEU	284	45.739	44.997	27.414	1.00	0.00	H
ATOM	4456	C	LEU	284	47.935	48.538	24.208	1.00	0.00	C
ATOM	4457	O	LEU	284	48.837	48.483	23.381	1.00	0.00	O
ATOM	4458	N	ASP	285	48.159	48.913	25.470	1.00	0.00	N
ATOM	4459	H	ASP	285	47.371	48.949	26.100	1.00	0.00	H
ATOM	4460	CA	ASP	285	49.474	49.101	26.092	1.00	0.00	C
ATOM	4461	HA	ASP	285	50.220	48.521	25.545	1.00	0.00	H
ATOM	4462	CB	ASP	285	49.877	50.582	26.046	1.00	0.00	C
ATOM	4463	HB2	ASP	285	49.003	51.197	26.266	1.00	0.00	H
ATOM	4464	HB3	ASP	285	50.620	50.767	26.824	1.00	0.00	H
ATOM	4465	CG	ASP	285	50.492	51.014	24.716	1.00	0.00	C
ATOM	4466	OD1	ASP	285	51.588	50.498	24.388	1.00	0.00	O
ATOM	4467	OD2	ASP	285	49.962	51.965	24.087	1.00	0.00	O
ATOM	4468	C	ASP	285	49.445	48.594	27.546	1.00	0.00	C
ATOM	4469	O	ASP	285	48.472	48.820	28.266	1.00	0.00	O

ATOM 4470 N CYS 286 50.518 47.935 27.988 1.00 0.00 N
ATOM 4471 H CYS 286 51.316 47.853 27.376 1.00 0.00 H
ATOM 4472 CA CYS 286 50.641 47.306 29.309 1.00 0.00 C
ATOM 4473 HA CYS 286 49.805 47.607 29.944 1.00 0.00 H
ATOM 4474 CB CYS 286 50.572 45.782 29.120 1.00 0.00 C
ATOM 4475 HB2 CYS 286 49.652 45.533 28.588 1.00 0.00 H
ATOM 4476 HB3 CYS 286 51.423 45.447 28.525 1.00 0.00 H
ATOM 4477 SG CYS 286 50.568 44.899 30.709 1.00 0.00 S
ATOM 4478 HG CYS 286 51.722 45.353 31.215 1.00 0.00 H
ATOM 4479 C CYS 286 51.938 47.743 30.008 1.00 0.00 C
ATOM 4480 O CYS 286 52.919 48.083 29.340 1.00 0.00 O
ATOM 4481 N ARG 287 51.942 47.711 31.343 1.00 0.00 N
ATOM 4482 H ARG 287 51.066 47.461 31.805 1.00 0.00 H
ATOM 4483 CA ARG 287 53.078 47.996 32.230 1.00 0.00 C
ATOM 4484 HA ARG 287 53.995 48.011 31.647 1.00 0.00 H
ATOM 4485 CB ARG 287 52.856 49.368 32.890 1.00 0.00 C
ATOM 4486 HB2 ARG 287 51.919 49.338 33.450 1.00 0.00 H
ATOM 4487 HB3 ARG 287 53.658 49.529 33.605 1.00 0.00 H
ATOM 4488 CG ARG 287 52.800 50.564 31.909 1.00 0.00 C
ATOM 4489 HG2 ARG 287 51.950 50.447 31.237 1.00 0.00 H
ATOM 4490 HG3 ARG 287 52.652 51.478 32.485 1.00 0.00 H
ATOM 4491 CD ARG 287 54.089 50.701 31.087 1.00 0.00 C
ATOM 4492 HD2 ARG 287 54.918 50.780 31.786 1.00 0.00 H
ATOM 4493 HD3 ARG 287 54.237 49.794 30.504 1.00 0.00 H
ATOM 4494 NE ARG 287 54.083 51.845 30.147 1.00 0.00 N
ATOM 4495 HE ARG 287 53.270 51.966 29.569 1.00 0.00 H
ATOM 4496 CZ ARG 287 55.119 52.620 29.862 1.00 0.00 C
ATOM 4497 NH1 ARG 287 56.229 52.579 30.527 1.00 0.00 N
ATOM 4498 HH11 ARG 287 56.386 51.927 31.275 1.00 0.00 H
ATOM 4499 HH12 ARG 287 57.041 53.073 30.181 1.00 0.00 H
ATOM 4500 NH2 ARG 287 55.111 53.452 28.865 1.00 0.00 N
ATOM 4501 HH21 ARG 287 54.377 53.462 28.159 1.00 0.00 H
ATOM 4502 HH22 ARG 287 55.941 53.977 28.662 1.00 0.00 H
ATOM 4503 C ARG 287 53.247 46.872 33.263 1.00 0.00 C
ATOM 4504 O ARG 287 52.285 46.164 33.553 1.00 0.00 O
ATOM 4505 N PHE 288 54.469 46.654 33.755 1.00 0.00 N
ATOM 4506 H PHE 288 55.224 47.232 33.411 1.00 0.00 H
ATOM 4507 CA PHE 288 54.853 45.408 34.435 1.00 0.00 C
ATOM 4508 HA PHE 288 53.984 44.984 34.940 1.00 0.00 H
ATOM 4509 CB PHE 288 55.325 44.429 33.342 1.00 0.00 C
ATOM 4510 HB2 PHE 288 54.831 44.674 32.400 1.00 0.00 H
ATOM 4511 HB3 PHE 288 56.389 44.579 33.174 1.00 0.00 H
ATOM 4512 CG PHE 288 55.083 42.957 33.599 1.00 0.00 C
ATOM 4513 CD1 PHE 288 54.020 42.303 32.945 1.00 0.00 C
ATOM 4514 HD1 PHE 288 53.350 42.860 32.305 1.00 0.00 H
ATOM 4515 CE1 PHE 288 53.824 40.922 33.114 1.00 0.00 C
ATOM 4516 HE1 PHE 288 53.003 40.425 32.617 1.00 0.00 H
ATOM 4517 CZ PHE 288 54.699 40.188 33.931 1.00 0.00 C
ATOM 4518 HZ PHE 288 54.553 39.124 34.057 1.00 0.00 H
ATOM 4519 CE2 PHE 288 55.759 40.839 34.586 1.00 0.00 C
ATOM 4520 HE2 PHE 288 56.426 40.269 35.215 1.00 0.00 H
ATOM 4521 CD2 PHE 288 55.953 42.221 34.424 1.00 0.00 C

ATOM 4522 HD2 PHE 288 56.777 42.711 34.923 1.00 0.00 H
ATOM 4523 C PHE 288 55.961 45.639 35.483 1.00 0.00 C
ATOM 4524 O PHE 288 56.941 46.331 35.196 1.00 0.00 O
ATOM 4525 N GLY 289 55.834 45.042 36.672 1.00 0.00 N
ATOM 4526 H GLY 289 54.985 44.511 36.855 1.00 0.00 H
ATOM 4527 CA GLY 289 56.782 45.153 37.788 1.00 0.00 C
ATOM 4528 HA2 GLY 289 57.724 45.549 37.428 1.00 0.00 H
ATOM 4529 HA3 GLY 289 56.397 45.857 38.523 1.00 0.00 H
ATOM 4530 C GLY 289 57.023 43.822 38.510 1.00 0.00 C
ATOM 4531 O GLY 289 56.078 43.056 38.711 1.00 0.00 O
ATOM 4532 N ILE 290 58.273 43.537 38.908 1.00 0.00 N
ATOM 4533 H ILE 290 59.008 44.191 38.663 1.00 0.00 H
ATOM 4534 CA ILE 290 58.652 42.275 39.583 1.00 0.00 C
ATOM 4535 HA ILE 290 57.739 41.838 39.985 1.00 0.00 H
ATOM 4536 CB ILE 290 59.260 41.229 38.606 1.00 0.00 C
ATOM 4537 HB ILE 290 60.301 41.505 38.433 1.00 0.00 H
ATOM 4538 CG2 ILE 290 59.252 39.830 39.257 1.00 0.00 C
ATOM 4539 HG21 ILE 290 59.737 39.842 40.231 1.00 0.00 H
ATOM 4540 HG22 ILE 290 59.792 39.114 38.638 1.00 0.00 H
ATOM 4541 HG23 ILE 290 58.225 39.482 39.380 1.00 0.00 H
ATOM 4542 CG1 ILE 290 58.563 41.176 37.226 1.00 0.00 C
ATOM 4543 HG12 ILE 290 57.512 40.915 37.358 1.00 0.00 H
ATOM 4544 HG13 ILE 290 58.614 42.160 36.761 1.00 0.00 H
ATOM 4545 CD1 ILE 290 59.204 40.192 36.237 1.00 0.00 C
ATOM 4546 HD11 ILE 290 60.288 40.302 36.248 1.00 0.00 H
ATOM 4547 HD12 ILE 290 58.844 40.409 35.234 1.00 0.00 H
ATOM 4548 HD13 ILE 290 58.938 39.166 36.492 1.00 0.00 H
ATOM 4549 C ILE 290 59.606 42.501 40.771 1.00 0.00 C
ATOM 4550 O ILE 290 60.665 43.121 40.622 1.00 0.00 O
ATOM 4551 N ASN 291 59.286 41.894 41.919 1.00 0.00 N
ATOM 4552 H ASN 291 58.433 41.338 41.919 1.00 0.00 H
ATOM 4553 CA ASN 291 60.156 41.763 43.096 1.00 0.00 C
ATOM 4554 HA ASN 291 61.156 42.122 42.848 1.00 0.00 H
ATOM 4555 CB ASN 291 59.615 42.619 44.257 1.00 0.00 C
ATOM 4556 HB2 ASN 291 58.530 42.618 44.252 1.00 0.00 H
ATOM 4557 HB3 ASN 291 59.929 42.202 45.213 1.00 0.00 H
ATOM 4558 CG ASN 291 60.108 44.044 44.179 1.00 0.00 C
ATOM 4559 OD1 ASN 291 59.619 44.865 43.429 1.00 0.00 O
ATOM 4560 ND2 ASN 291 61.111 44.388 44.955 1.00 0.00 N
ATOM 4561 HD21 ASN 291 61.561 43.725 45.557 1.00 0.00 H
ATOM 4562 HD22 ASN 291 61.436 45.332 44.860 1.00 0.00 H
ATOM 4563 C ASN 291 60.311 40.295 43.526 1.00 0.00 C
ATOM 4564 O ASN 291 59.527 39.434 43.141 1.00 0.00 O
ATOM 4565 N ALA 292 61.292 40.016 44.386 1.00 0.00 N
ATOM 4566 H ALA 292 61.922 40.750 44.668 1.00 0.00 H
ATOM 4567 CA ALA 292 61.432 38.732 45.069 1.00 0.00 C
ATOM 4568 HA ALA 292 60.464 38.232 45.068 1.00 0.00 H
ATOM 4569 CB ALA 292 62.423 37.845 44.303 1.00 0.00 C
ATOM 4570 HB1 ALA 292 63.407 38.312 44.278 1.00 0.00 H
ATOM 4571 HB2 ALA 292 62.071 37.698 43.281 1.00 0.00 H
ATOM 4572 HB3 ALA 292 62.496 36.871 44.789 1.00 0.00 H
ATOM 4573 C ALA 292 61.828 38.946 46.538 1.00 0.00 C

ATOM	4574	O	ALA	292	62.544	39.901	46.858	1.00	0.00	O
ATOM	4575	N	GLY	293	61.343	38.083	47.429	1.00	0.00	N
ATOM	4576	H	GLY	293	60.737	37.334	47.100	1.00	0.00	H
ATOM	4577	CA	GLY	293	61.498	38.229	48.878	1.00	0.00	C
ATOM	4578	HA2	GLY	293	62.527	38.002	49.157	1.00	0.00	H
ATOM	4579	HA3	GLY	293	61.282	39.258	49.166	1.00	0.00	H
ATOM	4580	C	GLY	293	60.566	37.304	49.661	1.00	0.00	C
ATOM	4581	O	GLY	293	59.837	36.507	49.077	1.00	0.00	O
ATOM	4582	N	ASP	294	60.608	37.384	50.990	1.00	0.00	N
ATOM	4583	H	ASP	294	61.195	38.078	51.424	1.00	0.00	H
ATOM	4584	CA	ASP	294	59.771	36.555	51.867	1.00	0.00	C
ATOM	4585	HA	ASP	294	59.830	35.530	51.506	1.00	0.00	H
ATOM	4586	CB	ASP	294	60.347	36.575	53.296	1.00	0.00	C
ATOM	4587	HB2	ASP	294	61.431	36.696	53.240	1.00	0.00	H
ATOM	4588	HB3	ASP	294	59.944	37.432	53.839	1.00	0.00	H
ATOM	4589	CG	ASP	294	60.065	35.287	54.081	1.00	0.00	C
ATOM	4590	OD1	ASP	294	59.229	34.486	53.604	1.00	0.00	O
ATOM	4591	OD2	ASP	294	60.700	35.112	55.148	1.00	0.00	O
ATOM	4592	C	ASP	294	58.287	36.970	51.805	1.00	0.00	C
ATOM	4593	O	ASP	294	57.965	38.140	51.551	1.00	0.00	O
ATOM	4594	N	CYS	295	57.367	36.026	52.005	1.00	0.00	N
ATOM	4595	H	CYS	295	57.693	35.088	52.238	1.00	0.00	H
ATOM	4596	CA	CYS	295	55.924	36.266	51.988	1.00	0.00	C
ATOM	4597	HA	CYS	295	55.765	37.228	52.453	1.00	0.00	H
ATOM	4598	CB	CYS	295	55.417	36.353	50.538	1.00	0.00	C
ATOM	4599	HB2	CYS	295	54.452	36.861	50.523	1.00	0.00	H
ATOM	4600	HB3	CYS	295	56.119	36.932	49.936	1.00	0.00	H
ATOM	4601	SG	CYS	295	55.217	34.704	49.811	1.00	0.00	S
ATOM	4602	HG	CYS	295	56.446	34.248	50.119	1.00	0.00	H
ATOM	4603	C	CYS	295	55.132	35.262	52.834	1.00	0.00	C
ATOM	4604	O	CYS	295	55.550	34.127	53.051	1.00	0.00	O
ATOM	4605	N	MET	296	53.968	35.690	53.324	1.00	0.00	N
ATOM	4606	H	MET	296	53.676	36.644	53.145	1.00	0.00	H
ATOM	4607	CA	MET	296	53.080	34.869	54.142	1.00	0.00	C
ATOM	4608	HA	MET	296	53.658	34.059	54.591	1.00	0.00	H
ATOM	4609	CB	MET	296	52.545	35.749	55.279	1.00	0.00	C
ATOM	4610	HB2	MET	296	53.367	36.369	55.633	1.00	0.00	H
ATOM	4611	HB3	MET	296	51.759	36.414	54.920	1.00	0.00	H
ATOM	4612	CG	MET	296	52.022	34.924	56.454	1.00	0.00	C
ATOM	4613	HG2	MET	296	51.067	34.479	56.174	1.00	0.00	H
ATOM	4614	HG3	MET	296	52.726	34.119	56.668	1.00	0.00	H
ATOM	4615	SD	MET	296	51.801	35.893	57.969	1.00	0.00	S
ATOM	4616	CE	MET	296	53.521	36.347	58.357	1.00	0.00	C
ATOM	4617	HE1	MET	296	53.898	37.062	57.625	1.00	0.00	H
ATOM	4618	HE2	MET	296	53.564	36.811	59.342	1.00	0.00	H
ATOM	4619	HE3	MET	296	54.150	35.458	58.355	1.00	0.00	H
ATOM	4620	C	MET	296	51.987	34.247	53.267	1.00	0.00	C
ATOM	4621	O	MET	296	50.989	34.896	52.952	1.00	0.00	O
ATOM	4622	N	VAL	297	52.209	33.010	52.823	1.00	0.00	N
ATOM	4623	H	VAL	297	53.060	32.547	53.130	1.00	0.00	H
ATOM	4624	CA	VAL	297	51.274	32.229	51.995	1.00	0.00	C
ATOM	4625	HA	VAL	297	50.782	32.896	51.291	1.00	0.00	H

ATOM 4626 CB VAL 297 52.018 31.141 51.190 1.00 0.00 C
ATOM 4627 HB VAL 297 52.409 30.395 51.881 1.00 0.00 H
ATOM 4628 CG1 VAL 297 51.078 30.432 50.205 1.00 0.00 C
ATOM 4629 HG11 VAL 297 51.635 29.697 49.624 1.00 0.00 H
ATOM 4630 HG12 VAL 297 50.289 29.907 50.744 1.00 0.00 H
ATOM 4631 HG13 VAL 297 50.629 31.159 49.528 1.00 0.00 H
ATOM 4632 CG2 VAL 297 53.207 31.697 50.394 1.00 0.00 C
ATOM 4633 HG21 VAL 297 53.677 30.901 49.817 1.00 0.00 H
ATOM 4634 HG22 VAL 297 53.957 32.109 51.070 1.00 0.00 H
ATOM 4635 HG23 VAL 297 52.872 32.480 49.715 1.00 0.00 H
ATOM 4636 C VAL 297 50.214 31.577 52.883 1.00 0.00 C
ATOM 4637 O VAL 297 50.574 30.914 53.856 1.00 0.00 O
ATOM 4638 N GLY 298 48.925 31.702 52.557 1.00 0.00 N
ATOM 4639 H GLY 298 48.664 32.281 51.762 1.00 0.00 H
ATOM 4640 CA GLY 298 47.870 31.005 53.297 1.00 0.00 C
ATOM 4641 HA2 GLY 298 47.922 29.943 53.061 1.00 0.00 H
ATOM 4642 HA3 GLY 298 48.065 31.116 54.364 1.00 0.00 H
ATOM 4643 C GLY 298 46.440 31.481 53.040 1.00 0.00 C
ATOM 4644 O GLY 298 46.154 32.227 52.101 1.00 0.00 O
ATOM 4645 N VAL 299 45.541 31.012 53.910 1.00 0.00 N
ATOM 4646 H VAL 299 45.883 30.425 54.655 1.00 0.00 H
ATOM 4647 CA VAL 299 44.141 31.446 54.013 1.00 0.00 C
ATOM 4648 HA VAL 299 43.696 31.421 53.022 1.00 0.00 H
ATOM 4649 CB VAL 299 43.350 30.485 54.929 1.00 0.00 C
ATOM 4650 HB VAL 299 43.865 30.408 55.888 1.00 0.00 H
ATOM 4651 CG1 VAL 299 41.915 30.953 55.196 1.00 0.00 C
ATOM 4652 HG11 VAL 299 41.393 31.113 54.255 1.00 0.00 H
ATOM 4653 HG12 VAL 299 41.385 30.203 55.785 1.00 0.00 H
ATOM 4654 HG13 VAL 299 41.920 31.879 55.773 1.00 0.00 H
ATOM 4655 CG2 VAL 299 43.269 29.080 54.315 1.00 0.00 C
ATOM 4656 HG21 VAL 299 44.267 28.669 54.167 1.00 0.00 H
ATOM 4657 HG22 VAL 299 42.742 29.117 53.362 1.00 0.00 H
ATOM 4658 HG23 VAL 299 42.732 28.413 54.991 1.00 0.00 H
ATOM 4659 C VAL 299 44.061 32.881 54.551 1.00 0.00 C
ATOM 4660 O VAL 299 44.784 33.225 55.484 1.00 0.00 O
ATOM 4661 N PHE 300 43.165 33.696 53.989 1.00 0.00 N
ATOM 4662 H PHE 300 42.668 33.358 53.174 1.00 0.00 H
ATOM 4663 CA PHE 300 42.875 35.054 54.462 1.00 0.00 C
ATOM 4664 HA PHE 300 42.961 35.076 55.551 1.00 0.00 H
ATOM 4665 CB PHE 300 43.927 36.016 53.876 1.00 0.00 C
ATOM 4666 HB2 PHE 300 44.857 35.899 54.434 1.00 0.00 H
ATOM 4667 HB3 PHE 300 44.129 35.715 52.847 1.00 0.00 H
ATOM 4668 CG PHE 300 43.551 37.491 53.868 1.00 0.00 C
ATOM 4669 CD1 PHE 300 43.130 38.134 55.049 1.00 0.00 C
ATOM 4670 HD1 PHE 300 43.072 37.584 55.978 1.00 0.00 H
ATOM 4671 CE1 PHE 300 42.783 39.497 55.024 1.00 0.00 C
ATOM 4672 HE1 PHE 300 42.450 39.988 55.928 1.00 0.00 H
ATOM 4673 CZ PHE 300 42.900 40.233 53.833 1.00 0.00 C
ATOM 4674 HZ PHE 300 42.683 41.294 53.825 1.00 0.00 H
ATOM 4675 CE2 PHE 300 43.309 39.595 52.652 1.00 0.00 C
ATOM 4676 HE2 PHE 300 43.405 40.170 51.743 1.00 0.00 H
ATOM 4677 CD2 PHE 300 43.618 38.224 52.666 1.00 0.00 C

ATOM	4678	HD2	PHE	300	43.941	37.742	51.756	1.00	0.00	H
ATOM	4679	C	PHE	300	41.452	35.509	54.097	1.00	0.00	C
ATOM	4680	O	PHE	300	41.101	35.590	52.913	1.00	0.00	O
ATOM	4681	N	GLY	301	40.647	35.873	55.097	1.00	0.00	N
ATOM	4682	H	GLY	301	40.913	35.621	56.052	1.00	0.00	H
ATOM	4683	CA	GLY	301	39.471	36.727	54.921	1.00	0.00	C
ATOM	4684	HA2	GLY	301	39.823	37.734	54.715	1.00	0.00	H
ATOM	4685	HA3	GLY	301	38.891	36.391	54.065	1.00	0.00	H
ATOM	4686	C	GLY	301	38.554	36.804	56.144	1.00	0.00	C
ATOM	4687	O	GLY	301	39.015	36.856	57.279	1.00	0.00	O
ATOM	4688	N	CYS	302	37.242	36.819	55.901	1.00	0.00	N
ATOM	4689	H	CYS	302	36.948	36.729	54.936	1.00	0.00	H
ATOM	4690	CA	CYS	302	36.204	36.662	56.924	1.00	0.00	C
ATOM	4691	HA	CYS	302	36.621	36.874	57.912	1.00	0.00	H
ATOM	4692	CB	CYS	302	35.084	37.677	56.632	1.00	0.00	C
ATOM	4693	HB2	CYS	302	35.475	38.671	56.837	1.00	0.00	H
ATOM	4694	HB3	CYS	302	34.807	37.612	55.579	1.00	0.00	H
ATOM	4695	SG	CYS	302	33.594	37.436	57.645	1.00	0.00	S
ATOM	4696	HG	CYS	302	33.087	38.670	57.494	1.00	0.00	H
ATOM	4697	C	CYS	302	35.684	35.217	56.926	1.00	0.00	C
ATOM	4698	O	CYS	302	35.627	34.579	55.876	1.00	0.00	O
ATOM	4699	N	SER	303	35.188	34.724	58.064	1.00	0.00	N
ATOM	4700	H	SER	303	35.293	35.260	58.914	1.00	0.00	H
ATOM	4701	CA	SER	303	34.598	33.380	58.191	1.00	0.00	C
ATOM	4702	HA	SER	303	35.363	32.651	57.918	1.00	0.00	H
ATOM	4703	CB	SER	303	34.207	33.114	59.650	1.00	0.00	C
ATOM	4704	HB2	SER	303	33.336	33.717	59.911	1.00	0.00	H
ATOM	4705	HB3	SER	303	33.953	32.060	59.761	1.00	0.00	H
ATOM	4706	OG	SER	303	35.275	33.434	60.528	1.00	0.00	O
ATOM	4707	HG	SER	303	35.058	33.079	61.400	1.00	0.00	H
ATOM	4708	C	SER	303	33.381	33.111	57.279	1.00	0.00	C
ATOM	4709	O	SER	303	32.972	31.957	57.149	1.00	0.00	O
ATOM	4710	N	LYS	304	32.791	34.142	56.644	1.00	0.00	N
ATOM	4711	H	LYS	304	33.134	35.066	56.867	1.00	0.00	H
ATOM	4712	CA	LYS	304	31.820	33.999	55.533	1.00	0.00	C
ATOM	4713	HA	LYS	304	31.159	33.151	55.730	1.00	0.00	H
ATOM	4714	CB	LYS	304	30.960	35.275	55.386	1.00	0.00	C
ATOM	4715	HB2	LYS	304	31.615	36.130	55.206	1.00	0.00	H
ATOM	4716	HB3	LYS	304	30.333	35.147	54.502	1.00	0.00	H
ATOM	4717	CG	LYS	304	30.036	35.603	56.573	1.00	0.00	C
ATOM	4718	HG2	LYS	304	29.439	34.723	56.818	1.00	0.00	H
ATOM	4719	HG3	LYS	304	30.640	35.872	57.440	1.00	0.00	H
ATOM	4720	CD	LYS	304	29.091	36.772	56.219	1.00	0.00	C
ATOM	4721	HD2	LYS	304	29.683	37.642	55.927	1.00	0.00	H
ATOM	4722	HD3	LYS	304	28.465	36.474	55.376	1.00	0.00	H
ATOM	4723	CE	LYS	304	28.190	37.154	57.402	1.00	0.00	C
ATOM	4724	HE2	LYS	304	27.694	36.251	57.770	1.00	0.00	H
ATOM	4725	HE3	LYS	304	28.814	37.547	58.210	1.00	0.00	H
ATOM	4726	NZ	LYS	304	27.161	38.162	57.032	1.00	0.00	N
ATOM	4727	HZ1	LYS	304	27.538	39.053	56.703	1.00	0.00	H
ATOM	4728	HZ2	LYS	304	26.609	38.443	57.839	1.00	0.00	H
ATOM	4729	HZ3	LYS	304	26.522	37.843	56.308	1.00	0.00	H

ATOM	4730	C	LYS	304	32.498	33.717	54.179	1.00	0.00	C
ATOM	4731	O	LYS	304	32.026	32.888	53.402	1.00	0.00	O
ATOM	4732	N	LYS	305	33.584	34.441	53.882	1.00	0.00	N
ATOM	4733	H	LYS	305	34.005	34.953	54.647	1.00	0.00	H
ATOM	4734	CA	LYS	305	34.260	34.521	52.578	1.00	0.00	C
ATOM	4735	HA	LYS	305	34.156	33.559	52.074	1.00	0.00	H
ATOM	4736	CB	LYS	305	33.561	35.587	51.699	1.00	0.00	C
ATOM	4737	HB2	LYS	305	32.627	35.164	51.323	1.00	0.00	H
ATOM	4738	HB3	LYS	305	33.302	36.440	52.328	1.00	0.00	H
ATOM	4739	CG	LYS	305	34.368	36.138	50.505	1.00	0.00	C
ATOM	4740	HG2	LYS	305	33.735	36.838	49.958	1.00	0.00	H
ATOM	4741	HG3	LYS	305	35.204	36.715	50.902	1.00	0.00	H
ATOM	4742	CD	LYS	305	34.908	35.095	49.511	1.00	0.00	C
ATOM	4743	HD2	LYS	305	35.345	34.256	50.047	1.00	0.00	H
ATOM	4744	HD3	LYS	305	34.098	34.724	48.879	1.00	0.00	H
ATOM	4745	CE	LYS	305	35.994	35.762	48.655	1.00	0.00	C
ATOM	4746	HE2	LYS	305	35.527	36.442	47.936	1.00	0.00	H
ATOM	4747	HE3	LYS	305	36.636	36.351	49.315	1.00	0.00	H
ATOM	4748	NZ	LYS	305	36.842	34.779	47.948	1.00	0.00	N
ATOM	4749	HZ1	LYS	305	37.142	34.039	48.575	1.00	0.00	H
ATOM	4750	HZ2	LYS	305	37.685	35.238	47.616	1.00	0.00	H
ATOM	4751	HZ3	LYS	305	36.370	34.389	47.131	1.00	0.00	H
ATOM	4752	C	LYS	305	35.753	34.791	52.790	1.00	0.00	C
ATOM	4753	O	LYS	305	36.161	35.881	53.195	1.00	0.00	O
ATOM	4754	N	LEU	306	36.548	33.780	52.450	1.00	0.00	N
ATOM	4755	H	LEU	306	36.101	32.935	52.135	1.00	0.00	H
ATOM	4756	CA	LEU	306	38.007	33.761	52.520	1.00	0.00	C
ATOM	4757	HA	LEU	306	38.353	34.725	52.889	1.00	0.00	H
ATOM	4758	CB	LEU	306	38.440	32.703	53.561	1.00	0.00	C
ATOM	4759	HB2	LEU	306	39.524	32.730	53.676	1.00	0.00	H
ATOM	4760	HB3	LEU	306	38.020	33.018	54.518	1.00	0.00	H
ATOM	4761	CG	LEU	306	37.993	31.245	53.298	1.00	0.00	C
ATOM	4762	HG	LEU	306	36.952	31.233	52.977	1.00	0.00	H
ATOM	4763	CD1	LEU	306	38.837	30.534	52.236	1.00	0.00	C
ATOM	4764	HD11	LEU	306	38.658	30.965	51.254	1.00	0.00	H
ATOM	4765	HD12	LEU	306	38.555	29.483	52.191	1.00	0.00	H
ATOM	4766	HD13	LEU	306	39.896	30.607	52.482	1.00	0.00	H
ATOM	4767	CD2	LEU	306	38.081	30.434	54.589	1.00	0.00	C
ATOM	4768	HD21	LEU	306	39.106	30.400	54.954	1.00	0.00	H
ATOM	4769	HD22	LEU	306	37.453	30.887	55.358	1.00	0.00	H
ATOM	4770	HD23	LEU	306	37.726	29.417	54.421	1.00	0.00	H
ATOM	4771	C	LEU	306	38.638	33.589	51.130	1.00	0.00	C
ATOM	4772	O	LEU	306	37.955	33.359	50.128	1.00	0.00	O
ATOM	4773	N	ASN	307	39.958	33.719	51.082	1.00	0.00	N
ATOM	4774	H	ASN	307	40.432	33.954	51.948	1.00	0.00	H
ATOM	4775	CA	ASN	307	40.810	33.627	49.900	1.00	0.00	C
ATOM	4776	HA	ASN	307	40.263	33.144	49.088	1.00	0.00	H
ATOM	4777	CB	ASN	307	41.214	35.053	49.469	1.00	0.00	C
ATOM	4778	HB2	ASN	307	41.983	35.423	50.148	1.00	0.00	H
ATOM	4779	HB3	ASN	307	41.638	35.027	48.466	1.00	0.00	H
ATOM	4780	CG	ASN	307	40.061	36.045	49.475	1.00	0.00	C
ATOM	4781	OD1	ASN	307	39.261	36.110	48.553	1.00	0.00	O

ATOM 4782 ND2 ASN 307 39.915 36.815 50.530 1.00 0.00 N
ATOM 4783 HD21 ASN 307 40.502 36.661 51.346 1.00 0.00 H
ATOM 4784 HD22 ASN 307 39.184 37.501 50.524 1.00 0.00 H
ATOM 4785 C ASN 307 42.044 32.772 50.242 1.00 0.00 C
ATOM 4786 O ASN 307 42.346 32.593 51.421 1.00 0.00 O
ATOM 4787 N TYR 308 42.782 32.292 49.237 1.00 0.00 N
ATOM 4788 H TYR 308 42.490 32.458 48.287 1.00 0.00 H
ATOM 4789 CA TYR 308 44.099 31.670 49.435 1.00 0.00 C
ATOM 4790 HA TYR 308 44.390 31.776 50.479 1.00 0.00 H
ATOM 4791 CB TYR 308 44.038 30.167 49.142 1.00 0.00 C
ATOM 4792 HB2 TYR 308 43.152 29.750 49.624 1.00 0.00 H
ATOM 4793 HB3 TYR 308 43.930 30.005 48.069 1.00 0.00 H
ATOM 4794 CG TYR 308 45.253 29.412 49.650 1.00 0.00 C
ATOM 4795 CD1 TYR 308 46.381 29.224 48.828 1.00 0.00 C
ATOM 4796 HD1 TYR 308 46.391 29.619 47.822 1.00 0.00 H
ATOM 4797 CE1 TYR 308 47.496 28.511 49.314 1.00 0.00 C
ATOM 4798 HE1 TYR 308 48.363 28.351 48.691 1.00 0.00 H
ATOM 4799 CZ TYR 308 47.484 27.980 50.622 1.00 0.00 C
ATOM 4800 OH TYR 308 48.544 27.258 51.072 1.00 0.00 O
ATOM 4801 HH TYR 308 48.291 26.624 51.760 1.00 0.00 H
ATOM 4802 CE2 TYR 308 46.364 28.194 51.453 1.00 0.00 C
ATOM 4803 HE2 TYR 308 46.363 27.815 52.461 1.00 0.00 H
ATOM 4804 CD2 TYR 308 45.254 28.909 50.965 1.00 0.00 C
ATOM 4805 HD2 TYR 308 44.396 29.071 51.601 1.00 0.00 H
ATOM 4806 C TYR 308 45.140 32.404 48.585 1.00 0.00 C
ATOM 4807 O TYR 308 45.042 32.421 47.354 1.00 0.00 O
ATOM 4808 N THR 309 46.087 33.080 49.232 1.00 0.00 N
ATOM 4809 H THR 309 46.181 32.938 50.237 1.00 0.00 H
ATOM 4810 CA THR 309 46.954 34.096 48.606 1.00 0.00 C
ATOM 4811 HA THR 309 47.196 33.775 47.594 1.00 0.00 H
ATOM 4812 CB THR 309 46.172 35.426 48.496 1.00 0.00 C
ATOM 4813 HB THR 309 45.184 35.209 48.089 1.00 0.00 H
ATOM 4814 CG2 THR 309 45.979 36.157 49.826 1.00 0.00 C
ATOM 4815 HG21 THR 309 45.363 37.042 49.665 1.00 0.00 H
ATOM 4816 HG22 THR 309 46.937 36.469 50.238 1.00 0.00 H
ATOM 4817 HG23 THR 309 45.478 35.504 50.541 1.00 0.00 H
ATOM 4818 OG1 THR 309 46.806 36.314 47.600 1.00 0.00 O
ATOM 4819 HG1 THR 309 46.331 36.251 46.762 1.00 0.00 H
ATOM 4820 C THR 309 48.290 34.224 49.357 1.00 0.00 C
ATOM 4821 O THR 309 48.600 33.389 50.206 1.00 0.00 O
ATOM 4822 N ALA 310 49.101 35.237 49.040 1.00 0.00 N
ATOM 4823 H ALA 310 48.771 35.914 48.363 1.00 0.00 H
ATOM 4824 CA ALA 310 50.395 35.493 49.674 1.00 0.00 C
ATOM 4825 HA ALA 310 50.453 34.934 50.605 1.00 0.00 H
ATOM 4826 CB ALA 310 51.502 34.983 48.745 1.00 0.00 C
ATOM 4827 HB1 ALA 310 51.489 35.541 47.808 1.00 0.00 H
ATOM 4828 HB2 ALA 310 51.353 33.924 48.536 1.00 0.00 H
ATOM 4829 HB3 ALA 310 52.468 35.120 49.226 1.00 0.00 H
ATOM 4830 C ALA 310 50.564 36.981 50.012 1.00 0.00 C
ATOM 4831 O ALA 310 50.348 37.827 49.147 1.00 0.00 O
ATOM 4832 N ILE 311 50.927 37.313 51.256 1.00 0.00 N
ATOM 4833 H ILE 311 51.059 36.563 51.931 1.00 0.00 H

ATOM	4834	CA	ILE	311	50.938	38.697	51.765	1.00	0.00	C
ATOM	4835	HA	ILE	311	50.792	39.384	50.933	1.00	0.00	H
ATOM	4836	CB	ILE	311	49.768	38.922	52.760	1.00	0.00	C
ATOM	4837	HB	ILE	311	49.882	38.216	53.585	1.00	0.00	H
ATOM	4838	CG2	ILE	311	49.801	40.349	53.347	1.00	0.00	C
ATOM	4839	HG21	ILE	311	50.733	40.525	53.884	1.00	0.00	H
ATOM	4840	HG22	ILE	311	48.994	40.485	54.066	1.00	0.00	H
ATOM	4841	HG23	ILE	311	49.696	41.087	52.553	1.00	0.00	H
ATOM	4842	CG1	ILE	311	48.412	38.643	52.069	1.00	0.00	C
ATOM	4843	HG12	ILE	311	48.285	39.328	51.233	1.00	0.00	H
ATOM	4844	HG13	ILE	311	48.417	37.629	51.673	1.00	0.00	H
ATOM	4845	CD1	ILE	311	47.184	38.735	52.979	1.00	0.00	C
ATOM	4846	HD11	ILE	311	47.313	38.080	53.841	1.00	0.00	H
ATOM	4847	HD12	ILE	311	46.311	38.408	52.418	1.00	0.00	H
ATOM	4848	HD13	ILE	311	47.016	39.760	53.309	1.00	0.00	H
ATOM	4849	C	ILE	311	52.292	39.040	52.391	1.00	0.00	C
ATOM	4850	O	ILE	311	52.753	38.327	53.281	1.00	0.00	O
ATOM	4851	N	SER	312	52.923	40.133	51.945	1.00	0.00	N
ATOM	4852	H	SER	312	52.513	40.634	51.170	1.00	0.00	H
ATOM	4853	CA	SER	312	54.068	40.782	52.612	1.00	0.00	C
ATOM	4854	HA	SER	312	53.789	41.026	53.638	1.00	0.00	H
ATOM	4855	CB	SER	312	55.301	39.863	52.653	1.00	0.00	C
ATOM	4856	HB2	SER	312	56.087	40.332	53.247	1.00	0.00	H
ATOM	4857	HB3	SER	312	55.048	38.936	53.156	1.00	0.00	H
ATOM	4858	OG	SER	312	55.804	39.615	51.352	1.00	0.00	O
ATOM	4859	HG	SER	312	56.591	39.034	51.447	1.00	0.00	H
ATOM	4860	C	SER	312	54.498	42.076	51.913	1.00	0.00	C
ATOM	4861	O	SER	312	54.014	42.432	50.833	1.00	0.00	O
ATOM	4862	N	ASP	313	55.511	42.720	52.498	1.00	0.00	N
ATOM	4863	H	ASP	313	55.805	42.409	53.413	1.00	0.00	H
ATOM	4864	CA	ASP	313	56.266	43.830	51.919	1.00	0.00	C
ATOM	4865	HA	ASP	313	55.586	44.671	51.835	1.00	0.00	H
ATOM	4866	CB	ASP	313	57.367	44.211	52.924	1.00	0.00	C
ATOM	4867	HB2	ASP	313	56.945	44.224	53.930	1.00	0.00	H
ATOM	4868	HB3	ASP	313	58.161	43.462	52.896	1.00	0.00	H
ATOM	4869	CG	ASP	313	57.950	45.594	52.647	1.00	0.00	C
ATOM	4870	OD1	ASP	313	57.287	46.424	51.991	1.00	0.00	O
ATOM	4871	OD2	ASP	313	59.110	45.878	53.038	1.00	0.00	O
ATOM	4872	C	ASP	313	56.870	43.536	50.520	1.00	0.00	C
ATOM	4873	O	ASP	313	57.108	44.447	49.728	1.00	0.00	O
ATOM	4874	N	THR	314	57.069	42.265	50.147	1.00	0.00	N
ATOM	4875	H	THR	314	56.853	41.522	50.801	1.00	0.00	H
ATOM	4876	CA	THR	314	57.555	41.898	48.803	1.00	0.00	C
ATOM	4877	HA	THR	314	58.475	42.449	48.613	1.00	0.00	H
ATOM	4878	CB	THR	314	57.887	40.397	48.729	1.00	0.00	C
ATOM	4879	HB	THR	314	56.978	39.812	48.874	1.00	0.00	H
ATOM	4880	CG2	THR	314	58.537	40.010	47.401	1.00	0.00	C
ATOM	4881	HG21	THR	314	59.493	40.522	47.299	1.00	0.00	H
ATOM	4882	HG22	THR	314	57.899	40.280	46.563	1.00	0.00	H
ATOM	4883	HG23	THR	314	58.695	38.931	47.384	1.00	0.00	H
ATOM	4884	OG1	THR	314	58.827	40.075	49.731	1.00	0.00	O
ATOM	4885	HG1	THR	314	58.462	39.373	50.298	1.00	0.00	H

ATOM	4886	C	THR	314	56.549	42.296	47.712	1.00	0.00	C
ATOM	4887	O	THR	314	56.937	42.817	46.663	1.00	0.00	O
ATOM	4888	N	VAL	315	55.247	42.136	47.979	1.00	0.00	N
ATOM	4889	H	VAL	315	54.990	41.786	48.895	1.00	0.00	H
ATOM	4890	CA	VAL	315	54.164	42.576	47.078	1.00	0.00	C
ATOM	4891	HA	VAL	315	54.439	42.291	46.065	1.00	0.00	H
ATOM	4892	CB	VAL	315	52.817	41.916	47.445	1.00	0.00	C
ATOM	4893	HB	VAL	315	52.375	42.497	48.252	1.00	0.00	H
ATOM	4894	CG1	VAL	315	51.874	41.965	46.234	1.00	0.00	C
ATOM	4895	HG11	VAL	315	51.591	42.995	46.020	1.00	0.00	H
ATOM	4896	HG12	VAL	315	52.345	41.545	45.348	1.00	0.00	H
ATOM	4897	HG13	VAL	315	50.974	41.393	46.437	1.00	0.00	H
ATOM	4898	CG2	VAL	315	52.905	40.466	47.953	1.00	0.00	C
ATOM	4899	HG21	VAL	315	53.468	40.424	48.884	1.00	0.00	H
ATOM	4900	HG22	VAL	315	53.374	39.813	47.222	1.00	0.00	H
ATOM	4901	HG23	VAL	315	51.903	40.090	48.163	1.00	0.00	H
ATOM	4902	C	VAL	315	53.999	44.102	47.078	1.00	0.00	C
ATOM	4903	O	VAL	315	53.675	44.700	46.050	1.00	0.00	O
ATOM	4904	N	ASN	316	54.294	44.733	48.219	1.00	0.00	N
ATOM	4905	H	ASN	316	54.564	44.165	49.009	1.00	0.00	H
ATOM	4906	CA	ASN	316	54.271	46.183	48.415	1.00	0.00	C
ATOM	4907	HA	ASN	316	53.257	46.539	48.237	1.00	0.00	H
ATOM	4908	CB	ASN	316	54.694	46.459	49.871	1.00	0.00	C
ATOM	4909	HB2	ASN	316	54.323	45.662	50.506	1.00	0.00	H
ATOM	4910	HB3	ASN	316	55.779	46.478	49.941	1.00	0.00	H
ATOM	4911	CG	ASN	316	54.191	47.766	50.439	1.00	0.00	C
ATOM	4912	OD1	ASN	316	53.448	48.492	49.802	1.00	0.00	O
ATOM	4913	ND2	ASN	316	54.576	48.099	51.645	1.00	0.00	N
ATOM	4914	HD21	ASN	316	55.193	47.486	52.174	1.00	0.00	H
ATOM	4915	HD22	ASN	316	54.101	48.855	52.114	1.00	0.00	H
ATOM	4916	C	ASN	316	55.212	46.914	47.455	1.00	0.00	C
ATOM	4917	O	ASN	316	54.864	47.957	46.902	1.00	0.00	O
ATOM	4918	N	VAL	317	56.409	46.356	47.250	1.00	0.00	N
ATOM	4919	H	VAL	317	56.642	45.522	47.779	1.00	0.00	H
ATOM	4920	CA	VAL	317	57.379	46.904	46.300	1.00	0.00	C
ATOM	4921	HA	VAL	317	57.319	47.991	46.357	1.00	0.00	H
ATOM	4922	CB	VAL	317	58.825	46.520	46.669	1.00	0.00	C
ATOM	4923	HB	VAL	317	58.965	45.445	46.545	1.00	0.00	H
ATOM	4924	CG1	VAL	317	59.831	47.281	45.798	1.00	0.00	C
ATOM	4925	HG11	VAL	317	59.710	46.993	44.758	1.00	0.00	H
ATOM	4926	HG12	VAL	317	59.664	48.355	45.882	1.00	0.00	H
ATOM	4927	HG13	VAL	317	60.850	47.047	46.104	1.00	0.00	H
ATOM	4928	CG2	VAL	317	59.159	46.905	48.118	1.00	0.00	C
ATOM	4929	HG21	VAL	317	60.198	46.656	48.332	1.00	0.00	H
ATOM	4930	HG22	VAL	317	58.530	46.341	48.804	1.00	0.00	H
ATOM	4931	HG23	VAL	317	58.997	47.974	48.260	1.00	0.00	H
ATOM	4932	C	VAL	317	57.007	46.537	44.860	1.00	0.00	C
ATOM	4933	O	VAL	317	57.010	47.433	44.022	1.00	0.00	O
ATOM	4934	N	ALA	318	56.579	45.297	44.576	1.00	0.00	N
ATOM	4935	H	ALA	318	56.574	44.602	45.311	1.00	0.00	H
ATOM	4936	CA	ALA	318	56.235	44.858	43.213	1.00	0.00	C
ATOM	4937	HA	ALA	318	57.142	44.866	42.606	1.00	0.00	H

ATOM	4938	CB	ALA	318	55.699	43.419	43.267	1.00	0.00	C
ATOM	4939	HB1	ALA	318	56.415	42.759	43.751	1.00	0.00	H
ATOM	4940	HB2	ALA	318	55.525	43.058	42.252	1.00	0.00	H
ATOM	4941	HB3	ALA	318	54.761	43.386	43.821	1.00	0.00	H
ATOM	4942	C	ALA	318	55.212	45.781	42.520	1.00	0.00	C
ATOM	4943	O	ALA	318	55.434	46.232	41.396	1.00	0.00	O
ATOM	4944	N	SER	319	54.115	46.112	43.212	1.00	0.00	N
ATOM	4945	H	SER	319	53.997	45.711	44.137	1.00	0.00	H
ATOM	4946	CA	SER	319	53.067	46.996	42.679	1.00	0.00	C
ATOM	4947	HA	SER	319	52.788	46.642	41.690	1.00	0.00	H
ATOM	4948	CB	SER	319	51.832	46.895	43.580	1.00	0.00	C
ATOM	4949	HB2	SER	319	51.489	45.860	43.602	1.00	0.00	H
ATOM	4950	HB3	SER	319	52.102	47.194	44.595	1.00	0.00	H
ATOM	4951	OG	SER	319	50.779	47.719	43.130	1.00	0.00	O
ATOM	4952	HG	SER	319	50.539	47.470	42.227	1.00	0.00	H
ATOM	4953	C	SER	319	53.541	48.447	42.500	1.00	0.00	C
ATOM	4954	O	SER	319	53.230	49.086	41.497	1.00	0.00	O
ATOM	4955	N	ARG	320	54.370	48.968	43.413	1.00	0.00	N
ATOM	4956	H	ARG	320	54.657	48.389	44.192	1.00	0.00	H
ATOM	4957	CA	ARG	320	54.900	50.339	43.304	1.00	0.00	C
ATOM	4958	HA	ARG	320	54.098	50.982	42.934	1.00	0.00	H
ATOM	4959	CB	ARG	320	55.298	50.858	44.699	1.00	0.00	C
ATOM	4960	HB2	ARG	320	56.063	50.205	45.123	1.00	0.00	H
ATOM	4961	HB3	ARG	320	55.711	51.863	44.599	1.00	0.00	H
ATOM	4962	CG	ARG	320	54.075	50.910	45.646	1.00	0.00	C
ATOM	4963	HG2	ARG	320	53.307	51.554	45.217	1.00	0.00	H
ATOM	4964	HG3	ARG	320	53.655	49.909	45.737	1.00	0.00	H
ATOM	4965	CD	ARG	320	54.424	51.422	47.053	1.00	0.00	C
ATOM	4966	HD2	ARG	320	55.433	51.087	47.301	1.00	0.00	H
ATOM	4967	HD3	ARG	320	54.419	52.512	47.054	1.00	0.00	H
ATOM	4968	NE	ARG	320	53.508	50.876	48.077	1.00	0.00	N
ATOM	4969	HE	ARG	320	53.579	49.879	48.255	1.00	0.00	H
ATOM	4970	CZ	ARG	320	52.660	51.497	48.883	1.00	0.00	C
ATOM	4971	NH1	ARG	320	52.401	52.770	48.851	1.00	0.00	N
ATOM	4972	HH11	ARG	320	52.785	53.350	48.134	1.00	0.00	H
ATOM	4973	HH12	ARG	320	51.731	53.104	49.527	1.00	0.00	H
ATOM	4974	NH2	ARG	320	52.037	50.819	49.785	1.00	0.00	N
ATOM	4975	HH21	ARG	320	52.282	49.849	49.934	1.00	0.00	H
ATOM	4976	HH22	ARG	320	51.534	51.288	50.551	1.00	0.00	H
ATOM	4977	C	ARG	320	56.007	50.458	42.239	1.00	0.00	C
ATOM	4978	O	ARG	320	56.111	51.503	41.603	1.00	0.00	O
ATOM	4979	N	ILE	321	56.771	49.391	41.977	1.00	0.00	N
ATOM	4980	H	ILE	321	56.655	48.575	42.568	1.00	0.00	H
ATOM	4981	CA	ILE	321	57.722	49.299	40.851	1.00	0.00	C
ATOM	4982	HA	ILE	321	58.284	50.230	40.798	1.00	0.00	H
ATOM	4983	CB	ILE	321	58.723	48.138	41.084	1.00	0.00	C
ATOM	4984	HB	ILE	321	58.152	47.246	41.348	1.00	0.00	H
ATOM	4985	CG2	ILE	321	59.541	47.785	39.826	1.00	0.00	C
ATOM	4986	HG21	ILE	321	58.893	47.347	39.065	1.00	0.00	H
ATOM	4987	HG22	ILE	321	60.307	47.049	40.071	1.00	0.00	H
ATOM	4988	HG23	ILE	321	60.010	48.677	39.413	1.00	0.00	H
ATOM	4989	CG1	ILE	321	59.682	48.440	42.261	1.00	0.00	C

ATOM	4990	HG12	ILE	321	59.099	48.537	43.173	1.00	0.00	H
ATOM	4991	HG13	ILE	321	60.349	47.587	42.389	1.00	0.00	H
ATOM	4992	CD1	ILE	321	60.536	49.710	42.139	1.00	0.00	C
ATOM	4993	HD11	ILE	321	61.050	49.738	41.183	1.00	0.00	H
ATOM	4994	HD12	ILE	321	61.277	49.724	42.939	1.00	0.00	H
ATOM	4995	HD13	ILE	321	59.906	50.593	42.236	1.00	0.00	H
ATOM	4996	C	ILE	321	57.003	49.185	39.500	1.00	0.00	C
ATOM	4997	O	ILE	321	57.424	49.831	38.541	1.00	0.00	O
ATOM	4998	N	GLU	322	55.886	48.453	39.418	1.00	0.00	N
ATOM	4999	H	GLU	322	55.601	47.892	40.214	1.00	0.00	H
ATOM	5000	CA	GLU	322	55.010	48.481	38.236	1.00	0.00	C
ATOM	5001	HA	GLU	322	55.576	48.139	37.369	1.00	0.00	H
ATOM	5002	CB	GLU	322	53.823	47.522	38.444	1.00	0.00	C
ATOM	5003	HB2	GLU	322	54.221	46.512	38.515	1.00	0.00	H
ATOM	5004	HB3	GLU	322	53.346	47.734	39.396	1.00	0.00	H
ATOM	5005	CG	GLU	322	52.763	47.535	37.325	1.00	0.00	C
ATOM	5006	HG2	GLU	322	53.274	47.501	36.360	1.00	0.00	H
ATOM	5007	HG3	GLU	322	52.172	46.625	37.413	1.00	0.00	H
ATOM	5008	CD	GLU	322	51.783	48.721	37.362	1.00	0.00	C
ATOM	5009	OE1	GLU	322	51.519	49.285	38.446	1.00	0.00	O
ATOM	5010	OE2	GLU	322	51.318	49.118	36.271	1.00	0.00	O
ATOM	5011	C	GLU	322	54.552	49.914	37.938	1.00	0.00	C
ATOM	5012	O	GLU	322	54.713	50.398	36.818	1.00	0.00	O
ATOM	5013	N	THR	323	54.059	50.636	38.944	1.00	0.00	N
ATOM	5014	H	THR	323	53.850	50.154	39.814	1.00	0.00	H
ATOM	5015	CA	THR	323	53.565	52.008	38.769	1.00	0.00	C
ATOM	5016	HA	THR	323	52.919	52.023	37.895	1.00	0.00	H
ATOM	5017	CB	THR	323	52.670	52.364	39.964	1.00	0.00	C
ATOM	5018	HB	THR	323	53.170	52.074	40.889	1.00	0.00	H
ATOM	5019	CG2	THR	323	52.286	53.841	40.054	1.00	0.00	C
ATOM	5020	HG21	THR	323	53.167	54.446	40.268	1.00	0.00	H
ATOM	5021	HG22	THR	323	51.564	53.977	40.859	1.00	0.00	H
ATOM	5022	HG23	THR	323	51.839	54.162	39.112	1.00	0.00	H
ATOM	5023	OG1	THR	323	51.447	51.666	39.849	1.00	0.00	O
ATOM	5024	HG1	THR	323	51.589	50.782	39.442	1.00	0.00	H
ATOM	5025	C	THR	323	54.690	53.023	38.481	1.00	0.00	C
ATOM	5026	O	THR	323	54.456	54.027	37.807	1.00	0.00	O
ATOM	5027	N	ALA	324	55.944	52.748	38.861	1.00	0.00	N
ATOM	5028	H	ALA	324	56.103	51.934	39.444	1.00	0.00	H
ATOM	5029	CA	ALA	324	57.100	53.580	38.496	1.00	0.00	C
ATOM	5030	HA	ALA	324	56.887	54.606	38.799	1.00	0.00	H
ATOM	5031	CB	ALA	324	58.319	53.096	39.291	1.00	0.00	C
ATOM	5032	HB1	ALA	324	58.602	52.092	38.976	1.00	0.00	H
ATOM	5033	HB2	ALA	324	58.089	53.089	40.358	1.00	0.00	H
ATOM	5034	HB3	ALA	324	59.159	53.768	39.117	1.00	0.00	H
ATOM	5035	C	ALA	324	57.398	53.610	36.976	1.00	0.00	C
ATOM	5036	O	ALA	324	58.005	54.565	36.482	1.00	0.00	O
ATOM	5037	N	THR	325	56.936	52.611	36.212	1.00	0.00	N
ATOM	5038	H	THR	325	56.449	51.854	36.679	1.00	0.00	H
ATOM	5039	CA	THR	325	57.164	52.496	34.753	1.00	0.00	C
ATOM	5040	HA	THR	325	58.222	52.325	34.566	1.00	0.00	H
ATOM	5041	CB	THR	325	56.379	51.322	34.161	1.00	0.00	C

```
ATOM   5042  HB  THR   325      56.455  51.382  33.080  1.00  0.00           H
ATOM   5043  CG2 THR   325      56.877  49.940  34.579  1.00  0.00           C
ATOM   5044 HG21 THR   325      56.970  49.867  35.662  1.00  0.00           H
ATOM   5045 HG22 THR   325      57.851  49.759  34.129  1.00  0.00           H
ATOM   5046 HG23 THR   325      56.188  49.173  34.224  1.00  0.00           H
ATOM   5047  OG1 THR   325      55.016  51.446  34.483  1.00  0.00           O
ATOM   5048  HG1 THR   325      54.915  50.996  35.354  1.00  0.00           H
ATOM   5049  C   THR   325      56.761  53.733  33.944  1.00  0.00           C
ATOM   5050  O   THR   325      57.420  54.041  32.951  1.00  0.00           O
ATOM   5051  N   ARG   326      55.708  54.464  34.348  1.00  0.00           N
ATOM   5052  H   ARG   326      55.236  54.175  35.195  1.00  0.00           H
ATOM   5053  CA  ARG   326      55.215  55.644  33.606  1.00  0.00           C
ATOM   5054  HA  ARG   326      55.058  55.359  32.564  1.00  0.00           H
ATOM   5055  CB  ARG   326      53.880  56.180  34.170  1.00  0.00           C
ATOM   5056  HB2 ARG   326      54.036  56.557  35.182  1.00  0.00           H
ATOM   5057  HB3 ARG   326      53.596  57.036  33.555  1.00  0.00           H
ATOM   5058  CG  ARG   326      52.681  55.209  34.158  1.00  0.00           C
ATOM   5059  HG2 ARG   326      51.766  55.797  34.073  1.00  0.00           H
ATOM   5060  HG3 ARG   326      52.743  54.539  33.300  1.00  0.00           H
ATOM   5061  CD  ARG   326      52.602  54.402  35.457  1.00  0.00           C
ATOM   5062  HD2 ARG   326      53.464  53.741  35.521  1.00  0.00           H
ATOM   5063  HD3 ARG   326      52.647  55.095  36.299  1.00  0.00           H
ATOM   5064  NE  ARG   326      51.356  53.629  35.589  1.00  0.00           N
ATOM   5065  HE  ARG   326      50.493  54.152  35.776  1.00  0.00           H
ATOM   5066  CZ  ARG   326      51.265  52.318  35.719  1.00  0.00           C
ATOM   5067  NH1 ARG   326      52.225  51.508  35.395  1.00  0.00           N
ATOM   5068 HH11 ARG   326      53.094  51.828  35.013  1.00  0.00           H
ATOM   5069 HH12 ARG   326      52.054  50.523  35.611  1.00  0.00           H
ATOM   5070  NH2 ARG   326      50.215  51.742  36.204  1.00  0.00           N
ATOM   5071 HH21 ARG   326      49.386  52.282  36.453  1.00  0.00           H
ATOM   5072 HH22 ARG   326      50.252  50.731  36.348  1.00  0.00           H
ATOM   5073  C   ARG   326      56.237  56.786  33.574  1.00  0.00           C
ATOM   5074  O   ARG   326      56.240  57.558  32.616  1.00  0.00           O
ATOM   5075  N   ILE   327      57.086  56.894  34.601  1.00  0.00           N
ATOM   5076  H   ILE   327      57.034  56.191  35.327  1.00  0.00           H
ATOM   5077  CA  ILE   327      58.120  57.933  34.717  1.00  0.00           C
ATOM   5078  HA  ILE   327      57.731  58.868  34.307  1.00  0.00           H
ATOM   5079  CB  ILE   327      58.482  58.180  36.206  1.00  0.00           C
ATOM   5080  HB  ILE   327      58.831  57.238  36.634  1.00  0.00           H
ATOM   5081  CG2 ILE   327      59.624  59.208  36.340  1.00  0.00           C
ATOM   5082 HG21 ILE   327      60.511  58.885  35.796  1.00  0.00           H
ATOM   5083 HG22 ILE   327      59.919  59.312  37.384  1.00  0.00           H
ATOM   5084 HG23 ILE   327      59.297  60.181  35.968  1.00  0.00           H
ATOM   5085  CG1 ILE   327      57.239  58.645  37.005  1.00  0.00           C
ATOM   5086 HG12 ILE   327      56.898  59.606  36.617  1.00  0.00           H
ATOM   5087 HG13 ILE   327      56.432  57.925  36.870  1.00  0.00           H
ATOM   5088  CD1 ILE   327      57.469  58.773  38.517  1.00  0.00           C
ATOM   5089 HD11 ILE   327      57.895  57.849  38.909  1.00  0.00           H
ATOM   5090 HD12 ILE   327      56.515  58.960  39.010  1.00  0.00           H
ATOM   5091 HD13 ILE   327      58.136  59.607  38.735  1.00  0.00           H
ATOM   5092  C   ILE   327      59.353  57.549  33.889  1.00  0.00           C
ATOM   5093  O   ILE   327      59.829  58.343  33.078  1.00  0.00           O
```

ATOM	5094	N	PHE	328	59.860	56.327	34.081	1.00	0.00	N
ATOM	5095	H	PHE	328	59.384	55.728	34.743	1.00	0.00	H
ATOM	5096	CA	PHE	328	61.163	55.865	33.579	1.00	0.00	C
ATOM	5097	HA	PHE	328	61.806	56.741	33.481	1.00	0.00	H
ATOM	5098	CB	PHE	328	61.803	54.947	34.635	1.00	0.00	C
ATOM	5099	HB2	PHE	328	61.178	54.060	34.755	1.00	0.00	H
ATOM	5100	HB3	PHE	328	62.778	54.615	34.281	1.00	0.00	H
ATOM	5101	CG	PHE	328	62.009	55.615	35.981	1.00	0.00	C
ATOM	5102	CD1	PHE	328	62.902	56.699	36.090	1.00	0.00	C
ATOM	5103	HD1	PHE	328	63.445	57.038	35.220	1.00	0.00	H
ATOM	5104	CE1	PHE	328	63.082	57.345	37.326	1.00	0.00	C
ATOM	5105	HE1	PHE	328	63.759	58.182	37.417	1.00	0.00	H
ATOM	5106	CZ	PHE	328	62.377	56.899	38.456	1.00	0.00	C
ATOM	5107	HZ	PHE	328	62.519	57.392	39.408	1.00	0.00	H
ATOM	5108	CE2	PHE	328	61.491	55.814	38.353	1.00	0.00	C
ATOM	5109	HE2	PHE	328	60.953	55.478	39.228	1.00	0.00	H
ATOM	5110	CD2	PHE	328	61.305	55.171	37.116	1.00	0.00	C
ATOM	5111	HD2	PHE	328	60.619	54.340	37.041	1.00	0.00	H
ATOM	5112	C	PHE	328	61.137	55.234	32.172	1.00	0.00	C
ATOM	5113	O	PHE	328	61.986	54.403	31.859	1.00	0.00	O
ATOM	5114	N	ASN	329	60.174	55.619	31.327	1.00	0.00	N
ATOM	5115	H	ASN	329	59.553	56.338	31.672	1.00	0.00	H
ATOM	5116	CA	ASN	329	59.897	55.110	29.970	1.00	0.00	C
ATOM	5117	HA	ASN	329	59.001	55.632	29.628	1.00	0.00	H
ATOM	5118	CB	ASN	329	61.018	55.499	28.980	1.00	0.00	C
ATOM	5119	HB2	ASN	329	61.936	54.986	29.263	1.00	0.00	H
ATOM	5120	HB3	ASN	329	60.747	55.146	27.986	1.00	0.00	H
ATOM	5121	CG	ASN	329	61.290	56.992	28.863	1.00	0.00	C
ATOM	5122	OD1	ASN	329	60.456	57.780	28.441	1.00	0.00	O
ATOM	5123	ND2	ASN	329	62.490	57.420	29.169	1.00	0.00	N
ATOM	5124	HD21	ASN	329	63.210	56.741	29.401	1.00	0.00	H
ATOM	5125	HD22	ASN	329	62.717	58.391	29.039	1.00	0.00	H
ATOM	5126	C	ASN	329	59.553	53.605	29.857	1.00	0.00	C
ATOM	5127	O	ASN	329	58.558	53.284	29.205	1.00	0.00	O
ATOM	5128	N	SER	330	60.330	52.694	30.458	1.00	0.00	N
ATOM	5129	H	SER	330	61.120	53.053	30.985	1.00	0.00	H
ATOM	5130	CA	SER	330	60.263	51.235	30.248	1.00	0.00	C
ATOM	5131	HA	SER	330	60.478	51.045	29.196	1.00	0.00	H
ATOM	5132	CB	SER	330	61.338	50.517	31.073	1.00	0.00	C
ATOM	5133	HB2	SER	330	62.322	50.924	30.839	1.00	0.00	H
ATOM	5134	HB3	SER	330	61.135	50.662	32.135	1.00	0.00	H
ATOM	5135	OG	SER	330	61.301	49.134	30.767	1.00	0.00	O
ATOM	5136	HG	SER	330	61.476	48.611	31.576	1.00	0.00	H
ATOM	5137	C	SER	330	58.902	50.612	30.578	1.00	0.00	C
ATOM	5138	O	SER	330	58.274	50.951	31.584	1.00	0.00	O
ATOM	5139	N	LYS	331	58.444	49.659	29.754	1.00	0.00	N
ATOM	5140	H	LYS	331	59.048	49.377	28.983	1.00	0.00	H
ATOM	5141	CA	LYS	331	57.197	48.905	29.981	1.00	0.00	C
ATOM	5142	HA	LYS	331	56.473	49.586	30.419	1.00	0.00	H
ATOM	5143	CB	LYS	331	56.609	48.407	28.642	1.00	0.00	C
ATOM	5144	HB2	LYS	331	57.298	47.706	28.168	1.00	0.00	H
ATOM	5145	HB3	LYS	331	55.669	47.891	28.845	1.00	0.00	H

ATOM	5146	CG	LYS	331	56.338	49.591	27.695	1.00	0.00	C
ATOM	5147	HG2	LYS	331	55.856	50.382	28.270	1.00	0.00	H
ATOM	5148	HG3	LYS	331	57.295	49.974	27.335	1.00	0.00	H
ATOM	5149	CD	LYS	331	55.443	49.288	26.481	1.00	0.00	C
ATOM	5150	HD2	LYS	331	55.883	48.500	25.867	1.00	0.00	H
ATOM	5151	HD3	LYS	331	54.453	48.986	26.827	1.00	0.00	H
ATOM	5152	CE	LYS	331	55.358	50.602	25.690	1.00	0.00	C
ATOM	5153	HE2	LYS	331	55.180	51.399	26.416	1.00	0.00	H
ATOM	5154	HE3	LYS	331	56.328	50.808	25.231	1.00	0.00	H
ATOM	5155	NZ	LYS	331	54.284	50.667	24.663	1.00	0.00	N
ATOM	5156	HZ1	LYS	331	53.361	50.391	25.000	1.00	0.00	H
ATOM	5157	HZ2	LYS	331	54.488	50.149	23.825	1.00	0.00	H
ATOM	5158	HZ3	LYS	331	54.128	51.646	24.429	1.00	0.00	H
ATOM	5159	C	LYS	331	57.350	47.783	31.017	1.00	0.00	C
ATOM	5160	O	LYS	331	56.350	47.390	31.613	1.00	0.00	O
ATOM	5161	N	VAL	332	58.578	47.328	31.280	1.00	0.00	N
ATOM	5162	H	VAL	332	59.347	47.741	30.768	1.00	0.00	H
ATOM	5163	CA	VAL	332	58.910	46.309	32.293	1.00	0.00	C
ATOM	5164	HA	VAL	332	58.031	46.117	32.905	1.00	0.00	H
ATOM	5165	CB	VAL	332	59.333	44.974	31.636	1.00	0.00	C
ATOM	5166	HB	VAL	332	60.269	45.131	31.098	1.00	0.00	H
ATOM	5167	CG1	VAL	332	59.565	43.875	32.684	1.00	0.00	C
ATOM	5168	HG11	VAL	332	59.834	42.943	32.187	1.00	0.00	H
ATOM	5169	HG12	VAL	332	60.387	44.148	33.346	1.00	0.00	H
ATOM	5170	HG13	VAL	332	58.662	43.717	33.273	1.00	0.00	H
ATOM	5171	CG2	VAL	332	58.297	44.456	30.627	1.00	0.00	C
ATOM	5172	HG21	VAL	332	58.207	45.149	29.791	1.00	0.00	H
ATOM	5173	HG22	VAL	332	57.325	44.338	31.102	1.00	0.00	H
ATOM	5174	HG23	VAL	332	58.618	43.493	30.229	1.00	0.00	H
ATOM	5175	C	VAL	332	60.015	46.829	33.214	1.00	0.00	C
ATOM	5176	O	VAL	332	61.025	47.346	32.732	1.00	0.00	O
ATOM	5177	N	LEU	333	59.844	46.687	34.530	1.00	0.00	N
ATOM	5178	H	LEU	333	58.961	46.316	34.869	1.00	0.00	H
ATOM	5179	CA	LEU	333	60.856	47.036	35.532	1.00	0.00	C
ATOM	5180	HA	LEU	333	61.828	47.102	35.046	1.00	0.00	H
ATOM	5181	CB	LEU	333	60.526	48.399	36.167	1.00	0.00	C
ATOM	5182	HB2	LEU	333	59.487	48.379	36.501	1.00	0.00	H
ATOM	5183	HB3	LEU	333	61.150	48.515	37.049	1.00	0.00	H
ATOM	5184	CG	LEU	333	60.740	49.628	35.263	1.00	0.00	C
ATOM	5185	HG	LEU	333	60.154	49.525	34.351	1.00	0.00	H
ATOM	5186	CD1	LEU	333	60.267	50.883	36.001	1.00	0.00	C
ATOM	5187	HD11	LEU	333	59.226	50.763	36.297	1.00	0.00	H
ATOM	5188	HD12	LEU	333	60.359	51.750	35.349	1.00	0.00	H
ATOM	5189	HD13	LEU	333	60.867	51.039	36.895	1.00	0.00	H
ATOM	5190	CD2	LEU	333	62.209	49.843	34.887	1.00	0.00	C
ATOM	5191	HD21	LEU	333	62.826	49.880	35.783	1.00	0.00	H
ATOM	5192	HD22	LEU	333	62.556	49.025	34.255	1.00	0.00	H
ATOM	5193	HD23	LEU	333	62.322	50.773	34.331	1.00	0.00	H
ATOM	5194	C	LEU	333	60.982	45.950	36.607	1.00	0.00	C
ATOM	5195	O	LEU	333	59.989	45.363	37.038	1.00	0.00	O
ATOM	5196	N	ILE	334	62.208	45.697	37.069	1.00	0.00	N
ATOM	5197	H	ILE	334	62.989	46.202	36.659	1.00	0.00	H

```
ATOM   5198  CA  ILE   334      62.500  44.682  38.093  1.00  0.00           C
ATOM   5199  HA  ILE   334      61.569  44.449  38.609  1.00  0.00           H
ATOM   5200  CB  ILE   334      62.994  43.354  37.459  1.00  0.00           C
ATOM   5201  HB  ILE   334      62.983  42.612  38.256  1.00  0.00           H
ATOM   5202  CG2 ILE   334      62.024  42.852  36.373  1.00  0.00           C
ATOM   5203 HG21 ILE   334      60.996  42.935  36.722  1.00  0.00           H
ATOM   5204 HG22 ILE   334      62.223  41.806  36.143  1.00  0.00           H
ATOM   5205 HG23 ILE   334      62.134  43.443  35.463  1.00  0.00           H
ATOM   5206  CG1 ILE   334      64.441  43.441  36.923  1.00  0.00           C
ATOM   5207 HG12 ILE   334      64.496  44.227  36.168  1.00  0.00           H
ATOM   5208 HG13 ILE   334      65.106  43.705  37.744  1.00  0.00           H
ATOM   5209  CD1 ILE   334      64.979  42.141  36.308  1.00  0.00           C
ATOM   5210 HD11 ILE   334      64.930  41.334  37.039  1.00  0.00           H
ATOM   5211 HD12 ILE   334      66.016  42.290  36.011  1.00  0.00           H
ATOM   5212 HD13 ILE   334      64.410  41.870  35.419  1.00  0.00           H
ATOM   5213  C   ILE   334      63.452  45.228  39.164  1.00  0.00           C
ATOM   5214  O   ILE   334      64.330  46.047  38.882  1.00  0.00           O
ATOM   5215  N   HIE   335      63.280  44.774  40.407  1.00  0.00           N
ATOM   5216  H   HIE   335      62.506  44.140  40.580  1.00  0.00           H
ATOM   5217  CA  HIE   335      64.203  45.072  41.511  1.00  0.00           C
ATOM   5218  HA  HIE   335      64.552  46.101  41.403  1.00  0.00           H
ATOM   5219  CB  HIE   335      63.443  44.971  42.840  1.00  0.00           C
ATOM   5220  HB2 HIE   335      62.640  45.710  42.844  1.00  0.00           H
ATOM   5221  HB3 HIE   335      62.994  43.979  42.906  1.00  0.00           H
ATOM   5222  CG  HIE   335      64.306  45.182  44.061  1.00  0.00           C
ATOM   5223  ND1 HIE   335      64.804  44.160  44.873  1.00  0.00           N
ATOM   5224  CE1 HIE   335      65.630  44.756  45.750  1.00  0.00           C
ATOM   5225  HE1 HIE   335      66.181  44.241  46.529  1.00  0.00           H
ATOM   5226  NE2 HIE   335      65.704  46.075  45.505  1.00  0.00           N
ATOM   5227  HE2 HIE   335      66.323  46.729  45.992  1.00  0.00           H
ATOM   5228  CD2 HIE   335      64.873  46.363  44.443  1.00  0.00           C
ATOM   5229  HD2 HIE   335      64.747  47.322  43.956  1.00  0.00           H
ATOM   5230  C   HIE   335      65.453  44.175  41.488  1.00  0.00           C
ATOM   5231  O   HIE   335      65.445  43.084  40.919  1.00  0.00           O
ATOM   5232  N   SER   336      66.520  44.613  42.162  1.00  0.00           N
ATOM   5233  H   SER   336      66.439  45.527  42.590  1.00  0.00           H
ATOM   5234  CA  SER   336      67.823  43.934  42.250  1.00  0.00           C
ATOM   5235  HA  SER   336      68.300  43.990  41.272  1.00  0.00           H
ATOM   5236  CB  SER   336      68.693  44.682  43.267  1.00  0.00           C
ATOM   5237  HB2 SER   336      68.333  45.704  43.348  1.00  0.00           H
ATOM   5238  HB3 SER   336      68.608  44.221  44.253  1.00  0.00           H
ATOM   5239  OG  SER   336      70.046  44.710  42.860  1.00  0.00           O
ATOM   5240  HG  SER   336      70.487  43.929  43.268  1.00  0.00           H
ATOM   5241  C   SER   336      67.759  42.450  42.626  1.00  0.00           C
ATOM   5242  O   SER   336      68.501  41.651  42.059  1.00  0.00           O
ATOM   5243  N   HID   337      66.839  42.036  43.505  1.00  0.00           N
ATOM   5244  H   HID   337      66.216  42.718  43.925  1.00  0.00           H
ATOM   5245  CA  HID   337      66.663  40.615  43.845  1.00  0.00           C
ATOM   5246  HA  HID   337      67.601  40.222  44.240  1.00  0.00           H
ATOM   5247  CB  HID   337      65.581  40.478  44.924  1.00  0.00           C
ATOM   5248  HB2 HID   337      64.655  40.925  44.559  1.00  0.00           H
ATOM   5249  HB3 HID   337      65.390  39.418  45.089  1.00  0.00           H
```

ATOM	5250	CG	HID	337	65.912	41.090	46.262	1.00	0.00	C
ATOM	5251	ND1	HID	337	65.047	41.158	47.329	1.00	0.00	N
ATOM	5252	HD1	HID	337	64.091	40.802	47.338	1.00	0.00	H
ATOM	5253	CE1	HID	337	65.689	41.738	48.355	1.00	0.00	C
ATOM	5254	HE1	HID	337	65.262	41.923	49.333	1.00	0.00	H
ATOM	5255	NE2	HID	337	66.946	42.047	48.002	1.00	0.00	N
ATOM	5256	CD2	HID	337	67.104	41.624	46.679	1.00	0.00	C
ATOM	5257	HD2	HID	337	68.011	41.705	46.095	1.00	0.00	H
ATOM	5258	C	HID	337	66.298	39.755	42.623	1.00	0.00	C
ATOM	5259	O	HID	337	66.789	38.637	42.482	1.00	0.00	O
ATOM	5260	N	CYS	338	65.491	40.286	41.702	1.00	0.00	N
ATOM	5261	H	CYS	338	65.175	41.240	41.819	1.00	0.00	H
ATOM	5262	CA	CYS	338	65.112	39.610	40.460	1.00	0.00	C
ATOM	5263	HA	CYS	338	64.780	38.597	40.694	1.00	0.00	H
ATOM	5264	CB	CYS	338	63.945	40.372	39.824	1.00	0.00	C
ATOM	5265	HB2	CYS	338	64.293	41.360	39.523	1.00	0.00	H
ATOM	5266	HB3	CYS	338	63.594	39.836	38.941	1.00	0.00	H
ATOM	5267	SG	CYS	338	62.588	40.538	41.015	1.00	0.00	S
ATOM	5268	HG	CYS	338	61.907	41.503	40.374	1.00	0.00	H
ATOM	5269	C	CYS	338	66.274	39.491	39.460	1.00	0.00	C
ATOM	5270	O	CYS	338	66.208	38.672	38.551	1.00	0.00	O
ATOM	5271	N	VAL	339	67.338	40.286	39.618	1.00	0.00	N
ATOM	5272	H	VAL	339	67.318	40.964	40.368	1.00	0.00	H
ATOM	5273	CA	VAL	339	68.599	40.111	38.879	1.00	0.00	C
ATOM	5274	HA	VAL	339	68.387	39.682	37.898	1.00	0.00	H
ATOM	5275	CB	VAL	339	69.308	41.465	38.658	1.00	0.00	C
ATOM	5276	HB	VAL	339	69.538	41.913	39.624	1.00	0.00	H
ATOM	5277	CG1	VAL	339	70.621	41.312	37.880	1.00	0.00	C
ATOM	5278	HG11	VAL	339	70.432	40.831	36.919	1.00	0.00	H
ATOM	5279	HG12	VAL	339	71.068	42.292	37.710	1.00	0.00	H
ATOM	5280	HG13	VAL	339	71.331	40.709	38.446	1.00	0.00	H
ATOM	5281	CG2	VAL	339	68.422	42.443	37.877	1.00	0.00	C
ATOM	5282	HG21	VAL	339	68.169	42.019	36.906	1.00	0.00	H
ATOM	5283	HG22	VAL	339	68.954	43.382	37.730	1.00	0.00	H
ATOM	5284	HG23	VAL	339	67.508	42.656	38.432	1.00	0.00	H
ATOM	5285	C	VAL	339	69.517	39.124	39.608	1.00	0.00	C
ATOM	5286	O	VAL	339	70.054	38.206	39.001	1.00	0.00	O
ATOM	5287	N	GLN	340	69.699	39.289	40.919	1.00	0.00	N
ATOM	5288	H	GLN	340	69.221	40.061	41.371	1.00	0.00	H
ATOM	5289	CA	GLN	340	70.712	38.561	41.700	1.00	0.00	C
ATOM	5290	HA	GLN	340	71.629	38.515	41.109	1.00	0.00	H
ATOM	5291	CB	GLN	340	71.012	39.362	42.977	1.00	0.00	C
ATOM	5292	HB2	GLN	340	70.080	39.543	43.515	1.00	0.00	H
ATOM	5293	HB3	GLN	340	71.676	38.783	43.620	1.00	0.00	H
ATOM	5294	CG	GLN	340	71.688	40.707	42.647	1.00	0.00	C
ATOM	5295	HG2	GLN	340	72.680	40.517	42.238	1.00	0.00	H
ATOM	5296	HG3	GLN	340	71.115	41.246	41.895	1.00	0.00	H
ATOM	5297	CD	GLN	340	71.813	41.608	43.867	1.00	0.00	C
ATOM	5298	OE1	GLN	340	71.075	42.571	44.028	1.00	0.00	O
ATOM	5299	NE2	GLN	340	72.745	41.327	44.748	1.00	0.00	N
ATOM	5300	HE21	GLN	340	72.898	41.973	45.525	1.00	0.00	H
ATOM	5301	HE22	GLN	340	73.351	40.540	44.631	1.00	0.00	H

ATOM	5302	C	GLN	340	70.346	37.099	42.020	1.00	0.00	C
ATOM	5303	O	GLN	340	71.238	36.286	42.278	1.00	0.00	O
ATOM	5304	N	TYR	341	69.057	36.746	41.978	1.00	0.00	N
ATOM	5305	H	TYR	341	68.362	37.477	41.871	1.00	0.00	H
ATOM	5306	CA	TYR	341	68.575	35.360	42.074	1.00	0.00	C
ATOM	5307	HA	TYR	341	69.385	34.741	42.458	1.00	0.00	H
ATOM	5308	CB	TYR	341	67.433	35.280	43.102	1.00	0.00	C
ATOM	5309	HB2	TYR	341	66.574	35.815	42.697	1.00	0.00	H
ATOM	5310	HB3	TYR	341	67.141	34.235	43.221	1.00	0.00	H
ATOM	5311	CG	TYR	341	67.760	35.834	44.487	1.00	0.00	C
ATOM	5312	CD1	TYR	341	68.892	35.377	45.195	1.00	0.00	C
ATOM	5313	HD1	TYR	341	69.539	34.628	44.760	1.00	0.00	H
ATOM	5314	CE1	TYR	341	69.190	35.896	46.472	1.00	0.00	C
ATOM	5315	HE1	TYR	341	70.059	35.551	47.013	1.00	0.00	H
ATOM	5316	CZ	TYR	341	68.357	36.876	47.051	1.00	0.00	C
ATOM	5317	OH	TYR	341	68.650	37.387	48.279	1.00	0.00	O
ATOM	5318	HH	TYR	341	68.140	38.181	48.454	1.00	0.00	H
ATOM	5319	CE2	TYR	341	67.218	37.329	46.350	1.00	0.00	C
ATOM	5320	HE2	TYR	341	66.572	38.071	46.794	1.00	0.00	H
ATOM	5321	CD2	TYR	341	66.921	36.802	45.076	1.00	0.00	C
ATOM	5322	HD2	TYR	341	66.046	37.142	44.540	1.00	0.00	H
ATOM	5323	C	TYR	341	68.221	34.741	40.701	1.00	0.00	C
ATOM	5324	O	TYR	341	67.648	33.653	40.639	1.00	0.00	O
ATOM	5325	N	LEU	342	68.568	35.408	39.592	1.00	0.00	N
ATOM	5326	H	LEU	342	69.080	36.276	39.692	1.00	0.00	H
ATOM	5327	CA	LEU	342	68.465	34.870	38.233	1.00	0.00	C
ATOM	5328	HA	LEU	342	67.693	34.102	38.220	1.00	0.00	H
ATOM	5329	CB	LEU	342	68.037	36.009	37.290	1.00	0.00	C
ATOM	5330	HB2	LEU	342	67.108	36.444	37.654	1.00	0.00	H
ATOM	5331	HB3	LEU	342	68.803	36.782	37.328	1.00	0.00	H
ATOM	5332	CG	LEU	342	67.854	35.599	35.821	1.00	0.00	C
ATOM	5333	HG	LEU	342	68.730	35.054	35.470	1.00	0.00	H
ATOM	5334	CD1	LEU	342	66.615	34.729	35.622	1.00	0.00	C
ATOM	5335	HD11	LEU	342	66.671	33.848	36.254	1.00	0.00	H
ATOM	5336	HD12	LEU	342	66.553	34.425	34.577	1.00	0.00	H
ATOM	5337	HD13	LEU	342	65.722	35.298	35.879	1.00	0.00	H
ATOM	5338	CD2	LEU	342	67.695	36.844	34.963	1.00	0.00	C
ATOM	5339	HD21	LEU	342	66.848	37.435	35.308	1.00	0.00	H
ATOM	5340	HD22	LEU	342	68.602	37.443	35.042	1.00	0.00	H
ATOM	5341	HD23	LEU	342	67.547	36.554	33.924	1.00	0.00	H
ATOM	5342	C	LEU	342	69.794	34.238	37.779	1.00	0.00	C
ATOM	5343	O	LEU	342	70.854	34.839	37.921	1.00	0.00	O
ATOM	5344	N	GLY	343	69.731	33.049	37.171	1.00	0.00	N
ATOM	5345	H	GLY	343	68.838	32.590	37.131	1.00	0.00	H
ATOM	5346	CA	GLY	343	70.879	32.415	36.501	1.00	0.00	C
ATOM	5347	HA2	GLY	343	71.805	32.722	36.990	1.00	0.00	H
ATOM	5348	HA3	GLY	343	70.798	31.334	36.608	1.00	0.00	H
ATOM	5349	C	GLY	343	71.000	32.746	34.999	1.00	0.00	C
ATOM	5350	O	GLY	343	72.066	33.190	34.564	1.00	0.00	O
ATOM	5351	N	PRO	344	69.940	32.530	34.186	1.00	0.00	N
ATOM	5352	CD	PRO	344	68.739	31.778	34.530	1.00	0.00	C
ATOM	5353	HD2	PRO	344	67.994	32.457	34.938	1.00	0.00	H

ATOM 5354 HD3 PRO 344 68.949 30.976 35.238 1.00 0.00 H
ATOM 5355 CG PRO 344 68.228 31.186 33.217 1.00 0.00 C
ATOM 5356 HG2 PRO 344 67.152 31.019 33.234 1.00 0.00 H
ATOM 5357 HG3 PRO 344 68.760 30.258 32.999 1.00 0.00 H
ATOM 5358 CB PRO 344 68.623 32.258 32.206 1.00 0.00 C
ATOM 5359 HB2 PRO 344 67.908 33.082 32.248 1.00 0.00 H
ATOM 5360 HB3 PRO 344 68.691 31.856 31.194 1.00 0.00 H
ATOM 5361 CA PRO 344 69.987 32.712 32.732 1.00 0.00 C
ATOM 5362 HA PRO 344 70.742 32.022 32.351 1.00 0.00 H
ATOM 5363 C PRO 344 70.341 34.125 32.243 1.00 0.00 C
ATOM 5364 O PRO 344 69.907 35.138 32.791 1.00 0.00 O
ATOM 5365 N LYS 345 71.109 34.171 31.150 1.00 0.00 N
ATOM 5366 H LYS 345 71.384 33.285 30.755 1.00 0.00 H
ATOM 5367 CA LYS 345 71.709 35.376 30.553 1.00 0.00 C
ATOM 5368 HA LYS 345 72.127 35.991 31.353 1.00 0.00 H
ATOM 5369 CB LYS 345 72.867 34.940 29.637 1.00 0.00 C
ATOM 5370 HB2 LYS 345 72.498 34.191 28.933 1.00 0.00 H
ATOM 5371 HB3 LYS 345 73.225 35.798 29.063 1.00 0.00 H
ATOM 5372 CG LYS 345 74.049 34.372 30.441 1.00 0.00 C
ATOM 5373 HG2 LYS 345 74.629 35.196 30.858 1.00 0.00 H
ATOM 5374 HG3 LYS 345 73.683 33.756 31.263 1.00 0.00 H
ATOM 5375 CD LYS 345 74.944 33.492 29.565 1.00 0.00 C
ATOM 5376 HD2 LYS 345 74.353 32.644 29.213 1.00 0.00 H
ATOM 5377 HD3 LYS 345 75.304 34.055 28.702 1.00 0.00 H
ATOM 5378 CE LYS 345 76.136 32.985 30.382 1.00 0.00 C
ATOM 5379 HE2 LYS 345 76.929 33.737 30.353 1.00 0.00 H
ATOM 5380 HE3 LYS 345 75.825 32.846 31.423 1.00 0.00 H
ATOM 5381 NZ LYS 345 76.621 31.694 29.851 1.00 0.00 N
ATOM 5382 HZ1 LYS 345 76.679 31.712 28.834 1.00 0.00 H
ATOM 5383 HZ2 LYS 345 75.996 30.947 30.145 1.00 0.00 H
ATOM 5384 HZ3 LYS 345 77.540 31.458 30.213 1.00 0.00 H
ATOM 5385 C LYS 345 70.688 36.254 29.813 1.00 0.00 C
ATOM 5386 O LYS 345 70.556 36.197 28.589 1.00 0.00 O
ATOM 5387 N ILE 346 69.979 37.095 30.562 1.00 0.00 N
ATOM 5388 H ILE 346 70.049 36.983 31.566 1.00 0.00 H
ATOM 5389 CA ILE 346 69.280 38.280 30.033 1.00 0.00 C
ATOM 5390 HA ILE 346 68.874 38.034 29.051 1.00 0.00 H
ATOM 5391 CB ILE 346 68.093 38.681 30.939 1.00 0.00 C
ATOM 5392 HB ILE 346 67.623 39.558 30.492 1.00 0.00 H
ATOM 5393 CG2 ILE 346 67.043 37.556 30.951 1.00 0.00 C
ATOM 5394 HG21 ILE 346 66.778 37.282 29.930 1.00 0.00 H
ATOM 5395 HG22 ILE 346 66.137 37.891 31.453 1.00 0.00 H
ATOM 5396 HG23 ILE 346 67.429 36.676 31.467 1.00 0.00 H
ATOM 5397 CG1 ILE 346 68.560 39.070 32.360 1.00 0.00 C
ATOM 5398 HG12 ILE 346 68.932 38.184 32.870 1.00 0.00 H
ATOM 5399 HG13 ILE 346 69.387 39.771 32.289 1.00 0.00 H
ATOM 5400 CD1 ILE 346 67.480 39.738 33.218 1.00 0.00 C
ATOM 5401 HD11 ILE 346 67.179 40.679 32.761 1.00 0.00 H
ATOM 5402 HD12 ILE 346 67.881 39.944 34.210 1.00 0.00 H
ATOM 5403 HD13 ILE 346 66.614 39.089 33.325 1.00 0.00 H
ATOM 5404 C ILE 346 70.253 39.458 29.839 1.00 0.00 C
ATOM 5405 O ILE 346 71.307 39.527 30.481 1.00 0.00 O

ATOM	5406	N	LYS	347	69.884	40.434	29.001	1.00	0.00	N
ATOM	5407	H	LYS	347	69.011	40.329	28.501	1.00	0.00	H
ATOM	5408	CA	LYS	347	70.581	41.731	28.887	1.00	0.00	C
ATOM	5409	HA	LYS	347	71.648	41.579	29.058	1.00	0.00	H
ATOM	5410	CB	LYS	347	70.427	42.314	27.465	1.00	0.00	C
ATOM	5411	HB2	LYS	347	69.369	42.448	27.231	1.00	0.00	H
ATOM	5412	HB3	LYS	347	70.905	43.295	27.448	1.00	0.00	H
ATOM	5413	CG	LYS	347	71.087	41.438	26.387	1.00	0.00	C
ATOM	5414	HG2	LYS	347	72.091	41.161	26.714	1.00	0.00	H
ATOM	5415	HG3	LYS	347	70.499	40.530	26.247	1.00	0.00	H
ATOM	5416	CD	LYS	347	71.198	42.189	25.051	1.00	0.00	C
ATOM	5417	HD2	LYS	347	70.206	42.515	24.729	1.00	0.00	H
ATOM	5418	HD3	LYS	347	71.833	43.067	25.188	1.00	0.00	H
ATOM	5419	CE	LYS	347	71.812	41.278	23.981	1.00	0.00	C
ATOM	5420	HE2	LYS	347	72.734	40.839	24.376	1.00	0.00	H
ATOM	5421	HE3	LYS	347	71.115	40.464	23.764	1.00	0.00	H
ATOM	5422	NZ	LYS	347	72.125	42.027	22.743	1.00	0.00	N
ATOM	5423	HZ1	LYS	347	72.873	42.698	22.917	1.00	0.00	H
ATOM	5424	HZ2	LYS	347	71.324	42.526	22.385	1.00	0.00	H
ATOM	5425	HZ3	LYS	347	72.467	41.400	22.019	1.00	0.00	H
ATOM	5426	C	LYS	347	70.122	42.713	29.980	1.00	0.00	C
ATOM	5427	O	LYS	347	69.555	43.755	29.679	1.00	0.00	O
ATOM	5428	N	ALA	348	70.332	42.358	31.251	1.00	0.00	N
ATOM	5429	H	ALA	348	70.804	41.485	31.427	1.00	0.00	H
ATOM	5430	CA	ALA	348	69.904	43.168	32.399	1.00	0.00	C
ATOM	5431	HA	ALA	348	68.819	43.286	32.351	1.00	0.00	H
ATOM	5432	CB	ALA	348	70.244	42.428	33.701	1.00	0.00	C
ATOM	5433	HB1	ALA	348	69.932	43.033	34.554	1.00	0.00	H
ATOM	5434	HB2	ALA	348	71.319	42.255	33.766	1.00	0.00	H
ATOM	5435	HB3	ALA	348	69.721	41.477	33.746	1.00	0.00	H
ATOM	5436	C	ALA	348	70.537	44.575	32.382	1.00	0.00	C
ATOM	5437	O	ALA	348	71.735	44.737	32.625	1.00	0.00	O
ATOM	5438	N	ARG	349	69.713	45.590	32.116	1.00	0.00	N
ATOM	5439	H	ARG	349	68.739	45.356	31.927	1.00	0.00	H
ATOM	5440	CA	ARG	349	70.079	47.007	32.040	1.00	0.00	C
ATOM	5441	HA	ARG	349	71.146	47.090	31.828	1.00	0.00	H
ATOM	5442	CB	ARG	349	69.290	47.611	30.865	1.00	0.00	C
ATOM	5443	HB2	ARG	349	69.480	47.000	29.980	1.00	0.00	H
ATOM	5444	HB3	ARG	349	68.222	47.561	31.079	1.00	0.00	H
ATOM	5445	CG	ARG	349	69.670	49.061	30.540	1.00	0.00	C
ATOM	5446	HG2	ARG	349	69.409	49.715	31.373	1.00	0.00	H
ATOM	5447	HG3	ARG	349	70.745	49.115	30.362	1.00	0.00	H
ATOM	5448	CD	ARG	349	68.923	49.515	29.279	1.00	0.00	C
ATOM	5449	HD2	ARG	349	69.043	48.756	28.504	1.00	0.00	H
ATOM	5450	HD3	ARG	349	67.859	49.595	29.497	1.00	0.00	H
ATOM	5451	NE	ARG	349	69.458	50.786	28.762	1.00	0.00	N
ATOM	5452	HE	ARG	349	70.352	50.755	28.288	1.00	0.00	H
ATOM	5453	CZ	ARG	349	68.914	51.986	28.812	1.00	0.00	C
ATOM	5454	NH1	ARG	349	67.781	52.254	29.385	1.00	0.00	N
ATOM	5455	HH11	ARG	349	67.224	51.513	29.817	1.00	0.00	H
ATOM	5456	HH12	ARG	349	67.420	53.189	29.369	1.00	0.00	H
ATOM	5457	NH2	ARG	349	69.540	52.976	28.254	1.00	0.00	N

ATOM	5458	HH21	ARG	349	70.414	52.791	27.780	1.00	0.00	H
ATOM	5459	HH22	ARG	349	69.114	53.895	28.271	1.00	0.00	H
ATOM	5460	C	ARG	349	69.781	47.693	33.372	1.00	0.00	C
ATOM	5461	O	ARG	349	68.637	47.725	33.810	1.00	0.00	O
ATOM	5462	N	PHE	350	70.793	48.248	34.032	1.00	0.00	N
ATOM	5463	H	PHE	350	71.712	48.200	33.625	1.00	0.00	H
ATOM	5464	CA	PHE	350	70.593	49.107	35.205	1.00	0.00	C
ATOM	5465	HA	PHE	350	69.895	48.628	35.894	1.00	0.00	H
ATOM	5466	CB	PHE	350	71.938	49.283	35.925	1.00	0.00	C
ATOM	5467	HB2	PHE	350	72.239	48.320	36.341	1.00	0.00	H
ATOM	5468	HB3	PHE	350	72.692	49.569	35.190	1.00	0.00	H
ATOM	5469	CG	PHE	350	71.939	50.314	37.038	1.00	0.00	C
ATOM	5470	CD1	PHE	350	71.375	50.010	38.292	1.00	0.00	C
ATOM	5471	HD1	PHE	350	70.912	49.048	38.460	1.00	0.00	H
ATOM	5472	CE1	PHE	350	71.411	50.958	39.330	1.00	0.00	C
ATOM	5473	HE1	PHE	350	70.973	50.721	40.289	1.00	0.00	H
ATOM	5474	CZ	PHE	350	72.005	52.214	39.116	1.00	0.00	C
ATOM	5475	HZ	PHE	350	72.040	52.945	39.912	1.00	0.00	H
ATOM	5476	CE2	PHE	350	72.550	52.528	37.860	1.00	0.00	C
ATOM	5477	HE2	PHE	350	73.001	53.497	37.693	1.00	0.00	H
ATOM	5478	CD2	PHE	350	72.515	51.581	36.822	1.00	0.00	C
ATOM	5479	HD2	PHE	350	72.933	51.832	35.856	1.00	0.00	H
ATOM	5480	C	PHE	350	69.987	50.456	34.786	1.00	0.00	C
ATOM	5481	O	PHE	350	70.464	51.060	33.827	1.00	0.00	O
ATOM	5482	N	MET	351	68.968	50.935	35.510	1.00	0.00	N
ATOM	5483	H	MET	351	68.596	50.360	36.259	1.00	0.00	H
ATOM	5484	CA	MET	351	68.299	52.216	35.227	1.00	0.00	C
ATOM	5485	HA	MET	351	68.839	52.715	34.422	1.00	0.00	H
ATOM	5486	CB	MET	351	66.858	51.964	34.741	1.00	0.00	C
ATOM	5487	HB2	MET	351	66.539	50.956	35.014	1.00	0.00	H
ATOM	5488	HB3	MET	351	66.171	52.667	35.212	1.00	0.00	H
ATOM	5489	CG	MET	351	66.760	52.126	33.219	1.00	0.00	C
ATOM	5490	HG2	MET	351	67.502	51.476	32.753	1.00	0.00	H
ATOM	5491	HG3	MET	351	65.776	51.785	32.899	1.00	0.00	H
ATOM	5492	SD	MET	351	67.006	53.824	32.601	1.00	0.00	S
ATOM	5493	CE	MET	351	65.422	54.575	33.063	1.00	0.00	C
ATOM	5494	HE1	MET	351	65.311	54.573	34.147	1.00	0.00	H
ATOM	5495	HE2	MET	351	65.385	55.602	32.700	1.00	0.00	H
ATOM	5496	HE3	MET	351	64.604	54.010	32.613	1.00	0.00	H
ATOM	5497	C	MET	351	68.334	53.221	36.386	1.00	0.00	C
ATOM	5498	O	MET	351	68.223	54.423	36.132	1.00	0.00	O
ATOM	5499	N	GLY	352	68.526	52.786	37.637	1.00	0.00	N
ATOM	5500	H	GLY	352	68.589	51.791	37.816	1.00	0.00	H
ATOM	5501	CA	GLY	352	68.742	53.711	38.754	1.00	0.00	C
ATOM	5502	HA2	GLY	352	69.742	54.137	38.667	1.00	0.00	H
ATOM	5503	HA3	GLY	352	68.024	54.528	38.677	1.00	0.00	H
ATOM	5504	C	GLY	352	68.605	53.104	40.151	1.00	0.00	C
ATOM	5505	O	GLY	352	68.536	51.887	40.329	1.00	0.00	O
ATOM	5506	N	GLN	353	68.546	53.986	41.149	1.00	0.00	N
ATOM	5507	H	GLN	353	68.601	54.970	40.924	1.00	0.00	H
ATOM	5508	CA	GLN	353	68.164	53.687	42.532	1.00	0.00	C
ATOM	5509	HA	GLN	353	67.747	52.686	42.578	1.00	0.00	H

ATOM	5510	CB	GLN	353	69.377	53.756	43.481	1.00	0.00	C
ATOM	5511	HB2	GLN	353	69.725	54.788	43.546	1.00	0.00	H
ATOM	5512	HB3	GLN	353	69.043	53.446	44.471	1.00	0.00	H
ATOM	5513	CG	GLN	353	70.562	52.868	43.058	1.00	0.00	C
ATOM	5514	HG2	GLN	353	70.207	51.853	42.894	1.00	0.00	H
ATOM	5515	HG3	GLN	353	70.966	53.240	42.117	1.00	0.00	H
ATOM	5516	CD	GLN	353	71.701	52.825	44.080	1.00	0.00	C
ATOM	5517	OE1	GLN	353	71.602	53.247	45.223	1.00	0.00	O
ATOM	5518	NE2	GLN	353	72.861	52.328	43.704	1.00	0.00	N
ATOM	5519	HE21	GLN	353	73.608	52.367	44.379	1.00	0.00	H
ATOM	5520	HE22	GLN	353	73.016	52.042	42.754	1.00	0.00	H
ATOM	5521	C	GLN	353	67.082	54.696	42.946	1.00	0.00	C
ATOM	5522	O	GLN	353	67.256	55.885	42.681	1.00	0.00	O
ATOM	5523	N	ILE	354	65.974	54.246	43.548	1.00	0.00	N
ATOM	5524	H	ILE	354	65.880	53.248	43.704	1.00	0.00	H
ATOM	5525	CA	ILE	354	64.812	55.105	43.858	1.00	0.00	C
ATOM	5526	HA	ILE	354	65.139	56.145	43.805	1.00	0.00	H
ATOM	5527	CB	ILE	354	63.685	54.955	42.803	1.00	0.00	C
ATOM	5528	HB	ILE	354	62.966	55.747	43.015	1.00	0.00	H
ATOM	5529	CG2	ILE	354	64.208	55.203	41.376	1.00	0.00	C
ATOM	5530	HG21	ILE	354	64.786	56.128	41.353	1.00	0.00	H
ATOM	5531	HG22	ILE	354	63.375	55.308	40.683	1.00	0.00	H
ATOM	5532	HG23	ILE	354	64.837	54.376	41.050	1.00	0.00	H
ATOM	5533	CG1	ILE	354	62.922	53.612	42.895	1.00	0.00	C
ATOM	5534	HG12	ILE	354	63.552	52.804	42.521	1.00	0.00	H
ATOM	5535	HG13	ILE	354	62.678	53.398	43.934	1.00	0.00	H
ATOM	5536	CD1	ILE	354	61.592	53.622	42.129	1.00	0.00	C
ATOM	5537	HD11	ILE	354	60.960	54.433	42.494	1.00	0.00	H
ATOM	5538	HD12	ILE	354	61.077	52.676	42.293	1.00	0.00	H
ATOM	5539	HD13	ILE	354	61.761	53.748	41.061	1.00	0.00	H
ATOM	5540	C	ILE	354	64.283	54.920	45.288	1.00	0.00	C
ATOM	5541	O	ILE	354	64.459	53.869	45.906	1.00	0.00	O
ATOM	5542	N	GLU	355	63.613	55.948	45.810	1.00	0.00	N
ATOM	5543	H	GLU	355	63.577	56.810	45.274	1.00	0.00	H
ATOM	5544	CA	GLU	355	63.004	55.964	47.148	1.00	0.00	C
ATOM	5545	HA	GLU	355	63.500	55.222	47.776	1.00	0.00	H
ATOM	5546	CB	GLU	355	63.204	57.342	47.803	1.00	0.00	C
ATOM	5547	HB2	GLU	355	62.715	58.082	47.169	1.00	0.00	H
ATOM	5548	HB3	GLU	355	62.702	57.358	48.771	1.00	0.00	H
ATOM	5549	CG	GLU	355	64.683	57.711	48.018	1.00	0.00	C
ATOM	5550	HG2	GLU	355	65.017	57.258	48.953	1.00	0.00	H
ATOM	5551	HG3	GLU	355	65.297	57.289	47.221	1.00	0.00	H
ATOM	5552	CD	GLU	355	64.938	59.224	48.033	1.00	0.00	C
ATOM	5553	OE1	GLU	355	64.359	59.962	47.201	1.00	0.00	O
ATOM	5554	OE2	GLU	355	65.812	59.678	48.811	1.00	0.00	O
ATOM	5555	C	GLU	355	61.499	55.644	47.118	1.00	0.00	C
ATOM	5556	O	GLU	355	60.759	56.148	46.266	1.00	0.00	O
ATOM	5557	N	ALA	356	61.034	54.871	48.103	1.00	0.00	N
ATOM	5558	H	ALA	356	61.699	54.493	48.765	1.00	0.00	H
ATOM	5559	CA	ALA	356	59.624	54.630	48.424	1.00	0.00	C
ATOM	5560	HA	ALA	356	59.070	55.566	48.346	1.00	0.00	H
ATOM	5561	CB	ALA	356	59.048	53.612	47.425	1.00	0.00	C

ATOM	5562	HB1	ALA	356	59.141	53.993	46.407	1.00	0.00	H
ATOM	5563	HB2	ALA	356	57.991	53.440	47.634	1.00	0.00	H
ATOM	5564	HB3	ALA	356	59.589	52.669	47.502	1.00	0.00	H
ATOM	5565	C	ALA	356	59.495	54.117	49.872	1.00	0.00	C
ATOM	5566	O	ALA	356	60.495	53.702	50.469	1.00	0.00	O
ATOM	5567	N	LYS	357	58.267	54.063	50.424	1.00	0.00	N
ATOM	5568	H	LYS	357	57.504	54.488	49.915	1.00	0.00	H
ATOM	5569	CA	LYS	357	58.013	53.558	51.799	1.00	0.00	C
ATOM	5570	HA	LYS	357	56.945	53.632	52.007	1.00	0.00	H
ATOM	5571	CB	LYS	357	58.408	52.058	51.776	1.00	0.00	C
ATOM	5572	HB2	LYS	357	58.582	51.771	50.737	1.00	0.00	H
ATOM	5573	HB3	LYS	357	59.361	51.925	52.285	1.00	0.00	H
ATOM	5574	CG	LYS	357	57.383	51.037	52.301	1.00	0.00	C
ATOM	5575	HG2	LYS	357	57.449	50.955	53.382	1.00	0.00	H
ATOM	5576	HG3	LYS	357	56.372	51.351	52.034	1.00	0.00	H
ATOM	5577	CD	LYS	357	57.630	49.639	51.714	1.00	0.00	C
ATOM	5578	HD2	LYS	357	57.091	48.920	52.332	1.00	0.00	H
ATOM	5579	HD3	LYS	357	57.227	49.597	50.701	1.00	0.00	H
ATOM	5580	CE	LYS	357	59.121	49.273	51.687	1.00	0.00	C
ATOM	5581	HE2	LYS	357	59.662	49.984	51.059	1.00	0.00	H
ATOM	5582	HE3	LYS	357	59.495	49.353	52.708	1.00	0.00	H
ATOM	5583	NZ	LYS	357	59.339	47.905	51.186	1.00	0.00	N
ATOM	5584	HZ1	LYS	357	60.302	47.613	51.209	1.00	0.00	H
ATOM	5585	HZ2	LYS	357	58.921	47.748	50.282	1.00	0.00	H
ATOM	5586	HZ3	LYS	357	58.835	47.252	51.800	1.00	0.00	H
ATOM	5587	C	LYS	357	58.728	54.489	52.822	1.00	0.00	C
ATOM	5588	O	LYS	357	59.003	55.638	52.479	1.00	0.00	O
ATOM	5589	N	GLY	358	59.013	54.162	54.088	1.00	0.00	N
ATOM	5590	H	GLY	358	59.446	54.924	54.590	1.00	0.00	H
ATOM	5591	CA	GLY	358	58.586	53.025	54.916	1.00	0.00	C
ATOM	5592	HA2	GLY	358	58.366	53.415	55.911	1.00	0.00	H
ATOM	5593	HA3	GLY	358	57.648	52.624	54.544	1.00	0.00	H
ATOM	5594	C	GLY	358	59.605	51.887	55.121	1.00	0.00	C
ATOM	5595	O	GLY	358	59.294	50.934	55.822	1.00	0.00	O
ATOM	5596	N	LYS	359	60.818	51.976	54.546	1.00	0.00	N
ATOM	5597	H	LYS	359	60.966	52.744	53.909	1.00	0.00	H
ATOM	5598	CA	LYS	359	61.950	51.053	54.822	1.00	0.00	C
ATOM	5599	HA	LYS	359	61.793	50.631	55.816	1.00	0.00	H
ATOM	5600	CB	LYS	359	61.983	49.889	53.798	1.00	0.00	C
ATOM	5601	HB2	LYS	359	61.552	50.231	52.857	1.00	0.00	H
ATOM	5602	HB3	LYS	359	63.014	49.602	53.581	1.00	0.00	H
ATOM	5603	CG	LYS	359	61.243	48.623	54.273	1.00	0.00	C
ATOM	5604	HG2	LYS	359	60.251	48.898	54.627	1.00	0.00	H
ATOM	5605	HG3	LYS	359	61.122	47.950	53.424	1.00	0.00	H
ATOM	5606	CD	LYS	359	61.989	47.856	55.376	1.00	0.00	C
ATOM	5607	HD2	LYS	359	62.816	47.290	54.944	1.00	0.00	H
ATOM	5608	HD3	LYS	359	62.410	48.560	56.094	1.00	0.00	H
ATOM	5609	CE	LYS	359	61.053	46.922	56.152	1.00	0.00	C
ATOM	5610	HE2	LYS	359	61.577	46.555	57.039	1.00	0.00	H
ATOM	5611	HE3	LYS	359	60.198	47.496	56.523	1.00	0.00	H
ATOM	5612	NZ	LYS	359	60.560	45.754	55.383	1.00	0.00	N
ATOM	5613	HZ1	LYS	359	61.303	45.145	55.089	1.00	0.00	H

ATOM	5614	HZ2	LYS	359	59.975	45.998	54.584	1.00	0.00	H
ATOM	5615	HZ3	LYS	359	59.977	45.189	56.012	1.00	0.00	H
ATOM	5616	C	LYS	359	63.330	51.723	54.938	1.00	0.00	C
ATOM	5617	O	LYS	359	64.295	51.028	55.227	1.00	0.00	O
ATOM	5618	N	ALA	360	63.424	53.041	54.726	1.00	0.00	N
ATOM	5619	H	ALA	360	62.579	53.548	54.544	1.00	0.00	H
ATOM	5620	CA	ALA	360	64.657	53.835	54.855	1.00	0.00	C
ATOM	5621	HA	ALA	360	64.429	54.829	54.470	1.00	0.00	H
ATOM	5622	CB	ALA	360	64.978	54.008	56.351	1.00	0.00	C
ATOM	5623	HB1	ALA	360	65.268	53.050	56.787	1.00	0.00	H
ATOM	5624	HB2	ALA	360	64.108	54.399	56.880	1.00	0.00	H
ATOM	5625	HB3	ALA	360	65.804	54.708	56.479	1.00	0.00	H
ATOM	5626	C	ALA	360	65.863	53.344	54.006	1.00	0.00	C
ATOM	5627	O	ALA	360	67.012	53.646	54.332	1.00	0.00	O
ATOM	5628	N	GLU	361	65.618	52.613	52.914	1.00	0.00	N
ATOM	5629	H	GLU	361	64.662	52.449	52.648	1.00	0.00	H
ATOM	5630	CA	GLU	361	66.631	51.900	52.118	1.00	0.00	C
ATOM	5631	HA	GLU	361	67.614	52.315	52.337	1.00	0.00	H
ATOM	5632	CB	GLU	361	66.624	50.420	52.560	1.00	0.00	C
ATOM	5633	HB2	GLU	361	66.705	50.367	53.646	1.00	0.00	H
ATOM	5634	HB3	GLU	361	65.653	50.000	52.291	1.00	0.00	H
ATOM	5635	CG	GLU	361	67.710	49.515	51.949	1.00	0.00	C
ATOM	5636	HG2	GLU	361	67.341	48.487	51.991	1.00	0.00	H
ATOM	5637	HG3	GLU	361	67.856	49.760	50.896	1.00	0.00	H
ATOM	5638	CD	GLU	361	69.051	49.576	52.699	1.00	0.00	C
ATOM	5639	OE1	GLU	361	69.439	50.669	53.175	1.00	0.00	O
ATOM	5640	OE2	GLU	361	69.719	48.520	52.816	1.00	0.00	O
ATOM	5641	C	GLU	361	66.326	52.056	50.608	1.00	0.00	C
ATOM	5642	O	GLU	361	65.158	51.923	50.225	1.00	0.00	O
ATOM	5643	N	PRO	362	67.319	52.357	49.744	1.00	0.00	N
ATOM	5644	CD	PRO	362	68.726	52.563	50.072	1.00	0.00	C
ATOM	5645	HD2	PRO	362	69.130	51.736	50.655	1.00	0.00	H
ATOM	5646	HD3	PRO	362	68.836	53.498	50.624	1.00	0.00	H
ATOM	5647	CG	PRO	362	69.461	52.666	48.736	1.00	0.00	C
ATOM	5648	HG2	PRO	362	69.745	51.669	48.394	1.00	0.00	H
ATOM	5649	HG3	PRO	362	70.336	53.313	48.804	1.00	0.00	H
ATOM	5650	CB	PRO	362	68.394	53.247	47.810	1.00	0.00	C
ATOM	5651	HB2	PRO	362	68.598	53.011	46.765	1.00	0.00	H
ATOM	5652	HB3	PRO	362	68.339	54.328	47.951	1.00	0.00	H
ATOM	5653	CA	PRO	362	67.100	52.598	48.315	1.00	0.00	C
ATOM	5654	HA	PRO	362	66.279	53.304	48.187	1.00	0.00	H
ATOM	5655	C	PRO	362	66.772	51.321	47.524	1.00	0.00	C
ATOM	5656	O	PRO	362	67.293	50.234	47.793	1.00	0.00	O
ATOM	5657	N	LEU	363	65.938	51.460	46.490	1.00	0.00	N
ATOM	5658	H	LEU	363	65.524	52.375	46.337	1.00	0.00	H
ATOM	5659	CA	LEU	363	65.538	50.387	45.576	1.00	0.00	C
ATOM	5660	HA	LEU	363	65.733	49.419	46.033	1.00	0.00	H
ATOM	5661	CB	LEU	363	64.021	50.488	45.316	1.00	0.00	C
ATOM	5662	HB2	LEU	363	63.820	51.447	44.838	1.00	0.00	H
ATOM	5663	HB3	LEU	363	63.734	49.706	44.612	1.00	0.00	H
ATOM	5664	CG	LEU	363	63.132	50.382	46.570	1.00	0.00	C
ATOM	5665	HG	LEU	363	63.421	51.136	47.302	1.00	0.00	H

ATOM	5666	CD1	LEU	363	61.676	50.639	46.179	1.00	0.00	C
ATOM	5667	HD11	LEU	363	61.585	51.650	45.780	1.00	0.00	H
ATOM	5668	HD12	LEU	363	61.037	50.552	47.056	1.00	0.00	H
ATOM	5669	HD13	LEU	363	61.362	49.926	45.419	1.00	0.00	H
ATOM	5670	CD2	LEU	363	63.223	48.998	47.219	1.00	0.00	C
ATOM	5671	HD21	LEU	363	63.003	48.223	46.486	1.00	0.00	H
ATOM	5672	HD22	LEU	363	64.227	48.848	47.616	1.00	0.00	H
ATOM	5673	HD23	LEU	363	62.520	48.934	48.047	1.00	0.00	H
ATOM	5674	C	LEU	363	66.332	50.456	44.261	1.00	0.00	C
ATOM	5675	O	LEU	363	66.033	51.287	43.404	1.00	0.00	O
ATOM	5676	N	ASN	364	67.321	49.575	44.077	1.00	0.00	N
ATOM	5677	H	ASN	364	67.558	48.956	44.843	1.00	0.00	H
ATOM	5678	CA	ASN	364	68.033	49.411	42.803	1.00	0.00	C
ATOM	5679	HA	ASN	364	68.339	50.396	42.450	1.00	0.00	H
ATOM	5680	CB	ASN	364	69.297	48.552	42.992	1.00	0.00	C
ATOM	5681	HB2	ASN	364	69.040	47.633	43.504	1.00	0.00	H
ATOM	5682	HB3	ASN	364	69.688	48.301	42.008	1.00	0.00	H
ATOM	5683	CG	ASN	364	70.405	49.213	43.790	1.00	0.00	C
ATOM	5684	OD1	ASN	364	70.213	49.704	44.887	1.00	0.00	O
ATOM	5685	ND2	ASN	364	71.611	49.231	43.272	1.00	0.00	N
ATOM	5686	HD21	ASN	364	71.810	48.740	42.416	1.00	0.00	H
ATOM	5687	HD22	ASN	364	72.330	49.657	43.831	1.00	0.00	H
ATOM	5688	C	ASN	364	67.102	48.803	41.737	1.00	0.00	C
ATOM	5689	O	ASN	364	66.402	47.819	42.004	1.00	0.00	O
ATOM	5690	N	LEU	365	67.116	49.382	40.533	1.00	0.00	N
ATOM	5691	H	LEU	365	67.760	50.154	40.390	1.00	0.00	H
ATOM	5692	CA	LEU	365	66.055	49.255	39.531	1.00	0.00	C
ATOM	5693	HA	LEU	365	65.394	48.434	39.814	1.00	0.00	H
ATOM	5694	CB	LEU	365	65.261	50.574	39.584	1.00	0.00	C
ATOM	5695	HB2	LEU	365	64.935	50.737	40.614	1.00	0.00	H
ATOM	5696	HB3	LEU	365	65.933	51.391	39.319	1.00	0.00	H
ATOM	5697	CG	LEU	365	64.031	50.651	38.669	1.00	0.00	C
ATOM	5698	HG	LEU	365	64.337	50.580	37.626	1.00	0.00	H
ATOM	5699	CD1	LEU	365	63.038	49.534	38.988	1.00	0.00	C
ATOM	5700	HD11	LEU	365	63.365	48.602	38.529	1.00	0.00	H
ATOM	5701	HD12	LEU	365	62.056	49.793	38.600	1.00	0.00	H
ATOM	5702	HD13	LEU	365	62.968	49.396	40.065	1.00	0.00	H
ATOM	5703	CD2	LEU	365	63.344	51.999	38.888	1.00	0.00	C
ATOM	5704	HD21	LEU	365	62.991	52.071	39.916	1.00	0.00	H
ATOM	5705	HD22	LEU	365	64.045	52.811	38.691	1.00	0.00	H
ATOM	5706	HD23	LEU	365	62.500	52.103	38.209	1.00	0.00	H
ATOM	5707	C	LEU	365	66.609	48.956	38.126	1.00	0.00	C
ATOM	5708	O	LEU	365	67.548	49.619	37.671	1.00	0.00	O
ATOM	5709	N	TYR	366	66.011	47.973	37.443	1.00	0.00	N
ATOM	5710	H	TYR	366	65.242	47.480	37.884	1.00	0.00	H
ATOM	5711	CA	TYR	366	66.548	47.365	36.218	1.00	0.00	C
ATOM	5712	HA	TYR	366	67.264	48.060	35.782	1.00	0.00	H
ATOM	5713	CB	TYR	366	67.307	46.066	36.562	1.00	0.00	C
ATOM	5714	HB2	TYR	366	66.579	45.315	36.863	1.00	0.00	H
ATOM	5715	HB3	TYR	366	67.790	45.693	35.658	1.00	0.00	H
ATOM	5716	CG	TYR	366	68.350	46.166	37.664	1.00	0.00	C
ATOM	5717	CD1	TYR	366	69.726	46.230	37.359	1.00	0.00	C

```
ATOM   5718  HD1 TYR   366      70.049  46.209  36.327  1.00  0.00           H
ATOM   5719  CE1 TYR   366      70.680  46.290  38.398  1.00  0.00           C
ATOM   5720  HE1 TYR   366      71.737  46.314  38.177  1.00  0.00           H
ATOM   5721  CZ  TYR   366      70.256  46.303  39.745  1.00  0.00           C
ATOM   5722  OH  TYR   366      71.155  46.334  40.763  1.00  0.00           O
ATOM   5723  HH  TYR   366      70.845  45.798  41.511  1.00  0.00           H
ATOM   5724  CE2 TYR   366      68.882  46.262  40.042  1.00  0.00           C
ATOM   5725  HE2 TYR   366      68.555  46.304  41.064  1.00  0.00           H
ATOM   5726  CD2 TYR   366      67.936  46.159  39.009  1.00  0.00           C
ATOM   5727  HD2 TYR   366      66.885  46.092  39.251  1.00  0.00           H
ATOM   5728  C   TYR   366      65.474  47.099  35.141  1.00  0.00           C
ATOM   5729  O   TYR   366      64.276  47.060  35.421  1.00  0.00           O
ATOM   5730  N   GLU   367      65.946  46.903  33.911  1.00  0.00           N
ATOM   5731  H   GLU   367      66.958  46.925  33.820  1.00  0.00           H
ATOM   5732  CA  GLU   367      65.240  46.831  32.622  1.00  0.00           C
ATOM   5733  HA  GLU   367      64.189  46.584  32.777  1.00  0.00           H
ATOM   5734  CB  GLU   367      65.337  48.252  32.007  1.00  0.00           C
ATOM   5735  HB2 GLU   367      64.741  48.922  32.628  1.00  0.00           H
ATOM   5736  HB3 GLU   367      66.374  48.579  32.084  1.00  0.00           H
ATOM   5737  CG  GLU   367      64.896  48.449  30.544  1.00  0.00           C
ATOM   5738  HG2 GLU   367      65.540  47.853  29.894  1.00  0.00           H
ATOM   5739  HG3 GLU   367      63.867  48.099  30.435  1.00  0.00           H
ATOM   5740  CD  GLU   367      65.015  49.912  30.090  1.00  0.00           C
ATOM   5741  OE1 GLU   367      65.917  50.637  30.575  1.00  0.00           O
ATOM   5742  OE2 GLU   367      64.232  50.370  29.227  1.00  0.00           O
ATOM   5743  C   GLU   367      65.875  45.724  31.736  1.00  0.00           C
ATOM   5744  O   GLU   367      66.925  45.177  32.088  1.00  0.00           O
ATOM   5745  N   ILE   368      65.257  45.380  30.595  1.00  0.00           N
ATOM   5746  H   ILE   368      64.432  45.898  30.317  1.00  0.00           H
ATOM   5747  CA  ILE   368      65.766  44.449  29.559  1.00  0.00           C
ATOM   5748  HA  ILE   368      66.853  44.399  29.626  1.00  0.00           H
ATOM   5749  CB  ILE   368      65.183  43.021  29.740  1.00  0.00           C
ATOM   5750  HB  ILE   368      64.129  43.053  29.455  1.00  0.00           H
ATOM   5751  CG2 ILE   368      65.918  42.053  28.793  1.00  0.00           C
ATOM   5752 HG21 ILE   368      65.834  42.399  27.763  1.00  0.00           H
ATOM   5753 HG22 ILE   368      65.474  41.060  28.835  1.00  0.00           H
ATOM   5754 HG23 ILE   368      66.973  42.006  29.062  1.00  0.00           H
ATOM   5755  CG1 ILE   368      65.266  42.506  31.198  1.00  0.00           C
ATOM   5756 HG12 ILE   368      66.311  42.476  31.509  1.00  0.00           H
ATOM   5757 HG13 ILE   368      64.739  43.199  31.853  1.00  0.00           H
ATOM   5758  CD1 ILE   368      64.626  41.130  31.431  1.00  0.00           C
ATOM   5759 HD11 ILE   368      63.607  41.123  31.041  1.00  0.00           H
ATOM   5760 HD12 ILE   368      64.595  40.927  32.502  1.00  0.00           H
ATOM   5761 HD13 ILE   368      65.208  40.347  30.948  1.00  0.00           H
ATOM   5762  C   ILE   368      65.446  45.018  28.168  1.00  0.00           C
ATOM   5763  O   ILE   368      64.355  45.615  28.026  1.00  0.00           O
ATOM   5764  OXT ILE   368      66.259  44.868  27.225  1.00  0.00           O
TER    5765      ILE   368
END
```
